# Accurate multiple sequence alignment of transmembrane proteins with PSI-Coffee

**DOI:** 10.1186/1471-2105-13-S4-S1

**Published:** 2012-03-28

**Authors:** Jia-Ming Chang, Paolo Di Tommaso, Jean-François Taly, Cedric Notredame

**Affiliations:** 1 Bioinformatics and Genomics program, Centre for Genomic Regulation (CRG) and UPF, Barcelona 08003, Spain

## Abstract

**Background:**

Transmembrane proteins (TMPs) constitute about 20~30% of all protein coding genes. The relative lack of experimental structure has so far made it hard to develop specific alignment methods and the current state of the art (PRALINE™) only manages to recapitulate 50% of the positions in the reference alignments available from the BAliBASE2-ref7.

**Methods:**

We show how homology extension can be adapted and combined with a consistency based approach in order to significantly improve the multiple sequence alignment of alpha-helical TMPs. TM-Coffee is a special mode of PSI-Coffee able to efficiently align TMPs, while using a reduced reference database for homology extension.

**Results:**

Our benchmarking on BAliBASE2-ref7 alpha-helical TMPs shows a significant improvement over the most accurate methods such as MSAProbs, Kalign, PROMALS, MAFFT, ProbCons and PRALINE™. We also estimated the influence of the database used for homology extension and show that highly non-redundant UniRef databases can be used to obtain similar results at a significantly reduced computational cost over full protein databases. TM-Coffee is part of the T-Coffee package, a web server is also available from http://tcoffee.crg.cat/tmcoffee and a freeware open source code can be downloaded from http://www.tcoffee.org/Packages/Stable/Latest.

## Background

Transmembrane proteins (TMPs) are non-soluble proteins anchored in a cell membrane and containing one or more membrane-spanning segments separated with intra or extra-cellular domains of variable length. This figure reflects the bi-layer membrane width, though the segments can also be tilted within the membrane, thus requiring more amino acids to span the interval (up to 30). TMPs constitute about 20~30% of all protein coding genes in prokaryotic and eukaryotic organisms [1,2]. The problem of generating multiple sequence alignments (MSAs) of TMPs was first addressed by [3], and over the last years several packages have been published, specifically designed for that task [4-6]. To our knowledge PRALINE™ is the only TMPs multiple aligner currently available. PRALINE™ belongs to a category of aligners using the process of homology extension. PROMALS [7] and PSI-Coffee [8] also belong to this category. Homology extension is a method that involves using database searches to replace each sequence with a profile made of close homologues. As a result, in any sequence, each position becomes a column in a multiple alignment, thus reflecting the pattern of acceptable mutations. These patterns are very informative, as they tend to reflect the sum of constraints (mostly functional and structural) that have shaped the diversity observed along the proteins of the same family. A natural consequence of these patterns conservation is the high sensitivity of profile-profile comparisons when doing remote homology search [9].

In this paper we show that the PSI-Coffee homology extension can also be used to reveal and use specific conservation patterns of TMPs like the amphiphilic properties of transmembrane alpha helices and thus yield significant improvements when aligning TMPs. A critical parameter when doing homology extension is to determine the influence of the database used to make the extension. Typical homology extension involves performing BLAST or PSI-BLAST [10] against the NR database [11]. This step is time consuming and results in a prohibitive cost for homology extension, as compared with faster methods. We show here that one can go over this problem by using smaller non-redundant databases. We go even further by showing that a TMP specific database can be used for homology extension at a much lower CPU cost and without any significant reduction in alignment accuracy.

## Methods

### Homology extension

The process of homology extension involves replacing individual sequences with a set of multiply aligned homologues. Given a dataset, this procedure involves performing BLAST for each individual sequence against a protein database and turning the resulting output into a one-against-all MSA (i.e. query against hits). These MSAs (one per sequence in the original dataset) are then turned into profiles. The purpose of homology extension is to reveal the evolutionary variability associated with each site of the considered sequences thus producing more accurate pair-wise alignments [7]. We used blast+ (version 2.2.25) against various databases (see next section). In practice, homology extension is made automatically by T-Coffee [12] either using the public web service maintained by the European Bioinformatics Institute (default) or using a locally installed BLAST against locally maintained databases.

### Databases

Homology extension was carried out against two databases: NR and UniRef [13]. In order to check the effect of redundancy, we used the versions of UniRef non-redundant database (UniRef100, UniRef90 and UniRef50) trimmed at various levels of redundancy. In these versions, the database is modified so as to make sure that no pair of sequences exists with an identity higher than the specified level. UniRef100-TM, UniRef90-TM and UniRef50-TM are even smaller databases produced by filtering the corresponding UniRef dataset with the following query string: "keyword:transmembrane". These TMP specific databases are typically 20% of the size of their sources.

### TM-Coffee algorithm

TM-Coffee uses the PSI-Coffee (Position Specific Iterative T-Coffee) mode of T-Coffee to multiply align TMPs. The algorithm can be summarized as follows:

1. Perform BLAST for each query sequence against the selected database with default parameters.

2. Keep hits having a level of identity between 50% and 90% and a coverage higher than 70%.

3. Turn the BLAST output into a profile by removing all columns corresponding to positions unaligned to the query (i.e. gaps in the query) and by filling with gaps query positions unmatched by BLAST.

4. Produce a T-Coffee library by aligning every pair of profiles with a pair-HMM. When doing so, every pair of matched column with a posterior probability of being aligned higher than 0.99 is added to the library. The pair-HMM is adapted from the ProbCons pair-HMM [14] in order to deal with profiles. It uses the ProbCons bi-phasic gap penalty set (i.e. two distinct sets of gap opening and extension penalties for short and longer gaps). The parameter values are those initially reported [14].

### Benchmarking

We used as a gold standard the reference 7 of BAliBASE2 [15]. This dataset is made of 435 alpha-helical TMPs classified into eight distinct families that can be multiply aligned. The core regions of BAliBASE defined by the authors examine the alignment of structurally equivalent residues only (Additional file 1). Evaluation is made by assessing the capacity of the methods to recapitulate these core regions, mostly made of alpha helices. Two metrics are used to assess accuracy: the Sum of Pairs score (SP) that estimates the fraction of residue pairs from the reference core identically aligned in the target and the reference MSA and the Total Column score (TC) that estimates the fraction of columns identically aligned in the target and the reference.

### Aligners

We used BAliBASE-ref7 dataset to compare PSI-Coffee with the six most accurate methods currently available, MSAProbs 0.9.4 [16], Kalign 2.04 [17], PROMALS, MAFFT 6.815 [18], ProbCons 1.12 and PRALINE™. All methods were run using default parameters. PSI-Coffee is part of the T-Coffee suite (Version 8.99). It was run with default parameters except for the database used for homology extension. This was done with the following command line:

t_coffee <seq.fasta> -mode psicoffee -blast_server LOCAL -protein_db <database> -template_file PSITM

**Figure 1:**
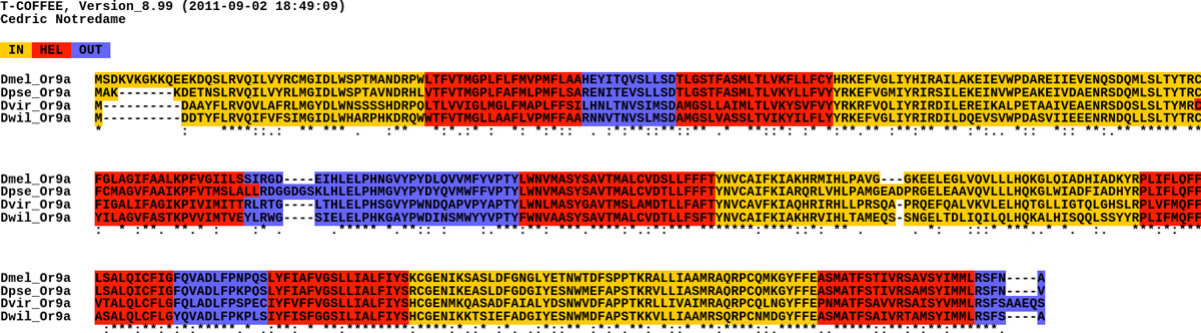
**Typical colour output (tm_html)**. In this example, the protein Or9a of *Drosophila melanogaster *and its orthologues of other *Drosophila *species were aligned with PSITM template. The colour code corresponds to prediction by HMMTOP, where *yellow*: in loop, *red*: TM helix, *blue*: out loop. Notably, the predicted topology of the Or9a set is consistent with the Benton *et al*.'s conclusion [20].

The PSITM template file mode is used here to display a coloured MSA version (.tm_html output file, Figure 1) reflecting predictions carried out by HMMTOP [19] using the profile associated with each sequence. This prediction is only used for display purposes and is not required by the alignment procedure.

## Results

We first asked whether applying the PSI-Coffee homology extension algorithm on our reference dataset of TMPs could lead to some improvement over existing alignment methods. We did so using the NR database for homology extension. Results (Table 1) show that TM-Coffee outperforms the other methods, most notably when considering entire columns (TC comparison). When doing so, we find an improvement of nearly 10% over PRALINE™. Owing to the small dataset size (eight families), the observed differences are not highly statistically significant, although the differences between PSI-Coffee and the other methods are consistently more marked than the differences between the other methods (Table 2). This increased accuracy comes, however, at a significant computational cost. One may therefore argue that the over-head for turning single sequences into profiles is so significant that it is not worth using this approach for large-scale analysis. In order to address this problem we asked whether one could achieve a similar level of accuracy while doing the homology extension on smaller databases.

**Table 1:** Comparison between the PSI-Coffee and other multiple sequence alignment methods on each BAliBASE2-ref7 family

family	MSAProbs	Kalign	PROMALS	MAFFT	ProbCons	PRALINE™	PSI-Coffee
SP							
7TM	0.981	0.938	0.985	0.962	0.978	0.983	**0.986**
Nat	0.789	0.765	**0.815**	0.797	0.777	0.732	0.779
ACR	0.989	0.969	0.964	**0.994**	0.989	0.987	0.992
DTD	0.972	0.961	0.965	0.975	0.967	0.960	**0.977**
ION	0.817	0.810	0.761	0.788	**0.862**	0.837	0.783
MSL	0.965	0.936	**1.000**	0.980	0.958	0.986	0.971
PHOTO	0.957	0.928	0.954	0.949	0.957	**0.965**	0.955
PTGA	0.899	0.826	0.863	0.886	0.903	0.808	**0.926**

avg	0.921	0.892	0.913	0.916	**0.924**	0.907	0.921
Pairs	3,117,244	3,014,033	3,109,227	3,093,269	3,108,377	3,080,356	**3,124,007**

TC							
7TM	0.600	0.360	**0.690**	0.440	0.550	0.560	0.620
Nat	0.190	0.190	0.100	0.110	0.180	0.180	**0.250**
ACR	0.830	0.620	0.530	**0.890**	0.830	0.810	0.880
DTD	0.540	0.580	0.400	0.540	0.520	0.580	**0.620**
ION	0.270	0.130	0.260	0.260	**0.320**	0.000	0.210
MSL	0.910	0.850	**1.000**	0.950	0.900	0.960	0.930
PHOTO	0.510	0.440	0.510	0.510	0.540	**0.690**	0.520
PTGA	**0.400**	0.320	0.280	0.370	**0.400**	0.270	**0.400**

avg	0.531	0.436	0.471	0.509	0.530	0.506	**0.554**
Cols	1,066	863	814	1,057	1,054	1,058	**1,146**

SP and TC are the accuracy figures reported by BAliScore 3.01 based on core regions (Additional file 1). The rows, *Pairs *and *Cols*, denote the sum of corrected aligned pairs and columns, respectively. The number of pairs and columns in the reference alignments are 3,294,102 and 1,781, respectively. The best performance of each family is marked in bold.

**Table 2:** Statistical significance test of the performance between two methods

SP	MSAProbs	Kalign	PROMALS	MAFFT	ProbCons	PRALINE™	PSI-Coffee
MSAProbs	NA	**0.014**	0.547	0.483	0.675	0.779	0.726
Kalign	**0.014**	NA	0.195	**0.039**	**0.008**	0.195	0.078
PROMALS	0.547	0.195	NA	0.575	0.742	0.742	0.528
MAFFT	0.483	**0.039**	0.575	NA	0.889	0.844	0.779
ProbCons	0.675	**0.008**	0.742	0.889	NA	0.461	0.262
PRALINE™	0.779	0.195	0.742	0.844	0.461	NA	0.641
PSI-Coffee	0.726	0.078	0.528	0.779	0.262	0.641	NA

TC	MSAProbs	Kalign	PROMALS	MAFFT	ProbCons	PRALINE™	PSI-Coffee

MSAProbs	NA	**0.035**	0.150	0.529	1.000	0.779	0.204
Kalign	**0.035**	NA	0.742	0.078	0.055	0.272	**0.014**
PROMALS	0.150	0.742	NA	0.529	0.362	0.624	0.233
MAFFT	0.529	0.078	0.529	NA	0.362	0.945	0.262
ProbCons	1.000	0.055	0.362	0.362	NA	1.000	0.353
PRALINE™	0.779	0.272	0.624	0.945	1.000	NA	0.195
PSI-Coffee	0.204	**0.014**	0.233	0.262	0.353	0.195	NA

Wilcoxon Signed-Rank Test (R: wilcoxon.test, paired = TRUE)

NULL hypothesis: *x *and *y *have identical performance

0.05 significance level: *p*-value smaller than 0.05 marked in bold, reject null hypothesis

When using PSI-Coffee, profiles are built by performing BLAST search for each sequence against NR. This procedure defines the database as a key ingredient of homology extension. It is therefore an interesting question to ask how this parameter may affect the overall accuracy of the procedure. We did so by providing PSI-Coffee with databases of various redundancy levels (UniRefXX), all built upon UniProt, and then realigning the reference datasets. Results (Table 3 and 4, detailed performance per family in Additional file 2) show that the difference in accuracy is very small when comparing to NR. Overall, the accuracy level of PSI-Coffee remains high regardless of the redundancy level. In practice, however, using a UniRef50 means using a database with 50% redundancy and approximately 3.5 times smaller than the full database. As one would expect the CPU requirements of the extension process decrease accordingly and the time required by the alignment goes down to 26,442 as compared with the 72,199 seconds required when using the full database (2.7 times faster).

**Table 3:** Performance comparison of different database sizes for the BAliBASE2-ref7

database	# of seqs	SP	TC	extension(s)	total(s)
default T-Coffee	0	0.911	0.498	0	2,735
UniRef50-TM	87,989	0.916	0.561	1,483	8,177
UniRef90-TM	263,306	0.918	0.548	3,343	9,610
UniRef100-TM	613,015	0.925	0.545	6,499	12,111
UniProt-TM	818,635	0.923	0.536	7,871	13,285
UniRef50	3,077,464	0.920	0.553	19,087	26,442
UniRef90	6,544,144	0.924	0.561	40,448	46,478
UniRef100	9,865,668	0.922	0.554	66,696	71,895
UniProt	11,009,767	0.923	0.563	66,964	72,199
NCBI NR	10,565,004	0.921	0.554	65,201	70,375

*# of seqs *indicates the number of sequences contained in the considered database subset. *extension *indicates the CPU time required by the homology extension process. *total *indicates the total CPU time usage.

**Table 4:** Statistical significance test of the performance in different databases

SP	default	UniRef50-TM	UniRef90-TM	UniRef100-TM	UniProt-TM	UniRef50	UniRef90	UniRef100	UniProt	NR
default	NA	0.447	0.141	**0.039**	**0.042**	0.195	**0.039**	**0.035**	**0.034**	**0.035**
UniRef50-TM	0.447	NA	0.833	0.675	0.483	0.410	0.141	0.446	0.160	0.446
UniRef90-TM	0.141	0.833	NA	0.161	0.395	0.799	0.074	0.172	0.205	0.495
UniRef100-TM	**0.039**	0.675	0.161	NA	0.713	0.834	0.674	0.202	0.786	0.293
UniProt-TM	**0.042**	0.483	0.395	0.713	NA	0.933	0.752	0.735	0.892	0.779
UniRef50	0.195	0.410	0.799	0.834	0.933	NA	0.447	0.461	0.553	0.483
UniRef90	**0.039**	0.141	0.074	0.674	0.752	0.447	NA	1.000	0.598	0.430
UniRef100	**0.035**	0.446	0.172	0.202	0.735	0.461	1.000	NA	0.798	0.203
UniProt	**0.034**	0.160	0.205	0.786	0.892	0.553	0.598	0.798	NA	0.528
NR	**0.035**	0.446	0.495	0.293	0.779	0.483	0.430	0.203	0.528	NA

TC	default	UniRef50-TM	UniRef90-TM	UniRef100-TM	UniProt-TM	UniRef50	UniRef90	UniRef100	UniProt	NR

default	NA	**0.035**	**0.021**	0.092	0.148	**0.042**	0.050	0.050	**0.039**	**0.034**
UniRef50-TM	**0.035**	NA	0.834	0.281	0.207	1.000	0.799	0.396	1.000	0.396
UniRef90-TM	**0.021**	0.834	NA	0.855	0.584	0.865	0.281	0.672	0.462	0.892
UniRef100-TM	0.092	0.281	0.855	NA	0.174	0.798	0.554	0.916	0.423	0.832
UniProt-TM	0.148	0.207	0.584	0.174	NA	0.611	0.397	0.396	0.178	0.402
UniRef50	**0.042**	1.000	0.865	0.798	0.611	NA	0.674	0.832	0.670	1.000
UniRef90	0.050	0.799	0.281	0.554	0.397	0.674	NA	0.581	1.000	0.588
UniRef100	0.050	0.396	0.672	0.916	0.396	0.832	0.581	NA	0.528	1.000
UniProt	**0.039**	1.000	0.462	0.423	0.178	0.670	1.000	0.528	NA	0.528
NR	**0.034**	0.396	0.892	0.832	0.402	1.000	0.588	1.000	0.528	NA

Wilcoxon Signed-Rank Test (R: wilcoxon.test, paired = TRUE)

NULL hypothesis: *x *and *y *have identical performance

0.05 significance level: *p*-value smaller than 0.05 marked in bold, reject null hypothesis

Even so, the CPU requirements can be considered excessive when compared with the time needed by the default T-Coffee (that does not need to do any extension), we therefore decided to take advantage of the observation that when doing homology extension with TMPs, one spends a lot of CPU time searching databases mostly made of unlikely TMPs homologues. Indeed, 80% of the proteins in UniRef are non TMPs. We therefore asked if a simple database, built by filtering UniRef on keywords could be used instead of the full DB. This database, named UniRefXX-TM is significantly more compact than its source. UniRef50-TM contains about 100 times fewer sequences than the full UniProt. Results obtained on this database show that using such a reduced protein set for the extension does not result in any trade-off between accuracy and efficiency. The level accuracy is comparable and even superior to that achieved with the default PSI-Coffee while the CPU time requirements are dramatically decreased by a factor 10. We named TM-Coffee the flavour of PSI-Coffee running homology extension against UniRef50-TM. Table 5 shows that TM-Coffee remains relatively slower than non-homology extension based methods but dramatically faster than PROMALS, a well-known aligner using homology extension.

**Table 5:** Comparison of running time

	MSAProbs	Kalign	PROMALS	MAFFT	ProbCons	T-Coffee	TM-Coffee	
							extension	alignment
7TM	759.42	0.94	19,587.13	14.03	711.32	1,164.66	488.04	2,684.41
Nat	244.02	0.60	13,285.11	43.51	269.07	243.42	175.40	395.88
ACR	548.96	1.13	19,102.74	41.73	620.30	418.28	174.24	1,130.15
DTD	343.45	0.71	12,555.83	70.90	396.70	335.35	187.64	949.92
ION	413.04	1.06	10,034.86	75.68	498.60	364.19	203.80	931.70
MSL	0.75	0.01	646.19	0.12	0.72	0.84	16.72	9.17
PHOTO	16.73	0.06	2,388.75	1.66	15.40	18.05	55.89	25.85
PTGA	202.76	0.48	9,745.36	21.75	217.93	190.35	181.00	567.69

SUM	2,529.13	4.99	87,345.97	269.37	2,730.04	2,735.14	1,482.73	6,694.77

The PRALINE™ is run through its web server (standalone version not available), so the comparison does not include PRALINE™. MSAProbs is measured by using single threaded for comparison with TM-Coffee in single core. The unit is second.

**Figure 2:**
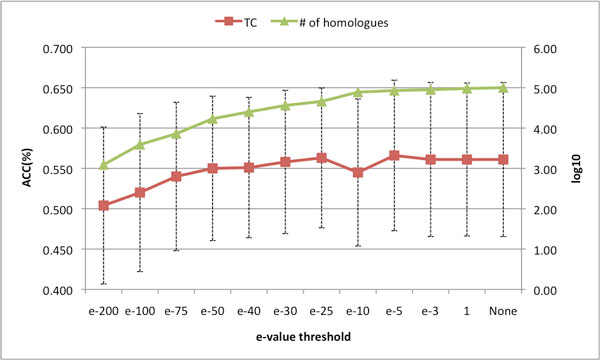
**Line chart of the average TC respect to different *e*-value thresholds on UniRef50-TM database**. The number of homologues is counted by summing all homologues found in eight families and plotted in log_10 _scale. The standard error of TC score cross eight families is the range of dash line. SP is skipped due to minor change respect to different *e*-value thresholds.

We finally asked whether one can alter the effect of homology extension by filtering the BLAST output based on *e*-value and removing distantly related sequences (i.e. remote homologues) that are likely to be inaccurately or only partially aligned. Results are shown in Figure 2. As one can see, no benefit is gained from filtering the BLAST output and the overall accuracy tends to increase when more hits are included. The slight drop on the curve is not statistically significant and can be attributed to a single misaligned sequence. Overall this result indicates that using the default BLAST parameters (NONE in Figure 2) leads to profiles where low quality hits only have a negligible impact on the overall alignment accuracy, probably because remote homologues tend to be filtered out through their low coverage.

## Discussion and conclusions

In this work we show that homology extension can be used to significantly increase the accuracy of transmembrane protein multiple sequence alignments. When considering entire columns (the most stringent measure of multiple alignment accuracy), our results suggest that PSI-Coffee is about 10% more accurate than MSAProbs, the next best method. This improvement comes, however, at a cost and we show that the default PSI-Coffee requires about 30 times more CPU time than simpler methods. We therefore explored the possibility of using more compact non-redundant databases and found that when using a database trimmed to 50% redundancy and containing only sequences annotated as TMPs, we could achieve the same level of accuracy as PSI-Coffee while only requiring a tenth of the CPU time. This new protocol is named TM-Coffee.

Regardless of the improvement reported here in terms of CPU, TM-Coffee remains a relatively slow method. One may argue whether the increased computation cost is worth the improvement reported here. There is no simple answer to this question. For instance, if we consider Kalign and TM-Coffee, the difference in CPU requirement is about a thousand fold. The difference in accuracy at the column level, however, is about 28%. These are major differences, bound to dramatically affect any modelling based on an MSA. Of course, one may argue that the column score can be affected by a single misaligned sequence and is therefore an amplification of reality. This is probably true for some applications of MSAs, yet many circumstances exist like homology or phylogenetic modelling where the misalignment of a single sequence can have a major impact on the conclusion drawn upon the analysis of a dataset.

## List of abbreviations used

TMPs: transmembrane proteins; MSAs: multiple sequence alignments; SP: the Sum of Pairs score; TC: the Total Column score.

## Competing interests

The authors declare that they have no competing interests.

## Authors' contributions

JMC and CN conceived of the experiments and drafted the manuscript. JFT and PDT constructed web service and open source code. All authors read and approved the final manuscript.

## Supplementary Material

Additional file 1 **The core region of BAliBASE 2**. For BAliBASE 2, authors did not publish the XML file allowing automated use of these blocks. The location of the block is only available in HTML file, the uppercase of character (i.e., http://bips.u-strasbg.fr/en/Products/Databases/BAliBASE2/ref7/test/msl_ref7.html). We have generated the XML following the original BAliBASE annotation. Click here for file

Additional file 2 **The performance of each TMP family by individual database**. *default *means T-Coffee without homology extension. Others are PSI-Coffee searching against corresponding databases. The construction of databases is explained in "Methods" section. Click here for file
