# Supplementary material for: Accurate multiple sequence alignment of transmembrane proteins with PSI-Coffee
Source: BMC Bioinformatics. 2012 Mar 28;13(Suppl 4):S1. doi: 10.1186/1471-2105-13-S4-S1 (PMC3303701; doi:10.1186/1471-2105-13-S4-S1)
Supplement: Additional file 1 — The core region of BAliBASE 2. For BAliBASE 2, authors did not publish the XML file allowing automated use of these blocks. The location of the block is only available in HTML file, the uppercase of character (i.e., http://bips.u-strasbg.fr/en/Products/Databases/BAliBASE2/ref7/test/msl_ref7.html). We have generated the XML following the original BAliBASE annotation. [file 1471-2105-13-S4-S1-S1.pdf]

The core region of BAliBASE 2

For BAliBASE 2, authors did not publish the XML file allowing automated use of these blocks. The location of the block is only available in HTML file, the uppercase of character (i.e., http://bips.u-strasbg.fr/en/Products/Databases/BAliBASE2/ref7/test/msl_ref7.html). We have generated the XML following the original BAliBASE annotation (<column-score>) and are now providing these files as the following.

::::::::::::::
7tm_core-block.xml
::::::::::::::
<?xml version="1.0"?>
<!DOCTYPE macsim SYSTEM "http://www-bio3d-igbmc.u-strasbg.fr/macsim.dtd">
<macsim>
<alignment>
<aln-name>1</aln-name>
<sequence seq-type="Protein">
<seq-name>grhr_claga</seq-name>
<seq-info><length>1066</length></seq-info>
<seq-data>--MSG
NTTLLLSNPTNVLDNSSVLNVSVSPPVLKWETPTF----TTAARFRVAATLVLFVFRAASNLSVLLSVTRGRGRRLAS-HLRPLIA
SLASADLVMTFVVMPLDAVWNVTV--QWYAGDA------MCKLMCFLKLFAMHSAAFILVVVSLDRHHAILHPLDTL----DA
GRRNRRMLLTAWILSLLLASPQLFIFRAIKAKGVD---FVQCATHGSF---------------
---QQH
WQETAYNMFHFVTLYVFPLLVMSLCYTRILVEINRQMHRSKDKAGEP---

---------------------CLRRSGTDMIPKARMKT-LKMTIIIVASFVICWTPYYLLGIWYWFQ--------PQMLHVIPDYVHHVFFVFG
NLNTCCDPVIYGFFTPSFRADLSRCFCWRNQNASAKSLPHFSGHRREVSGEAESDLGSG---DQPSGQ--------------------------
---</seq-data>
</sequence>
<sequence seq-type="Protein">
<seq-name>v1ar_rat</seq-name>
<seq-info><length>1066</length></seq-info>
<seq-data>---MSFPRGSQD
RSVGNSSPWWPLTTEGSNGSQEAARLGEG-DSPLGDVRN----EELAKLEIAVLAVIFVVAVLGNSSVLLALHRTPRKTS---R
MHLFIRHLSLADLAVAFFQVLPQLCWDITS--SFRGPDW------LCRVVKHLQVFAMFASAYMLVVMTADRYIAVCHPLKTL-
--QQPARRSRLMIATSWVLSFILSTPQYFIFSVIEIEVNNGTK---TQDCWATFIQ-----
----
----PWGTRAYVTWMTSGVFVAPVVVLGTCYGFICYHIWRNIRGKTASSRHSKGDKGSGEAVGPFH----------------------------
----
------------------------------------KGLLVTPCVSSVKSISRAKIRT-VKMTFVIVSAYILCWAPFFIVQMWSVWD-------ENFIWTDS
ENPSITITALLASLNSCCNPWIYMFFSGHLLQDCVQSFPCCHSMAQKFAKDDSDSMSRRQTSYSNNRSPTNSTGMWKDS
PKSSKSIRFIPVST---</seq-data>

</sequence>
<sequence seq-type="Protein">
<seq-name>oxyr_mouse</seq-name>
<seq-info><length>1066</length></seq-info>
<seq-data>---ME
GTPAANWSIELDLGSG-VPPGAEGNLTAGPPRRN----EALARVEVAVLCLILFLALSGNACVLLALRTTRHKHS---RLFFFMK
HLSIADLVVAVFQVLPQLLWDITF--RFYGPDL------LCRLVKYLQVVGMFASTYLLLLMSLDRCLAICQPLRSL-----RRRTDRL
AVLATWLGCLVASVPQVHIFSLREVADG--VFDCWAVFIQ------------------------
---PWGPKA
YVTWITLAVYIVPVIVLAACYGLISFKIWQNLRLKTAAAAAAAEGSDAAGG--

-----------AGRAALARVSSVKLISKAKIRT-VKMTFIIVLAFIVCWTPFFFVQMWSVWD----------VNAPKEASAFIIAMLLASL
NSCCNPWIYMLFTGHLFHELVQRFLCCSARYLKGSRPGETSISKKSNSSTFVLSRCSSSQRSCSQPSSA---------------------------
--</seq-data>
</sequence>
<sequence seq-type="Protein">
<seq-name>5h1b_human</seq-name>
<seq-info><length>1066</length></seq-info>
<seq-data>---MEEPGAQ
CAPPPPAGSETWVPQANLSSAPSQNCSAKD-YIYQDSIS---LPWKVLLVMLLALITLATTLSNAFVIATVYRTRKLHT---PANY
LIASLAVTDLLVSILVMPISTMYTVTG--RWTLGQV------VCDFWLSSDITCCTASILHLCVIALDRYWAITDAVEYS--AKRTPK
RAAVMIALVWVFSISISLPPFF--WRQAKA--EEEVSECVVNTDH------------------
---ILY
TVYSTVGAFYFPTLLLIALYGRIYVEARSRILKQTPNRTGKRLTRAQLITDSPGSTSSVTSINSRVPDVPSES--------GSPVYVNQ
VKVRVS--
---------------------------------------DALLEKKKLMAARERKA-TKTLGIILGAFIVCWLPFFIISLVMPIC-------KDAC--WFHLAIFD
FFTWLGYLNSLINPIIYTMSNEDFKQAFHKLIRFKCTS---
--</seq-data>
</sequence>
<sequence seq-type="Protein">
<seq-name>5h1b_rabit</seq-name>
<seq-info><length>1066</length></seq-info>
<seq-data>---MEEPGAQ
CAPPLAAGSQIAVPQANLSAAHSHNCSAEG-YIYQDSIA---LPWKVLLVLLLALFTLATTLSNAFVVATVYRTRKLHT---PANY
LIASLAVTDLLVSILVMPISTMYTVTG--RWTLGQV------VCDLWLSSDITCCTASIMHLCVIALDRYWAITDAVEYS--AKRTP
KRAAIMIRLVWVFSICISLPPFF--WRQAKA--EEEVSECLVNTDH------------------
---VL
YTVVYSTVGAFYLPTLLLIALYGRIYVEARSRILKQTPNRTGKRLTRAQLITDSPGSTTSVTSINSRAPDVPSES--------GSPVYVN
QVKVRVS---
--DALLEKKKLMAARERKA-TKTLGIILGVFIVCWLPFFIISLVMPIC-------KDAC--WFHQAIF
DFFTWLGYVNSLINPIIYTMSNEDFKQAFHKLIRFKCTS---
--</seq-data>

</sequence>
<sequence seq-type="Protein">
<seq-name>5h1b_mouse</seq-name>
<seq-info><length>1066</length></seq-info>
<seq-data>---MEEQGIQ
CAPPPPAASQTGVPLTNLS----HNCSADG-YIYQDSIA---LPWKVLLVALLALITLATTLSNAFVIATVYRTRKLHT---PANYLIA
SLAVTDLLVSILVMPISTMYTVTG--RWTLGQV------VCDFWLSSDITCCTASIMHLCVIALDRYWAITDAVEYS--AKRTPKR
AAIMIVLVWVFSISISLPPFF--WRQAKA---EEEMLDCFVNTDH--------------------
--VLYT
VYSTVGAFYLPTLLLIALYGRIYVEARSRILKQTPNKTGKRLTRAQLITDSPGSTSSVTSINSRAPDVPSES--------GSPVYVNQV
KVRVS---
------------------------------------DALLEKKKLMAARERKA-TKTLGIILGAFIVCWLPFFIISLVMPIC-------KDAC--WFHMAIFDF
FNWLGYLNSLINPIIYTMSNEDFKQAFHKLIRFKCAG---
---</seq-data>
</sequence>
<sequence seq-type="Protein">
<seq-name>5h1b_spaeh</seq-name>
<seq-info><length>1066</length></seq-info>
<seq-data>---MEEPGAR
CAPPPPAGSQTQTPSSNLS----HNCSADS-YIYQDSIA---LPWKVLLVALLALITLATTLSNAFVIATVYRTRKLHT---PANYLIA
SLAVTDLLVSILVMPISTMYTVTG--RWTLGQV------VCDFWLSSDITCCTASIMHLCVIALDRYWAITDAVEYS--AKRTPRR
AAVMIALVWVFSISISLPRFF--WRQAKA---EEEVLDCLVNTDH--------------------
--VLYT
VYSTVGAFYLPTLLLIALYGRIYVEARSRILKQTPNKTGKRLSRAQLISDSPGSTSSVTSINSRVPDVPSES--------GSPVYVNQV
KVRVS---
------------------------------------DALLEKKKLMAARERKA-TKTLGIILGAFIVCWLPFFIISLVMPIC-------KDAC--WFHMAIFDF
FNWLGYLNSLINPIIYTMPNEDFKQAFHKLIRFKCTG---
---</seq-data>
</sequence>
<sequence seq-type="Protein">
<seq-name>5h1b_cavpo</seq-name>
<seq-info><length>1066</length></seq-info>
<seq-data>---MGNPEAS
CTPPAVLGSQTGLPHANVS-APPNNCSAPS-HIYQDSIA---LPWKVLLVVLLALITLATTLSNAFVIATVYRTRKLHT---PANYL
IASLAFTDLLVSILVMPISTMYTVTG--RWTLGQA------LCDFWLSSDITCCTASIMHLCVIALDRYWAITDAVGYS--AKRTPR
RAAGMIALVWVFSICISLPPFF--WRQAKA---EEEVLDCLVNTDH------------------
--VL
YTVYSTGGAFYLPTLLLIALYGRIYVEARSRILKQTPNKTGKRLTRAQLITDSPGSTSSVTSINSRAPEVPCDS--------GSPVYVN
QVKVRVS--
---DALLEKKKLMAARERKA-TKTLGVILGAFIVCWLPFFIISLVMPIC-------KDAC--WFHMAI
FDFFTWLGYLNSLINPIIYTMSNEDFKQAFHKLIRFKCTT---
--</seq-data>

</sequence>
<sequence seq-type="Protein">
<seq-name>5h1b_didma</seq-name>
<seq-info><length>1066</length></seq-info>
<seq-data>---MEQPSRL
CSPP-ASGSLTSS-QTNHSTFPNPNCSAPDLEPYQDSIA---LPWKVLLATFLGLITLGTTLSNAFVIATVSRTRKLHT---PANYLI
ASLAVTDLLVSILVMPISTMYTVTG--RWTLGQV------VCDFWLSSDITCCTASILHLCVIALDRYWAITDAVEYS--AKRTPKR
AAGMIIMVWVFSVSISMPPLF--WRQAKA---EEVADCSVNTDH-----------------
---IL
YTVYSTVGAFYFPTLLLIALYGRIYVEARSRILKQTPNRTGKRLTRAQLITDSPGSSSSGTSINSRAPEGPSES--------GSPVYVN
QVKVKVS--
---DALLEKKKLMAARERKA-TRTLGIILGAFIVCWLPFFIISLALPIC-------DDAC--WFHLAIFD
FFNWLGYLNSLINPIIYTKSNDDFKQAFQKLMRFRRTS--
--</seq-data>
</sequence>
<sequence seq-type="Protein">
<seq-name>5h1d_human</seq-name>
<seq-info><length>1066</length></seq-info>
<seq-data>--MSPL
NQSAEGLPQEASNRS----LNATETS-EAWDPRTL---QALKISLAVVLSVITLATVLSNAFVLTTILLTRKLHT---PANYLIGSLAT
TDLLVSILVMPISIAYTITH--TWNFGQI------LCDIWLSSDITCCTASILHLCVIALDRYWAITDALEYS--KRRTAGHAATMIAIV
WAISICISIPPLF--WRQAKA---QEEMSDCLVNTSQ------------------------------------
--ISYTIYSTCGAFY
IPSVLLIILYGRIYRAARNRILNP-PSLYGKRFTTAHLITGSAG--SSLCSLNSSLHEGHSHSA-------GSPLFFNHVKIKLA----------
----
----------------DSALERKRISAARERKA-TKILGIILGAFIICWLPFFVVSLVLPIC-------RDSC--WIHPALFDFFTWLGYLNSLINPII
YTVFNEEFRQAFQKIVPFRKAS--
--</seq-data>
</sequence>
<sequence seq-type="Protein">
<seq-name>aaf61808</seq-name>
<seq-info><length>1066</length></seq-info>
<seq-data>--MSP
PNQSVEDLLQGTSNRS----LNATETP-KAWDPGTL---QVLKISLAVVLSIITLATVLSNAFVLTTIFLTRKLHT---PANYLIGSLA
MTDLLVSILVMPISIPYTITQ--TWSFGQL------LCDIWLSSDITCCTASILHLCVIALDRYWAITDALEYS--KRRTAGHAAAMI
AIVWAISICISIPPLF--WRQARA---HEEISDCLVNTSQ---------------------------------
--ISYTIYSTCGAF
YIPSLLLIILYGRIYRAARNRILNP-PSLYGKRFTTAHLITGSAG--SSLCSLNPSLHEEHAHSA-------GSPLFFNHVKIKLA---------
----
-----------------DSVLERKRISAARERKA-TKTLGIILGAFIICWLPFFVASLVLPIC-------RDSC--WIHPALFDFFTWLGYLNSLINP
IIYTVFNEEFRQAFQKVVHFRKAS---
---</seq-data>

</sequence>
<sequence seq-type="Protein">
<seq-name>5h1d_rat</seq-name>
<seq-info><length>1066</length></seq-info>
<seq-data>--MSLP
NQSLEGLPQEASNRS----LNAT----GAWDPEVL---QALRISLVVVLSIITLATVLSNAFVLTTILLTKKLHT---PANYLIGSLATTD
LLVSILVMPISIAYTTTR--TWNFGQI------LCDIWVSSDITCCTASILHLCVIALDRYWAITDALEYS--KRRTAGHAAAMIAAV
WAISICISIPPLF--WRQATA---HEEMSDCLVNTSQ--------------------------------
---ISYTIYSTCGAFYI
PSILLIILYGRIYVAARSRILNP-PSLYGKRFTTAQLITGSAG--SSLCSLNPSLHESHTHTV-------GSPLFFNQVKIKLA------------
----
-----------DSILERKRISAARERKA-TKTLGIILGAFIICWLPFFVVSLVLPIC-------RDSC--WIHPALFDFFTWLGYLNSLINPVIY
TVFNEDFRQAFQRVVHFRKAS---
--</seq-data>
</sequence>
<sequence seq-type="Protein">
<seq-name>5h1d_cavpo</seq-name>
<seq-info><length>1066</length></seq-info>
<seq-data>--MSP
PNQSEEGLPQEASNRS----LNATETP-GDWDPGLL---QALKVSLVVVLSIITLATVLSNAFVLTTILLTRKLHT---PANYLIGSLA
TTDLLVSILVMPISIAYTTTR--TWNFGQI------LCDIWVSSDITCCTASILHLCVIALDRYWAITDALEYS--KRRTAGHAGAMI
AAVWVISICISIPPLF--WRQAQA--QEEMSDCLVNTSQ--------------------------
--ISYTIYSTC
GAFYIPSVLLIILYSRIYRAARSRILNP-PSLSGKRFTTAHLITGSAG--SSLCSLNPSLHEGHMHP--------GSPLFFNHVRIKLA----
----
--------------------DSVLERKRISAARERKA-TKTLGIILGAFIVCWLPFFVVSLVLPIC-------RDSC--WIHPALFDFFTWLGYLNS
LINPIIYTVFNEDFRQAFQKVVHFRKAS--
---</seq-data>
</sequence>
<sequence seq-type="Protein">
<seq-name>5h1d_rabit</seq-name>
<seq-info><length>1066</length></seq-info>
<seq-data>--MSPS
NQSAEGLPQEAANRS----LNATGTP-EAWDPGTL---QALKISLAVVLSIITVATVLSNTFVLTTILLTRKLHT---PANYLIGSLAT
TDLLVSILVMPISIAYTITH--TWNFGQV------LCDIWVSSDITCCTASILHLCVIALDRYWAITDALEYS--KRRTAGHAAAMIA
VVWAISICISIPPLF--WRQAKA---HEEVSDCLVNTSQ-----------------------------
--ISYTIYSTCGAF
YIPSVLLIVLYGRIYMAARNRILNP-PSLYGKRFTTAHLITGSAG--SSLCSLSPSLGEGHSHSA-------GSPLFFNPVRIKLA-------
----
-----------------DSVLERKRISAARERKA-TKTLGIILGAFIGCWLPFFVASLVLPIC-------RDSC--WMPPGLFDFFTWLGYLNS
LINPIIYTVFNEDFRQAFQRVIHFRKAF--
--</seq-data>

</sequence>
<sequence seq-type="Protein">
<seq-name>5h1d_canfa</seq-name>
<seq-info><length>1066</length></seq-info>
<seq-data>--MSP
PNQSLEGLLQEASNRS----LNATETP-EAWGPETL---QALKISLALLLSIITMATALSNAFVLTTIFLTRKLHT---PANYLIGSLA
MTDLLVSILVMPISIAYTTTR--TWSFGQI------LCDIWLSSDITCCTASILHLCVIALDRYWAITDALEYS--KRRTAGRAAVMIA
TVWVISICISIPPLF--WRQAKA---QEDMSDCQVNTSQ-----------------------------
--ISYTIYSTCG
AFYIPSVLLIILYGRIYVAARNRILNP-PSLYGKRFTTAQLITGSAG--SSLCSLSPSLQEERSHAA-------GPPLFFNHVQVKLA----
----
--------------------EGVLERKRISAARERKA-TKTLGIILGAFIVCWLPFFVASLVLPIC-------RASC--WLHPALFDFFTWLGYLN
SLINPIIYTVFNEEFRQAFQRVVHVRKAS--
---</seq-data>
</sequence>
<sequence seq-type="Protein">
<seq-name>5h1d_fugru</seq-name>
<seq-info><length>1066</length></seq-info>
<seq-data>--MEL
DNNSLDYFSSNFTDIP----SNTTV---AHWTEATL---LGLQISVSVVLAIVTLATMLSNAFVIATIFLTRKLHT---PANFLIGSLAV
TDMLVSILVMPISIVYTVSK--TWSLGQI------VCDIWLSSDITFCTASILHLCVIALDRYWAITDALEYS--KRRTMRRAAVMV
AVVWVISISISMPPLF--WRQAKA---HEELKECMVNTDQ--------------------------
---ISYTLYSTF
GAFYVPTVLLIILYGRIYVAARSRIFKT-PSYSGKRFTTAQLIQTSAG--SSLCSLNSASNQEAHLHSGAGGEGGGSPLFVNSVK
VKLA---
--------------------------------DNVLERKRLCAARERKA-TKTLGIILGAFIICWLPFFVVTLVWAIC--------KEC--SFDPLLFDVFT
WLGYLNSLINPVIYTVFNDEFKQAFQKLIKFRR--
---</seq-data>
</sequence>
<sequence seq-type="Protein">
<seq-name>5h1e_human</seq-name>
<seq-info><length>1066</length></seq-info>
<seq-data>--
-------------MNITNCTTEA-SMAIRPKT---ITEKMLICMTLVVITTLTTLLNLAVIMAIGTTKKLHQ---PANYLICSLAVTDLLVAV
LVMPLSIIYIVMD--RWKLGYF------LCEVWLSVDMTCCTCSILHLCVIALDRYWAITNAIEYA--RKRTAKRAALMILTVWTISI
FISMPPLF--WRSHRR--LSPPPSQCTIQHDH---
--VIYTIYSTLGAFYIPLTLI
LILYYRIYHAAKSLYQKRGSSRHLSNRSTDSQNSFAS---CKLTQTFCVSDFSTSDP--------TTEFEKFHASIRIPPFDNDL---------
----
----------DHPGERQQISSTRERKA-ARILGLILGAFILSWLPFFIKELIVGLS---------IY--TVSSEVADFLTWLGYVNSLINPLLYTS
FNEDFKLAFKKLIRCREHT---
--</seq-data>

</sequence>
<sequence seq-type="Protein">
<seq-name>5h1f_human</seq-name>
<seq-info><length>1066</length></seq-info>
<seq-data>--
-------------MDFLNSSDQNLTSEELLNR---MPSKILVSLTLSGLALMTTTINSLVIAAIIVTRKLHH---PANYLICSLAVTDFLVAV
LVMPFSIVYIVRE--SWIMGQV------VCDIWLSVDITCCTCSILHLSAIALDRYRAITDAVEYA--RKRTPKHAGIMITIVWIISVF
ISMPPLF--WRHQG--TSRDDECIIKHDH---
---IVSTIYSTFGAFYIPLALILIL
YYKIYRAAKTLYHKRQASRIAKEEVNGQVLLESGEKSTKSVSTSYVLEKSLSDP--------STDFDKIHSTVRSLRSEFKH-----------
----
--------EKSWRRQKISGTRERKA-ATTLGLILGAFVICWLPFFVKELVVNVC--------DKC--KISEEMSNFLAWLGYLNSLINPLIY
TIFNEDFKKAFQKLVRCRC--
---</seq-data>
</sequence>
<sequence seq-type="Protein">
<seq-name>5h1f_cavpo</seq-name>
<seq-info><length>1066</length></seq-info>
<seq-data>--
-------------MDFLNSSDQNLTSEELLHR---MPSKILVSLTLSGLALMTTTINSLVIAAIIVTRKLHH---PANYLICSLAVTDFLVAV
LVMPFSIVYIVRE--SWIMGQV------LCDIWLSVDIICCTCSILHLSAIALDRYRAITDAVEYA--RKRTPKQAGIMITIVWIISVFI
SMPPLF--WRHQG--TSRDDECIIKHDH--
--IVSTIYSTFGAFYIPLVLILIL
YYKIYKAAKTLYHKRQASRIAKEELNGQVLLESGEKSIKMVSTTYVPEKSLSDP--------STDFDKIHNTVKSPRCKLRH----------
----
---------EKSWRRQKISGTRERKA-ATTLGLILGAFVICWLPFFVKELVVNVC--------EKC--KISEEMANFLAWLGYLNSLINPLI
YTIFNEDFKKAFQKLVRCQY--
---</seq-data>
</sequence>
<sequence seq-type="Protein">
<seq-name>5h1f_mouse</seq-name>
<seq-info><length>1066</length></seq-info>
<seq-data>--
-------------MDFLNASDQNLTSEELLNR---MPSKILVSLTLSGLALMTTTINSLVIAAIIVTRKLHH---PANYLICSLAVTDFLVAV
LVMPFSIVYIVRE--SWIMGQV------LCDIWLSVDIICCTCSILHLSAIALDRYRAITDAVEYA--RKRTPRHAGIMITIVWVISVFI
SMPPLF--WRHQG--TSRDDECVIKHDH--
---IVSTIYSTFGAFYIPLVLILIL
YYKIYRAARTLYHKRQASRMIKEELNGQVFLESGEKSIKLVSTSYMLEKSLSDP--------STDFDRIHSTVKSPRSELKH----------
----
---------EKSWRRQKISGTRERKA-ATTLGLILGAFVICWLPFFVKELVVNVC--------EKC--KISEEMSNFLAWLGYLNSLINPLI
YTIFNEDFKKAFQKLVRCRY---
---</seq-data>

</sequence>
<sequence seq-type="Protein">
<seq-name>5h1a_fugru</seq-name>
<seq-info><length>1066</length></seq-info>
<seq-data>--MDLR
ATSSNDSNATSGYSDTAAVDWDEGENATGSGSLPDPE---LSYQIITSLFLGALILCSIFGNSCVVAAIALERSLQN---VANYLI
GSLAVTDLMVSVLVLPMAALYQVLN--KWTLGQD------ICDLFIALDVLCCTSSILHLCAIALDRYWAITDPIDYV--NKRTPRR
AAVLISVTWLIGFSISIPPML-GWRS---AEDRANPDACIISQDP----------------------
--GYTI
YSTFGAFYIPLILMLVLYGRIFKAARFRIRKTVKKTEKAKASDMCLTLSPAVFHKRANGDAVSAEWKRGYKFKPS-SPCANGA
VRHGEEMESLEIIEVN--SNSKTHLPLPNTP-QSSSHE---
--NINEKTTGTRRKIALARERKT-VKTLGIIMGTFIFCWLPFFIVALVLPFC------
-AENC--YMPEWLGAVINWLGYSNSLLNPIIYAYFNKDFQSAFKKILRCKFHRH---
--</seq-data>
</sequence>
<sequence seq-type="Protein">
<seq-name>5h1b_fugru</seq-name>
<seq-info><length>1066</length></seq-info>
<seq-data>--
---MEGTNNTTGWTHFDSTSNRTSKSFDEEVK---LSYQVVTSFLLGALILCSIFGNACVVAAIALERSLQN---VANYLIGSLAVT
DLMVSVLVLPMAALYQVLN--RWTLGQI------PCDIFISLDMLCCTSSILHLCVIALDRYWAITEPIDYM--KKRTPRRAAVLIS
VTWLVGFSISIPPML-IMRSQPSSM--AEDRANSKQCKITQDP---------------------------
--WYTIYS
TFGAFYIPLTLMLVLYGRIFKAARFRIRRTVRKTEKKKVSDTCLALSPAMFHRKTPGDAHGKSWKRSVEPRP--LPNVNGAVK
HAGEGESLDIIEVQ--SNSRCNLPLPNTPGTVPLFE--
---NRHEKATETKRKIALARERKT-VKTLGIIMGTFILCWLPFFIVALVMPFC-------
QESC--FMPHWLKDVINWLGYSNSLLNPIIYAYFNKDFQSAFKKIIKCHFCRA--
---</seq-data>
</sequence>
<sequence seq-type="Protein">
<seq-name>5h1a_rat</seq-name>
<seq-info><length>1066</length></seq-info>
<seq-data>---
MDVFSFGQGNNTTASQEPFGTGGNVTSISDVT----FSYQVITSLLLGTLIFCAVLGNACVVAAIALERSLQN---VANYLIGSLA
VTDLMVSVLVLPMAALYQVLN--KWTLGQV------TCDLFIALDVLCCTSSILHLCAIALDRYWAITDPIDYV--NKRTPRRAAA
LISLTWLIGFLISIPPML-GWRT---PEDRSDPDACTISKDH-----------------------------
--GYTIYSTF
GAFYIPLLLMLVLYGRIFRAARFRIRKTVRKVEKKGAGTSLGTSSAPPPKKSLNGQPGSGDWRRCAENRAVGTPCTNGAVR
QGDDEATLEVIEVHRVGNSKEHLPLPSESGSNSYAPACLE--
---RKNERNAEAKRKMALARERKT-VKTLGIIMGTFILCWLPFFIVALVLPFC
-------ESSC--HMPALLGAIINWLGYSNSLLNPVIYAYFNKDFQNAFKKIIKCKFCRR---
--</seq-data>

</sequence>
<sequence seq-type="Protein">
<seq-name>5h1a_mouse</seq-name>
<seq-info><length>1066</length></seq-info>
<seq-data>--
MDMFSLGQGNNTTTSLEPFGTGGNDTGLSNVT----FSYQVITSLLLGTLIFCAVLGNACVVAAIALERSLQN---VANYLIGSL
AVTDLMVSVLVLPMAALYQVLN--KWTLGQV------TCDLFIALDVLCCTSSILHLCAIALDRYWAITDPIDYV--NKRTPRRAA
ALISLTWLIGFLISIPPML-GWRA---PEDRSNPNECTISKDH-------------------------
---GYTIYST
FGAFYIPLLLMLVLYGRIFRAARFRIRKTVKKVEKKGAGTSFGTSSAPPPKKSLNGQPGSGDCRRSAENRAVGTPCANGAVR
QGEDDATLEVIEVHRVGNSKGDLPLPSESGATSYVPACLE---
--RKNERTAEAKRKMALARERKT-VKTLGIIMGTFILCWLPFFIVALVLPFC
-------ESSC--HMPELLGAIINWLGYSNSLLNPVIYAYFNKDFQNAFKKIIKCKFCR---
--</seq-data>
</sequence>
<sequence seq-type="Protein">
<seq-name>5h1a_human</seq-name>
<seq-info><length>1066</length></seq-info>
<seq-data>--
MDVLSPGQGNNTTSPPAPFETGGNTTGISDVT----VSYQVITSLLLGTLIFCAVLGNACVVAAIALERSLQN---VANYLIGSLA
VTDLMVSVLVLPMAALYQVLN--KWTLGQV------TCDLFIALDVLCCTSSILHLCAIALDRYWAITDPIDYV--NKRTPRRAAA
LISLTWLIGFLISIPPML-GWRT---PEDRSDPDACTISKDH---------------------------
---GYTIYSTF
GAFYIPLLLMLVLYGRIFRAARFRIRKTVKKVEKTGADTRHGASPAPQPKKSVNGESGSRNWRLGVESKAGGALCANGAVR
QGDDGAALEVIEVHRVGNSKEHLPLPSEAGPTPCAPASFE--
---RKNERNAEAKRKMALARERKT-VKTLGIIMGTFILCWLPFFIVALVLPF
C-------ESSC--HMPTLLGAIINWLGYSNSLLNPVIYAYFNKDFQNAFKKIIKCKFCRQ---
---</seq-data>
</sequence>
<sequence seq-type="Protein">
<seq-name>q98998</seq-name>
<seq-info><length>1066</length></seq-info>
<seq-data>---
----MDASNNTTSWNILQRGRMG-PSWRRCP----VSYQIIASLFLGRSFSAGIFGNACVIAAIALERSLQN---VANYLIGSLAVT
DLMVSVLVLPMAAQNQVLN--KWTLGQV------TCDIFISLDVLCCTSSILHLCAIALDRYWAITDPIDYV--NKRTPRRAAVLIS
ITWIVGFSISIPPML-GWRT---PEDRSDPNACRISEDP------------------------------
--GYTIYSTFGA
FYIPLILMLVLYGKIFKAARFRIRKTVKKAEKKKVADTCLSVSQQSPKEKQRGAQQELEEVGGAQAQR----CVNGAIRHGEEG
AVLEIIEVHHYVNSKCHLHCKPVPPPEQLPPA--
--LKNDRATEAKRKVALARERKT-VKTLGIIMGTFILCWLPFFIVALVLPFC--------ETC
--HMPHLLFDIITWLGYSNSLLNPIIYAYFNKDFQSAFKKIIKCKFCRQ--
--</seq-data>

</sequence>
<sequence seq-type="Protein">
<seq-name>5h7_human</seq-name>
<seq-info><length>1066</length></seq-info>
<seq-data>--MMDVNSSGRPDLYGHLRSFLLPEVGR
GLPDLSPDGGADPVAGSWAPHLLS----EVTASPAPTWDAPPDNASGCGEQINYG--RVEKVVIGSILTLITLLTIAGNCLVVIS
VCFVKKLRQ---PSNYLIVSLALADLSVAVAVMPFVSVTDLIG-GKWIFGHF------FCNVFIAMDVMCCTASIMTLCVISIDRYL
GITRPLTYP--VRQNGKCMAKMILSVWLLSASITLPPLF-GWAQN---VNDDKV
CLISQD---
-----------------------FGYTIYSTAVAFYIPMSVMLFMYYQIYKAARKSAAKHKFPGFPRVEPDSVIALNGIVKLQKEVEECANL
SRLLKHE--
--RKNISIFKREQKA-ATTLGIIVGAFTVCWLPFFLLSTARPFI------CGTS
C-SCIPLWVERTFLWLGYANSLINPFIYAFFNRDLRTTYRSLLQCQYRNINRKLSAAGMHEALKLAERPERPEFVLQNADYCR
KKGHDS---</seq-data>
</sequence>
<sequence seq-type="Protein">
<seq-name>5h7_rat</seq-name>
<seq-info><length>1066</length></seq-info>
<seq-data>--MMDVNSSGRPDLYGHLRSLILPEVGRG
LQDLSPDGGAHPVVSSWMPHLLSGFL-EVTASPAPTWDAPPDNVSGCGEQINYG--RVEKVVIGSILTLITLLTIAGNCLVVIS
VCFVKKLRQ---PSNYLIVSLALADLSVAVAVMPFVSVTDLIG-GKWIFGHF------FCNVFIAMDVMCCTASIMTLCVISIDRYL
GITRPLTYP--VRQNGKCMAKMILSVWLLSASITLPPLF-GWAQN---VNDDKV
CLISQD---
-----------------------FGYTIYSTAVAFYIPMSVMLFMYYQIYKAARKSAAKHKFPGFPRVQPESVISLNGVVKLQKEVEECAN
LSRLLKHE--
--RKNISIFKREQKA-ATTLGIIVGAFTVCWLPFFLLSTARPFI------CG
TSC-SCIPLWVERTCLWLGYANSLINPFIYAFFNRDLRTTYRSLLQCQYRNINRKLSAAGMHEALKLAERPERSEFVLQNSDH
CGKKGHDT---</seq-data
>
</sequence>
<sequence seq-type="Protein">
<seq-name>5h7_cavpo</seq-name>
<seq-info><length>1066</length></seq-info>
<seq-data>--MMGVNSSGRPDLYGHLHSILLP--GRG
LPDWSPDGGADPGVSTWTPRLLSGVP-EVAASPSPSWDGTWDNVSGCGEQINYG--RAEKVVIGSILTLITLLTIAGNCLVV
ISVCFVKKLRQ---PSNYLIVSLALADLSVAVAVIPFVSVTDLIG-GKWIFGHF------FCNVFIAMDVMCCTASIMTLCVISIDRYL
GITRPLTYP--VRQNGKCMPKMILSVWLLSASITLPPLF-GWAQN---VNDDKV
CLISQD---
-----------------------FGYTIYSTAVAFYIPMSVMLFMYYRIYKAARKSAAKHKFPGFPRVQPESIISLNGMVKLQKEVEECANL
SRLLKHE--
--RKNISIFKREQKA-ATTLGIIVGAFTVCWLPFFLLSTARPFI------CGT
AC-SCIPLWVERTCLWLGYANSLINPFIYAFFNRDLRTTYRSLLQCQYRNINRKLSAAGMHEALKLAERPERPECVLQNSDYC

RKKGHDS---</seq-data>
</sequence>
<sequence seq-type="Protein">
<seq-name>5ht1_drome</seq-name>
<seq-info><length>1066</length></seq-info>
<seq-data>MALSGQDWRRHQSHRQHRNHRTQGNHQKLISTATLTLFVLFLSSWIAYAAGK-ATVPAPLVEGETESATSQ
DFNSSSAFLGAIASASSTGSGSGSGSGSGSGSGSGSYGLASMNSSPIAIVSYQGITSSNLGDSNTTLVPLSDTPLLLEEFAAGE
FVLP---PLTSIFVSIVLLIVILGTVVGNVLVCIAVCMVRKLRR---PCNYLLVSLALSDLCVALLVMPMALLYEVLE--KWNFGPL--
----LCDIWVSFDVLCCTASILNLCAISVDRYLAITKPLEYG--VKRTPRRMMLCVGIVWLAAACISLPPLL-ILGNEHE------------
--DEEGQPICTVCQNF---
---AYQIYATLGSFYIPLSVMLFVYYQIFRAARRIVLEEKRA
QTHLQQALNGTGSPSAPQAPPLGHTELASSGNGQRHSSVGNTSLTYSTCGGLSSGGGALAGHGSGGGVSGSTGL---------
---L
GSPHHKKLRFQLAKEKKA-STTLGIIMSAFTVCWLPFFILALIRPFE----------T-MHVPASLSSLFLWLGYANSLLNPIIYATLNR
DFRKPFQEILYFRCSSLNTMMRENYYQDQYGEPPSQRVMLGDERHGARESFL---
---</seq-data>
</sequence>
<sequence seq-type="Protein">
<seq-name>o15970</seq-name>
<seq-info><length>1066</length></seq-info>
<seq-data>---
MSTINSTNCTFYFQLASKNNSAVQ-VPFYCIKFS--FEFEVFLIIVLTIFLVGTAGGNLLVISSVAIVKKLQT---SSNFLIVNLACSD
FLVSILVLPGAVHQVIYR-GQWPFSEI------LCDIFISFDVILCTSSILNLCAISIDRYLVITRPLQYA--VNRTPARIGTMVAISWV
TSALISIPPMF-GLKETFI---PGQCNYSDNL---
---IYQIYATFGAFYIPLIV
MLILYGRIFKLAREMAQNDAKLKIGISPNSSDKEQHSHLRIVDSHICNTPNGQMNLSLYSGSLCSISKSNNLSDSSP-----------
----
---------------NKVNLRNRVKLKSSTETKA-ITTLGVIMGCFTICWLPFFLIQISKPVL---KVANVDSM-NYFPIWLIELCLWLGYF
NSFLNPIIYAKFNREFRTPFKQILLCKCNSINARLRSETFVQEYGLPNSSAPTKRGSFDQTSLNNGSFTKNRRRRTPGVLRQNP
HLNNCKIENSRKIYECSLINKTNKFPPESSVLLNSIANGNTKPVKKDTDDNPFHFSDQISPNCSINDQRSEKMFMILVQHLNL
----------------</seq-data>
</sequence>
<sequence seq-type="Protein">
<seq-name>o15969</seq-name>
<seq-info><length>1066</length></seq-info>
<seq-data>--MWKLPFVFIIIS
NSFSIVVMQDLILNCTNYSIYVTVNNLSRLAIPFECEKYP--VGMEVFLGIIFFIIVVGAIGGNFLVILAIILVKKLQT---ASNWLIL
SLAFSDFFVSVLVMPIAAFNQLSR-FRWPFSEK------LCDFYNCCDVMLCTSSILNLCAISIDRYLVITRPMQYV--VRRTPALIG
GMIGVAWLMSGLISIPPVL-GWKEKFT---PGICQLTDNL-------------------------
---LYQIYA
TFCAFYIPLIVMLVLYYQIFKLARNMAQEDAKRKLGTG-QMTDEEQTSIPIQLGRTNSGDEDRKLLRLEDAQRLSKGNQGN
GFDPEAQ--

--TGPKNNAKKKKQVNNESKA-ITTLGVIMGCFTLCWLPFFIIQILKPIL---IVSKVDHE-KYL
MPWCYELFLWLGYFNSFLNPVIYAKFNREFRNPFKQILFFHCFNINARIRVDTFAEQYGLPMQKTMATSLYETSHLNTHNT
ANSRRRSSVPFTPVSRKNKSSSQSRPTSDQSTNKA--
</seq-data>
</sequence>
<sequence seq-type="Protein">
<seq-name>a2aa_human</seq-name>
<seq-info><length>1066</length></seq-info>
<seq-data>---
-MGSLQPDAGNASWNGTEAPGG-GARATPYS----LQVTLTLVCLAGLLMLLTVFGNVLVIIAVFTSRALKA---PQNLFLVSLA
SADILVATLVIPFSLANEVMG--YWYFGKA------WCEIYLALDVLFCTSSIVHLCAISLDRYWSITQAIEYN--LKRTPRRIKAIIIT
VWVISAVISFPPLI-SIEKKGGGG--GPQPAEPRCEINDQK-------------------------------
--WYVISSCIGS
FFAPCLIMILVYVRIYQIAKRRTRVPPSRR-GPDA-VAAPPGGTERRPNGLGPERS-AGPGGAEAEPLPTQLNGAPGEPAPAG
PRDTDALDLEESSSSDHAERPPGPRRPERGPRGKGKARASQVKPGDSLPRRGPGATGIGTPAAGPGEER---------------------
--VGAAKASRWRGRQNREKRF-TFVLAVVIGVFVVC
WFPFFFTYTLTAVG----------C--SVPRTLFKFFFWFGYCNSSLNPVIYTIFNHDFRRAFKKILCRGDRKRIV-----------------------
---</seq-data
>
</sequence>
<sequence seq-type="Protein">
<seq-name>a2aa_mouse</seq-name>
<seq-info><length>1066</length></seq-info>
<seq-data>--
-MGSLQPDAGNSSWNGTEAPGG-GTRATPYS----LQVTLTLVCLAGLLMLFTVFGNVLVIIAVFTSRALKA---PQNLFLVSLA
SADILVATLVIPFSLANEVMG--YWYFGKV------WCEIYLALDVLFCTSSIVHLCAISLDRYWSITQAIEYN--LKRTPRRIKAIIVT
VWVISAVISFPPLI-SIEKKGAGG--GQQPAEPSCKINDQK------------------------------
---WYVISSSIG
SFFAPCLIMILVYVRIYQIAKRRTRVPPSRR-GPDA-CSAPPGGADRRPNGLGPERG-AGPTGAEAEPLPTQLNGAPGEPAPA
GPRDGDALDLEESSSSEHAERPPGPRRPDRGPRAKGKTRASQVKPGDSLPRRGPGAAGPGASGSGHGEER-----------------
---GGGAKASRWRGRQNREKRF-TFVLAVVIGVFVV
CWFPFFFTYTLIAVG----------C--PVPSQLFNFFFWFGYCNSSLNPVIYTIFNHDFRRAFKKILCRGDRKRIV----------------------
---</seq-dat
a>
</sequence>
<sequence seq-type="Protein">
<seq-name>a2aa_cavpo</seq-name>
<seq-info><length>1066</length></seq-info>
<seq-data>---
-MGSLQPDSGNASWNGTEGPGG-GTRATPYS----LQVTVTLVCLVGLLILLTVFGNVLVIIAVFTSRALKA---PQNLFLVSLAS
ADILVATLVIPFSLANEVMG--YWYFGKA------WCEIYLALDVLFCTSSIVHLCAISLDRYWSITQAIEYN--LKRTPRRIKAIIVTV
WVISAVISFPPLI-SFEKAG-GG--GQQPAEPRCEINDQK-------------------------------

--WYVISSSIGS
FFAPCLIMILVYVRIYQIAKRRTRVPPSRR-GPDAHAAAPPGGAERRPNGLGLERG-VGPGGAEAEPLPTQVNGAPGEPAPA
GPRDAEALDLEESSSSEHAERPPGARRPERGLRAKSKARASQVKPGDSLPRRAPGAAGSGTSGSGPGEER-------------------
--GGGAKASRWRGRQNREKRF-TFVLAVVIGVFVVC
WFPFFFTYTLTAVG----------C--SVPRTLFKFFFWFGYCNSSLNPVIYTIFNHDFRRAFKKILCRGDRKRIV----------------------
--</seq-data
>
</sequence>
<sequence seq-type="Protein">
<seq-name>q28838</seq-name>
<seq-info><length>1066</length></seq-info>
<seq-data>---
-MGSLQPDAGNASWNGTEAPGG-GARATPYS----LQVTLTLVCLAGLLMLFTVFGNVLVIIAVFTSRALKA---PQNLFLVSLA
SADILVATLVIPFSLANEVMG--YWYFGKA------WCEIYLALDVLFCTSSIVHLCAISLDRYWSITQAIEYN--LKRTPRRIKAIIVT
VWVISAVISFPPLI-SFEKKRGRS---GQPSAEPRCEINDQK-----------------------------
---WYVISSSIGS
FFAPCLIMILVYVRIYQIAKRRTRVPPSRR-GPDATAAELPGSAERRPNGLGPERGGVGPVGAEVESLQVQLNGAPGEPAPA
GAG-ADALDLEESSSSEHAERPPGSRRSERGPRAKGKARASQVKPGDSLPRRGPGATGLGAPTAGPAEERS-----------------
--GGGAKASRWRGRQNREKRF-TFVLAVVIGVFVV
CWFPFFFTYTLTAIG----------C--PVPPTLFKFFFWFGYCNSSLNPVIYTIFNHDFRRAFKKILCRGDRKRIV---------------------
---</seq-dat
a>
</sequence>
<sequence seq-type="Protein">
<seq-name>a2ac_mouse</seq-name>
<seq-info><length>1066</length></seq-info>
<seq-data>--MASPALAAA
LAAAAAEGPNGSDAGEWGSGGGANASGTDW-VPPPGQYS----AGAVAGLAAVVGFLIVFTVVGNVLVVIAVLTSRALRA-
--PQNLFLVSLASADILVATLVMPFSLANELMA--YWYFGQV------WCGVYLALDVLFCTSSIVHLCAISLDRYWSVTQAVEY
N--LKRTPRRVKATIVAVWLISAVISFPPLV-SFYRRPDGA---AYPQCGLNDET----

----------WYILSSCIGSFFAPCLIMGLVYARIYRVAKLRTRTLSEKR-GPAG----PDGASPTTENGLG-----KAAGENGHCAPPRT
EVEPDESSAAERRRRR-GALRRGGRRREGAEGDTGSADGPGP--
----------------------------------GLAAEQGARTASRSPGPGGRLSRASSRSVEFFLSRRRRARSSVCRRKVAQAREKRF-TFVLA
VVMGVFVLCWFPFFFSYSLYGIC-------REAC--QLPEPLFKFFFWIGYCNSSLNPVIYTVFNQDFRRSFKHILFRRRRRGFRQ--
----
-----</seq-data>
</sequence>
<sequence seq-type="Protein">
<seq-name>a2ac_cavpo</seq-name>
<seq-info><length>1066</length></seq-info>
<seq-data>--MASPALAAA

LLA--AEGPNASGAGEG--GGGVNASGAVW-GPPPSQYS----AGAVAGLAAVVGFLIVFTVVGNVLVVIAVLTSRALRA---P
QNLFLVSLASADILVATLVMPFSLANELMA--YWYFGQV------WCGVYLALDVLFCTSSIVHLCAISLDRYWSVTQAVEYN--
LKRTPRRVKATIVAVWLISAIISFPPLV-SFYRQPDGA---AYPRCGLNDET---------
----
-----WYILSSCIGSFFAPCLIMGLVYARIYRVAKLRTRTLSEKR-GPAG----PEGESPTTENGLGAAAA-AAAGENGHCAPPRAD
VEPDESSAAERRRRR-GALRRGARQREAGVEAPGPLG---
-----------------------------SAAADPGALSVSRSPGPGGRLSRASSRSVEFFLSRRRRARSSVCRRKVAQAREKRF-TFVLAVVM
GVFVLCWFPFFFSYSLYGIC-------REAC--QLPTPLFKFFFWIGYCNSSLNPVIYTIFNQDFRRSFKHILFRRRRRGFRQ---------
---<
/seq-data>
</sequence>
<sequence seq-type="Protein">
<seq-name>a2ac_human</seq-name>
<seq-info><length>1066</length></seq-info>
<seq-data>--MASPALAAA
LAVAAAAGPNASGAGERGSGGVANASGASW-GPPRGQYS----AGAVAGLAAVVGFLIVFTVVGNVLVVIAVLTSRALRA--
-PQNLFLVSLASADILVATLVMPFSLANELMA--YWYFGQV------WCGVYLALDVLFCTSSIVHLCAISLDRYWSVTQAVEYN
--LKRTPRRVKATIVAVWLISAVISFPPLV-SLYRQPDGA---AYPQCGLNDET------

--------WYILSSCIGSFFAPCLIMGLVYARIYRVAKRRTRTLSEKR-APVG----PDGASPTTENGLGAAAGEARTG---TARPRPP
TWSRTRAAQRPRGGAP-GPLRRGGRRRAGAEGGAGGADGQGAG---
---------------------------------------PGAAQSGALTASRSPGPGGRLSRASSRSVEFFLSRRRRARSSVCRRKVAQAREKRF-TF
VLAVVMGVFVLCWFPFFFIYSLYGIC-------REAC--QVPGPLFKFFFWIGYCNSSLNPVIYTVFNQDFRPSFKHILFRRRRRGF
RQ--
-----------</seq-data>
</sequence>
<sequence seq-type="Protein">
<seq-name>a2ad_human</seq-name>
<seq-info><length>1066</length></seq-info>
<seq-data>--MASPALAAA
LAVAAAAGPNASGAGERGSGGVANASGASW-GPPRGQYS----AGAVAGLAAVVGFLIVFTVVGNVLVVIAVLTSRALRA--
-PQNLFLVSLASADILVATLVMPFSLANELMA--YWYFGQV------WCGVYLALDVLFCTSSIVHLCAISLDRYWSVTQAVEYN
--LKRTPRRVKATIVAVWLISAVISFPPLV-SLYRQPDGA---AYPQCGLNDET------

--------WYILSSCIGSFFAPCLIMGLVYARIYRVAKLRTRTLSEKR-APVG----PDGASPTTENGLGAAAGAGENG---HCAPPPA
DVEPDESSAAAERRRRRGALRRGGRRRAGAEGGAGGADGQ---
------------------------------------GAAESGALTASRSPGPGGRLSRASSRSVEFFLSRRRRARSSVCRRKVAQAREKRF-TFVLA
VVMGVFVLCWFPFFFSYSLYGIC-------REAC--QVPGPLFKFFFWIGYCNSSLNPVIYTVFNQDFRRSFKHILFRRRRRGFRQ-
----
------</seq-data>
</sequence>
<sequence seq-type="Protein">

<seq-name>a2ac_didma</seq-name>
<seq-info><length>1066</length></seq-info>
<seq-data>---MDL
QLTTNSTDSGDR--GGSSNESLQRQ---PPSQYS----PAEVAGLAAVVSFLIVFTIVGNVLVVIPVLTSRALKA---PQNLFLVSLA
SADILVATLVMPFSLANELMN--YWYFGKV------WCDIYLALDVLFCTSSIVHLCAISLDRYWSVTQAVGYN--LKRTPRRIKGI
IVTVWLISAVISFPPLI-SLYRDPEDD---LYPQCELNDET------------------------------
--WYILSSCIGS
FFAPCIIMVLVYVRIYRVAKLRTRTLSEKRTVPEGSSQTENGLSRPPVGAGPSTAAAAAASLRLQAGENGHYHLHHHHHHL
HHHHHHHHHQLRKSAELEDIELEESSTSENRRRRRSREEAARPKGRPR--
-----------------------------FSFSFSSTKGGQSAGAGSRLSRASNRSLEFFSSHRRRKRSSLCRRKVTQAREKRF-TFVLAVVMGV
FVVCWFPFFFTYSLYGIC-------REAC--QVPETLFKFFFWFGYCNSSLNPVIYTIFNQDFRRSFKHILFKKKKKTSLQ-------------
--</se
q-data>
</sequence>
<sequence seq-type="Protein">
<seq-name>a2ar_labos</seq-name>
<seq-info><length>1066</length></seq-info>
<seq-data>--M
DPLNATGMDAF-TAIHLNASWSA-----DSGYS----LAAIASIAALVSFLILFTVVGNILVVIAVLTSRALKA---PQNLFLVSLATA
DILVATLVMPFSLANELMG--YWYFGKV------WCGIYLALDVLFCTSSIVHLCAISLDRYWSVTQAVEYN--LKRTPKRVKCIIV
IVWLISAFISSPPLL-SIDSNNYIS---SQPQCMLNDDT--------------------------------
--WYILSSSMAS
FFAPCLIMILVYIRIYQVAKTRTRSMSGKEPRPDGVTQTENGLNKANSPCHGDRENGHCQCPPTPSQRTVTIGQQTDDAD
MDESFSSEGKGHKPQRQDSQ---
------------RAKRPGLKKSSISKQSARISRVSNKSVDLFASRRKRRRSSIAEKKVSQAREKRF-TFVLAVVMGVFVVCWFPFFFSY
SLHAVC-------RDYC--KIPDTLFK-FFWIGYCNSSLNPAIYTIFNRDFRRAFQKILCKSWKKSF-------------------------------------
--</seq-data>
</sequence>
<sequence seq-type="Protein">
<seq-name>a2ab_human</seq-name>
<seq-info><length>1066</length></seq-info>
<seq-data>---
-----------------------MDHQDPYS----VQATAAIAAAITFLILFTIFGNALVILAVLTSRSLRA---PQNLFLVSLAAADILVATLIIPFS
LANELLG--YWYFRRT------WCEVYLALDVLFCTSSIVHLCAISLDRYWAVSRALEYN--SKRTPRRIKCIILTVWLIAAVISLPPLI
-YKGDQGPQP--RGRPQCKLNQEA--
--WYILASSIGSFFAPCLIMILVYLR
IYLIAKRSNRRGPRAKGGPGQGESKQPRPDHGGALASAKLPALASVASAREVNGHSKSTGEKEEGETPEDTGTRALPPSW
AALPNSGQGQKEGVCGASPEDEAEEEEEEEEEEEEECEPQAV--
------------PVSPASACSPPLQQPQGSRVLATLRGQVLLGRGVGAIGGQWWRRRAHVTREKRF-TFVLAVVIGVFVLCWFPF
FFSYSLGAIC-------PKHC--KVPHGLFQFFFWIGYCNSSLNPVIYTIFNQDFRRAFRRILCRPWTQTAW-------------------------
---</seq-data>
</sequence>

<sequence seq-type="Protein">
<seq-name>oar_bommo</seq-name>
<seq-info><length>1066</length></seq-info>
<seq-data>--MGQAATHDA
NNYTSINYTEIYDVIEDEKDVCAVADEPNIPCSFGISLAVP----EWEAICTAIILTMIIISTVVGNILVILSVFTYKPLRI---VQNFFI
VSLAVADLTVAILVLPLNVAYSILG--QWVFGIY------VCKMWLTCDIMCCTSSILNLCAIALDRYWAITDPINYA--QKRTLER
VLFMIGIVWILSLVISSPPLL-GWNDWPEVF---EPDTPCRLTSQP-------------------
--GFV
IFSSSGSFYIPLVIMTVVYFEIYLATKKRLRDRAKATKISTISSGRNKYETKESDPNDQDSVSSDANPNEHQGGTRLVAENEKK
HRTRKLTPKKKPKRRYWSKDDKSHNKLIIPILSNENSVTDIGENLENRNTSSESNSKETHEDNMIEITEAAPVKIQ-------------
---KRPKQNQTNAVYQFIEEKQRISLTRERRA-ARTLGIIMG
VFVVCWLPFFVIYLVIPFC--------VSC--CLSNKFINFITWLGYVNSALNPLIYTIFNMDFRRAFKKLLFIKC------------------------
--</seq-d
ata>
</sequence>
<sequence seq-type="Protein">
<seq-name>oar_helvi</seq-name>
<seq-info><length>1066</length></seq-info>
<seq-data>--MGQAATHV
DANYTLINYTEEVIEDDRDACAVADDPKYPSSFGITLAVP----EWEAICTAIVLTLIIISTIVGNILVILSVFTYKPLRI---VQNFFIV
SLAVADLTVAILVLPLNVAYSILG--QWVFGIY------VCKMWLTCDIMCCTSSILNLCAIALDRYWAITDPINYA--QKRTLERV
LLMIGVVWVLSLIISSPPLL-GWNDWPDVF---EPDTPCRLTSQP--------------------
---GFVI
FSSSGSFYIPLVIMTVVYFEIYLATKKRLRDRAKATKISTISSGQNKYNNKD-DHHDQDSVSSEANHNEHQGVTRLVSDNEKK
KRTRKLTPKKKPKRKYWSKDDKSQNKLIIPILSNENSVTDMGENLENRNTSSESNSKETHEDDLIEVNETVPVKTHH----------
---KKPKPNQQSAVYQFIEEKQRISLTRERRA-ARTLGIIM
GVFVVCWLPFFVIYLVIPFC--------ASC--CLSNKFINFITWLGYCNSALNPLIYTIFNMDFRRAFKKLLCMKP-------------------
---</se
q-data>
</sequence>
<sequence seq-type="Protein">
<seq-name>q93127</seq-name>
<seq-info><length>1066</length></seq-info>
<seq-data>--
---MSGGEASITGRTAPELNASAAPLDDERE----LGETVAATALLLAIILVTIVGNSLVIISVFTYRPLRS---VQNFFVVSLAVADL
TVALFVLPLNVAYRLLN--QWLLGSY------LCQMWLTCDILCCTSSILNLCVIALDRYWAITDPINYA--QKRTIRRVNTMIAAV
WALSLVISVPPLL-GWNDWPAQF---TEDTPCTLTQER-----------------------------
---LFVVYSSSGS
FFIPLIIMSVVYAKIFFATKRRLRERTR--KLGTLAVPAPPQRTSSRPLAELESVASQEDETEPSPEPEPLSSR---------------------
----
-----ADKPANGISVHQFIEEKQRISLSKERKA-ARVLGVIMGVFVVCWLPFFLMYAIVPFC--------TNC-APPSQRVVDFVTWL
GYVNSSLNPIIYTIYNKDFRTAFSRLLRCDRRMS---

--</seq-data>
</sequence>
<sequence seq-type="Protein">
<seq-name>oar1_locmi</seq-name>
<seq-info><length>1066</length></seq-info>
<seq-data>---MVRVELQA
ASLMNGSSAAEEPQDALVGGDACGGRRPPSVLGVRLAVP----EWEVAVTAVSLSLIILITIVGNVLVVLSVFTYKPLRI---VQ
NFFIVSLAVADLTVAVLVMPFNVAYSLIQ--RWVFGIV------VCKMWLTCDVLCCTASILNLCAIALDRYWAITDPINYA--QK
RTLRRVLAMIAGVWLLSGVISSPPLI-GWNDWPMEF--NDTTPCQLTEEQ-------

-------GYVIYSSLGSFFIPLFIMTIVYVEIFIATKRRLRERAKASKLNSAMKQQMAAQAVPSSVPSHDQESVSSETNHNELPPP
PAPPSKEKRRKTKKKSKKKEQAAEEGRFLAPAMVAEDSVTDNSVSVGPVARNHLAEDGYTCTTTTTTTTTTAVTDSPRSR
TASQKGSTAPP---TPVQPKSIPVYQFIEEKQRISLSKE
RRA-ARTLGIIMGVFVVCWLPFFLMYVIVPFC-------NPSC--KPSPKLVNFITWLGYINSALNPIIYTIFNLDFRRAFKKLLHFKT

-------------</seq-data>
</sequence>
<sequence seq-type="Protein">
<seq-name>cab76374</seq-name>
<seq-info><length>1066</length></seq-info>
<seq-data>---MN
SSGESGGTMTEDYDMTGCG----PPEEETGSNLP----VWEAAAASLTLGFLVLATVLGNALVILSVFTYRPLRI---VQNFFIVSL
AVADLAVAILVMPFNVAYLLLG--KWIFGIH------LCKLWLTCDVLCCTASILNLCAIALDRYWAITDPINYA--QKRTLKRVLA
TIAGVWILSGAISSPPLA-GWNDWPEEL--EPGTPCQLTRRQ----------------------
---GYVIY
SSLGSFFIPLLLMSLVYLEIYLATRRRLRERARQSRINAVQSTRHREADDAEESVSSETNHNERSTPRSHAKPSLIDDEPTEVTI
GGGGTTSSRRTTGS--
--------------------------------RAAATTTTVYQFIEERQRISLSKERRA-ARTLGVIMGVFVVCWLPFFLMYVIVPFC--------PDC-
-CPSDRMVYFITWLGYVNSALNPLIYTIFNLDYRRAFRRLLRIR---
---</seq-data>
</sequence>
<sequence seq-type="Protein">
<seq-name>o77254</seq-name>
<seq-info><length>1066</length></seq-info>
<seq-data>---MVRPHTRTMEE
DGGILWTTTPLPSVTVISEAVNTSSFEMDDVVEAYPSGIAMS----VQEAVGTALSLSFITVFTVVGNVLVICSVFNHRPLRT---
VQNVFLVSLALADIAVALLVMPFNVAYSIMG--RWVFGLH------FCELWLTCDVLCCTASILNLCAIALDRYWAIHDPINYA--
QKRTLRRVLLSIFLVWVISALISVPPLI-GWNDWPEQF--DETTPCRLTQET--------

------GYVLYSASGSFFIPLLIMSIVYLKIFLATRRRLRERANAAAKVPSSATRCAATVEHSVALLQERHPSSSEETPPPQHRGQT
TENRPSLADTSVTLEQ--
--NGRPPSVKVFTCWEERQRISLSRERRA-ARVLGIVMGVFVLCWLPFFIMYVTAAFC---

-----DHC--VQSDRLVNFITWLGYVNSALNPVIYTVFNTDFRRAFRSLLCSGNRRTHL--
---</seq-data>
</sequence>
<sequence seq-type="Protein">
<seq-name>dcdr_xenla</seq-name>
<seq-info><length>1066</length></seq-info>
<seq-data>---
---MENFSIFNVTV---NVWHADLDVGNSD-----LSLRALTGLLLSLLILSTLLGNTLVCLAVIKFRHLRSK--VTNFFVISLAVSDLF
VALLVMPWKAVTEVAG--FWVFGD-------FCDTWVAFDIMCSTASILNLCIISLDRYWAIASPFRYE--RKMTQRVAFIMIGV
AWTLSILISFIPVQLSWHKSHEADEELN---GVNHTENCDSSLNR-------------------------
--TYAISSSL
ISFYIPVVIMIGTYTRIYRIAQTQIRRISSLERAVEHAQRCSSRLSNENSL--

----------KTSFRKETKV-LKTLSIIMGVFVFCWLPFFVLNCMIPFCHMNLPGQNEPEPPCVSETTFNIFVWFGWANSSLNPVI
Y-AFNADFRKAFTTILGCNRFCSSNNVEAVNFSNELVSYHHDTTFQKDIPVTFNNSHLPNVVDQDQEVLEGTCFDKVSVLST
SHGTRSQKNLHLPAGVQFECEAEITLETITPFTSTGPLECLPQLVADEDRHYTTKLY-----------------------------------</seq-d
ata>
</sequence>
<sequence seq-type="Protein">
<seq-name>dadr_human</seq-name>
<seq-info><length>1066</length></seq-info>
<seq-data>---
-----------MRTLNTSAMDGTGLVVERD-----FSVRILTACFLSLLILSTLLGNTLVCAAVIRFRHLRSK--VTNFFVISLAVSDLLVA
VLVMPWKAVAEIAG--FWPFGS-------FCNIWVAFDIMCSTASILNLCVISVDRYWAISSPFRYE--RKMTPKAAFILISVAWT
LSVLISFIPVQLSWHKAKPTSPSDGNATS--------------------------------------LAETIDNCDSSLSR----------------------------
---TYAISSSVISF
YIPVAIMIVTYTRIYRIAQKQIRRIAALERAAVHAKNCQTTTGNGKPVECSQPESSF---

--------------KMSFKRETKV-LKTLSVIMGVFVCCWLPFFILNCILPFC-----GSGETQPFCIDSNTFDVFVWFGWANSSLNPIIY
-AFNADFRKAFSTLLGCYRLCPATNNAIETVSINNNGAAMFSSHHEPRGSISKECNLVYLIPHAVGSSEDLKKEEAAGIARPLE
KLSPALSVILDYDTDVSLEKIQPITQNGQHPT---</seq-data>
</sequence>
<sequence seq-type="Protein">
<seq-name>grec_balam</seq-name>
<seq-info><length>1066</length></seq-info>
<seq-data>---
--MEGPPLSPAPADNVTLNVSCGRPATLFDW----ADHRLISLLALAFLNLMVVAGNLLVVMAVFVHSKLRT---VTNLFIVSLA
CADLLVGMLVLPFSATLEVLD--VWLYGDV------WCSVWLAVDVWMCTSSILNLCAISLDRYLAVSQPISYP--SLMSTRRAK
QLIAAVWVLSFVICFPPLV-GWNDRPGTLIGSR--GSSACRLTCELTNER----------------
---G
YVIYSALGSFFLPSTVMLFFYGRIYRTAVSTTRAIAQGFRTTKEDEEGRLTLRIHRGRSVTQRAEQAAAGGARAHGQVRLTLS
EPGARRQNKPSFVVHCREDSRAKNQYEIYTVVEGDSRPGRRVPQPQRPAKKLSSASQSSEDD------------------------------

---SRPPRFISRVSRRNVRHQARRFRMETKA-AKTVGIIVGL
FILCWLPFFVCYLVRGFC--------ADC---VPPLLFSVFFWLGYCNSAVNPCVYALCSRDFRFAFSSILCKCVCRRGAMERRFRR
TLLVGNRSQTEEDCEVAD--
---------------</seq-data>
</sequence>
<sequence seq-type="Protein">
<seq-name>o77408</seq-name>
<seq-info><length>1066</length></seq-info>
<seq-data>---
------MSRDIFMKRLRLHLLFDEVAMVTHI----VGDVLSSVLLCAVV-LLVLVGNTLVVAAVATSRKLRT---VTNVFIVNLACAD
LLLGVLVLPFSAVNEIKD--VWIFGHV------WCQVWLAVDVWLCTASILNLCCISLDRYLAITRPIRYP--GLMSAKRAKTLVA
GVWLFSFVICCPPLI-GWNDGGDGIMDYNGTTATPIPVTTTQTPVTGRDDVLCDNGFNYSTNSNMNTTCTYSGDSSLSTT
CELTNSR--
--------------------------GYRIYAALGSFFIPMLVMVFFYLQIYRAAVKTISAYAKGELKTKYSVRENGSKTNSVTLRIHRGGRGP
STGSSVYRHGSTYGGSAAGAATREGCGDKDAAGGRRFGRQEMDSHLPVRKCRSSDASLVTLTGLKCEIIDNGNAKHGPIS
ELIKGRGKSFFWRKEKKRSVGGERESFENSTRNGRSTRAKLCGGRCLAIETDICSSGECSPRTKRIKEHARATQHNSLPVTPSL
SSQNEETDAVFVRGTSNSEYKPRRSRLSAHKPGHAMRLHMQKFNREKKA-AKTLAIIVGAFIMCWMPFFTIYLVGAFC-----
---ENC---ISPIVFSVAFWLGYCNSAMNPCVYALFSRDFRFAFRKLLT-CSCKAWSKNRSFRPQTSDVPAIQLHCATQDDAKS
SSDIGPTASGGNGGYT--</se
q-data>
</sequence>
<sequence seq-type="Protein">
<seq-name>q9ttm9</seq-name>
<seq-info><length>1066</length></seq-info>
<seq-data>---MTFRDLLSVNFEGSRSDGSAGGASAGGSGGG---SGG
AAASEGRAVDGVPGTAG-SGG--VVGAGSDENNRSSAGEPGAAGAGGEVNGTAAVGGLVV----SAQGVGVGVFLAAFIL
MAVAGNLLVILSVACNRHLQT---VTNYFIVNLAVADLLLSATVLPFSATMEVLG--FWAFGRA------FCDVWAAVDVLCCT
ASILSLCTISVDRYVGVRHSLKYP--SIMTERKAAAILALLWAVAIVVSVGPLL-GWKEP--
---------VPPDERFCGITEEA---
---GYAVFSSLCSFYLPMAVIVVMYCRVYVVARSTTRSLEAGVKRERGKASEVVLRIHC
RGSSTGTDRGHGAMRSTKGHTF--
---RSSLSLRLLKFSREKKA-AKTLAIVVGVFVLCWFP
FFFVLPLGSLF----------PQLKPSEGVFKVIFWLGYFNSCVNPLIYPCSSREFKRAFLRLLRCQCHHSRRRRRPLW--RAYGHH
WLASNGG-PRPDCAPGLGAAPREAPLALPAPEATD--TPSAPEAQAPVVGRRKPPYSFRDWRLLGPFRRPTTQLRAKVSSLS
QKIRAGSAPCAEAPCALRSEVEAVSLNVPHDAAEGATWQAYELADYSHLRETDI</seq-data>
</sequence>
<sequence seq-type="Protein">
<seq-name>a1ad_human</seq-name>
<seq-info><length>1066</length></seq-info>
<seq-data>---MTFRDLLSVSFEGPRPDSSAGGSSAGGGGGS---AGGA
APSEGPAVGGVPGGAGGGGG--VVGAGSGEDNRSSAGEPGSAGAGGDVNGTAAVGGLVV----SAQGVGVGVFLAAFIL
MAVAGNLLVILSVACNRHLQT---VTNYFIVNLAVADLLLSATVLPFSATMEVLG--FWAFGRA------FCDVWAAVDVLCCT

ASILSLCTISVDRYVGVRHSLKYP--AIMTERKAAAILALLWVVALVVSVGPLL-GWKEP---
----------VPPDERFCGITEEA--
--GYAVFSSVCSFYLPMAVIVVMYCRVYVVARSTTRSLEAGVKRERGKASEVVLRIH
CRGAATGADGAHG-MRSAKGHTF--
---RSSLSVRLLKFSREKKA-AKTLAIVVGVFVLCW
FPFFFVLPLGSLF----------PQLKPSEGVFKVIFWLGYFNSCVNPLIYPCSSREFKRAFLRLLRCQCRR-RRRRRPLW--RVYGHH
WRASTSG-LRQDCAPSSGDAPPGAPLALTALPDPDPEPPGTPEMQAPVASRRKPPSAFREWRLLGPFRRPTTQLRAKVSSL
SHKIRAGGAQRAEAACAQRSEVEAVSLGVPHEVAEGATCQAYELADYSNLRETDI</seq-data>
</sequence>
<sequence seq-type="Protein">
<seq-name>a1ad_rabit</seq-name>
<seq-info><length>1066</length></seq-info>
<seq-data>---MTFRDLLSVTFEGPRPDISAGGSGAGGGAGAGAGAG
DTASSESPAVGGVPGAAGGGGGGSVVGAGSGEDNRSSAGEPGGAGGGGEVNGTAAVGGLVV----SAQSVGVGVFLAA
FILTAVAGNLLVILSVACNRHLQT---VTNYFIVNLAVADLLLSATVLPFSATMEVLG--FWAFGRA------FCDVWAAVDVLCC
TASILSLCTISVDRYVGVRHSLKYP--AIMTERKAAAILALLWAVALVVSMGPLL-GWKEP---
------------VPPDERFCGITEEV--
--GYAVFSSLCSFYLPMAVIVVMYCRVYVVARSTTRSLEAGVKRERGKASEVVLRI
HCRGAASGADGAPG-TRGAKGHTF---
--RSSLSVRLLKFSREKKA-AKTLAIVVGVFVLC
WFPFFFVLPLGSLF----------PQLKPSEGVFKVIFWLGYFNSCVNPLIYPCSSREFKRAFLRLLRCQCRR-RRRRRPLW--RVYG
HHWRASAGGGPHPDCALSAGAALPGAALALTAAPAPS--SAAAPEGQAAGAGRRKPPCAFREWRLLGPLRRPTTQLRAK
VSSLSHKIRAGGAQRAEAACALRSEVEAVALSVARDVAEDNTCQAYELADYRNLRETDI</seq-data>
</sequence>
<sequence seq-type="Protein">
<seq-name>a1aa_rat</seq-name>
<seq-info><length>1066</length></seq-info>
<seq-data>---
---------MVLLSENASEGSNCTHPPAPVN----ISKAILLGVILGGLIIFGVLGNILVILSVACHRHLHS---VTHYYIVNLAVADLLLT
STVLPFSAIFEILG--YWAFGRV------FCNIWAAVDVLCCTASIMGLCIISIDRYIGVSYPLRYP--TIVTQRRGVRALLCVWVLSL
VISIGPLF-GWRQP---APEDETICQINEEP---
---GYVLFSALGSFYVPLAIIL
VMYCRVYVVAKRESRGLKSGLKTDKSDSEQVTLRIHRKNVPAEGGGVSSAKN--

-KTHFSVRLLKFSREKKA-AKTLGIVVGCFVLCWLPFFLVMPIGSFF----------PDFKPSETVFKIVFWLGYLNSCINPIIYPCSSQ
EFKKAFQNVLRIQC---LRRRQSSKHALGYTLHPPSQALEGQHRDMVRIPVGSGETFYKISKTDGVCEWKFFSSMPQGSARI
TVPKDQSACTTARVRSKSFLQVCCCVGSSAPRPEENHQVPTIKIHTISLGENGEEV--------------------------------</seq-data
>
</sequence>
<sequence seq-type="Protein">
<seq-name>a1aa_rabit</seq-name>
<seq-info><length>1066</length></seq-info>

<seq-data>---
---------MVFLSGNASDSSNCTHPPAPVN----ISKAILLGVILGGLILFGVLGNILVILSVACHRHLHS---VTHYYIVNLAVADLLLT
STVLPFSAIFEILG--YWAFGRV------FCNIWAAVDVLCCTASIISLCVISIDRYIGVSYPLRYP--TIVTQRRGLRALLCVWAFSLV
ISVGPLF-GWRQP--APDDETICQINEEP--
--GYVLFSALGSFYVPLTIIL
AMYCRVYVVAKRESRGLKSGLKTDKSDSEQVTLRIHRKNAPAGGSGVASAKN--

-KTHFSVRLLKFSREKKA-AKTLGIVVGCFVLCWLPFFLVMPIGSFF----------PDFKPPETVFKIVFWLGYLNSCINPIIYPCSSQ
EFKKAFQNVLKIQC---LRRKQSSKHALGYTLHAPSQALEGQHKDMVRIPVGSGETFYKISKTDGVCEWKFFSSMPRGSARIT
VPKDQSACTTARVRSKSFLQVCCCVGPSTPNPGENHQVPTIKIHTISLSENGEEV-------------------------------</seq-data>
</sequence>
<sequence seq-type="Protein">
<seq-name>a1ab_rat</seq-name>
<seq-info><length>1066</length></seq-info>
<seq-data>---MNPDL
DTGHNTSAPAHWGELKDDNFTGPNQTSSNSTLPQLD----VTRAISVGLVLGAFILFAIVGNILVILSVACNRHLRT---PTNYFI
VNLAIADLLLSFTVLPFSATLEVLG--YWVLGRI------FCDIWAAVDVLCCTASILSLCAISIDRYIGVRYSLQYP--TLVTRRKAILA
LLSVWVLSTVISIGPLL-GWKEP--APNDDKECGVTEEP---------------------------
---FYALFSSL
GSFYIPLAVILVMYCRVYIVAKRTTKNLEAGVMKEMSNSKELTLRIHSKNFHEDTLSSTKAKGHNP-----------------------------
----
-------------------RSSIAVKLFKFSREKKA-AKTLGIVVGMFILCWLPFFIALPLGSLF----------STLKPPDAVFKVVFWLGYFNSC
LNPIIYPCSSKEFKRAFMRILGCQCRGGRRRRRRRR-LGACAYTYRPWTRGGSLERSQSRKDSLDDSGSCMSGTQRTLPSAS
PSPGYLGRGTQPPVELCAFPEWKP-GALLSLP--EPPGRRGRLDSGPLFTFKLLGDPESPGTEGDTSNGGCDTTTDLANGQP
GFKSNMPLAPGHF---</seq-data>
</sequence>
<sequence seq-type="Protein">
<seq-name>a1ab_human</seq-name>
<seq-info><length>1066</length></seq-info>
<seq-data>---MNPDL
DTGHNTSAPAHWGELKNANFTGPNQTSSNSTLPQLD----ITRAISVGLVLGAFILFAIVGNILVILSVACNRHLRT---PTNYFI
VNLAMADLLLSFTVLPFSAALEVLG--YWVLGRI------FCDIWAAVDVLCCTASILSLCAISIDRYIGVRYSLQYP--TLVTRRKAI
LALLSVWVLSTVISIGPLL-GWKEP--APNDDKECGVTEEP------------------------
--FYALFS
SLGSFYIPLAVILVMYCRVYIVAKRTTKNLEAGVMKEMSNSKELTLRIHSKNFHEDTLSSTKAKGHNP--------------------------
----
----------------------RSSIAVKLFKFSREKKA-AKTLGIVVGMFILCWLPFFIALPLGSLF----------STLKPPDAVFKVVFWLGYFN
SCLNPIIYPCSSKEFKRAFVRILGCQCRGRRRRRRRRRRLGGCAYTYRPWTRGGSLERSQSRKDSLDDSGSCLSGSQRTLPSA
SPSPGYLGRGAPPPVELCAFPEWKAPGALLSLPAPEPPGRRGRHDSGPLFTFKLLTEPESPGTDGGASNGGCEAAADVAN
GQPGFKSNMPLAPGQF---</seq-data>
</sequence>
<sequence seq-type="Protein">

<seq-name>1BOJ</seq-name>
<seq-info><length>1066</length></seq-info>
<seq-data>---MN
GTEGPNFYVPFSNKTGVVRSPFEAPQYYLAE-------PWQFSMLAAYMFLLIMLGFPINFLTLYVTVQHKKLRT---PLNYILLNL
AVADLFMVFGGFTTTLYTSLHG--YFVFGPT------GCNLEGFFATLGGEIALWSLVVLAIERYVVVCKPMSNF---RFGENHAI
MGVAFTWVMALACAAPPLV-GWSRYIP---EGMQCSCGIDYYTPH--------------
--EETN
NESFVIYMFVVHFIIPLIVIFFCYGQLVFTVKEAAAQQQESAT---

--------------------TQKAEKEV-TRMVIIMVIAFLICWLPYAGVAFYIFTH------QGSDFG----PIFMTIPAFFAKTSAVYNPVIYI
MMNKQFRNCMVTTLCCGKNPLGDDEASTTVSKTE-----TSQVAPA---
---</seq-data>
</sequence>
<sequence seq-type="Protein">
<seq-name>opsv_chick</seq-name>
<seq-info><length>1066</length></seq-info>
<seq-data>---
MSSDDDFYLFTNG-SVPGPWDGPQYHIAP-------PWAFYLQTAFMGIVFAVGTPLNAVVLWVTVRYKRLRQ---PLNYILVN
ISASGFVSCVLSVFVVFVASARG--YFVFGKR------VCELEAFVGTHGGLVTGWSLAFLAFERYIVICKPFGNF---RFSSRHALL
VVVATWLIGVGVGLPPFF-GWSRYMP---EGLQCSCGPDWYTVG----------------
---TKYRSE
YYTWFLFIFCFIVPLSLIIFSYSQLLSALRAVAAQQQESAT---

--------------TQKAEREV-SRMVVVMVGSFCLCYVPYAALAMYMVNN------RDHGLD----LRLVTIPAFFSKSACVYNPIIYCF
MNKQFRACIME-TVCGKPLTDDSDASTSAQRTEVSSVSSSQVGPT---
---</seq-data>
</sequence>
<sequence seq-type="Protein">
<seq-name>opsr_anoca</seq-name>
<seq-info><length>1066</length></seq-info>
<seq-data>--MAGTVTEAWDVA
VFAARRRNDEDDTTRDSLFTYTNSNNTRGPFEGPNYHIAP-------RWVYNITSVWMIFVVIASIFTNGLVLVATAKFKKLRH-
--PLNWILVNLAIADLGETVIASTISVINQISG--YFILGHP------MCVLEGYTVSTCGISALWSLAVISWERWVVVCKPFGNV---
KFDAKLAVAGIVFSWVWSAVWTAPPVF-GWSRYWP---HGLKTSCGPDVFSGS

------DDPGVLSYMIVLMITCCFIPLAVILLCYLQVWLAIRAVAAQQKESES--

-----------------------------------TQKAEKEV-SRMVVVMIIAYCFCWGPYTVFACFAAAN-------PGYAFH---PLAAALPAYFAKS
ATIYNPIIYVFMNRQFRNCIMQ--LFGKKVDDGSELS-STSRTEVSSVSNSSVSPA---
---</seq-data>
</sequence>
<sequence seq-type="Protein">

<seq-name>trfr_human</seq-name>
<seq-info><length>1066</length></seq-info>
<seq-data>---
-----------MENETVSELNQTQLQPRAVVA---LEYQVVTILLVLIICGLGIVGNIMVVLVVMRTKHMRT---PTNCYLVSLAVAD
LMVLVAAGLPNITDSIYG--SWVYGYV------GCLCITYLQYLGINASSCSITAFTIERYIAICHPIKAQ--FLCTFSRAKKIIIFVWAF
TSLYCMLWFFLLDLNIST---YKDAIVISCGYKIS---
---RNYYSPIYLMDFGVFYVV
PMILATVLYGFIARILFLNPIPSDPKENSKTWKNDSTHQNTNLNVNTSNRCFNS---

--------------TVSSRKQV-TKMLAVVVILFALLWMPYRTLVVVNSFL----------SSPFQENWFLLFCRICIYLNSAINPVIYNLMS
QKFRAAFRKLCNCKQKPTEKPANYSVALNYSVIKESDHFSTELDDITVTDTYLSATKVSFDDTCLASEVSFSQS-----------------
---</seq-data>
</sequence>
<sequence seq-type="Protein">
<seq-name>gpra_human</seq-name>
<seq-info><length>1066</length></seq-info>
<seq-data>---MASSTTRGPRVSDLF
SGLPPAVTTPANQSAEASAGNGSVAGADAPAVTPFQSLQLV-----HQLKGLIVLLYSVVVVVGLVGNCLLVLVIARVPRLHN-
--VTNFLIGNLALSDVLMCTACVPLTLAYAFEP-RGWVFGGG------LCHLVFFLQPVTVYVSVFTLTTIAVDRYVVLVHPLR----
-RASRCASAYAVLAIWALSAVLALPPAVHTYHV--ELKP-HDVRLCEEFWGS------

--QERQRQLYAWGLLLVTYLLPLLVILLSYVRVSVKLRNRVVPGCVTQSQADW---

-----------------------------------DRARRRRT-FCLLVVVVVVFAVCWLPLHVFNLLRDLD---PHAIDPYAF----GLVQLLCHWLA
MSSACYNPFIYAWLHDSFREELRKLLVAWPRKIAPHGQNMTVSVVI---
--</seq-data>
</sequence>
<sequence seq-type="Protein">
<seq-name>ny2r_human</seq-name>
<seq-info><length>1066</length></seq-info>
<seq-data>--MGPIGAEAD
ENQTVEEMKVEQYGPQTTPRGELVPDPEPELIDSTKL------IEVQVVLILAYCSIILLGVIGNSLVIHVVIKFKSMRT---VTNFFI
ANLAVADLLVNTLCLPFTLTYTLMG--EWKMGPV------LCHLVPYAQGLAVQVSTITLTVIALDRHRCIVYHLES----KISKRISF
LIIGLAWGISALLASPLAIFREYS--LIEIIPDFEIVACTEKWPG--------------------------
--EEKSIYGTVYSLS
SLLILYVLPLGIISFSYTRIWSKLKNHVSPGAANDH---

-------YHQRRQKT-TKMLVCVVVVFAVSWLPLHAFQLAVDID---SQVLDLKEY----KLIFTVFHIIAMCSTFANPLLYGWMNS
NYRKAFLSAFRCEQRLDAIHSEVSVTFKAKKNLEVRKNSGPNDSFTEATNV--
--</seq-data>
</sequence>
<sequence seq-type="Protein">

<seq-name>ny6r_rabit</seq-name>
<seq-info><length>1066</length></seq-info>
<seq-data>---ME
VSLNDPASNKTSAKSNSSAFFYFESCQSP---------SLALLLLLIAYTVVLIMGICGNLSLITIIFKKQREAQN--VTNILIANLSLSDI
LVCVMCIPFTAIYTLMD--RWIFGNT------MCKLTSYVQSVSISVSIFSLVLIAIERYQLIVNPRGW----KPSASHAYWGIMLIW
LFSLLLSIPLLLSYHLTDEP--FRNLSLPTDLYSHHVVCVEHWP---------------------------------
---SKTNQLLYSTSLIM
LQYFVPLGFMFICYLKIVICLHKRNSKIDRRRENES---

----RLTENKRI-NTMLISIVVTFAACWLPLNTFNVIFDWY---HEVLMSCHH----DLVFAICHLVAMVSTCINPLFYGFLNRNFQ
KDLVVLIHHCLCFALRERYENIAIST---LHTDESKGSLRVAHIPAGI---
---</seq-data>
</sequence>
<sequence seq-type="Protein">
<seq-name>ny4r_human</seq-name>
<seq-info><length>1066</length></seq-info>
<seq-data>--MNTSHL
LALLLPKSPQGENRSKPLGTPYNFSEHCQDS---------VDVMVFIVTSYSIETVVGVLGNLCLMCVTVRQKEKAN---VTNLLIA
NLAFSDFLMCLLCQPLTAVYTIMD--YWIFGET------LCKMSAFIQCMSVTVSILSLVLVALERHQLIINPTGW----KPSISQAY
LGIVLIWVIACVLSLPFLANSILENVF--HKNHSKALEFLADKVVCTESWP-------------------
--LAHHR
TIYTTFLLLFQYCLPLGFILVCYARIYRRLQRQGRVFHKGTYSL--

-----------------RAGHMKQV-NVVLVVMVVAFAVLWLPLHVFNSLEDWH---HEAIPICHG----NLIFLVCHLLAMASTCVNPF
IYGFLNTNFKKEIKALVLTCQQSAPLEESEHLPLST---VHTEVSKGSLRLSGRSNPI--
--</seq-data>
</sequence>
<sequence seq-type="Protein">
<seq-name>nk3r_mouse</seq-name>
<seq-info><length>1066</length></seq-info>
<seq-data>---MASVPTGENWTDGTAGVGSHT
GNLSAALGITEWLALQAGNFSSALGLPVTSQAPSQVRDNLTNQFVQP-------SWRIALWSLAYGLVVAVAVFGNLIVIWIIL
AHKRMRT---VTNYFLVNLAFSDASVAAFNTLVNFIYGVHS--EWYFGAN------YCRFQNFFPITAVFASIYSMTAIAVDRYM
AIIDPLKP----RLSATATKIVIGSIWILAFLLAFPQCLYSKIK--VMPGRTLCYVQ
WPE---
--------------GPKQHFTYHIIVIILVYCFPLLIMGVTYTIVGITLWGGEILGDTCDKYHE---
----
--QLKAKRKV-VKMMIIVVVTFAICWLPYHVYFILTAIY-----QQLNRWK--YIQQVYLASF
WLAMSSTMYNPIIYCCLNKRFRAGFKRAFRWCPFIQVSSYDELELKTTRFHPTRQP--
---</seq-data>
</sequence>
<sequence seq-type="Protein">

<seq-name>nk2r_bovin</seq-name>
<seq-info><length>1066</length></seq-info>
<seq-data>---M
GACVVMTDINISSGLDSNATGITAFSMP---------GWQLALWTAAYLALVLVAVMGNATVIWIILAHQRMRT---VTNYFIV
NLALADLCMAAFNAAFNFVYASHN--IWYFGRA------FCYFQNLFPITAMFVSIYSMTAIAADRYMAIVHPFQP----RLSAPG
TRAVIAGIWLVALALAFPQCFYSTIT--TDEGATKCVVAWPE----------------------
---DSGGKML
LLYHLIVIALIYFLPLVVMFVAYSVIGLTLWRRSVPGHQAHGANLR--

------------------------HLQAKKKF-VKTMVLVVVTFAICWLPYHLYFILGTFQ-----EDIYCHK--FIQQVYLALFWLAMSSTMYNPI
IYCCLNHRFRSGFRLAFRCCPWVTPTEEDKMELTYTPSLSTRVNRCHTKEIFFMSGDVAPSEAVNGQAESPQAGVSTEP----
---</seq-data>
</sequence>
<sequence seq-type="Protein">
<seq-name>cckr_xenla</seq-name>
<seq-info><length>1066</length></seq-info>
<seq-data>---MESLRSLSNISALHELLC
RYSNLSGTLTWNLSSTNGTHNLTTANWPPWNLNCTPILDRKKPSPSDLNLWVRIVMYSVIFLLSVFGNTLIIIVLVMNKRLR
T---ITNSFLLSLALSDLMVAVLCMPFTLIPNLME--NFIFGEV------ICRAAAYFMGLSVSVSTFNLVAISIERYSAICNPLKSR--V
WQTRSHAYRVIAATWVLSSIIMIPYLVYNKTVTFP--MKDRRVGHQCRLVWP-----

----SKQVQQAWYVLLLTILFFIPGVVMIVAYGLISRELYRGIQFEMDLNKEAK------------AHKNGVSTPTTIPSGDEGDGCYI
QVTKRRNTMEMSTLTPSVCTKMDRARINNSEA---
---KLMAKKRV-IRMLIVIVAMFFICWMPIFVANTWKAF
D-----ELSAFNT--LTGAPISFIHLLSYTSACVNPLIYCFMNKRFRKAFLGTFSSCIKPCRNFRDTDEDIAATGASLSKFSYTTVSSL
GPA---</seq-data
>
</sequence>
<sequence seq-type="Protein">
<seq-name>gasr_human</seq-name>
<seq-info><length>1066</length></seq-info>
<seq-data>---MELLKLNRS
VQGTGPGPGASLCRPGAPLLNSSSVGNLSCEPPRIRGAGTR-ELELAIRITLYAVIFLMSVGGNMLIIVVLGLSRRLRT---VTN
AFLLSLAVSDLLLAVACMPFTLLPNLMG--TFIFGTV------ICKAVSYLMGVSVSVSTLSLVAIALERYSAICRPLQAR--VWQTR
SHAARVIVATWLLSGLLMVPYPVY--TVVQP--VGPRVLQCVHRWP---------------
---SAR
VRQTWSVLLLLLLLFFIPGVVMAVAYGLISRELYLGLRFDGDSDSDSQSRVRNQGGLPGAVHQNGRCRPETGAVGEDSDGC
YVQLPRSRPALELTALTAPGPGSGSRPTQA--
--KLLAKKRV-VRMLLVIVVLFFLCWLPVYSANTWRAFD--
----GPGAHRA-LSGAPISFIHLLSYASACVNPLVYCFMHRRFRQACLETCARCCPRPPRARPRALPDEDPPTPSIASLSRLSYTT
ISTLGPG---</seq-dat
a>

</sequence>
<sequence seq-type="Protein">
<seq-name>gpro_human</seq-name>
<seq-info><length>1066</length></seq-info>
<seq-data>--MLCPSKTDGSGHSGRIHQETHGEGKRDKISN
SEGRENGGRGFQMNGGSLEAEHASRMSVLRAKPMSNSQRLLLLSPGSPPRTGSI-----SYINIIMPSVFGTICLLGIIGNSTVIF
AVVKKSKLHWCNNVPDIFIINLSVVDLLFLLG-MPFMIHQLMGN-GVWHFGET------MCTLITAMDANSQFTSTYILTAMA
IDRYLATVHPISST--KFRKPSVATLVICLLWALSFISITPVWLYARLIPFP--GGAV
GCGIRLPN--
----------------------PDTDLYWFTLYQFFLAFALPFVVITAAYVRILQRMTS---
----
----------------------------------SVAPASQRSIRLRTKRV-TRTAIAICLVFFVCWAPYYVLQ-LTQLS-----ISRPTLT---FVYLYNAA
ISLGYANSCLNPFVYIVLCETFRKRLVLSVKPAAQGQLRAVSNAQTADEERTESKGT---
---</seq-data>
</sequence>
<sequence seq-type="Protein">
<seq-name>gals_human</seq-name>
<seq-info><length>1066</length></seq-info>
<seq-data>---
--------MNVSGCPGAGNASQAGGGGGW------HPEAVIVPLLFALIFLVGTVGNTLVLAVLLRGGQAVS---TTNLFILNLGVA
DLCFILCCVPFQATIYTLD--GWVFGSL------LCKAVHFLIFLTMHASSFTLAAVSLDRYLAIRYPLHSR--ELRTPRNALAAIGLI
WGLSLLFSGPYLS-YYRQS--QLANLTVCHPAWSAP-------------------------------
---RRRAMDICTFVF
SYLLPVLVLGLTYARTLRYLWRAVDPVAAGSG---

---ARRAKRKV-TRMILIVAALFCLCWMPHHALILCVWFG-----QFPLTRA---TYALRILSHLVSYANSCVNPIVYALVSKHFRK
GFRTICAGLLGRAPGRASGRVCAAARGTHSGSVLERESSDLLHMSEAAGALRPCPGASQPCILEPCPGPSWQGPKAGDSIL
TVDVA---</seq-data>
</sequence>
<sequence seq-type="Protein">
<seq-name>ssr1_human</seq-name>
<seq-info><length>1066</length></seq-info>
<seq-data>--MFPNGTASSPSSSPS
PSPGSCGEGGGSRGPGAGAADGMEEPGRNASQNGTLSEG--------QGSAILISFIYSVVCLVGLCGNSMVIYVILRYAKMKT
---ATNIYILNLAIADELLMLS-VPFLVTSTLLR--HWPFGAL------LCRLVLSVDAVNMFTSIYCLTVLSVDRYVAVVHPIKAA--R
YRRPTVAKVVNLGVWVLSLLVILPIVVFSRTAANSD-G---TVACNMLMPE-------

PAQRWLVGFVLYTFLMGFLLPVGAICLCYVLIIAKMRMVALKAGWQ--

-------------------------------QRKRSERKI-TLMVMMVMVFVICWMPFYVVQLVNVFA---------EQD---DATVSQLSVILGY
ANSCANPILYGFLSDNFKRSFQRILCLSWMDNAAEEPVDYYATALKSRAYSVEDFQPENLESGGVFRNGTCTSRITTL--------
--</seq-data>

</sequence>
<sequence seq-type="Protein">
<seq-name>oprk_human</seq-name>
<seq-info><length>1066</length></seq-info>
<seq-data>---MESPIQIFRGEPGPTC
APSACLPPNSSAWFPGWAEPDSNGSAGSEDAQLEPAHIS-------PAIPVIITAVYSVVFVVGLVGNSLVMFVIIRYTKMKT---
ATNIYIFNLALADALVTTT-MPFQSTVYLMN--SWPFGDV------LCKIVISIDYYNMFTSIFTLTMMSVDRYIAVCHPVKAL--D
FRTPLKAKIINICIWLLSSSVGISAIVLGGTKVREDVD--VIECSLQFPD-------------
--DDYS
WWDLFMKICVFIFAFVIPVLIIIVCYTLMILRLKSVRLLSGSR---

-------------------EKDRNLRRI-TRLVLVVVAVFVVCWTPIHIFILVEALG-------STSHST-AALSSYYFCIALGYTNSSLNPILYAFL
DENFKRCFR-DFCFPLKMRMERQSTSRVRNTVQDPAY-LRDIDGMNKPV--
--</seq-data>
</sequence>
<sequence seq-type="Protein">
<seq-name>ki01_human</seq-name>
<seq-info><length>1066</length></seq-info>
<seq-data>--
---------MINSTSTQPPDESCSQNL--------LITQQIIPVLYCMVFIAGILLNGVSGWIFFYVPSSKS----FIIYLKNIVIADFVMSLT-
FPFKILGDSGL-GPWQLNVF------VCRVSAVLFYVNMYVSIVFFGLISFDRYYKIVKPLWTS--FIQSVSYSKLLSVIVWMLMLL
LAVPNIILTNQSVR---EVTQIKCIELKSEL--
---GRKWHKASNYIFVAIFWIVFLL
LIVFYTAITKKIFK--
--SHLKSSRNSTSVKKKS-S
RNIFSIVFVFFVCFVPYHIARIPYTKS--QTEAHYSCQSKEILRYMKEFTLLLSAANVCLDPIIYFFLCQPFREILCKKLHIPLKAQN
DLDISRIKRGNTTLESTDTL---
---------------------</seq-data>
</sequence>
<sequence seq-type="Protein">
<seq-name>p2yr_human</seq-name>
<seq-info><length>1066</length></seq-info>
<seq-data>---MTEVLWPAVP
NGTDAAFLAGPGSSWGNSTVASTAAVSSSFKCALTKT-------GFQFYYLPAVYILVFIIGFLGNSVAIWMFVFHMKPWS---G
ISVYMFNLALADFLYVLT-LPALIFYYFNK-TDWIFGDA------MCKLQRFIFHVNLYGSILFLTCISAHRYSGVVYPLKSL--GRLK
KKNAICISVLVWLIVVVAISPILFYSGTGVR---KNKTITCYDTTSDE-----------------
--YLRSYFI
YSMCTTVAMFCVPLVLILGCYGLIVRALIY---

----KDLDNSPLRRKS-IYLVIIVLTVFAVSYIPFHVMKTMNLRARLDFQTPAMCAFNDRVYATYQVTRGLASLNSCVDPILYFL
AGDTFRRRLSRATRKASRRSEANLQSKSEDMTLNILPEFKQNGDTSL--
---</seq-data>

</sequence>
<sequence seq-type="Protein">
<seq-name>tda8_mouse</seq-name>
<seq-info><length>1066</length></seq-info>
<seq-data>---
----------------MAMNSMCIEEQR-------HLEHYLFPVVYIIVFIVSVPANIGSLCVSFLQAKKEN---ELGIYLFSLSLSDLLYALT-L
PLWINYTWNK-DNWTFSPT------LCKGSVFFTYMNFYSSTAFLTCIALDRYLAVVYPLKFS--FLRTRRFAFITSLSIWILESFFN
SMLLWKDETSVEYCDSD---KSNFTLCYDKYPLE---
--KWQINLNLFRTCMGYAIP
LITIMICNHKVYRAVRH---
---NQATENSEK
RRI-IKLLASITLTFVLCFTPFHVMVLIRCVL--ERDMNVNDKSGWQTFTVYRVTVALTSLNCVADPILYCFVTETGRADMWN
ILKLCTRKHNRHQGKKRDILSVSTRDAVELEIID--
---</seq-data>
</sequence>
<sequence seq-type="Protein">
<seq-name>thrr_mouse</seq-name>
<seq-info><length>1066</length></seq-info>
<seq-data>--MGPRRLLIVALGLSLCGPLLSSRVPMSQPESERTDATVNPRSFF
LRNPSENTFELVPLGDEEEEEKNESVLLEGRAVYLNISLPPHTPPPPFISEDASGYLTS-------PWLTLFMPSVYTIVFIVSLPLN
VLAIAVFVLRMKVKK---PAVVYMLHLAMADVLFVSV-LPFKISYYFSG-TDWQFGSG------MCRFATAAFYGNMYASIMLM
TVISIDRFLAVVYPIQSL--SWRTLGRANFTCVVIWVMAIMGVVPLLLKEQTTRVP---
----GLNITTCHDVLSEN--
----------------------------------LMQGFYSYYFSAFSAIFFLVPLIVSTVCYTSIIRCLSS--
----
--SAVANRSKKSRA-LFLSAAVFCIFIVCFGPTNVLLIVHYLF------LSDSPGTEAAYFA
YLLCVCVSSVSCCIDPLIYYYASSECQRHLYSILCCKESSDPNSCNSTGQLMPSKMDTCSSHLNNSIYKKLLA--------------------
--</seq-data>
</sequence>
<sequence seq-type="Protein">
<seq-name>o43495</seq-name>
<seq-info><length>1066</length></seq-info>
<seq-data>---
---------------MNGTYNTCGSSDLTWP----PAIKLGFYAYLGVLLVLGLLLNSLALWVFCCRMQQWT---ETRIYMTNLAVADL
CLLCT-LPFVLHSLRDT-SDTP----------LCQLSQGIYLTNRYMSISLVTAIAVDRYVAVRHPLRAR--GLRSPRQAAAVCAVLW
VLVIGSLVARWLLGIQE---GGFCFRST---
--RHNFNSMRFPLLGFYLP
LAVVVFCSLKVVTALAQRPPTDV--
---GQAE
ATRKA-ARMVWANLLVFVVCFLPLHVGLTVRLAV-----GWNACALLETIRRALYITSKLSDANCCLDAICYYYMAKEFQEASA
LAVAPRAKAHKSQDSLCVTLA---
------------------------------------</seq-data>

</sequence>
<sequence seq-type="Protein">
<seq-name>ccr4_human</seq-name>
<seq-info><length>1066</length></seq-info>
<seq-data>--ME
GISIYTSDNYTEEMGSGDYDSMKEPCFREENA------NFNKIFLPTIYSIIFLTGIVGNGLVILVMGYQKKLRS---MTDKYRLHLS
VADLLFVIT-LPFWAVDAVAN---WYFGNF------LCKAVHVIYTVNLYSSVLILAFISLDRYLAIVHATNSQ--RPRKLLAEKVVYV
GVWIPALLLTIPDFIFANVSE---ADDRYICDRFYPN--------------------------------
---DLWVVVFQFQHI
MVGLILPGIVILSCYCIIISKLSH--
---SKGH
QKRKA-LKTTVILILAFFACWLPYYIGISIDSFI-LLEIIKQGCEFENTVHKWISITEALAFFHCCLNPILYAFLGAKFKTSAQHALTS
VSRGSSLKILSKGKRGGHSSVSTESESSSFHSS--
------------------------------------</seq-data>
</sequence>
<sequence seq-type="Protein">
<seq-name>ccr3_human</seq-name>
<seq-info><length>1066</length></seq-info>
<seq-data>--MVLEVSDHQVL
NDAEVAALLENFSSSYDYGENESDSCCTSPPCPQDFSL------NFDRAFLPALYSLLFLLGLLGNGAVAAVLLSRRTALS---STD
TFLLHLAVADTLLVLT-LPLWAVDAAVQ---WVFGSG------LCKVAGALFNINFYAGALLLACISFDRYLNIVHATQLY--RRGP
PARVTLTCLAVWGLCLLFALPDFIFLSAHHDE---RLNATHCQYNFP---------------
---QV
GRTALRVLQLVAGFLLPLLVMAYCYAHILAVLLV--

---------------SRGQRRLRA-MRLVVVVVVAFALCWTPYHLVVLVDILM-DLGALARNCGRESRVDVAKSVTSGLGYMHCCL
NPLLYAFVGVKFRERMWMLLLRLGCPNQRGLQRQPSSSRRDSSWSETSEASYSGL--
--</seq-data>
</sequence>
<sequence seq-type="Protein">
<seq-name>ckr9_human</seq-name>
<seq-info><length>1066</length></seq-info>
<seq-data>--M
ADDYGSESTSSMEDYVNFNFTDFYCEKNNVR------QFASHFLPPLYWLVFIVGALGNSLVILVYWYCTRVKT---MTDMFLL
NLAIADLLFLVT-LPFWAIAAADQ---WKFQTF------MCKVVNSMYKMNFYSCVLLIMCISVDRYIAIAQAMRAHTWREKRL
LYSKMVCFTIWVLAAALCIPEILYSQIKE---ESGIAICTMVYPSD------------------
--ESTKLKS
AVLTLKVILGFFLPFVVMACCYTIIIHTLIQ---

------AKKSSKHKA-LKVTITVLTVFVLSQFPYNCILLVQTID-AYAMFISNCAVSTNIDICFQVTQTIAFFHSCLNPVLYVFVGER
FRRDLVKTLKNLGCISQAQWVSFTRREGSLKLSSMLLETTSGALSL--
--</seq-data>

</sequence>
<sequence seq-type="Protein">
<seq-name>ckr6_human</seq-name>
<seq-info><length>1066</length></seq-info>
<seq-data>---MSGESM
NFSDVFDSSEDYFVSVNTSYYSVDSEMLLCSLQEVR------QFSRLFVPIAYSLICVFGLLGNILVVITFAFYKKARS---MTDVYLL
NMAIADILFVLT-LPFWAVSHATG--AWVFSNA------TCKLLKGIYAINFNCGMLLLTCISMDRYIAIVQATKSFRLRSRTLPRS
KIICLVVWGLSVIISSSTFVFNQKYN--TQGSDVCEPKYQTVS---------------------
---EPIRWKLL
MLGLELLFGFFIPLMFMIFCYTFIVKTLVQ---

-------AQNSKRHKA-IRVIIAVVLVFLACQIPHNMVLLVTAAN--LGKMNRSCQSEKLIGYTKTVTEVLAFLHCCLNPVLYAFIG
QKFRNYFLKILKDLWCVRRKYKSSGFSCAGRYSENISRQTSETADNDNASSFTM---
---</seq-data>
</sequence>
<sequence seq-type="Protein">
<seq-name>il8a_human</seq-name>
<seq-info><length>1066</length></seq-info>
<seq-data>--MSNI
TDPQMWDFDDLNFTGMPPADEDYSPCM-LETE------TLNKYVVIIAYALVFLLSLLGNSLVMLVILYSRVGRS---VTDVYLLN
LALADLLFALT-LPIWAASKVNG---WIFGTF------LCKVVSLLKEVNFYSGILLLACISVDRYLAIVHATRTL---TQKRHLVKFVC
LGCWGLSMNLSLPFFLFRQAYHP---NNSSPVCYEVLGN---------------------------
---DTAKWRMVL
RILPHTFGFIVPLFVMLFCYGFTLRTLFK--

----AHMGQKHRA-MRVIFAVVLIFLLCWLPYNLVLLADTLM-RTQVIQETCERRNNIGRALDATEILGFLHSCLNPIIYAFIGQ
NFRHGFLKILAMHGLVSKEFLARHRVTSYTSSSVNVSSNL--
---</seq-data>
</sequence>
<sequence seq-type="Protein">
<seq-name>c3x1_human</seq-name>
<seq-info><length>1066</length></seq-info>
<seq-data>---
--MDQFPESVTENFEYDDLAEACYIGDIV------VFGTVFLSIFYSVIFAIGLVGNLLVVFALTNSKKPKS---VTDIYLLNLALSDLL
FVAT-LPFWTHYLINE---KGLHNA------MCKFTTAFFFIGFFGSIFFITVISIDRYLAIVLAANSM--NNRTVQHGVTISLGVWA
AAILVAAPQFMFTKQKE--NECLGDYPEV--
---LQEIWPVLRNVETNFLGF
LLPLLIMSYCYFRIIQTLFS--
---CKNHKKA
KA-IKLILLVVIVFFLFWTPYNVMIFLETLK--LYDFFPSCDMRKDLRLALSVTETVAFSHCCLNPLIYAFAGEKFRRYLYHLYGKC
LAVLCGRSVHVDFSSSESQRSRHGSVLSSNFTYHTSDGDALLLL---
---</seq-data>

</sequence>
<sequence seq-type="Protein">
<seq-name>ckr2_human</seq-name>
<seq-info><length>1066</length></seq-info>
<seq-data>--MLSTS
RSRFIRNTNESGEEVTTFFDYDYGAPCHKFDVK------QIGAQLLPPLYSLVFIFGFVGNMLVVLILINCKKLKC---LTDIYLLNLA
ISDLLFLIT-LPLWAHSAANE---WVFGNA------MCKLFTGLYHIGYFGGIFFIILLTIDRYLAIVHAVFAL--KARTVTFGVVTSVIT
WLVAVFASVPGIIFTKCQK---EDSVYVCGPYFP------------------------------------
--RGWNNFHTIMRNIL
GLVLPLLIMVICYSGILKTLLR--
--CRNEK
KRHRA-VRVIFTIMIVYFLFWTPYNIVILLNTFQ--EFFGLSNCESTSQLDQATQVTETLGMTHCCINPIIYAFVGEKFRSLFHIA
LGCRIAPLQKPVCGGPGVRPGKNVKVTTQGLLDGRGKGKSIGRAPEASLQDKEGA--
---</seq-data>
</sequence>
<sequence seq-type="Protein">
<seq-name>rdc1_human</seq-name>
<seq-info><length>1066</length></seq-info>
<seq-data>--MDLHLF
DYAEPGNFSDISWPCNSSDCIVVDTVMCPNMPNK------SVLLYTLSFIYIFIFVIGMIANSVVVWVNIQAKTTGY---DTHCYI
LNLAIADLWVVLT-IPVWVVSLVQH-NQWPMGEL------TCKVTHLIFSINLFSGIFFLTCMSVDRYLSITYFTNTP--SSRKKMV
RRVVCILVWLLAFCVSLPDTYYLKTVTSA---SNNETYCRSFYPEH------------------
--SIKEWLI
GMELVSVVLGFAVPFSIIAVFYFLLARAISA---

--------SSDQEKHSS-RKIIFSYVVVFLVCWLPYHVAVLLDIFS-ILHYIPFTCRLEHALFTALHVTQCLSLVHCCVNPVLYSFINRN
YRYELMKAFIFKYSAKTGLTKLIDASRVSETEYSALEQNAK--
---</seq-data>
</sequence>
<sequence seq-type="Protein">
<seq-name>ag22_rat</seq-name>
<seq-info><length>1066</length></seq-info>
<seq-data>---MKDNFS
FAATSRNITSSLPFDNLNATGTNESAFNCSHKPA-------DKHLEAIPVLYYMIFVIGFAVNIVVVSLFCCQKGPKK---VSSIYIFN
LAVADLLLLAT-LPLWATYYSYR-YDWLFGPV------MCKVFGSFLTLNMFASIFFITCMSVDRYQSVIYPFLS---QRRNPWQA
SYVVPLVWCMACLSSLPTFYFRDVRTIE---YLGVNACIMAFPPE-------------------
---KYAQWS
AGIALMKNILGFIIPLIFIATCYFGIRKHLLK---

-TNSYGKNRITRDQV-LKMAAAVVLAFIICWLPFHVLTFLDALT--WMGIINSCEVIAVIDLALPFAILLGFTNSCVNPFLYCFV
GNRFQQKLRSVFRVPITWLQGKRETMSCRKSSSLREMDTFVS---
--</seq-data>

</sequence>
<sequence seq-type="Protein">
<seq-name>ag2s_xenla</seq-name>
<seq-info><length>1066</length></seq-info>
<seq-data>--
---MLSNISAGENSEVEKIVVKCSKSGMH------NYIFITIPIIYSTIFVVGVFGNSLVVIVIYSYMKMKT---MASVFLMNLALSDL
CFVIT-LPLWAVYTAMH-YHWPFGDL------LCKIASTAITLNLYTTVFLLTCLSIDRYSAIVHPMKSR--IRRTVMVARLTCVGI
WLVAFLASLPSVIYRQIFIFP--DTNQTVCALVYHS------------------------------------
---GHIYFMVGMSLVKNI
VGFFIPFVIILTSYTLIGKTLKE--
---VYRAQR
ARNDDI-FKMIVAVVLLFFFCWIPHQVFTFLDVLI--QMDVIQNCKMYDIVDTGMPITICIAYFNSCLNPFLYGFFGKKFRKHF
LQLIKYIPPKMRTHASVNTKSSTVSQRLSDTKCASNKIALWIFDIEEHCK---
--</seq-data>
</sequence>
<sequence seq-type="Protein">
<seq-name>ag2s_mouse</seq-name>
<seq-info><length>1066</length></seq-info>
<seq-data>--
-----MILNSSIEDGIKRIQDDCPKAGRH------NYIFVMIPTLYSIIFVVGIFGNSLVVIVIYFYMKLKT---VASVFLLNLALADLCFL
LT-LPLWAVYTAME-YQWPFGNH------LCKIASASVSFNLYASVFLLTCLSIDRYLAIVHPMKSR--LRRTMLVAKVTCIIIWLM
AGLASLPAVIHRNVYFIE--NTNITVCAFHYES---
--QNSTLPIGLGLTKNILGFV
FPFVIILTSYTLIWKALKK---
---AYKIQKNTPRN
DDI-FRIIMAIVLFFFFSWVPHQIFSFLDVLI--QLGVIHDCEIADVVDTAMPITICIAYFNNCLNPLFYGFLGKKFKRYFLQLLKYI
PPKARSHAGLSTKMSTLSYRPSDNMSSSARKSAYCFEVE--
---</seq-data>
</sequence>
<sequence seq-type="Protein">
<seq-name>brb1_human</seq-name>
<seq-info><length>1066</length></seq-info>
<seq-data>--M
ASSWPPLELQSSNQSQLFPQNATACDNAPEAW-----DLLHRVLPTFIISICFFGLLGNLFVLLVFLLPRRQLN---VAEIYLANLA
ASDLVFVLG-LPFWAENIWNQ-FNWPFGAL------LCRVINGVIKANLFISIFLVVAISQDRYRVLVHPMASG--RQQRRRQAR
VTCVLIWVVGGLLSIPTFLLRSIQAVP--DLNITACILLLPH----------------------------
--EAWHFARIV
ELNILGFLLPLAAIVFFNYHILASLRT--
--REEVS
RTRVRGPKDSKT-TALILTLVVAFLVCWAPYHFFAFLEFLF--QVQAVRGCFWEDFIDLGLQLANFFAFTNSSLNPVIYVFVGR
LFRTKVWELYKQCTPKSLAPISSSHRKEIFQLFWRN---
---</seq-data>

</sequence>
<sequence seq-type="Protein">
<seq-name>apj_human</seq-name>
<seq-info><length>1066</length></seq-info>
<seq-data>--
----MEEGGDFDNYYGADNQSECEYTDW-------KSSGALIPAIYMLVFLLGTTGNGLVLWTVFRSSREKRR--SADIFIASLAVA
DLTFVVT-LPLWATYTYRD-YDWPFGTF------FCKLSSYLIFVNMYASVFCLTGLSFDRYLAIVRPVANA--RLRLRVSGAVATA
VLWVLAALLAMPVMVLRTTGDLE--NTTKVQCYMDYSMVA-T-----------------
---VSSEWAWE
VGLGVSSTTVGFVVPFTIMLTCYFFIAQTIAG--

----HFRKERIEGLRKRRRL-LSIIVVLVVTFALCWMPYHLVKTLYMLG---SLLHWPCDFDLFLMNIFPYCTCISYVNSCLNPFLY
AFFDPRFRQACTSMLCCGQSRCAGTSHSSSGEKSASYSSGHSQGPGPNMGKGGEQMHEKSIPYSQETLVVD--------------
---</seq-data>
</sequence>
<sequence seq-type="Protein">
<seq-name>gprf_macne</seq-name>
<seq-info><length>1066</length></seq-info>
<seq-data>--
-MDPEETSVYLDYYYATSPNPDIRETHSHV-----PYTSVFLPVFYTAVFLTGVLGN-LVLMGALHFKPGSRR--LIDIFIINLAASD
FIFLVT-LPLWVDKEASL-GLWRTGSF------LCKGSSYMISVNMHCSVFLLTCMSVDRYLAIVCPVVSR--KFRRTDCAYVVCA
SIWFISCLLGLPTLLSRELTLID--DKPYCAEKKAT-----------------------------------
---PLKLIWSLVALIFTFF
VPLLSIVTCYCCIARKLCA--
---HYQQSGKHNK
KLKKS-IKIIFIVVAAFLVSWLPFNTSKLLAIVS---GLQQERYFPSAILQLGMEVSGPLAFANSCVNPFIYYIFDSYIRRAIVHCLCP
CLKNYDFGSSTETSDSHLTKALSTFIHAEDFTRRRKRSVSL---
---</seq-data>
</sequence>
<sequence seq-type="Protein">
<seq-name>c3ar_human</seq-name>
<seq-info><length>1066</length></seq-info>
<seq-data>--
------------MASFSAETNSTDLLSQPWN----EPPVILSMVILSLTFLLGLPGNGLVLWVAGLKMQRT----VNTIWFLHLTLADLL
CCLS-LPFSLAHLALQ-GQWPYGRF------LCKLIPSIIVLNMFASVFLLTAISLDRCLVVFKPIWCQ--NHRNVGMACSICGCIW
VVAFVMCIPVFVYREIFTTD--NHNRCGYKFGLSSSLDYPDFYGDPLENRSLEN
IVQRPGEMNDRLDPSSFQTNDHPWTVPTVFQPQTFQRPSADSLPRGSARLTSQNLYSNVFKPADVVSPKIPSGFPIEDHET
SPLDNSDAFLSTHLKLFPSASSNSFYESELPQGFQDYYNLGQFTDDDQVPTPLVAITITRLVVGFLLPSVIMIACYSFIVFRMQ
RG---
--RFAKSQSKT-FRVAVVVVAVFLV
CWTPYHIFGVLSLLT------DPETPLGKTLMSWDHVCIALASANSCFNPFLYALLGKDFRKKARQSIQGILEAAFSEELTRSTHC
PSNNVISERNSTTV--

---------</seq-data>
</sequence>
<sequence seq-type="Protein">
<seq-name>fml1_macmu</seq-name>
<seq-info><length>1066</length></seq-info>
<seq-data>---
------NFSTPLSEYEEVSYESAGY----------TVLQILPLVVLGVTFVLGVLGNGLVIWVAGFRMTRT----VTTICYLNLALADFSFT
AT-LPFLIVSMAMG-EKWPFGWF------LCKLIHIVVDINLFGSVFLIGFIALDRCICVLHPVWAQ--NHRTVSLAMKVIVGPWI
LALVLTLPVFLFLTTVTIP--NGDTYCTFNFASWGGTP------------------------------
--EKRLKVAITMLTARGIIRF
VIGFSMPMSIVATCYGLIAAKIHKK---

GMIKSSRP-LRVLTAVVASFFICWFPFQLVALLSTVW---LKEILVDGKYKIINILVNPTSSLAFFNSCLNPMLYVFVGQDFRERL
IHSLPTSLERALSEDSAPTNDTAASCASPPAETELQAM---
---</seq-data>
</sequence>
<sequence seq-type="Protein">
<seq-name>fmlr_human</seq-name>
<seq-info><length>1066</length></seq-info>
<seq-data>---
---METNSSLPTNISGGTPAVSAGY----------LFLDIITYLVFAVTFVLGVLGNGLVIWVAGFRMTHT----VTTISYLNLAVADFC
FTST-LPFFMVRKAMG-GHWPFGWF------LCKFLFTIVDINLFGSVFLIALIALDRCVCVLHPVWTQ--NHRTVSLAKKVIIGP
WVMALLLTLPVIIRVTTVPGK---TGTVACTFNFSPWTNDP------------------------
--KERINVAVAMLTVR
GIIRFIIGFSAPMSIVAVSYGLIATKIHKQ--

----GLIKSSRP-LRVLSFVAAAFFLCWSPYQVVALIATVR----IRELLQGMYKEIGIAVDVTSALAFFNSCLNPMLYVFMGQDF
RERLIHALPASLERALTEDSTQTSDTATNSTLPSAEVALQAK---
--</seq-data>
</sequence>
<sequence seq-type="Protein">
<seq-name>oar2_lymst</seq-name>
<seq-info><length>1066</length></seq-info>
<seq-data>--MMSFPIALFADVNQSFRANLVVSSYHH
AITFPTVRGANFSTFFPRNFSVSADVWLCGANFSQEWQLMQPVCSTKYDS------ITIFITVAVVLTLITLWTILGNFFVLMAL
YRYGTLRT---MSNCLIGNLAISDLLLAVTVLPISTVHDLLG--YWVFGEF------TCTLWLCMDVLYCTASIWGLCTVAFDRYLA
TVYPVWYH--DQRSVRKAVGCIVFVWIFSIVISFAPFI-GWQHMIPSFFS--FNASIQRY
QCILFT---
------------------------SSSYVLYSSMGSFVIPAILMAFMYVRIFVVLHNQSRGVKFKSGLKISSSKYNGCPVINEPSREGINGLGR
DVTNTTLLSDAVGSSADLTSNGKDDPRVLATAPIELTEDVPPLNGHHHRTVHETPYVSGLHTKRSNSFALPTELEKKCKPLT
NNILHMMDFDRRNSHNAVIQRSASEMVNLDVSKHELLISNVCHRSKSATALTSETGDPLGSLAGPRRSLQCNVGGLVRNK
HMTL-----------------------------------SMKRRFELREQRA-TKRMLLIMACFCVCWMPFLFMYILRSVC-------DTCHM---NQH

FVAAIIWLGYVNSSLNPVLYTLFNDDFKVAFKRLIGARSPSAYRSPGPRR---
--</seq-data>
</sequence>
<sequence seq-type="Protein">
<seq-name>brs3_human</seq-name>
<seq-info><length>1066</length></seq-info>
<seq-data>--MAQRQPH
SPNQTLISITNDTESSSSVVSNDNTNKGWSGDNSPG------IEALCAIYITYAVIISVGILGNAILIKVFFKTKSMQT---VPNIFITS
LAFGDLLLLLTCVPVDATHYLAE--GWLFGRI------GCKVLSFIRLTSVGVSVFTLTILSADRYKAVVKPLERQ--PSNAILKTCVK
AGCVWIVSMIFALPEAIFSNVYTFRDPN---KNMTFESCTSYPVSK-----------------------
---KLLQEIHS
LLCFLVFYIIPLSIISVYYSLIARTLYK---
--STLNIPTEEQ
SHARKQIESRKRI-ARTVLVLVALFALCWLPNHLLYLYHSFT-----SQTYVDPSAMHFIFTIFSRVLAFSNSCVNPFALYWLSKSF
QKHFKAQLFCCKAERPEPPVADTSLTTLAVMGTVPGTGSIQMSEISVTSFTGCSVKQAEDRF------------------------------------
--</seq-data>
</sequence>
<sequence seq-type="Protein">
<seq-name>nmbr_human</seq-name>
<seq-info><length>1066</length></seq-info>
<seq-data>--MPSKSL
SNLSVTTGANESGSVPEGWERDFLPASDGTTTE------LVIRCVIPSLYLLIITVGLLGNIMLVKIFITNSAMRS---VPNIFISNLA
AGDLLLLLTCVPVDASRYFFD--EWMFGKV------GCKLIPVIQLTSVGVSVFTLTALSADRYRAIVNPMDMQ--TSGALLRTCV
KAMGIWVVSVLLAVPEAVFSEVARISSL---DNSSFTACIPYPQTD---------------------
--ELHPKIH
SVLIFLVYFLIPLAIISIYYYHIAKTLIK---
---SAHNLPGEY
NEHTKKQMETRKRL-AKIVLVFVGCFIFCWFPNHILYMYRSFN------YNEIDPSLGHMIVTLVARVLSFGNSCVNPFALYLLSE
SFRRHFNSQLCCGRKSYQER-GTSYLLSSSAVRMTSLKSNAKNMVTNSVL-LNGHSMKQEMAM-------------------------------
---</seq-data>
</sequence>
<sequence seq-type="Protein">
<seq-name>etbr_human</seq-name>
<seq-info><length>1066</length></seq-info>
<seq-data>---MQPPPSLCGRALVALVLACGLSRIWGEERGFPPDRATP
LLQTAEIMTPPTKTLWPKGSNASLARSLAPAEVPKGDRTAGSPPRTISPPPCQGPIEIK-----ETFKYINTVVSCLVFVLGIIGNS
TLLRIIYKNKCMRN---GPNILIASLALGDLLHIVIDIPINVYKLLAE--DWPFGAE------MCKLVPFIQKASVGITVLSLCALSIDRY
RAVASWSRIK--GIGVPKWTAVEIVLIWVVSVVLAVPEAIGFDIITMDY---KGSYLR
ICLLHPVQKTA---
------------------------FMQFYKTAKDWWLFSFYFCLPLAITAFFYTLMTCEMLR---
----
--------------------------------------KKSGMQIALNDHLKQRREV-AKTVFCLVLVFALCWLPLHLSRILKLTL-YNQNDPNRCELLS

FLLVLDYIGINMASLNSCINPIALYLVSKRFKNCFKSCLCCWCQSFEEKQSLEEKQSCLKFKANDHGYDNFRSSNKYSSS-------
---</seq-data>
</sequence>
<sequence seq-type="Protein">
<seq-name>gp39_human</seq-name>
<seq-info><length>1066</length></seq-info>
<seq-data>--
-----MASPSLPGSDCSQIIDHSHVPEFEVA----TWIKITLILVYLIIFVMGLLGNSATIRVTQVLQKKGYLQKEVTDHMVSLACS
DILVFLIGMPMEFYSIIWNPLTTSSYTL------SCKLHTFLFEACSYATLLHVLTLSFERYIAICHPFRYK--AVSGPCQVKLLIGFV
WVTSALVALPLLFAMGTEYPLVNVPSHRGLTCNRSSTRH-----------------------------HEQPETSNMSICTNLSS----------------
--RWT
VFQSSIFGAFVVYLVVLLSVAFMCWNMMQVLMKSQKGSLAGGTRPPQLRKSESE--

------------------------------------ESRTARRQT-IIFLRLIVVTLAVCWMPNQIRRIMAAAK---PKHDWTRSYFRAYMILLPFSETF
FYLSSVINPLLYTVSSQQFRRVFVQVLCCRLSLQHANHEKRLRVHAHSTTDSARFVQRPLLFASRRQSSARRTEKIFLSTFQSE
AEPQSKSQSLSLESLEPNSGAKPANSAAENGFQEHEV--</seq-dat
a>
</sequence>
<sequence seq-type="Protein">
<seq-name>ntr2_rat</seq-name>
<seq-info><length>1066</length></seq-info>
<seq-data>--
-----METSSPWPPRPSPSAGLSLEARLGVDTR--LWAKVLFTALYSLIFAFGTAGNALSVHVVLKARAGRPG--RLRYHVLSLAL
SALLLLLVSMPMELYNFVWSHYPWVFGDL------GCRGYYFVRELCAYATVLSVASLSAERCLAVCQPLRAR--RLLTPRRTRR
LLSLVWVASLGLALPMAVIMGQKHE--VESADGEPEPASRVCTVLVS------------------
--RATL
QVFIQVNVLVSFALPLALTAFLNGITVNHLMALYSQVPSASAQVSSIPSRLELLSEEGLLGFITWRKTLSLGVQASLVRHKDAS

--QIRSLQHS-AQVLRAIVAVYVICWLPYHARRLMYCYI---PDDGWTNELYDFYHY
FYMVTNTLFYVSSAVTPILYNAVSSSFRKLFLESLGSLCGEQHSLVPLPQEAPESTTSTYSFRLWGSPRNPSLGEIQV-----------
---</seq-data>
</sequence>
<sequence seq-type="Protein">
<seq-name>gp38_human</seq-name>
<seq-info><length>1066</length></seq-info>
<seq-data>--
MGSPWNGSDGPEGAREPPWPALPPCDERRCSPFP---LGALVPVTAVCLCLFVVGVSGNVVTVMLIGRYRDMRT---TTNL
YLGSMAVSDLLILLG-LPFDLYR-LWRSRPWVFGPL------LCRLSLYVGEGCTYATLLHMTALSVERYLAICRPLRAR--VLVTRR
RVRALIAVLWAVALLSAGPFLFLVGVEQDPGISVVPGLNGTARIASSPLASS------PPLWLSRAPPPSPPSGPETAEAAALFSR
ECRPSPA---
-----------------------QLGALRVMLWVTTAYFFLPFLCLSILYGLIGRELWSSRRPLRGPAASG---------------------------------------
----

---RERGHRQT-VRVLLVVVLAFIICWLPFHVGRIIYINT-------EDSRMMYFSQYFN
IVALQLFYLSASINPILYNLISKKYRAAAFKLLLARKSRPRG--FHRSRDTAGEVAGDTGGDTVGYTETSANVKTMG------------
--</seq-data>
</sequence>
<sequence seq-type="Protein">
<seq-name>ml1a_human</seq-name>
<seq-info><length>1066</length></seq-info>
<seq-data>---
-------MQGNGSALPNASQPVLRGDGARP-----SWLASALACVLIFTIVVDILGNLLVILSVYRNKKLRN---AGNIFVVSLAVAD
LVVAIYPYPLVLMSIFNN--GWNLGYL------HCQVSGFLMGLSVIGSIFNITGIAINRYCYICHSLKYD--KLYSSKNSLCYVLLIW
LLTLAAVLPNLRAGTLQYDP--RIYSCTFAQSV--------------------------------------
--SSAYTIAVVVFHFLV
PMIIVIFCYLRIWILVLQVRQRVKPDRKPKLK--

PQDFRN-FVTMFVVFVLFAICWAPLNFIGLAVASD-------PASMVPRIPEWLFVASYYMAYFNSCLNAIIYGLLNQNFRKEYR
RIIVSLCTARVFFVDSSNDVADRVKWKPSPLMTNNNVVKVDSV--
--</seq-data>
</sequence>
<sequence seq-type="Protein">
<seq-name>gprl_human</seq-name>
<seq-info><length>1066</length></seq-info>
<seq-data>---
--------MNSTLDGNQSSHPFCLLAFGYLET---VNFCLLEVLIIVFLTVLIISGNIIVIFVFHCAPLLNHH--TTSYFIQTMAYADLFV
GVSCVVPSLSLLHHP--LPVEESL------TCQIFGFVVSVLKSVSMASLACISIDRYIAITKPLTYN--TLVTPWRLRLCIFLIWLYSTL
VFLPSFF-HWGKPGY---HGDVFQWCAESWH---
---TDSYFTLFIVMMLYAP
AALIVCFTYFNIFRICQQHTKDISERQARFSSQSGETGEV---

---QACPDKRY-AMVLFRITSVFYILWLPYIIYFLLESST------------GHSNRFASFLTTWLAISNSFCNCVIYSLSNSVFQRGLKRL
SGAMCTSCASQTTANDPYTVRSKGPLNGCHI--
---</seq-data>
</sequence>
<sequence seq-type="Protein">
<seq-name>gprj_human</seq-name>
<seq-info><length>1066</length></seq-info>
<seq-data>---MVFAHRMDNSKPHLII
PTLLVPLQNRSCTETATPLPSQYLMELSEEHSWMSNQTDLHYVLK---PGEVATASIFFGILWLFSIFGNSLVCLVIHRSRRTQ
S---TTNYFVVSMACADLLISVASTPFVLLQFTTG--RWTLGSA------TCKVVRYFQYLTPGVQIYVLLSICIDRFYTIVYPLSF----
KVSREKAKKMIAASWIFDAGFVTPVLFFYGSN--WDSHCNYFLP------------
---SS
WEGTAYTVIHFLVGFVIPSVLIILFYQKVIKYIWRIGTDGRTVRRTMN---

------------------------IVPRTKVKT-IKMFLILNLLFLLSWLPFHVAQLWHPHE---------QD-YKKSSLVFTAITWISFSSSASKPTL
YSIYNANFRRGMKETFCMSSMKCYRSNAYTITTSSRMAKKNYVGISEIPSMAKTITKDSIYDSFDREAKEKKLAWPINSNPP
NTFV---</seq-data>
</sequence>
<sequence seq-type="Protein">
<seq-name>acm2_human</seq-name>
<seq-info><length>1066</length></seq-info>
<seq-data>---
-------------MNNSTNSSNNSLALTSPYK---TFEVVFIVLVAGSLSLVTIIGNILVMVSIKVNRHLQT---VNNYFLFSLACADLIIGV
FSMNLYTLYTVIG--YWPLGPV------VCDLWLALDYVVSNASVMNLLIISFDRYFCVTKPLTYP--VKRTTKMAGMMIAAAW
VLSFILWAPAIL-FWQFIVG--VRTVEDGECYIQFFS------------------------------------
--NAAVTFGTAIAAF
YLPVIIMTVLYWHISRASKSRIKKDKKEPVANQDPVSPSLVQGRIVKPNNNNMPSSDDGLEHNKIQNGKAPRDPVTENCV
QGEEKESSNDSTSVSAVASNMRDDEITQDENTVSTSLGHSKDENSKQTCIRIGTKTPKSDSCTPTNTTVEVVGSSGQNGDE
KQNIVARKIVKMTKQP---AKKKPPPSREKKV-TRTILAI
LLAFIITWAPYNVMVLINTFC--------APC---IPNTVWTIGYWLCYINSTINPACYALCNATFKKTFKHLLMCHYKNIGATR-----
----
-</seq-data>
</sequence>
<sequence seq-type="Protein">
<seq-name>5ht_lymst</seq-name>
<seq-info><length>1066</length></seq-info>
<seq-data>---MANFTFGDLALDVARMGGLASTPSGLRSTGLT
TPGLSPTGLVTSDFNDSYGLTGQFINGSHSSRSRDNASANDTSATNMTDDRYWSLTVYS--HEHLVLTSVILGLFVLCCIIGN
CFVIAAVMLERSLHN---VANYLILSLAVADLMVAVLVMPLSVVSEISK--VWFLHSE------VCDMWISVDVLCCTASILHLVAI
AMDRYWAVTS-IDYI--RRRSARRILLMIMVVWIVALFISIPPLF-GWRDPNN---D
PDKTGTCIISQD---
---------------------------------KGYTIFSTVGAFYLPMLVMMIIYIRIWLVARSRIRKDKFQMTKARLKTEETTLVASPKTEYSVV
SDCNGCNSPDSTTEKKKRRAPFKSYGCSPRPERKKNRAKKLPENANGVNSNSSSSERLKQIQIETAEAFANGCAEEASIAML
ERQCNNGKKISSNDT--PYSRTRE
KLELKRERKA-ARTLAIITGAFLICWLPFFIIALIGPFV--------DPE--GIPPFARSFVLWLGYFNSLLNPIIYTIFSPEFRSAFQKILF
GKYRRGHR---
----------------------</seq-data>
</sequence>
<sequence seq-type="Protein">
<seq-name>o76267</seq-name>
<seq-info><length>1066</length></seq-info>
<seq-data>---MSWHNISVQRMGRNASLLEAN
MYAMFPGYNITTGWPPRDNNSNNSGYFDQVTVNGSDRPELLRQYNYHEPKYE--EPVMIVICIFLGCMILAIILGNIFVITAI
LVEKSLQG---VSNYLILSLAVTDLLVAVLVMPLSLIYEISI--HWFLGNA------VCDMWVSMDVLCCTASILHLVAIAFDRYWA
VSN-IDYV--RSRNARQILLMVAIVWTVSVFISISPLF-GWRHD---SDDPELTGQCL
ISQDH---

---------------------GYTVFSTVGRFYCPLLLMLVINFKIYRAARYRIRKKRFGGRGGKHQALHVPLPAVTVDTSHRQILATLEG
DVSQDGISMYMPSCTNAMTSTRVEHGETARCPGEWSTRRPAGSKPDSHVVQHTDRPSMHLLASPYPVQEPRSRFQQQ
RHQKTE---THRQRVVFRREKM
EMARERKVWLRVLGIITGAFVVCWLPFFVVAVVKPMC-------GTPC--DMPSYVYSLFLWLGYVNSLINPIIYTIFNPSFRCAF
NKIFLRRIKSVNRIT--
---------------------------</seq-data>
</sequence>
<sequence seq-type="Protein">
<seq-name>5ht_bommo</seq-name>
<seq-info><length>1066</length></seq-info>
<seq-data>---MEGAEGQEELDWE
ALYLRLPLQNCSWNSTGWEPNWNVTVVPNTTWWQASAPFDTPAA----LVRAAAKAVVLGLLILATVVGNVFVIAAILLER
HLRS---AANNLILSLAVADLLVACLVMPLGAVYEVVQ--RWTLGPE------LCDMWTSGDVLCCTASILHLVAIALDRYWAVT
N-IDYI--HASTAKRVGMMIACVWTVSFFVCIAQLL-GWKDPDW---NQRVSEDLR
CVVSQD--
-----------------------VGYQIFATASSFYVPVLIILILYWRIYQTARKRIRRRRGATARGGVGPPPVPAGGALVAGGGSGGIAA
AVVAVIGRPLPTISETTTTGFTNVSSNNTSPEKQSCANGLEADPPTTGYGAVAAAYYPSL-------------------------------------
--VRRKPKEAADSKRERKA-AKTLAIIT
GAFVACWLPFFVLAILVPTC---------DC--EVSPVLTSLSLWLGYFNSTLNPVIYTVFSPEFRHAFQRLLCGRRVRRRRAPQ-----
----
</seq-data>
</sequence>
<sequence seq-type="Protein">
<seq-name>5ht_helvi</seq-name>
<seq-info><length>1066</length></seq-info>
<seq-data>--MNASRLPGFNDTSQ
DQPYPTSSEWFDGSNCSWVDAVSWGCNVTINGTSTNATSTDVTS----FVLMAVTSVVLALIILATIVGNVFVIAAIIIERNLQ
N---VANYLVASLAVADLMVACLVMPLGAVYEVSQ--GWILGPE------LCDMWTSSDVLCSSASILHLVAIATDRYWAVTD-
VDYI--HIRNEKRIFTMIVLVWGAALVVSLAPQL-GWKDPDY---LARITQQQKCLVS
QD--
-----------------LAYQIFATMSTFYVPLAVILILYWKIFQTARRRIRRRRDPPPPRPTSADGATPSGRPVQSARDRRFVKKRFLN
LKKCNQRTRAETLAAALLLTEGQSTSTVDTLDEEPRTTAFTINEKVPPSVSPEKSSSTVTNGSKPERAIVPAP------------------
--THREKKESLEAKRERKA-AKTLAIIT
GAFVFCWLPFFIMALVMPIC--------QTC--VISDYLASFFLWLGYFNSTLNPVIYTIFSPDFRQAFARILFGTHRRRRYKKF------
----
</seq-data>
</sequence>
<sequence seq-type="Protein">
<seq-name>o18512</seq-name>
<seq-info><length>1066</length></seq-info>
<seq-data>---MIDETLLNL
TASPSSQPATLLSAVARGTHLVDQFPAHAEIFSDIERP----LVQTVILASVLLVLILSCFIGNLFVILAIIMERDLRGR--PQYYLIFS

LAVADLLVGMIVTPLGAWFTVTG--TWNLGVV------VCDFWISVDVLVCTASILHLVAIALDRYWSITD-ICYV--QNRTPKRI
TLMLAVIWFTSLLISLAPFA-GWKDEGF--SDRVLKSHVCLISQQ---------------------
--ISYQ
VFSTATAFYIPLIAIICVYWKIMRAAKKRFKRERDRRTVIRPPPDAIDEKKAMMPKKSKKCPLPPAVVISDIQANGGTGGKTN
SIKNPPRHNESSSSASEEERTMTQTNHAPGDVTTQETKIDEENGRSKPGIVK--
--RRRRTKESNEMKRERKA-WRTLAIITGTFVACWTPFFLV
SIYRPIC---------GC--QISPVLEQVTLWLGYLNSALNPIIYTVFSQDFRAAFKRIIKRMCLIHDY------------------------------------
--</seq-data>
</sequence>
<sequence seq-type="Protein">
<seq-name>d2d1_xenla</seq-name>
<seq-info><length>1066</length></seq-info>
<seq-data>---
-MDPQNLSMYNDDI-----NNGTNGTAVDQ-----KPHYNYYAMLLTLLVFVIVFGNVLVCIAVSREKALQT---TTNYLIVSLAV
ADLLVATLVMPWAVYMEVVG--EWRFSRI------HCDIFVTLDVMMCTASILNLCAISIDRYTAVAMPMLYN-TRYSSKRRVT
VMISVVWVLSFAISCPLLF-GLNNTG---SKVCIIDNP-----------------------------
--AFVIYSSIV
SFYVPFIVTLLVYVQIYIVLRKRRKRVNTKRNSRGV------AVDAHKDKCTHPEDVKLCSVFVKSNGSFPADKKKVILVQEAGK
HPEDMEMEMMSSTS------PPEKTKHKS--ASPDHNQLAVPATSNQCKNASLTSPVESPYKAEKNGHPKDSTKPAKVFEIQS
MP--NGKTRTSIKTMSKKKLSQHKEKKA-TQMLAIVLG
VFIICWLPFFIIHILNMHC---------NC--NIPQALYSAFTWLGYVNSAVNPIIYTTFNVEFRKAFIKILHC---------------------------
--</seq-da
ta>
</sequence>
<sequence seq-type="Protein">
<seq-name>d2dr_human</seq-name>
<seq-info><length>1066</length></seq-info>
<seq-data>---
-MDPLNLSWYDDDLERQNWSRPFNGSDGKAD----RPHYNYYATLLTLLIAVIVFGNVLVCMAVSREKALQT---TTNYLIVSL
AVADLLVATLVMPWVVYLEVVG--EWKFSRI------HCDIFVTLDVMMCTASILNLCAISIDRYTAVAMPMLYN-TRYSSKRR
VTVMISIVWVLSFTISCPLLF-GLNNAD---QNECIIANP--------------------------
---AFVVYS
SIVSFYVPFIVTLLVYIKIYIVLRRRRKRVNTKRSSRAF---RAHLRAPLKGNCTHPEDMKLCTVIMKSNGSFPVNRRRV----EAA
RRAQELEMEMLSSTS------PPERTRYSP--IPPSHHQLTLPDPS----HHGLHSTPDSPAKPEKNGHAKDHPKIAKIFEIQTMP--
--NGKTRTSLKTMSRRKLSQQKEKKA-TQMLAIVLGVFIIC
WLPFFITHILNIHC---------DC--NIPPVLYSAFTWLGYVNSAVNPIIYTTFNIEFRKAFLKILHC-----------------------------------
---</seq-data>
</sequence>
<sequence seq-type="Protein">
<seq-name>d2dr_fugru</seq-name>
<seq-info><length>1066</length></seq-info>
<seq-data>---

-MDVFTQYAYNDSI-FDNGTWSANETTKDE-----THPYNYYAMLLTLLIFVIVFGNVLVCMAVSREKALQT---TTNYLIVSLAV
ADLLVATLVMPWVVYLEVVG--EWRFSKI------HCDIFVTLDVMMCTASILNLCAISIDRYTAVAMPMLYN-TRYSSRRRVT
VMISVVWVLSFAISCPLLF-GLNNTA--TRDQSLCFIANP--------------------------
---AFVVYS
SIVSFYVPFIVTLLVYVQIYVVLRKRRKRVNTKPKQRLCQAADPDIPTSLKDKCTHPEDVRLCTMIVKSNGSFPVNKKKVIFIKD
GVNEVEGLELDELNYCGGSHKQPPPQQQPRALGDTPATSHQLLMSTKANASPTSTPPTPPEEGQRTEKNGDPTKEAQ-G
NPAPVVALR--NGKTQTSLKTLSKRKISQQKEKKA-TQ
MLAIVLGVFIICWLPFFITHILNTHC--------TRC--KVPAEMYNAFTWLGYVNSAVNPIIYTTFNVEFRKAFIKILHC-------------
----
--</seq-data>
</sequence>
<sequence seq-type="Protein">
<seq-name>5h6_human</seq-name>
<seq-info><length>1066</length></seq-info>
<seq-data>---
---------MVPEPGPTANSTPAWGAGPPSA----PGGSGWVAAALCVVIALTAAANSLLIALICTQPALRN---TSNFFLVSLFTSD
LMVGLVVMPPAMLNALYG--RWVLARG------LCLLWTAFDVMCCSASILNLCLISLDRYLLILSPLRYK--LRMTPLRALALVL
GAWSLAALASFLPLLLGWHELGHA---RPPVPGQCRLLASL--------------------------
--PFVLVAS
GLTFFLPSGAICFTYCRILLAARKQAVQVASLTTGMASQASETLQVPRTPRPGVE--

-----------SADSRRLATKHSRKALKA-SLTLGILLGMFFVTWLPFFVANIVQAVC---------DC---ISPGLFDVLTWLGYCNSTMN
PIIYPLFMRDFKRALGRFLP-CPRCPRERQASLASPSLRTSHSGPRPGLSLQQVLPLPLPPDSDSDSDAGSGGSSGLRLTAQLL
LPGEATQDPPLPTRAAAAVNFFNIDPAEPELRPHPLGIPTN---</seq-data>
</sequence>
<sequence seq-type="Protein">
<seq-name>aa2a_human</seq-name>
<seq-info><length>1066</length></seq-info>
<seq-data>---
-----------------------MP----------IMGSSVYITVELAIAVLAILGNVLVCWAVWLNSNLQN---VTNYFVVSLAAADIAVGVLAIP
FAITISTG---FCAACH-------GCLFIACFVLVLTQSSIFSLLAIAIDRYIAIRIPLRYN--GLVTGTRAKGIIAICWVLSFAIGLTPML-G
WNNCGQPKEGKNHSQG--CGEGQVACLFEDVV---
--PMNYMVYFNFFACVLVPLL
LMLGVYLRIFLAARRQLKQMESQPLPGERAR---
--ST
LQKEVHA-AKSLAIIVGLFALCWLPLHIINCFTFFC--------PDC-SHAPLWLMYLAIVLSHTNSVVNPFIYAYRIREFRQTFRKIIR
SHVLRQQEPFKAAGTSARVLAAHGSDGEQVSLRLNGHPPGVWANGSAPHPERRPNGYALGLVSGGSAQESQGNTGLPD
VELLSHELKGVCPEPPGLDDPLAQDGAGVS--</seq-data>
</sequence>
<sequence seq-type="Protein">
<seq-name>aa1r_human</seq-name>
<seq-info><length>1066</length></seq-info>

<seq-data>--
--------------------MPP--SI-----SAFQAAYIGIEVLIALVSVPGNVLVIWAVKVNQALRD---ATFCFIVSLAVADVAVGALVIP
LAILINIG---PQTYFH-------TCLMVACPVLILTQSSILALLAIAVDRYLRVKIPLRYK--MVVTPRRAAVAIAGCWILSFVVGLTP
MF-GWNNLSAVERAWAANGS-----------------------------------MGEPVIKCEFEKVI-----------------------------------
--SMEYMVYFNFFVWVL
PPLLLMVLIYLEVFYLIRKQLNKKVSASSGDPQ---
--K
YYGKELKI-AKSLALILFLFALSWLPLHILNCITLFC--------PSC--HKPSILTYIAIFLTHGNSAMNPIVYAFRIQKFRVTFLKIWND
HFRCQPAPPIDEDLPEERPDD--
---------------------------</seq-data>
</sequence>
<sequence seq-type="Protein">
<seq-name>o17496</seq-name>
<seq-info><length>1066</length></seq-info>
<seq-data>--
-------------MSEWQFTHVRVPVATR-----QTSNVLALLLLPLLCAVGLLGNLLVCMAIWFDRRLHN---VTNYFLFSLALADLF
VCSIVMPLSLLVEVRH-GVWTWSFS------VCLLYVYADVFLCTASIVHMSMISLDRFLGISRPLKI---RNRSRTMTTLKITFVW
LITILISCPIAIAALIDPT---NILQR-NSCAISNR---------------------------------------
---YYMVYGSTFAFLIPFIV
MAVTYTKTTTLLKKQASQLSQRASNRSNGLRRTLPHRRLGQSSMSNGMLHKNSVNIGGYSSLRGTHPPLLIEDELQLTALP
SDSKNKEERGKLRRIGARTSSAIFAV---
-------------------------------TSRISRKSSLLITSQDLANEHKA-TRVLAVVFGCFFVCWTPFFAANFAVGFC-------GESC--A
VPPGIASLFLWLGYVSSTINPLIYTIFNRRFRQAFLRIIRCQCLRPWRELSSMDYSRNYTYYPGDTYTWSNVERTAKMGGVC
KFGEYWSNVSCRSRNEFVLTDRRQLDNVKTTSESRPLLCGKHCNSSSGNSLQNNSARDQHDDISLNTLPANHSKYTDNDN
KYLTINERLAALC---------------------</seq-data>
</sequence>
<sequence seq-type="Protein">
<seq-name>5h2c_human</seq-name>
<seq-info><length>1066</length></seq-info>
<seq-data>---MVNLRNAVH
SFLVHLIGLLVWQCDISVSPVAAIVTDIFNTSDGGRFKFP----DGVQNWPALSIVIIIIMTIGGNILVIMAVSMEKKLHN---AT
NYFLMSLAIADMLVGLLVMPLSLLAILYD-YVWPLPRY------LCPVWISLDVLFSTASIMHLCAISLDRYVAIRNPIEH--SRFNS
RTKAIMKIAIVWAISIGVSVPIPVIGLRDEE---KVFVNNTTCVLNDP---------------
--N
FVLIGSFVAFFIPLTIMVITYCLTIYVLRRQALMLLHGHTEEPPGLSLDFLKCCKRNTAEEENSANPNQDQNARRRK----------
----
---------------------------KKERRPRGTMQAINNERKA-SKVLGIVFFVFLIMWCPFFITNILSVLC-------EKSCNQKLMEKLL
NVFVWIGYVCSGINPLVYTLFNKIYRRAFSNYLRCNYK---VEKKPPVRQIP
RVAATALSGRELNVNIYRHTNEPVIEKASDNEPGIEMQ--VENLELPVNPSSVVSERISSV----------------------</seq-data
>
</sequence>
<sequence seq-type="Protein">

<seq-name>5h2a_human</seq-name>
<seq-info><length>1066</length></seq-info>
<seq-data>--MDILCEENTSLSSTTNSLMQLN
DDTRLYSNDFNSGEANTSDAFNWTVDSENRTNLSCEGCLSPSCLSLLH----LQEKNWSALLTAVVIILTIAGNILVIMAVSLEK
KLQN---ATNYFLMSLAIADMLLGFLVMPVSMLTILYG-YRWPLPSK------LCAVWIYLDVLFSTASIMHLCAISLDRYVAIQN
PIHH--SRFNSRTKAFLKIIAVWTISVGISMPIPVFGLQDDS--KVFKE-GSCLLADD

---------------NFVLIGSFVSFFIPLTIMVITYFLTIKSLQKEATLCVSDLGTRAKLASFSFLPQSSLSSEKLFQRSIHRE---------------
----
-----------------------------------PGSYTGRRTMQSISNEQKA-CKVLGIVFFLFVVMWCPFFITNIMAVIC-------KESCNEDVIG
ALLNVFVWIGYLSSAVNPLVYTLFNKTYRSAFSRYIQCQYK--ENKKP-LQL
ILVNTIPALAYKSSQLQMGQKKNSKQDAKTTDNDCSMVALGKQHSEEASKDNSDGVNEKVSCV------------------------</s
eq-data>
</sequence>
<sequence seq-type="Protein">
<seq-name>5ht2_aplca</seq-name>
<seq-info><length>1066</length></seq-info>
<seq-data>---
-------------MLCGRLRHTMNSTTCFF-----SHRTVLIGIVGSLIIAVSVVGNVLVCLAIFTEPILSHS--KSKFFIVSLAVADLLLALL
VMTFALVNSLYG--YWLFGET------FCFIWMSADVMCETASIFSICVISYNRLKQVQKPLQYE--EFMTTTRALLIIASLWICSF
VVSFVPFFLEWHELSMEEIKTIFKDLISDKVKTSDAHTFSFAL-EQTLGDNR--------------TSNPKPECLFDVHF-----------------
---IY
SVIYSLFCFYIPCTLMLRNYLRLFLIAKKHHVRIKNLHRLH--

----------RNQGTQGSKA-ARTLTIITGTFLACWLPFFIINPIEAVD-----------EHLIPLECFMVTIWLGYFNSCVNPIIYGTSNSKF
RAAFQRLLRCRSVKSTVSSISPVASVYRAFSWIRPSLLDGPPSAVCDTGQDENRKGGGCVTTIPTESHVIISEEEIRANVMLSE
SDTVFS---</seq-data>
</sequence>
<sequence seq-type="Protein">
<seq-name>b3ar_human</seq-name>
<seq-info><length>1066</length></seq-info>
<seq-data>---
MAPWPHENSSLAPWPDLPTLAPNTANTSGLPGV----PWEAALAGALLALAVLATVGGNLLVIVAIAWTPRLQT---MTNVF
VTSLAAADLVMGLLVVPPAATLALTG--HWPLGAT------GCELWTSVDVLCVTASIETLCALAVDRYLAVTNPLRYG--ALVT
KRCARTAVVLVWVVSAAVSFAPIMSQWWRVGADA--EAQRCHSNPRCCAFASNM

--------------PYVLLSSSVSFYLPLLVMLFVYARVFVVATRQLRLLRGELGRFPP-EESPPAPSRSLAPAPVGTCAPPEGV---------
----
---PACGRRPARLLPLREHRA-LCTLGLIMGTFTLCWLPFFLANVLRALG----------GPSLVP
GPAFLALNWLGYANSAFNPLIY-CRSPDFRSAFRRLLCRCGRRLPPEPCAAARPALFPSGVPAARSSPAQPRLCQRLDGASW
GVS---</seq-data>
</sequence>

<sequence seq-type="Protein">
<seq-name>b1ar_human</seq-name>
<seq-info><length>1066</length></seq-info>
<seq-data>---MGAGVLVLGASE
PGNLSSAAPLPDGAATAARLLVPASPPASLLPPASESPEPLS----QQWTAGMGLLMALIVLLIVAGNVLVIVAIAKTPRLQT--
-LTNLFIMSLASADLVMGLLVVPFGATIVVWG--RWEYGSF------FCELWTSVDVLCVTASIETLCVIALDRYLAITSPFRYQ--S
LLTRARARGLVCTVWAISALVSFLPILMHWWRAESD--EARRCYNDPKCCDFVTNR-

------------AYAIASSVVSFYVPLCIMAFVYLRVFREAQKQVKKIDSCERRFLGGPARPPSPSPSPVPAPAPPPGPPRPAAAAA
TAPLANGR--
---AGKRRPSRLVALREQKA-LKTLGIIMGVFTLCWLPFFLANVVKAFH----------
-RELVPDRLFVFFNWLGYANSAFNPIIY-CRSPDFRKAFQGLLC-CARRAARRRHATHGDRPRASGCLARPGPPPSPGAASD
DDDDDVVGATPPARLLEPWAGCNGGAAADSDSSLDEPCRPGFASESKV---
----------------</seq-data>
</sequence>
<sequence seq-type="Protein">
<seq-name>aa3r_human</seq-name>
<seq-info><length>1066</length></seq-info>
<seq-data>---
---------------------MPNNSTAL----SLANVTYITMEIFIGLCAIVGNVLVICVVKLNPSLQT---TTFYFIVSLALADIAVGVLVMP
LAIVVSLG---ITIHFY-------SCLFMTCLLLIFTHASIMSLLAIAVDRYLRVKLTVRYK--RVTTHRRIWLALGLCWLVSFLVGLTP
MF-GWNMKLTSEYH---RNVTFLSCQFVSVM---
--RMDYMVYFSFLTWIFIPLV
VMCAIYLDIFYIIRNKLSLNLSNSKETG---
--AFYGRE
FKT-AKSLFLVLFLFALSWLPLSIINCIIYFN------------GEVPQLVLYMGILLSHANSMMNPIVYAYKIKKFKETYLLILKACVVC
HPSDSLDTSIEKNSE--
-------------------</seq-data>
</sequence>
<sequence seq-type="Protein">
<seq-name>mshr_human</seq-name>
<seq-info><length>1066</length></seq-info>
<seq-data>--M
AVQGSQRRLLGSLNSTPTAIPQLGLAANQTGAR----CLEVSISDGLFLSLGLVSLVENALVVATIAKNRNLHS---PMYCFICCL
ALSDLLVSGTNVLETAVILLLE--AGALVARAAVLQQLDNVIDVITCSSMLSSLCFLGAIAVDRYISIFYALRYH--SIVTLPRAPR
AVAAIWVASVVFSTLFIA-YYDHV---
--AVLLCLVVFFLA
MLVLMAVLYVHMLARACQ--
--HAQGIARLHKRQ
RPVHQGFGLKG-AVTLTILLGIFFLCWGPFFLHLTLIVLC-------PEHPTCGCIFKNFNLFLALIICNAIIDPLIYAFHSQELRRTLKE
VLTCSW---
----------------------</seq-data>

</sequence>
<column-score>
<colsco-name>coreblock</colsco-name>
<colsco-owner>0</colsco-owner>
<colsco-type>int</colsco-type>
<colsco-data>0 0 0 0 0 0 0 0 0 0 0 0 0 0 0 0 0 0 0 0 0 0 0 0 0 0 0 0 0 0 0 0 0 0 0 0 0 0 0 0 0 0 0 0 0 0 0 0 0 0 0 0 0
0 0 0 0 0 0 0 0 0 0 0 0 0 0 0 0 0 0 0 0 0 0 0 0 0 0 0 0 0 0 0 0 0 0 0 0 0 0 0 0 0 0 0 0 0 0 0 0 0 0 0 0 0 0 0 0 0 0 0 0
0 0 0 0 0 0 0 0 0 0 0 0 0 0 0 0 0 0 0 0 0 0 0 0 0 0 0 0 0 0 0 0 0 0 0 0 0 0 0 0 0 0 0 0 0 0 0 0 0 0 0 0 0 0 0 0 1 1 1 1
1 1 1 1 1 1 1 1 1 1 1 1 1 1 1 1 1 1 1 1 1 1 0 0 0 0 0 0 0 0 0 0 0 0 0 1 1 1 1 1 1 1 1 1 1 1 1 1 1 1 1 1 1 1 1 1 1 1 1 1
1 0 0 0 0 0 0 0 0 0 0 0 0 0 0 0 0 0 0 0 1 1 1 1 1 1 1 1 1 1 1 1 1 1 1 1 1 1 1 1 1 1 1 1 1 1 1 1 1 1 1 1 0 0 0 0 0 0 0 0
0 0 0 0 0 0 0 0 1 1 1 1 1 1 1 1 1 1 1 1 1 1 1 1 0 0 0 0 0 0 0 0 0 0 0 0 0 0 0 0 0 0 0 0 0 0 0 0 0 0 0 0 0 0 0 0 0 0 0 0
0 0 0 0 0 0 0 0 0 0 0 0 0 0 0 0 0 0 0 0 0 0 0 0 0 0 0 0 0 0 0 0 0 0 0 0 0 0 0 0 0 0 0 0 0 0 0 0 0 0 0 0 0 0 0 0 0 0 0 0
0 0 0 0 0 0 0 0 0 0 0 0 0 0 0 0 0 0 0 0 0 0 0 0 0 0 0 0 0 0 0 0 0 0 0 0 0 0 0 0 0 0 0 0 0 0 0 0 0 0 0 0 0 0 0 0 0 0 0 0
0 0 0 0 0 0 0 0 0 0 0 0 0 0 0 0 0 0 0 0 0 0 0 0 0 0 0 0 0 0 0 0 0 0 0 0 0 0 0 0 0 0 0 0 0 0 0 0 0 0 0 0 0 0 0 0 0 0 0 0
0 0 0 0 0 0 0 0 0 0 0 0 0 0 0 0 0 0 0 0 0 1 1 1 1 1 1 1 1 1 1 1 1 1 1 1 1 1 1 1 1 1 1 1 1 1 1 0 0 0 0 0 0 0 0 0 0 0 0 0
0 0 0 0 0 0 0 0 0 0 0 0 0 0 0 0 0 0 0 0 0 0 0 0 0 0 0 0 0 0 0 0 0 0 0 0 0 0 0 0 0 0 0 0 0 0 0 0 0 0 0 0 0 0 0 0 0 0 0 0
0 0 0 0 0 0 0 0 0 0 0 0 0 0 0 0 0 0 0 0 0 0 0 0 0 0 0 0 0 0 0 0 0 0 0 0 0 0 0 0 0 0 0 0 0 0 0 0 0 0 0 0 0 0 0 0 0 0 0 0
0 0 0 0 0 0 0 0 0 0 0 0 0 0 0 0 0 0 0 0 0 0 0 0 0 0 0 0 0 0 0 0 0 0 0 0 0 0 0 0 0 0 0 0 0 0 0 0 0 0 0 0 0 0 0 0 0 0 0 0
0 0 0 0 0 0 0 0 0 0 0 0 0 0 0 0 0 0 0 0 0 0 0 0 0 0 0 0 0 0 0 0 0 0 0 0 0 0 0 0 0 0 0 0 0 0 0 0 0 0 0 0 0 0 0 0 0 0 0 0
0 0 0 0 0 0 0 1 1 1 1 1 1 1 1 1 1 1 1 1 1 1 1 1 1 1 1 1 1 1 1 1 1 1 0 0 0 0 0 0 0 0 0 0 0 0 0 0 0 0 0 1 1 1 1 1 1 1 1 1
1 1 1 1 1 1 1 1 1 1 1 1 1 1 1 1 1 1 1 1 1 1 1 1 1 0 0 0 0 0 0 0 0 0 0 0 0 0 0 0 0 0 0 0 0 0 0 0 0 0 0 0 0 0 0 0 0 0 0 0
0 0 0 0 0 0 0 0 0 0 0 0 0 0 0 0 0 0 0 0 0 0 0 0 0 0 0 0 0 0 0 0 0 0 0 0 0 0 0 0 0 0 0 0 0 0 0 0 0 0 0 0 0 0 0 0 0 0 0 0
0 0 0 0 0 0 0 0 0 0 0 0 0 0 0 0 0 0 0 0 0 0 0 0 0 0 0 0 0 0 0 0 0 0 0 0 0 0 0 0 0 0 0 0 0 0 0 0 0 0 0 0 0 0 0 0 0 0 0 0
0 0 0 0 0 0 0 0 0 0 0 </colsco-data>
</column-score>
</alignment>
</macsim>
::::::::::::::
Nat_core-block.xml
::::::::::::::
<?xml version="1.0"?>
<!DOCTYPE macsim SYSTEM "http://www-bio3d-igbmc.u-strasbg.fr/macsim.dtd">
<macsim>
<alignment>
<aln-name>1</aln-name>
<sequence seq-type="Protein">
<seq-name>CITT_ECOLI</seq-name>
<seq-info><length>1375</length></seq-info>
<seq-data>--

---MSLAKDNIWKLLAPLVVMGVMFLIPVPDGMP-------PQAWHYFAVFV

AMIVGMILE--PIPATAISFIAVTICVIGSNYLLFDAKELADPAFNAQKQALKWGLAGFSSTTVWLVFGAFIFALGYEVSGLGR
RIALFLVKFMGKRTLTLGYAIVIIDILLAPFTPSNTARTGGTVFPVIKNLPPLFKSFPNDPSAR-----------------------------------
---RIGGYLMWMMVISTSLSSSMFVTGAAPNVLGLEFVSKIAGIQ----ISW
LQWFLCFLPVGVILLIIAPWLSYVLYKP--
---EITHSEEVATWAGDELKTMG-ALT
RREWTLIGLVLLSLGLWVFG-----------------------------SEVINATAVGLLAVSLMLAL-----------------------HVVPW-KDIT
RYNSAWNTLVNLATLVVMANGLTRSGFIDWFAGTMSTHLEG---FSPNATVIVLVLVFYFAHYLFASLSAHTATMLPVILAV
GKGIPGVP---------------MEQLCILLVLSIGIMGCLTPYATGPGVIIYGCG------YVKSKDYWRLGAIFGVIYISMLLLVGWPILA
MWN---------------------------------</seq-data>
</sequence>
<sequence seq-type="Protein">
<seq-name>TTDT_ECOLI</seq-name>
<seq-info><length>1375</length></seq-info>
<seq-data>---

--MKPSTE-WWRYLAPLAVIAIIALLPVPAGLE-------NHTWLYFAVFTGVI
VGLILE--PVPGAVVAMVGISIIAILSPWLLFSPEQLAQPGFKFTAKSLSWAVSGFSNSVIWLIFAAFMFGTGYEKTGLGRRIAL
ILVKKMGHRTLFLGYAVMFSELILAPVTPSNSARGAGIIYPIIRNLPPLYQSQPNDSSSR--
--SIGSYIMWMGIVADCVTSAIFLTAMAPNLLLIGLMKSASHAT----LSWGD
WFLGMLPLSILLVLLVPWLAYVLYPP--
---VLKSGDQVPRWAETELQAMG-PLC
SREKRMLGLMVGALVLWIFG-----------------------------GDYIDAAMVGYSVVALMLLL-----------------------RIISW-DDI
VSNKAAWNVFFWLASLITLATGLNNTGFISWFGKLLAGSLSG---YSPTMVMVALIVVFYLLRYFFASATAYTSALAPMMIA
AALAMPEIP--------------LPVFCLMVGAAIGLGSILTPYATGPSPIYYGSG------YLPTADYWRLGAIFGLIFLVLLVITGLLWM
PVVLL--------------------------------</seq-data>
</sequence>
<sequence seq-type="Protein">
<seq-name>O84207</seq-name>
<seq-info><length>1375</length></seq-info>
<seq-data>---

---MNKHKRFLSLVLLTFILLGIWFCPHSDLID-------SKAWHLFAIFTTTIIGII
VQ--PAPMGAIVIMGISLLLVTKTLT-----------------LDQALSGFHSPITWLVFLSFSIAKGVIKTGLGERVAYFFVKILGKSPLG
LSYGLVLTDFLLAPAIPSLTARAGGILFPVVMGLSESFGSSVEKGTEK---
-------------------------------LLGSFLIKVAYQSSVITSAMFLTAMAGNPIISALASH-SGVT----LTWAIWAKTAILPGIISLACM
PFVLFKLFPP--
--QITSCEEAVATAKTRLKEMG-PLNQGERIILLIFSLLISLWTFG-
---------------------------DSIGISATTTTFIGLSLLILT-----------------------NILDWQKDVLSNTTAWETFFWFGALIMMASFL
SAFGFIHFVGDSVIGSVQG---LSWKIGFPILFLIYFYSHYLFASNTAHIAAMYPIFLTVSISLGAN---------------PMFAALALAF
ASNLFGGLTHYGSGPAPLYFGSH------FVSVQEWWRSGFILSIVNLTIWLGLGSWWWYCLGLIR----------------------------

--</seq-data>
</sequence>
<sequence seq-type="Protein">
<seq-name>SOT1_SPIOL</seq-name>
<seq-info><length>1375</length></seq-info>
<seq-data>---

---MASMALSLTSSPTYSLSFRSLPS
LKPLSKSQPSISLPSLRSNASKSPSLSHKHFLSPPSLLLPHKLKPISASSPTNPPPPPAPVPSPAPVSAPAQVQPWQGASIKPLL
ASILTGVIIWFIPTPEGVS-------RNAWQLLAIFLSTIVGIITQ--PLPLGAVALMGLGASVLTKTLT------------------FSAAFSAF
GDPIPWLIALAFFFARGFIKTGLGNRIAYQFVKLFGSSSLGLGYSLVFSEALLAPAIPSVSARAGGIFLPLVKSLCIACGSNVGD
GTER--KLGAWLMLTCFQTSVISSSMFLT
AMAANPLSATLTFNTIGKA----IGWMDWAKAAFVPGLVSLIVVPLLLYVVYPP---

----EIKSSPDAPRLAKEKLDKMG-PMTKNESIMAVTLLLTVGLWVFG------------------------------GKLGVDAVTAAILGLSVL
LIT------------------------GVVTW-KECLAESVAWDTLTWFAALIAMAGYLNKYGLITWFSENVVKVVGG-LGLSWQMSFGV
LVLLYFYSHYFFASGAAHIGAMFTAFLSVASALGTP----------------PFLAAIVLSFLSNLMGGLTHYGIGSAPVFYGAN------Y
VPLPQWWGYGFLISIVNLIIWLGVGGLWWKAIGLW--------------------------------</seq-data>
</sequence>
<sequence seq-type="Protein">
<seq-name>YBHI_ECOLI</seq-name>
<seq-info><length>1375</length></seq-info>
<seq-data>---

--MNKKSL--WKLILILAIPCIIGFMPAPAGLS-------ELAWVLFGIYLAAIVGL
VIK--PFPEPVVLLIAVAASMVVVGNLSDG------------AFKTTAVLSGYSSGTTWLVFSAFTLSAAFVTTGLGKRIAYLLIGKIGN
TTLGLGYVTVFLDLVLAPATPSNTARAGGIVLPIINSVAVALGSEP-EKSPR---
--------------------------------------RVGHYLMMSIYMVTKTTSYMFFTAMAGNILALKMINDILHLQ----ISWGGWALAAGL
PGIIMLLVTPLVIYTMYPP---
---EIKKVDNKTIAKAGLAELG-PMKIREKMLLGVF
VLALLGWIFS------------------------------KSLGVDESTVAIVVMATMLLL------------------------GIVTW-EDVVKNKGGWNT
LIWYGGIIGLSSLLSKVKFFEWLAEVFKNNLAF--DGHGNVAFFVIIFLSIIVRYFFASGSAYIVAMLPVFAMLANVSGAP-------
---------LMLTALALLFSNSYGGMVTHYGGAAGPVIFGVG------YNDIKSWWLVGAVLTILTFLVHITLGVWWWNMLIGW
NML-----------------------------</seq-data>
</sequence>
<sequence seq-type="Protein">
<seq-name>YBHI_HAEIN</seq-name>
<seq-info><length>1375</length></seq-info>
<seq-data>---

--MALSKN--TKLVILMAIPLITFLLPAPDGLS-------LIAWRLLGVYIATIVGL
VLK--PYGEPVILLAAIAVSGVIIGNTEGA----------KELVKVGNMLDGYKSGTTWLIFTAFTLSSAFVITGLGKRIAYHMIGAM
GSTTLRLGYVTMFLDLLLSPATPSNTARSGGIIFPIINSVVVALGSDP-EKSPK---
---------------------------------------KAGRYLMMNVYMVVKTTSYIFLTAMAPNALALSLMAPILGFE----TTWIKWFLAASV
PGLLCLFLIPLICYWVSPP--
--ELKKVDNKAIAKKGLEELG-PMSFREKALSVLFV
IALFGWIFS-----------------------------NSLHINATIVAIIVMVLCIVL-----------------------SIVTW-DDILKSKGAWNTLVWY
GGIIGMSGLLEKSGFFKWLANTLSTILQF--EGHGMMALIVILTLSVSVRYLFASGGAYVAAMVPVFATVGHVTGAP---------
-------TELLALGLVFANSYGGSVTHYGGGPGPIAFGAG------YNDIKSWWITGAIIAFGSLIIHLTIGMAWWKLLMSLGWL---
--------------------------</seq-data>
</sequence>
<sequence seq-type="Protein">
<seq-name>MEMP_ALCEU</seq-name>
<seq-info><length>1375</length></seq-info>
<seq-data>--

-----------------------------MTDTSASPPSAPSAPSAPSAAAAQAVVPAPKLTLHWGLFAAVAALLVVLAIPQPEGLT-------VA
GQRMLAILAFAIVLWITE--AVSYETSAIMITSLMAGLIGFAPTV---NDPSVQYGTSKALGMALAGFSNTALALVAAALFISAA
MTVTGLDRRIALVTLSAIGTSTRRILIGTIAVTIALSLVVPSATARSAC-VVPIMMGVIAAFGVDKKS----------------------------
---NIAAGIMITVAQATSIWNVGIQTAAAQNLLTVGFMDKLLG
ER----ITWLQWLIAGA-PWAIAMSVVLYFLVRLLLP--
---AETDAIP-GGKEAVQRE
LSALG-PMSAPQKRLAAVSLGLLLFWATE----------------------------GKLHSFDTATVTFVGLVILMMPRI---------------------
GVMDW-KTMQQ-RTPWGTLIVFGVGISLGTALLSTNAGQWLGQFVVTHSGLAA-HGALLVFAILSAFLILIHLGFASATALT
AALLPILIAVLQTLPGDI---------------NRVGMTMLLGFTVSFGFILPINAPQNMVCLGTE------TFNGRQFARIGIPVTIIGYA
MMLLFAATYWRWLGWV--------------------------------</seq-data>
</sequence>
<sequence seq-type="Protein">
<seq-name>Q9Z6C0</seq-name>
<seq-info><length>1375</length></seq-info>
<seq-data>--

--MSLWLTYPLLLPSLIVGVTIVLWATS--LLPEF
ITALLFFTAAMTARIAP-----------------PEVIFGGFASSAFWLVFSGFVLGVAIRKTGLADRAARALSAKLTDSWVLMVASVV
LLSYALAFVMPSNMGR-IALLMPIVAAMAKRAGIADGS--
----------------------RAWFGLALAVGFGTFQLSATILPANVPNLVMSGAAEGSYGI----HLNYVPYLLLHTPVLGILKGLILIGLI
CWLFP---
---GSPKPPKEQAPSD-PMGRDEKRLAWLLAVVLGMWVTE----
-------------------------SWHGVGPAWTGLAASIVVMLPRV---------------------GFITG-EEFSA-GVNMRTCIYVAGILGLAIV
VTQTGIGTAVGEALLRIM-------PLDADRPFTSFLALTGITTALNFIMTANGVPALYTTLAQSFSDATG-------------FPLLSVIM

IQVLGYSTPLLPYQASPIVVAMGLG------KVPARAGMVLCLALAIATYLILLPLDYLWFSLLGKV--------------------------------
</seq-data>
</sequence>
<sequence seq-type="Protein">
<seq-name>O59712</seq-name>
<seq-info><length>1375</length></seq-info>
<seq-data>MKFSHSLQFNAVPEWSESYIAYSNLKKLIYSLEHEQITLQQGAPDEETRLLEHERRSPDDRFMFALDKELQGI--
-------------------------------------VEFYAPKEKEIADQYGRIKGEFETYENEYMSQGNNINYPTPERLQKSSASRKSGRMARS
QELPRITSSNREIYLNGQTSDGGYAAPAISRAESTAIQPSEPHDVDTSKNGLSKKQHSEAQPEVQGNDDEVEEEDDDDDDE
DEDEDEDEDNNNNNRWLLIEQYPSDIVAYENFVSLKRKLTQLYVSIHDLISYVHLNYTGFSKILKKY----DKTLGSSLRESYMK
RVNQAYPFLPATGKTLSKRLNIVAEWYAKLCCQGDTFVAIRRLRGHLREYVAWERNTIWREMMAMER--------------------
-----RTQAARL-SGLKPVAADEKESEQPPYFTIKTKFGVFRIPRCFFNSTIATLITIIVIFILLLSFPVIDN-------REQNNCLALLVM
VSLLWATE--AIPLFVTSFLVPFMTVFLKILRDEN------GSPLSGKESTKVIFSSMWNPTIVLLLGGFTIAAALSKYHIAKRLATSI
LAHAGRKPRSVLLTNMFVAMFASMWI-SNVA-APVLCFSIIQPLLRNLPAESD---
--FAKILIVGIALASNVGGIASPISSPQNIVALQNMDPA--------AGWGEWFAVSI-
PVSLLCIFSIWFLLSFGLL--
---KDEHITLAKIRSTKDTFTGVQWFISIVTIGTIV
LWCLERRF--------------------------DEVFGDMGVIALVPIIVFFGT-----------------------GLLTK-EDFN--NFLWTVIVLAMG
GVALGKVVSSSGLLELIALKIGNAVSS----LNTFRVLLIFSALTLVVSSFISHIVAAMVVLPIVHEVGSRLADP----------------HPR
LFVLASGMMCSLAMALPTSGFPNMTAIMME------NEAGKRYLKVSDFLKAGIPATLISFVILLLIGTPIMRALGF--------------
--------------</seq-data>
</sequence>
<sequence seq-type="Protein">
<seq-name>YN86_YEAST</seq-name>
<seq-info><length>1375</length></seq-info>
<seq-data>MKFSHSLQFNSVPEWSTKYLAYSQLKKLIYSLQKDKLYSNNKHHVVEPHDANDENLPLLADASPDDQFYISKF-
-------------------VAALNQELKKIDKFYISQETGLIANYNELKDDVMELENTNKATQLFNQQQQHQLQSVARNRKSKSQQRQ
RRFSSVSSTDSNPSLTDMSIDSAPVIHTQVSNTTNNGNSMQNLASASVSL-------------------------SNSNPVYLSPFTQHRLS
LKKRLISIYTQLSELKDFIELNQTGFSKICKKFDKSLNTNLKQNYLNYIKFHSHVFNPATINRIQHHITETILTYASLNKGTRRPSN
TFNLDADRINNDENSSGNEEDEDGNRQEVLDFQDAERELSSHLRDHVVWERNTVWKDMMNLERKYQSAKTDNKKFSK
LSSSQLRPNANITESMAMSSGGAGIIAPSTDSLTFRELMHLPPKQWLQFIMGQTSLLKFLLITSCFIALLTFNLTPFTQD-------
SLQKNCFAILIYASLLWATE--TIPLFVTSLMIPLLIVVFPVIKDPI-----TSQPMSPRDSSQFILSTMWSSVIMLLLGGFTLAAALS
KYNIAKVLSTHILASAGTNPHFILLTNMFVALFVSMWV-SNVA-APVLCYSIVQPLLRTLPRNCS-------------------------------
--YAKALILGIALASNIGGMSSPIASPQNIFSIGIMDPS--------PS
WAEWFMIAL-PVCFICVMAIWVLLIITFP---
--PEPNVKILQLHPSRDPFTLK
QWFVTLVCIITIVLWCLSNQI--------------------------SGIFGEMGIISIIPIVVFFGT-----------------------GLLTS-DDFN--NF
MWTIVVLAMGGTTLGKAVSSSGLLSTMAQLIKAQVEH----EPIFIIVLIFGLVILVMATFVSHTVAAMIIVPLMSEIGSNLPSG
D---------------HSRLLIVIAALLCSSAMGLPTSGFPNVTAISMI------DEVGDRYLTVGTFITRGVPASLLSYAAIVTVGYGILKV
MGF----------------------------</seq-data>
</sequence>
<sequence seq-type="Protein">

<seq-name>YJT8_YEAST</seq-name>
<seq-info><length>1375</length></seq-info>
<seq-data>MRFSHFLKYNAVPEWQNHYMDYSELKNLIYTLQTDELQVGDNEEGFGAGKSSNITDRFKNKFSFKNAKEDTS
S--------------------GMNKDAGIVEETIELRELPTAQTVAAKPSPFRRMKEKIFYKRRSSSASSVSSTANENLQLDTYDTFVGDL
TAEKQKVDDFYKRTEAKFYDKFDALVKDLKKIGVIEYDIDDDTLFNEPI-------------------------ASTNDEVPPLDLDDDEDDD
EFYDDQSNIEDNTALLHHSQYNIKSQKKSLLKKSIVNLYIDLCQLKSFIELNRIGFAKITKKSDKVLHLNTRTELIESEQFFKDTYA
FQAETIELLNSKISQLVTFYARITDRPHNISHSKQELKSYLHDHIVWERSNTWKDMLGLLS------------------QADELTPKETEY
NANKLVGKLDLEYYRWPLPRPINLKFTSINNVALPKLFFTKKAYKIYFIILVTGLLLGIKTFND-------AAQHRCMALVECVAFL
WASE--AIPLHITAFLVPLLVVLFKVLKTSD------GAIMSAASASSEILAAMWSSTIMILLAGFTLGEVLAQYNIAKVLASWLLA
FAGCKPRNVLLMAMCVVFFLSMWI-SNVA-APVLTYSLLSPLLDAMDADSP--
---FAQALVLGVALAANIGGMSSPISSPQNIISMSYLKPYG-------IGWGQFFAVAL-
PSGILAMLLVWILLFTTFK--
---MNKTKLEKFKPIKTKFTVKQYYIITVTVATI
LLWCVESQI--------------------------EGAFGSSGQIAIIPIVLFFGT-----------------------GLLST-QDLN--AFPWSIVILAMGGI
ALGKAVSSSGLLSTIAKALQKKIEN----DGVFAILCIFGILMLVVGTFVSHTVSAIIIIPLVQEVGDKLGNPK---------------AAPIL
VFGCALLSSCGMGLASSGFPNVTAISKV------DRKGDRYLSVMTFLTRGVPASILAFLCVITLGYGIMASVVKGNATSA-------
---------------</seq-data>
</sequence>
<sequence seq-type="Protein">
<seq-name>PH87_YEAST</seq-name>
<seq-info><length>1375</length></seq-info>
<seq-data>MRFSHFLKYNAVPEWQNHYLDYNELKNLIYTLQTDELKQETPTGDLNDDADSQTPGPIADIESNIAAGEPSPS
KRRFTHKLKRKLFGSKTPSGSKRGDSDEKAIDGNNINEETIELDELSPQGKTTSFNKNFIRKKFFESRSSSVSSEGKTLFSSYDTF
VTNLSDEKLKVDDFYKRMEAKFYERFDHLINDLEKEGIVTRLNETFNPEIQALPPLREIISGTSETHSSNNPFEIHSSNIDSELR
NRFDYSEEEMDEDDDVDVFADTTDNTALLNYSQFNIKSQKKSLLKQTIINLYIDLCQLKSFIELNRMGFSKITKKSDKVLHMN
TRQELIESEEFFKDTYIFQHETLSSLNSKIAQLIEFYAVLMGQPGNVDSCKQELKSYLHDHIVWERSNTWKDMLGLSS--------
----------QNNDIITIEDEAEKL-MQEKLQIEYFKYPLPKPINLKFTKIENLAVPKLFFGKRAMKIGFIIIVTGVLLGVKTFND-------
PVEHRCMALVECCAFLWASE--AIPLHITGLLVPLLTVLFRVLKDDD------GKVMGAAAASTEILGTMWSSTIMILLAGFTLG
EALSQYNVAKVLASWLLALAGTKPRNVLLMAMSVVFFLSMWI-SNVA-SPVLTYSLLTPLLDPLDYTSP------------------------
---FAKALVMGVALSADIGGMASPISSPQNIISMQYLKPY
G-------IGWGQFFAVAL-PTGILSMLCSWALMILTFK---
--IGKTKLEKFKPI
RTRFTIKQYFIIIVTIATILLWCVESQI--------------------------ESAFGSSGEIAVIPIVLFFGT-----------------------GLLST-KDF
N--TFPWSIVVLAMGGIALGKAVSSSGLLVTIARALQKKIQN----DGVFAILCIFGILMLVVGTFVSHTVSAIIIIPLVQEVGDKLS
DPK---------------AAPILVFGCALLASCGMGLASSGFPNVTAISMT------DKKGNRWLTVGAFISRGVPASLLAFVCVITLGYG
ISSSVLKGST-------------------------</seq-data>
</sequence>
<sequence seq-type="Protein">
<seq-name>Q9S4F3</seq-name>
<seq-info><length>1375</length></seq-info>
<seq-data>---

---MKRNLISIVLTLLTVGILFLIQVRFGLP-------INIAIMNCIFVLAAVFWV
LE--PIPGYATSILVIFLEIIFFANPIGV---SYFEFPRNKNPSTAVFLSSLADSSIVLFLGGFVLAKACVKTGLDRFFANRIIRRFGVK
SHQVLLGFMFTTGFISMWM-SNTA-TTSMMIALSFPMLQMLPEKEP--
--FRKALILGIPFAANIGGVGTPIGSPPNIIAMGILRQEGI-----VVSFGTWMFFAV-PLVIV
LILFAWLLLMKLFP---
--QKDLLELVVEFQEPEEG-TKSFSFRITTAIFFGTVLLW
FTE-----------------------------NLHGIPAGVVALLPLILFPVF-----------------------GILNE-KDIN--SLDWSVLLLIAGGIGIGVG
FQQSGMKFWFSEFLKPIIKP----EYSVSVLFLLCVLALLLSTFMSNTAATNLLVPFAFPLSTIVLPESNV-------------YLLEVCLGI
ALSASLAMSLPVSTPPNAIAYSVG------GFEIKDMIQAGLPIGILGLILVLAGYFIFL--------------------------------------</seq-
data>
</sequence>
<sequence seq-type="Protein">
<seq-name>Q9ZGJ3</seq-name>
<seq-info><length>1375</length></seq-info>
<seq-data>--

---MKRDLILIIGTLLLIGILFFVQVLFEFP-------LSVAIMICIFVLAAVFWVA
E--PIPGYATLILVIFLEIIFFANPIGI---PGFVFPQNKNPSAAVFLSSLADSSIILFLGGFVLAKACVKTGLDRFFANRIIRKFGVKS
HQVLLGFMFTTGFISMWM-SNTA-TTSMMIALSFPLLRVLPEKEP---
------------------------------------FRKALILGIPFAANIGGVGTPIGSPPNIIAMGILRQQGI-----VIPFGTWMFFAV-PLVVVLIL
FAWILLMKLFP--
---QKDSLDLVVEFQEPERG-MKSFSFRVTSMIFLGTVVLW
FTE-----------------------------NLHGVPAGVVALLPLILFPMF----------------------GILNE-KDIN--SLDWSVLILISGGIGIGV
GFQQSGMGPWFSGILKHIAKP----EYAISVLFFLCVLTLLLSTFMSNTAATNLLVPFAFPFSAILFPGSNV-------------YLLEVCL
GIALSASLAMSLPVSTPPNAIAYSVG------GIEIKDMICAGLPIGIFGLIVVLSSYFAFF--------------------------------------</se
q-data>
</sequence>
<sequence seq-type="Protein">
<seq-name>NASU_RAT</seq-name>
<seq-info><length>1375</length></seq-info>
<seq-data>--

--MKLLNYAFVYRRFLLVVFTVLVLLPLPLIIR-------SKEAECAYILFVIATFWI
TE--ALPLSITALLPGLMFPMFGIMS------------------STHVASAYFKDFHLLLIGVICLATSIEKWNLHKRIALRMVMMVGVN
PAWLTLGFMSSTAFLSMWL-SNTS-TAAMVMPIVEAVAQQITSAEAEAEATQMTYFNESAAQGLEVDETIIGQETNERKE
KTKPALGSSNDKGKVSSKMETE-----------------------------KNTVTGAKYRSKKDHMMCKLMCLCIAYSSTIGGLTTITGTSTN
LIFSEHFNTRYPDC--RCLNFGSWFLFSF-PVAVILLLLSWIWLQWLFL--
--GFNFKE-MFKCG
KTKTLKEKACAEVIKQ-EYEKLG-PMRYQEIVTLVIFIVMALLWFSRDPGFVTGW-SVLFSEY--------------PGYVTDSTVALVA

GILFFLIPAKKLTKMTSTG-----DIIAFDYSPLITW-KEFQS-FMPWDIAILVGGGFALADGCQVSGLSSWIGSKLSPLGSL-----P
VWLIILISSLIVTSLTEVASNPATITILFPILSPLAEAIHV-----------------NPLHILLPSTLCTSFAFLLPVANPPNAIVFSYG------HL
KVIDMVKAGLGVNILGVAVVMLGMFTWIEPMFNLHEYPSWAPDIVNQTMP-----------------</seq-data>
</sequence>
<sequence seq-type="Protein">
<seq-name>Q9UKG4</seq-name>
<seq-info><length>1375</length></seq-info>
<seq-data>--

---MGLLQGLLRVRKLLLVVCVPLLLLPLPVLHP-------SSEASCAYVLIVTAVY
WVSE--AVPLGAAALVPAFLYPFFGVLR------------------SNEVAAEYFKNTTLLLVGVICVAAAVEKWNLHKRIALRMVLMA
GAKPGMLLLCFMCCTTLLSMWL-SNTS-TTAMVMPIVEAVLQELVSAEDEQLVAGNSNTEEAEPISLDVKNSQPSLELIFVN
EDRSNADLTTLMHNENLNGVPSITNPIKTANQHQGKKQHPSQEKPQVLTPSPRKQKLNRKYRSHHDQMICKCLSLSISYSA
TIGGLTTIIGTSTSLIFLEHFNNQYPAA--EVVNFGTWFLFSF-PISLIMLVVSWFWMHWLFL--------------------------------------
----
--GCNFKE-TCSLSKKKKTKREQLSEKRIQEEYEKLG-DISYPEMVTGFFFILMTVLWFTREPGFVPGW-DSFFEK--------------
KGYRTDATVSVFLGFLLFLIPAKKPCFGKKNDGEN--QEHSLGTEPIITW-KDFQK-TMPWEIVILVGGGYALASGSKSSGLST
WIGNQMLSLSSL-----PPWAVTLLACILVSIVTEFVSNPATITIFLPILCSLSETMHI-----------------NPLYTLIPVTMCISFAVML
PVGNPPNAIVFSYG------HCQIKDMVKAGLGVNVIGLVIVMVAINTWGVSLFHLDTYPAWARVSNITDQA----------------
</seq-data>
</sequence>
<sequence seq-type="Protein">
<seq-name>Q9W7I2</seq-name>
<seq-info><length>1375</length></seq-info>
<seq-data>--

---MKISVVSKKLWCVHKQLILLIAPLIFLPLIFTLP-------EKEGRCLYVVLLMATF
WCTE--ALPLAVTAMLPVCLFPTLGIIP------------------SKQICPQYFLETNFLFLSGLVMASSIEEWGLHRRIALKVLSIVGVKP
AWLIFGMMMTSAFLSMWL-SNTA-TTAMMLPIANAILESLFGDLETLKKNCKSPKDSDSDIINGQCTLKMHSLPAVPVEK
QLLSIEGTDGPEPADVRT----------------------------------AEEIRSESEYQLKVWKGFLICIPYAASIGGTATLTGTAPNLILIGQL
KSYFPDC--DLINFGSWFAFAF-PLMLIFLFVGWLWIAYLYG--
--GLNTR--LCIQRHDRRAQA
EARAQALIQDDYRKLG-PMNFAQGAIAFFFVLFAVLLFTRDPKFITGW-SVFFK----------------KGYVSDAVTGVIIVSILFFFP
SQKPSLNWWFDP----QASNTRYVPLLSW-QKAQD-SIPWNIILLLGGGFAMAKACEESGLASWIGGHLQPLAEV-----PPAA
AVMLITAFLACFTEFASNTATIIIFLPVIAELAIRVSV-----------------HPLYFMIPATVGCSYAFMLPVSTPPNSIAFASG------H
LMVKDMVKTGFVMNILGILCVSLAMNTWGVAMFDLHTYPDWAHPINKTAAAEVVQALNATL-------</seq-data>
</sequence>
<sequence seq-type="Protein">
<seq-name>Q9Z0Z5</seq-name>
<seq-info><length>1375</length></seq-info>

<seq-data>--

---MAALAALAKKVWSARRLLVLLLVPLALLPILFALP-------PKEGRCLYVILLMA
VYWCTE--ALPLSVTALLPIILFPFMGILP------------------SSKVCPQYFLDTNFLFLSGLIMASAIEERNLHRRIALKVLMLVGV
QPARLILGMMVTTSFLSMWL-SNTA-STAMMLPIASAILKSLFGQRDTRKDLPREGEDSTAAVRGNGLRTVPTEMQFLASS
EGGHAEDVEAP---LELPDDSKEEEHRRNIWKGFLISIPYSASIGGTATLTGTAPNLILLGQL
KSFFPQC--DVVNFGSWFIFAF-PLMLLFLLVGWLWISFLYG--
--GMSWRG-WRKKNSKLQ
DVAEDKAKAVIQEEFQNLG-PIKFAEQAVFILFCLFAILLFSRDPKFIPGW-ASLFA----------------PGFVSDAVTGVAIVTILFFF
PSQKPSLKWWFDF----KAPNSETEPLLSW-KKAQE-TVPWNIILLLGGGFAMAKGCEESGLSAWIGGQLHPLEHV-----PPLL
AVLLITVVIAFFTEFASNTATIIIFLPVLAELAIRLHV-----------------HPLYLMIPGTVSCSYAFMLPVSTPPNSIAFSTG------HLL
VKDMVRTGLLMNLMGVLLLSLAMNTWAQAIFQLGTFPDWANTHAANVTALPPALTNNTVQTL----</seq-data>
</sequence>
<sequence seq-type="Protein">
<seq-name>NDC1_HUMAN</seq-name>
<seq-info><length>1375</length></seq-info>
<seq-data>--

--MATCWQALWAYRSYLIVFFVPILLLPLPILVP-------SKEAYCAYAIILMAL
FWCTE--ALPLAVTALFPLILFPMMGIVD------------------ASEVAVEYLKDSNLLFFGGLLVAIAVEHWNLHKRIALRVLLIVG
VRPAPLILGFMLVTAFLSMWI-SNTA-TSAMMVPIAHAVLDQL---HSSQASSNVEEGSN
NPTFELQEPSPQK-EVTKL---DNGQAL-PVTSASSE-GRAHLSQK--HLHLTQCMSLCVCYSASIGGIATLTGTAPNLVLQGQI
NSLFPQNG-NVVNFASWFSFAF-PTMVILLLLAWLWLQILFL---
--GFNFRKNFGIGE--KMQE
QQQAAYCVIQTEHRLLG-PMTFAEKAISILFVILVLLWFTREPGFFLGWGNLAFPNAKG------------ESMVSDGTVAIFIGIIM
FIIPSKFPGLTQDPEN----PGKLKAPLGLLDW-KTVNQ-KMPWNIVLLLGGGYALAKGSERSGLSEWLGNKLTPLQSV-----PA
PAIAIILSLLVATFTECTSNVATTTIFLPILASMAQAICL-----------------HPLYVMLPCTLATSLAFMLPVATPPNAIVFSFG------
DLKVLDMARAGFLLNIIGVLIIALAINSWGIPLFSLHSFPSWAQSNTTAQCLPSLANTTTPSP------</seq-data>
</sequence>
<sequence seq-type="Protein">
<seq-name>NDC1_RABIT</seq-name>
<seq-info><length>1375</length></seq-info>
<seq-data>--

--MATCWQGLWAYRMYLLVFLLPISLLPLPILVP-------RKEAYCAYAIILMA
LFWCTD--ALPLAVTALLPLCLFPMMGIME-------------------ASEVGLEYLKDTNVLFIGGLLLAIAVEHWNLHKRIALRVLLLT
GVRPALLILGFMVVTAFLSMWI-SNTA-STAMMVPIAHAVLQEL---NNTQSNVEEGSD
NPTFELQEPSPQK-ETSKVDEKDNGQAQ-PLPAVPLE-SGEHMTQE--QLRFSQGMSLCVCYSASIGGIATLTGTTPNLVLQG
QMTSLFPQNP-NVVNFASWFGFAF-PIMVILLLLSWLWLQILFL---

---GINFRKNFGIREQEHE
QQRKQAAYRVIQTQYRLLG-PMSFAEKAVFILFVILVLLWFTREPGFFHGWGNLVFSDASG------------RVMVSDGSASILIG
VFLFMVPSKIPGLTQDPDN----PGRLKAPPALLNW-KLVNK-KMPWNIVLLLGGGYALAKGSEESGLSQWLGNKLMPLQH
V-----PPPATVFIICLLVATFTECTSNAATTTLLLPILASMAQAICL-----------------HPLYVMLPCTLASSLAFMLPVATPPNAIV
FSFG------GLRVSDMARAGIMLNIIGVLVIMLAINSWGVPMFQLHTFPSWAHSNSTTHCLASPPTAPSP--------</seq-dat
a>
</sequence>
<sequence seq-type="Protein">
<seq-name>O35055</seq-name>
<seq-info><length>1375</length></seq-info>
<seq-data>---

---MATCWPALWAYRFYLIVLCLPIFLLPLPLIVQ-------TKEAYCAYSIILMALL
WCTE--ALPLAVTALFPIVLFPLMGIMD------------------ASEVCIEYFKDTNILFVGGLMVAIAVEHWNLHKRIALQVLLIIGVR
PALLLLGFMLVTAFLSMWI-SNTA-TTAMMVPIGHAVLEQL--QGSKKDVEGGNNNP
TFELQEECPQK-EVTKL---DNGQ---PV-SAPSE-PRTQKTQE--HHRFSQGLSLCICYSASIGGIATLTGTTPNLVLQGQVNSLF
PQNG-NVVNFASWFGFAF-PTMIILLLLAWLWLQVLFL---
---GVNFRKNFGFGEGEEERKQA
A--FQVIKTQYRLLG-PMSFAEKTVTVLFVLLVVLWFTREPGFFPGWGDTVFANEKG------------QSMASDGTVAIFISLVMF
IIPSKIPGLMQDPKK----PGKLKAPPAILTW-KTVND-KMPWNIVILLGGGFALAKGSEQSGLSEWLGDKLTPLQHI-----PPSA
TAVILCLLIAIFTECTSNVATTTLFLPILASMAQAICL-----------------HPLYVMLPCTLAASLAFMLPVATPPNAIVFSFG------G
LKVSDMARAGFLLNIIGVLAITLSINSWSIPIFKLDTFPSWAHSNTSQCLLNPSNSTVPGGL------</seq-data>
</sequence>
<sequence seq-type="Protein">
<seq-name>NDC2_RAT</seq-name>
<seq-info><length>1375</length></seq-info>
<seq-data>---

---MATCWPALWAYRFYLIVLCLPIFLLPLPLIVQ-------TKEAYCAYSIILMALL
WCTE--ALPLAVTVLFPIVLFLLMGIMD------------------ASEGLHRVLSRTPTYCLSGADGGHCCRTLEPAQTHCPPSASYHR
SAAALLLLGFMLVTAFLSMWI-SNTA-TTAMMVPIGHAVLEQL--QASKKDVEGGNN
NPTFELQEECPQK-EVTKL---DNGQ---PV-SAPSE-PRTQKTQE--HHRFSQGLSLCICYSASIGGIDNLTGTTPNLVLQGQVN
SLFPKMQCGELCFVVWFRLPH-HDHLAAAGLAMATGPLPGC--
---QLPEELWLWGRGRGT
KAGC---FPGHQDPVQAAGAHEFCREDLFTVLFVLLVVLWFTREPGFFPGWGDTVFANEKG------------QSMPSDGTVAIFI
SLVMFIIPSKIPGLMEDPKK----PGKLKAPPAILTW-KTVND-KMPWNIVILLGGGFALAKGSEQSGLSEWLGDKLTPLQHI---
--PPSATAVILCLLIAIFTECTSNVATTTLFLPILASMAQAICL-----------------HPLYVMLPCTLAASLAFMLPVATPPNAIVFSF
G------GLKVSDMARAGFLLNIIGVLATTLSINSWSIPIFKLDTFPSWAHSNTSQCLLNPSNSTVPGGL------</seq-data>
</sequence>
<sequence seq-type="Protein">

<seq-name>O57661</seq-name>
<seq-info><length>1375</length></seq-info>
<seq-data>--

---MVSIGKWILANRNYFIIFLVPLFLLPLPLVVP-------TKEASCGFVIIVMALF
WCTE--ALPLAVTALFPVLLFPMMGIMD-----------------STAVCSQYLKDTNMLFIGGLLVAISVEKWNLHKRIALRVLLIVG
VKPALLLLGFMVVTAFLSMWI-SNTA-TTAMMIPIAQAVMEQL------HSSEGKVDERVEGNSNTQKNVNGMENDMYESV
MPSGKMALAIDNTYATENEGFEIQEKSTKDPEPSK----QEKQSIGPIVIEPEDEKQTEEKQKEKHLKICKGMSLCVCYSASIGG
IATLTGTTPNLVMKGQMDELFPENN-NIINFASWFGFAF-PTMLVLLALSWLWLQFIYL-------------------------------------
----
GVNFKKNFGCGGNAEQKEKEKRAFRVISGEHKKLG-SMTFAEISVLVLFILLVLLWFTREPGFMPGWATISF-NKGG---------
---KEMVTDATVAIFVSLMMFFFPSELPSFKYQDTDKPGMKPKLRVPPALLDW-KTVNE-KMPWNIVILLGGGFALAKGSEE
SGLSLWLGEKLTPLQSI-----PPAAIALILCLLVATFTECTSNVATTTLFLPILASMAKAIQL----------------NPLYIMLPCTLSASL
AFMLPVATPPNAIAFSYG------QLKVIDMAKAGLLLNILGVLTITLAINSWGFYMFNLGTFPSWANATGQH-----------------
--</seq-data>
</sequence>
<sequence seq-type="Protein">
<seq-name>YV06_CAEEL</seq-name>
<seq-info><length>1375</length></seq-info>
<seq-data>--

--MARKRGISSLLLIYKQSFVIWGALLIFSPLLMFVG----DSHGLQAKCLYCVAV
MGSYWVFE--ALPLAITAFIPMILFPLFGIMR-----------------SEEVARAYLPDTCFLFMGGLMVALAVEKCELHARVALFVL
KTVGSEPARVMAGFMGVTGFLSMWI-SNTA-TTALMVPILQSVITELVSNHRMEDLVALCEAHHNSSRKHSVGMRRLSLP
NENNEIKREEMDTAMSPREQ---KMAKGLMLSVCFSANIGGAATITGTASNLV
LVGQLNELFPGAD-TGVNFLSWLIFAF-PMVFCCLIYCWCVLYLLYL---
---RDAPKG
SIIVTRKLQQKYNELH-AFSFAEMAVIFCFALLLVLWILREPQVVPGWGEMFKDELVFKSLTEKKNTHLTFRFVSDATSAMFI
VILLFTLPEKLPSSRGSS-------EQRKASSGLLDW-ATVQD-RFPWSVLFLLGGGFALAAGVKESGLSHDIGAIMRYLDVF-----
NHNIIMLICIIISVTLTNVCSNTVIASIFIPIVAELARSLEI----------------DPLNFMLPVTISASFAFLLPVATPPNAIVFSSG------
YLKVFDMFVSGLCVTLGCVVLSMLNMLLWAGFVFNLHLFPQWAANPSPPLDVQDWAVENNITFVGTSKL</seq-data>
</sequence>
<sequence seq-type="Protein">
<seq-name>YM22_CAEEL</seq-name>
<seq-info><length>1375</length></seq-info>
<seq-data>--

---MQIRHVRKMDFLEILAVAKNEKFWKIGSPPTIRKNP----ANWQLEWKCAYC
VCIIAVYWMSE--VMPLAVTAMLPVVLFPLVGVLD-----------------ANTTAKEYMNDTNFLFIGGLIMAAAVEKCDLHERV

ALSVLRCVGSEPKWIMLGFMTVTALLSSFI-SNTA-TTAMMVPIGQSVVQQLISSFQHHPTNGERGRLGCK----------------------
--KMATGLVLSICFAANIGGTGTATGTPSNLVMLGQLSALFPKV
D-GSLNYVTWIFFAY-PLMLLCLFVAWMTLVSFFL---
--RDAPEKDEAVTEMLKTR
YNELP-RMTYAEKSVFVCFCILLSLWVFRNPGVVPGFGVFFK-----------------KGAYTDATSAMIVAFLLFVLPSERPDLATYIK
K-----EDLKKRGCLMDW-KTMQE-TFPWSVVLLLGGGFALAAGVKESGLSLLIGNSLSSIEHL-----PLWILQLLTMLIAMVITN
ICSNTVTASIFVPIVATLAQRAGH-----------------HPFTLMLPTTLASSFAFIFPVGTPPNAIVFGSG------MVKVSDMAFVGG
IISLELLVLTVLYMNSIAYLTLPLLEFPTWAIIANSTMQQ-----------------</seq-data>
</sequence>
<sequence seq-type="Protein">
<seq-name>YKG6_CAEEL</seq-name>
<seq-info><length>1375</length></seq-info>
<seq-data>---

--MPFESTEGKCAFVILTMSCYWVAE--VVPL
AVTSFIPMIALPFLGIVS------------------IKEVAPKYFADTNIVFFNSLMLSLAVEECQLHKRIALKMLTYVGTRPHWLMAGF
MIITSFISLWI-SDTA-CCALMAPIAYALLEEIMIPKMRPEEKENEIEVMKIFDKEDPEEKEKKKLDTSRLSVRDR-----------------
---GICKCMMLLVAHASLIGGTGTINSTGPNLIFRDNIEKNFPNEDHGIS-YLSWMAFAI
-PPMIFYMFSSWFIVQLQFL---
--GPRHLMGMFREPTETEKQEEEVAKRAVWKSYDQLG-P
MTWAEKSTLVIFVLAVLSWVSSDPKVIPGWSDLFR-----------------KGYVTDSCSGLVAVFLLFIWPKKKPDFRIFRKD---KSR
PSVRQEPLIDW-DCVRR-RFPWSIILLLGAGFAISDAVRVSGLSSLIACSLNSTISK----MPFFVMQIILSIVVVVMTEFSTNSAT
ASIFIPISFKMAEAVGA-----------------HPLYFSIPTAIGPSFSFMLPMATPANAIVYETK------TIRMIDMVSCGVFLNIFCIAIT
AINMNTWAFWLFNMGTYPDYALRHATNMTGNSSQCF-----------</seq-data>
</sequence>
<sequence seq-type="Protein">
<seq-name>YNH1_CAEEL</seq-name>
<seq-info><length>1375</length></seq-info>
<seq-data>---

--MKPSPQRTLIKKLLVLLGPLVAVPLLFFGP---------EYRCLFSIIFLSTYWI
GE--AFPIGVTSLFPLALYPILQIVP------------------SKQISPVYFKDSIVLFMCTLIMAMAVEATGLHRRIALKLLTKVGAKQP--
----------VSFFV-SDTA-CTALMCPTAVALLMSMSDAVQHLKEDHRKPKP-PPDDATVAEKLRIDDMTPQDA-----------------
---GFCKALILACAHASLIGGTAIITSTGPNLVFRENIHKRYPEGQ-VTMTYLQWM
VFAI-PPMFVYLLASYIILVCYFM---
---GPSTFARWFERPSKEEAHLKKLIEKNIQTMYEDLG-DV
SWGEKSVFVFFILLIGSWISRDPGFTPGWGDLLPH----------------RNFISDSVSGVLISCILFVWPKDPFDPIDP-------------M
APILKW-TDMKS-KFSWSCTLLIGAGYAISEGVDKSGLSRLISCGMKNIFVG----MSSLPLQLTVTTIIVIMTEFASNVSTGSIFI
PISLGVAESMGV-----------------HPLYLALPTTVACSFAFMLPISTPPNAVVYDTK------VISMVEMIVCGFLLNIACILITSLN
MNTWTYFIFSLNIFPENIVISSENSSYPVC--------------</seq-data>

</sequence>
<sequence seq-type="Protein">
<seq-name>O25003</seq-name>
<seq-info><length>1375</length></seq-info>
<seq-data>--

--------------------------------MENHSHANTHTDTRTDDKSTKIVRLLGLIGGALIALVIYYALNSQMPHIVEEIPKLSSLNYKA
MPVVAGVAVLMGIWWMTE--AIDLPATALLPLVLFSVFSVDQ------------------FASVSSSYASPIIFLFMGGFILALSMQKW
NLHTRIALSIILLVGTSPRRLILGFMMATGFLSMWV-SNTA-TAVMMLPVGMSVLQLVAKLVGKEDASNSWHQKEEITKA
HGGIMSNIVHKGKDITQVIQEKTTIYRT--NFSICLMLGIAYAASIGSLGTLIGT
PPNALLAGYMKTAFNIE----IDFAQWMVFGT-PLAFIMLILAWLLLTYVIF---
--PL
KIKEIPGGKEVIRVELKKLG-RLSQAEISVGIIFILASLGWIFL-GVMLKSWGV--------------------KIDKIDSVIAMGVSALLFILP
ANHQ-----------------GDRLIDW--GVAK-KLPWDVLLLFGGGLALSAQFSKTGLSLWIGHLVSGFSHL-----PILFIIVMVTLM
VIFLTEITSNTATAAAFLPVIGGVAMGMGYENH--------------QSLLLTIPVALSATCAFMLPVVTPPNAIAYGSG------YVKIT
DMIKAGLWLNLVGVVLISTFSYFLVSLIFN---------------------------------</seq-data>
</sequence>
<sequence seq-type="Protein">
<seq-name>Y608_HAEIN</seq-name>
<seq-info><length>1375</length></seq-info>
<seq-data>--

---MGEMASLRSHKNGIIFILDIVLFFVLLNVLP-----FEPKANSGLALLAFIAV
LWLSE--ALHVTITALLVPLLAVALGLVS------------------TKQALVGFADPTIFLFFGGFSLATALHIQKLDKLIANKIMALARGN
LFIAVIYLFLITAFLSMWM-SNTA-TAAMMLPLAMGILSQLDREKDH--
-------------------------------------NTYVFVLLGIAYSASIGGMGTLVGSPPNAIVASNLN----------LTFSDWLWYGL-PIMIILLP
LMIGILYIIFK--
--PKLHLNFEQTFENIEMNPMRILT-FIIFPVIALTWIFSGKINPFIS-
-GLLGLQK---------------------NIASFDSIVALLAAIVICST------------------------GVASW-KQIQS-NTDWGVLMLFGGGLTL
SAVLKDSGASKILADSIVFMIDG----QHFYLIGLLVAAFIIFLTEFTSNTASAALLVPIFISIAQSLGM-----------------PEIGLALIIG
IGASCAFMLPVATPPNAIVFGSG------QVKQSEMVKVGFLLNLVCVVVIATMGYMFWLK------------------------------------
</seq-data>
</sequence>
<sequence seq-type="Protein">
<seq-name>O66449</seq-name>
<seq-info><length>1375</length></seq-info>
<seq-data>--

---MNKNVFLVLAILVFFGVLITSP-----FEGNVNKGIAILLLAAILWIT

E--ALPLPVTALLIPVSGVLLGVFD------------------VKTALSYFAHPLIFLFFGGFVLAVALSKYQIDEYIAHKIVSVAQGKFLPSV
FLLMLATSLISMWI-SNTS-TTAMMLPLALGILAGVRETERE--
----------------------------KVFPFVLLGIAYSASVGGIGTLVGSPPNGIAAGILG----------LSFPDWLKFGI-PVFLILFPLLFAILFLV
FR---
--PTSDLKVERVQEIKFEFTPQRVLVLLIFLFTALAWIFSKKIAPIFE------
----------------VKKYFDTVVALLAVVALFIF-------------------------RLLDW-RDVKE-GVSWGTLLLFGGGIALSGIMKKTGTAK
FISQELVDVLHG----LPTFLFLLTIVLFVIFLTELMSNTATTALIAPILFSTAQMIGK-----------------PPEMLVIPAAVAASCAFM
LPVATPPNAIVYGTG------YIKQSQMMRVGLILNIVFSIVLAAFFTVYVGG-------------------------------------</seq-data>
</sequence>
<sequence seq-type="Protein">
<seq-name>Y672_METJA</seq-name>
<seq-info><length>1375</length></seq-info>
<seq-data>---

---MRLSKEFIGLGIITASLIFGSSLP--------DIYKGIVILIVAGCLWFFE--
LLPLPVTSLAIPIMAVFLGIFN------------------LKEALTYFAHPIIFLFLGGFMLAQALKNHNLDKFIAYKLL-NYGKDFKTTCFL
MFLSAYFLSMWI-SNTS-ATLILLPIALGLLHKKTG---
-------------------KLRDFLLLGVAYSASIGGIATIIGSPPNAIASSYLD----------YGFFSWFKVGF-PISLLLFLICTLTLYIYFK--------
----
--KWIPKEDIAIQARMELSRNAYKLLVIFVLIASLWIISDYLSEIF-------------------
----NVQYFDSVIAIFAIILLFVF-------------------------NLVEV-NDFK--KIDWGTLILFGGALCLGGVIVKSGANTFLSEKLIAILG
N----LTPIVLLFLVVTITIILTNFISNTGLTGIIVPILFGVS--LGI-----------------PKEILILAVGMSASCSFILPVGTPPNAIVYSEG--
----VKKE-EMMKIGMILSILSAAVITLYSILYL--</seq-data>
</sequence>
<sequence seq-type="Protein">
<seq-name>ARSB_STAXY</seq-name>
<seq-info><length>1375</length></seq-info>
<seq-data>---

---MTILAIVIFLLTLIFVIWQPKGLDIGITA
LIGAVVAIITGVVSFS----------------DVLEVTGIVWNATLTFVAVILISLILDEIGFFEWSAIHMVKASNGNGLKMFVFIMLLGA
IVAAFF-ANDG-AALILTPIVLAMVRNLGFNKK--
--------------VIFPFIIASGFIADTTSLPLIVSNLVNIVSADYF----------DIGFIEYFSRMIIPNIF-SLIASILVLWLYFRK---------------
----
--SIPKTFNTENLSDPKNVIKDPKLFKLSWIVLAILLVGYLVSEFIQ----------------------------
--IPVSIIAGIIALIFVILA--------------------RKSKAVHT-KQVIK-GAPWNIVVFSIGMYLVVFGLKNVGITTILGDILTNISSYG----
-LFSSIMGMGFIAAFLSSIMNNMPTVLIDAIAIGQSSATGI------------------LKEGMVYANVIGSDLGPKITPIGSLATLLWLHV
LTQKGVK-ISWGTYFKTGIIITIPVLFVTLLGLYLTLIIF------------------------------------</seq-data>
</sequence>
<sequence seq-type="Protein">

<seq-name>P96678</seq-name>
<seq-info><length>1375</length></seq-info>
<seq-data>--

---MTSMILAVFIFLLTLVLVIWQPKNLSIG
WSACGGAVLALIAGVVNFH----------------DVLTVTGIVWNATLAFVAIILISLILDNIGFFEWAALHMAKAAKGYGVRMFVY
VSLLGAIVAALF-ANDG-AALILTPIVLAMVRALHFNEK---
-------------------------LVFPFIIASGFIADTTSLPFVVSNLVNIVSADYF----------HITFIDYASRMVVPYLF-SLLASIIVLYLFFRK--
----
--SIPKRYDLTEVKKPVEAIKDQNMFRLSWYILGLLLIGYFASEFFS--------------
----------------IPVSVVAGSIAIIFLIAA--------------------QKSPAVHT-KKVVK-EAPWAIVFFSIGMYVVVYGVRNAGLTDVLS
DVIQAAADQG-----LFAGTIGMGFIAAILSSIMNNLPTVMIDALAIAGTDTHGM-----------------MREALIYANVIGSDLGPKI
TPIGSLATLLWLHVLSHKGMK-ISWGTYFKTGIILTIPTLLITLVGLYIWLLIIHSCF---------------------------------</seq-data>
</sequence>
<sequence seq-type="Protein">
<seq-name>ARB2_ECOLI</seq-name>
<seq-info><length>1375</length></seq-info>
<seq-data>--

--MLLAGAIFVLTIVLVIWQPKGLGIGW
SATLGAVLALISGVVHIG----------------DIPVVWNIVWNATATFIAVIIISLLLDESGFFEWAALHVSRWGNGRGRLLFTYIVLL
GAAVAALF-ANDG-AALILTPIVIAMLLALGFSKG--
-----------------TTLAFVMAAGFIADTASLPLIVSNLVNIVSADFF----------GLGFTEYASVMV-PVDIAAIIATLVMLHLFFRK---
----
---DIPPTYDLALLKAPAKAIKDLATFRTGWIVLILLLVGFFVLEPLG----------------
--------------IPVSAIAAVGAVILFAVA--------------------NRGHAINT-GKVLR-GAPWQIVIFSLGMYLVVYGLRNAGLTEYLS
GVLNVLADKG-----LWAATFGTGFLTAFLSSIMNNMPTVLVGALSIDGSTATGV----------------IKEAMIFANVIGCDLGPKI
TPIGSLATLLWLHVLSQKNMT-ITWGYYFRTGIVMTLPVLFVTLAALALRLSFTL-----------------------------------</seq-data
>
</sequence>
<sequence seq-type="Protein">
<seq-name>ARSB_ECOLI</seq-name>
<seq-info><length>1375</length></seq-info>
<seq-data>--

--MLLAGAIFVLTIVLVIWQPKGLGIGW
SATLGAVLALVTGVVHPG----------------DIPVVWNIVWNATAAFIAVIIISLLLDESGFFEWAALHVSRWGNGRGRLLFTWI
VLLGAAVAALF-ANDG-AALILTPIVIAMLLALGFSKG---
----------------------TTLAFVMAAGFIADTASLPLIVSNLVNIVSADFF----------GLGFREYASVMV-PVDIAAIVATLVMLHLYF

RK---
--DIPQNYDMALLKSPAEAIKDPATFKTGWVVLLLLLVGFFVLEPLG-----
-------------------------IPVSAIAAVGALILFVVA--------------------KRGHAINT-GKVLR-GAPWQIVIFSLGMYLVVYGLRNAG
LTEYLSGVLNVLADNG-----LWAATLGTGFLTAFLSSIMNNMPTVLVGALSIDGSTASGV-----------------IKEAMVYANVIG
CDLGPKITPIGSLATLLWLHVLSQKNMT-ISWGYYFRTGIIMTLPVLFVTLAALALRLSFTL----------------------------------</s
eq-data>
</sequence>
<sequence seq-type="Protein">
<seq-name>ARB1_ECOLI</seq-name>
<seq-info><length>1375</length></seq-info>
<seq-data>--

---MLLAGAIFILTIVLVIWQPKGLGIGW
SATLGAVLALASGVIHIA----------------DIPVVWNIVWNATATFIAVIIISLLLDESGFFEWAALHVSRWGNGRGRLLFTYIVLL
GAAVAALF-ANDG-AALILTPIVIAMLLALGFSKS---
-----------------TTLAFVMAAGFISDTASLPLIVSNLVNIVSADFF----------KLGFTEYASVMV-PVDIAAIIATLVMLHLFFRK----
----
---DIPPTYELARLKEPAKAIKDPATFRTGWVVLLLLLVGFFVLEPMG--------------
----------------IPVSAIAAVGAAVLFAVA--------------------KKGHGINT-GKVLR-GAPWQIVIFSLGMYLVIYGLRNAGLTDYL
SDVLNELADKG-----LWAATLGTGFLTALLSSIMNNMPTVLIGALSIDGSTATGV-----------------IKEAMIYANVIGCDLGPKI
TPIGSLATLLWLHVLSQKNMT-ITWGYYFRTGIVMTLPVLFVTLAALALRLSVTL----------------------------------</seq-data
>
</sequence>
<sequence seq-type="Protein">
<seq-name>ARSB_YEREN</seq-name>
<seq-info><length>1375</length></seq-info>
<seq-data>--

---MLLAGSIFVLTLILAIWQPRGLSIGWS
ASICAALALVSGVIHVG----------------DIPVVWNIVWNATATFIAVIIISLLLDESGFFEWAALHVSRWGNGRGRLLFTWIVLL
GAAVAALF-ANDG-AALILTPIVIAMLLALGFSKR---
-----------------TTLAFVMAAGFIADTASLPLIVSNLVNIVSADFF----------RLGFTEYASVML-PVDIAAIAATLGMLHLFFRR--
----
---DIPATYDVSLLKMPASAIKDPATFRAGWIVLVLLLVGFFVLEPLG------------
------------------IPVSAIAAVGTVVLFAVA--------------------KKGHAINT-GKVLR-GAPWQIVVFSLGMYLVVYGLRNAGLTE
YLSGVLNMLADKG-----LLAATFGTGFLTAFLSSVMNNMPTVLVGALSIDGSTASGV-----------------IKEAMIYANVIGCDL
GPKITPIGSLATLLWLHVLSQKNMT-ISWGYYFRTGIIMTLPVLFVTLAALALRLSVTL----------------------------------</seq-
data>
</sequence>
<sequence seq-type="Protein">

<seq-name>Q9YG46</seq-name>
<seq-info><length>1375</length></seq-info>
<seq-data>---

--MDLVAALLFTLTIMLVVSR--YVDEVA
AALAGVTALILLTNYT------------------PEEAFNFVEWNVIAILLGMWIIAGYMIEGGFTE-ATIRLVSRSAGTYRKFLLYMALLS
GFISMFI-DNVL-VILLVGSLTIAAAKKAGG--
------------NPVLAILLVGFSANFMGTALLMGDLPPQLLHTIA----------GAEFLDFIWTRGKPSSFPLLTATFLLTLGVFYI--------
----
---LFIRREPNSRIDDAGLSGESTNRGGLLIISLTFFTMTVIAMALRP----------------------
-------LLGFPLGFITMAGASQLALTVEVLRRLGLA--------------GMVEF-ENALK-HVEWRALLFYAALFSLVGGLESEGVIEEIA
RLLVGSVSSD-----PAIAYTVMYWSVGLLSLVIEHDALLLTFLYIVKDAASLAGI----------------DPWNIYWGMAWSATLASN
ATTAAAPALYVAVAMSDREGHR-VRALEFLKYSLTFAFTSLIIHYIITLIVWI--------------------------------------</seq-data>
</sequence>
<sequence seq-type="Protein">
<seq-name>YQ84_MYCTU</seq-name>
<seq-info><length>1375</length></seq-info>
<seq-data>---

--MSVVAVTIFVAAYVLIASD--RVNKTM
VALTGAAAVVVLPVITSHD--------------IFYSHDTGIDWDVIFLLVGMMIIVGVLRQTGVFEYTAIWAAKRARGSPLRIMILL
VLVSALASALL-DNVT-TVLLIAPVTLLVCDRLNI---
------------------NTTSFLMAEVFASNIGGAATLVGDPPNIIVASRA----------GLTFNDFMLHLT-PLVVIVLIALIAVLPRLFG----
----
---SITVEADRIADVMALDEGEAIRDRGLLVKCGAVLVLVFAAFVAHP--------------
----------------VLHIQPSLVALLGAGMLIVVS-----------------------GLTR-SEYLS-SVEWDTLLFFAGLFIMVGALVKTGVVND
LARAATQLTGGN-----IVATAFLILGVSAPISGIIDNIPYVATMTPLVAELVAVMGGQP--------------STDTPWWALALGADF
GGNLTAIGASANVVMLGIARRAGAP-ISFWEFTRKGAVVTAVSIALAAIYLWLRYFVLLH----------------------------------</s
eq-data>
</sequence>
<sequence seq-type="Protein">
<seq-name>YQ85_MYCTU</seq-name>
<seq-info><length>1375</length></seq-info>
<seq-data>---

--MSIIAITVFVAGYALIASD--RVSKTRVA
LTCAAIMVGAGIVGSDD--------------VFYSHEAGIDWDVIFLLLGMMIIVSVLRHTGVFEYVAIWAVKRANAAPLRIMILLVL
VTALGSALL-DNVT-TVLLIAPVTLLVCDRLGV--
----------------NSTPFLVAEVFASNVGGAATLVGDPPNIIIASRA----------GLTFNDFLIHMA-PAVLVVMIALIGLLPWLLG----

----
--SVTAEPDRVADVLSLNEREAIHDRGLLIKCGVVLVLVFAAFIAHP---------------
-------------VLHIQPSLVALLGAGVLVRFS-----------------------GLER-SDYLS-SVEWDTLLFFAGLFVMVGALVKTGVVEQL
ARAATELTGGN-----ELLTVGLILGISAPVSGIIDNIPYVATMTPIVTELVAAMPGHV--------------HPDTFWWALALSADFGG
NLTAVAASANVVMLGIARRSGTP-ISFWKFTRKGAVVTAVSLVLSAVYLWLRYFVFG----------------------------------</seq
-data>
</sequence>
<sequence seq-type="Protein">
<seq-name>AG45_MYCLE</seq-name>
<seq-info><length>1375</length></seq-info>
<seq-data>---

--MSVIAITVFIVTYALIASD--RVNKTLAA
LTGAAIVVTLPIINSED--------------VFYSHETGIDWEVIFLLLSMMIIVSVLRQTGVFEYVAIWTAKRSHGSPLRILLLLVLVMA
LGSALL-DNVT-TVLLIAPVTLLVCERLTI--
---------NAAPFLMAEVFASNIGGARTLVGDPPNIIIASRA----------GFSFNDFLIHLT-PIVIIVTVVLSALLPRLFRG-------------
----
-------------------------------------AFAVDPERVADIMSLNEREAIRDRWLLIKCGVVLLLVFVAFIAHP--------------------------
----VLHTGPSLVGMLGAGILIVIS-----------------------KLER-SDYLS-SVKWETLLFFAGLFIMVGALVKTDVVNQLARATTTL
TGGH-----ELLTVVLTLGVSTLVSSIIDNIPYVATMTPIVSELVASMPDQS-------------HTDILWWALALGADFGGNLTAVGA
SANVVMLEIAKSAGTP-ISFWEFTRKGIAVTVISIALAGIYLWLRYLVMS----------------------------------</seq-data>
</sequence>
<sequence seq-type="Protein">
<seq-name>P_HUMAN</seq-name>
<seq-info><length>1375</length></seq-info>
<seq-data>---
-----------------------------------MHLEGRDGRRYPGAPAVELLQTSVPS------GLAELVAGKRRLPRGAGGADPSHSCPRGA
AGQSSWAPAGQEFASFLTKGRSHSSLPQMSSSRSKDSCFTENTPLLRNSLQEKGSRCIPVYHPEFITAEESWEDSSADWER
RYLLSREVSGLSASASSEKGDLLDSPHIRLRLSKLRRCVQWLKVMGLFAFVVLCSILFSLYPDQGKLWQ-LLALSPLENYSVNL
SSHVDSTLLQVDLAGALVASGPSRPGREEHIVVELTQDDALGSRWRRPQQVTHNWTVYLNPRRSEHSVMSRTFEVLTRET
VSIS-IRASLQQTQAVPLLMAHQYLRGSVE-------TQVTIATAILAGVYALIIFE--IVHRTLAAMLGSLAALAALAVIGDR--------
-----PSLTHVVEWIDFETLALLFGMMILVAIFSETGFFDYCAVKAYRLSRGRVWAMIIMLCLIAAVLSAFL-DNVT-TMLLFTPV
TIRLCEVLNL--DPRQVLIAEVIFT
NIGGAATAIGDPPNVIIVSNQELRKM-----GLDFAGFTAHMF-IGICLVLLVCFPLLRLLYWN------------------------------------
---RKLYNKEPSEIVELKHEIHVWRLTAQRI
SPASREETAVRRLLLGKVLALEHLLARRLHTFHRQISQEDKNWETNIQELQKKHRISDGILLAKCLTVLGFVIFMFFLNSFVP--
------------------------GIHLDLGWIAILGAIWLLILA----------------------DIHDF-EIILH-RVEWATLLFFAALFVLMEALAHLH
LIEYVGEQTALLIKMVPEEQRLIAAIVLVVWVSALASSLIDNIPFTATMIPVLLNLSHDPEVGL--------------PAPPLMYALAFG
ACLGGNGTLIGASANVVCAGIAEQHGYG-FSFMEFFRLGFPMMVVSCTVGMCYLLVAHVVVGWN------------------------
--------</seq-data>
</sequence>

<sequence seq-type="Protein">
<seq-name>Q62052</seq-name>
<seq-info><length>1375</length></seq-info>
<seq-data>--
---------------------------------------MRLENKDIRLASAVLEVELHQTSALSVPTCPDPGRLLTVKPATSNYKLGQADPCIPYAGEA
AGKSVCVPEHTEFGSFLVKG----------SSSLKDLSFKEDTPLLWNSSQKKRSQLMPVHHPEFIATEGSWENGLTAWEQKCM
LGKEVADLSALASSEKRDLAGSVHLRAQVSKLGCCVRWIKITGLFVFVVLCSILFSLYPDQGKFWQ-LLAVSPLENYSVNLSG
HADSMILQLDLAGALMAGGPSGSGKEEHVVVVVTQTDAAGNRRRRPQQLTYNWTVLLNPR-SEHVVVSRTFEIVSREAV
SIS-IQASLQQTRLVPLLLAHQFLGASVE-------AQVASAVAILAGVYTLIIFE--IVHRTLAAMLGALAALAALAVVGDR---------
----PSLTHVVEWIDFETLALLFGMMILVAVFSETGFFDYCAVKAYQLSRGRVWAMIFMLCLMAAILSAFL-DNVT-TMLLFT
PVTIRLCEVLNL---DPRQVLIAEVI
FTNIGGAATAIGDPPNVIIVSNQELRKM-----GLDFAGFTAHMF-LGICLVLLVSFPLLRLLYWN------------------------------------
--KKLYNKEPSEIVELKHEIHVWRLTA
QRISPASREETAVRGLLLEKVLALEHLLAQRLHTFHRQISQEDKNWETNIQELQRKHRISDRSLLVKCLTVLGFVISMFFLNSF
VP------------------------------GIHLDLGWIAILGAIWLLILA------------------------DIHDF-EIILH-RVEWATLLFFAALFVLMEALT
HLHLVEYVGEQTALLIKMVPEDQRFAAAIVLIVWVSALASSLIDNIPFTATMIPVLLNLSQDPEISL--------------PALPLMYAL
ALGACLGGNGTLIGASTNVVCAGIAEKHGYG-FSFMEFFRLGFPVMLMSCTIGMCYLLIAHIVVGWN--------------------------
---------</seq-data>
</sequence>
<sequence seq-type="Protein">
<seq-name>Q9Y163</seq-name>
<seq-info><length>1375</length></seq-info>
<seq-data>--
---------------------------------------MAYNPNSSAEDNAYEDDEVTEGALQVWRTLPERIRQDPSLASFRQEHERLHGDVDQLH
AEESELRVQNGHHEETALANQPFTQIGINVTNEAGQVRVLDGRDLENNNDDQHDEAEPHAKSLIRKYLIWFKIAILLVVWC
VFTAFLMSNNEHVDQLSLISVPRNSSPRVFPIATASLQRIGLGLRGPFRLQENELAINASVPLPLLQVQVMRSYFDGEGMEI
YIENASQVWQLDVVY--------------------PDLIDATRDTKKQRTFELSPTDPLWLAQANRT-------ELSFEFTSSIEGELP-LQLN
VDESPIYKRDG-------------------VIYAAAVLCGLYVMIIWE--IVNRTFAAIIASTLSVGILAALNSR-------------PSMATIMGWI
DVETLLLLFGMMILVAILSETGVFDYLAVYAYKITNGHVWPLINCLCLFTAVLSSFL-DNVT-TVLLMTPVTIRLCEVMCL-------
---NPVPILMCMVIYSNIGGALTPVGDP
PNVIIASNSYISKN-----GVNFAVFTLHML-PGVLLVMVQTYIQLRFKFRN--
--ISDLQFKDSPEVEELRHEIHVWKRAAASLSAYSKDEELVR
QTLMKKVNRL----KRSLKKRMTAVIEPAPNYQQTLANLQAKYPIRNKPLLIKCSAALVFVISLFFLHSVPE-----------------------
----LQRLSLGWTALLGAIFLIILA-----------------------DIEDM-EAILA-RVEWSTLLFFAALFILMEALTELGLIEWIGNMTEGIIL
GVGEDRRLMVAILIILWVSAVASAFVDNIPLTTMMVKITISLAQNSTLNL--------------PLQPLVWALALGACLGGNGTLIGA
SANVVCAGVAEQHGYK-FTFLQFFKVGFPIMIGSIIVTTGYLLVSHSLFAWH--------------------------------</seq-data>
</sequence>
<sequence seq-type="Protein">
<seq-name>O59575</seq-name>
<seq-info><length>1375</length></seq-info>
<seq-data>--

--MISEVEIITLGIFIATYAVIISE--KIHRTVAA
LLGASIMLVLNIVP------------------WEKVPEYLDLDTILLLAGMMVVVNISKESGLFEYIAIKVAKVSKGNPIRVLLLFSVTTAV
VSAFL-DNVT-TVLLLTPMLLYITRRMGV--
----------NPVPYLLAEIFASNIGGTATLIGDPPNIMIGSAA----------KLSFNDFIVNMA-PISFMDLFIMIALVYILYKK------------
----
---EFSRVKYNLDGIMNLREEDAIKDRELFKKSVITIGLIIVAFFFHDALG----------------------
-------IE-PAVVALTGATILLLWS------------------------RVSP-EVALE-KVEWATLFFFGGLFIIVGGLVETGLIDEAGKWIVRHIT
S------ENEAILLISWISAFSSAIIDNIPFTATMIPLIKSMAGSI------------------NIYPLWWALSLGACLGGNGTAIGASANVVVL
GIAYREGIR-ITFKDFLKVGMAVMISSVAAGSLVLIARYGGGI-----------------------------------</seq-data>
</sequence>
<sequence seq-type="Protein">
<seq-name>Q9UY19</seq-name>
<seq-info><length>1375</length></seq-info>
<seq-data>---

--MVSELEIIALGVFISVYAAIISE--KIHRTVA
AMLGASIMLFLKIVP------------------WENIPEYLDLDTILLLAGMMVIVNISRESGLFEYIAIKVARISKGDPMRVLLLFSVTTA
VVSAFL-DNVT-TVLLLTPMLLYITRRMGV--
------------DPVPYLLAEIFASNIGGTATLIGDPPNIMIASAA----------KLSFNEFIMNMT-PIAFLDLFIMVALIYLFYRR----------
----
---EFKNVEYHVDGIMNLREEEAIRDKQLFRKSVVTIAFVVLAFFLHDTIG------------------
-----------IE-PAVVALTGASILLLWS------------------------RVPP-EVALE-KVEWATLFFFGGLFIIVGGLVETGLIEDVGKWIVS
HISS------ENEAILMISWVSAFLSAIIDNIPFTATMIPLIKSMGGSI------------------NVYPLWWALSLGACLGGNGTAIGASA
NVVVLGIAYREGIR-ITFIDFLKVGMIIMLLTVGAGSLVLIARYGGGIL----------------------------------</seq-data>
</sequence>
<sequence seq-type="Protein">
<seq-name>Q9X032</seq-name>
<seq-info><length>1375</length></seq-info>
<seq-data>---

---MFEDAVVSLLIFIIVYLFIILE--KHHRAVIT
MLGGSAALFLVFKDP-----------------IEALVRYVDFNTIFLLIGMMIFVSVTKRSGLFHFLGLYSVKLSGGNVFLFFIAINTLVAL
LSSFL-DNVT-TILVFVPVTLVVCDTVDL---
---------DPVPFVISEIISSNIGGTATMIGDPPNIMIASAA----------KLHFLDFVVNVA-PAAVLTLIVTLIFLSAVYRR-------------
----
---VIFRKIPVEVVKDFDPRRAIVDKKLFYLSIILTLVVLVLFSLQKPLG--------------------------
---LESFEVALLAGFLSLAFLK------------------------------KKDI-EDVFK-EIEWGVIFFFIGLFLVVGALEEAGVLERISELVIRLSKGK----
-MESTLISVLGISGLSSAFVDNIPFTATMIPVIKKLAVLAPETFS-------------DLRPLWWALSLGACFGGNGTLIGASANVVGT
SLI-ADRKH-ITFWEYFKIGFPVLILSLLVSGVYLLIRY---------------------------------------</seq-data>

</sequence>
<sequence seq-type="Protein">
<seq-name>Q9X0D5</seq-name>
<seq-info><length>1375</length></seq-info>
<seq-data>--

---MNEAMLVLLFAILAYYFIIF-GKIAKSVV
TLLIALVLMAIKVVEGLD---------------LKNIGEVVDFNTLGLLLGMMIIVHILKGTGFFEYLAISAIKISRGRFWLLFAFLMVLT
AVTSAFL-DNLI-TIILVSPILFLILDTMEV---
---------NPVPFFLFTIFIDNIGGMSTLIGSPLNIVLGSIS----------GLSFNDFLKNMG-LVTVLMFVVVFFLFKRYVRI-------------
----
--DEKAFEKLRNLLSVDPKRSITDPVVLKKSLFVFLSTLVLFGLHSFVE------------------------
-----VE-LSLIALIAACALLLML------------------------GKNF-EKVSE-GIDWDTLFFYTGLFIISYSLEQIGVMEVIASFLKALSFN---
---RFLFVATVTWISILSTAFLSAVPATLIIAPTLKILVSQG------------------FPASLWWVYAVGANLGTNLTPLGAVQNIVGLSL
LEKYTKHTVSFKEFFKVAWSFMFIPFIIATLYSLIIY---------------------------------------</seq-data>
</sequence>
<sequence seq-type="Protein">
<seq-name>O27976</seq-name>
<seq-info><length>1375</length></seq-info>
<seq-data>--

--MIELLISILTYALISLQSFYR--KMDRPAAS
TIGAALMLAFGVMS------------------LKEAVRAIDYNTIMLLFGMMVLTAYLGIAGFFDWLASKIMGFS-GNGKRLLFTIVFSS
GFLSALF-VNDT-ICVFMTPVVVRVALSAGI--
--------------SPIPLLIALATSANVGSAVTIIGNPQNMLIGLKS----------GIGFVQFTAKML-PVGVAGIFIVYSVIYVLYRG-------
----
--ELGKLRNGKIEVNLNRRLAAKGLLTFALVLILFVTEFY-------------------------
-------PVSLSAFIGAAIMFAIG-----------------------GVK-P-REALE-KVDWGLLLLFCNLFIVMHGFEKS-YGEKILSMLSFGE---
-----SFADYLYFSLLTVAGSNLVSNVPFVMMVLPAAKGAAAD-----------------------FWLILAMASTFAGNLTLIGSVANLIVA
ESAERHGVH-LSFFEYLKAGVPVTFATVLVGALLIYIT--</seq-data>
</sequence>
<sequence seq-type="Protein">
<seq-name>Y753_SYNY3</seq-name>
<seq-info><length>1375</length></seq-info>
<seq-data>--

---MVANLPALFSLGVFVGVILLIMSE--KIHLTI
AAFLGALILVFTHVITLK----------------EGIDYISQSYATLALFFGVMVLVRSFEPTKIFEYLATQMVLLAKGSGKLLMLGVIAIT
TPICAVL-PNAT-TVMLLAPLIPPLAQEIGV--

------------DFVPILILMVFVANSAGLLTLVGDPATFIVGDAI----------NISFNDYLFKLS-FMGVLAIVSIVVITPFLFRH-----------
----
--IWRSRFTHLEDLPHPQINHPKVLMAGGVIITLVLIFFVIGESLP-----------------------
-----VPIPPASVALMGACLALLLA--------------------SQSKIDTV-HNILR-DVDWSTLIFFMSIFVIIGSLEKTGVTASLAQLLAVV
VGQN-----IAFGAIVLVFTVGLLSSVVPNIPLVVAMVPLLKQYVVNIGFAGPEILGANFDGQLPAEVLPLFYAMMFGATLGG
NGTLVGASSNIVAAGISEQHGRP-ISFHRFLRYGLPVMAVQLVVAALFVAWLMFTQQG----------------------------------</s
eq-data>
</sequence>
<sequence seq-type="Protein">
<seq-name>YOJA_BACSU</seq-name>
<seq-info><length>1375</length></seq-info>
<seq-data>--

--MPILIVAVGVLILLFLIIK-VKLNTFVSLI
VVSFLVAIGLGMDIN-----------KIVLSIETGIGGQLGHLALVFGLGAMLGRLVSDAGGGYRIAITLIDKFGRKRIQAAVVVASFI
IGIALFF-EV---GLVLLIPIVYAIAKEL--
------KMPFLYLGIPMAAALNVTHGFL--PPHPAPTAISVAY-------GAHIGQVLLFGI-IIAVPTTVIAGPLFNKFAMKRFPGAY
QKHG--
--NLSGLGPRKEFQLDETP-GFAISAVTSLFPVIFMAMATIFSLLLSEHSKGKDII
-----------------EFIGTPGTAMLISLLLALYTMGYARKISMQ---------------EIGRS-ISESI-SQIAMMLLIIGGGGAFKQVLIDGG
VGDYVADFFRQT--------NMSPLFVAWTIAAVLRLCLGSATVAALTTAGMAAPLMEAGSV-----------------NPALMVLATG
AGSVIACHVNDAGFWMVKEFFGLSM----KETFQTWTLLTTVLSVTGLGCVLLAGLVM--
</seq-data>
</sequence>
<sequence seq-type="Protein">
<seq-name>DSDX_ECOLI</seq-name>
<seq-info><length>1375</length></seq-info>
<seq-data>--

---MHSQIWVVSTLLISIVLIVLTIVK-FKFHPFL
ALLLASFFVGTMMGMGPL-----------DMVNAIESGIGGTLGFLAAVIGLGTILGKMMEVSGAAERIGLTLQRCR-WLSVDVI
MVLVGLICGITLFV-EV---GVVLLIPLAFSIAKKT--
--------------------NTSLLKLAIPLCTALMAVHCVV--PPHPAALYVANKL-------GADIGSVIVYGL-LVGLMASLIGGPLFLKFLG
QRLP----FKP---
--VPTEFADLKVRDEKTLP-SLGATLFTILLPIALMLVKTIAELNMARES
GLYILV-----------------EFIGNPITAMFIAVFVAYYVLGIRQHMSMG---------------TMLTH-TENGF-GSIANILLIIGAGGAFN
AILKSSSLADTLAVILSNM--------HMHPILLAWLVALILHAAVGSATVAMMGATAIVAPMLPLYPDI----------------SPEIIAI
AIGSGAIGCTIVTDSLFWLVKQYCGATL----NETFKYYTTATFIASVVALAGTFLLSFII---------------------------------------</s
eq-data>
</sequence>

<sequence seq-type="Protein">
<seq-name>GNTT_ECOLI</seq-name>
<seq-info><length>1375</length></seq-info>
<seq-data>--

---MPLVIVAIGVILLLLLMIR-FKMNGFIA
LVLVALAVGLMQGMPLD-----------KVIGSIKAGVAD-VGSLALIMGFGAMLGKMLADCGGAQRIATTLIAKFGKKHIQWA
VVLTGFTVGFALFY-EV---GFVLMLPLVFTIAASA---
-------------------NIPLLYVGVPMAAALSVTHGFL--PPHPGPTAIATIF-------NADMGKTLLYGT-ILAIPTVILAGPVYARVL
KGID------KP--
--IPEGLYSAKTFSEEEMP-SFGVSVWTSLVPVVLMAMRAIAEMILPK
GHAFLPVA-----------------EFLGDPVMATLIAVLIAMFTFGLNRGRSMD---------------QINDT-LVSSI-KIIAMMLLIIGGGG
AFKQVLVDSGVDKYIASMMHET--------NISPLLMAWSIAAVLRIALGSATVAAITAGGIAAPLIATTGV-----------------SPEL
MVIAVGSGSVIFSHVNDPGFWLFKEYFNLTI----GETIKSWSMLETIISVCGLVGCLLLNMVI-------------------------------
----</seq-data>
</sequence>
<sequence seq-type="Protein">
<seq-name>GNTP_ECOLI</seq-name>
<seq-info><length>1375</length></seq-info>
<seq-data>--

---MHVLNILWVVFGIGLMLVLNLK-FKINS
MVALLVAALSVGMLAGMDLM-----------SLLHTMKAGFGNTLGALAIIVVFGAVIGKLMVDSGAAHQIAHTLLARLGLRYV
QLSVIIIGLIFGLAMFY-EV---AFIMLAPLVIVIAAEA---
---------------------KIPFLKLAIPAVAAATTAHSLF--PPQPGPVALVNAY-------GADMGMVYIYGV-LVTIPSVICAGLILPKF
LGNLE------RP--
--TPSFLKADQPVDMNNLP-SFGVSILVPLIPAIIMISTTIANIWLVKD
TPAWEVV-----------------NFIGSSPIAMFIAMVVAFVLFGTARGHDMQ---------------WVMNA-FESAV-KSIAMVILIIGAG
GVLKQTIIDTGIGDTIGMLMSHG--------NISPYIMAWLITVLIRLATGQGVVSAMTAAGIISAAILDPATGQLVGV----------
NPALLVLATAAGSNTLTHINDASFWLFKGYFDLSV----KDTLKTWGLLELVNSVVGLIIVLIISMVA--------------------------
----------</seq-data>
</sequence>
<sequence seq-type="Protein">
<seq-name>GNTU_ECOLI</seq-name>
<seq-info><length>1375</length></seq-info>
<seq-data>--

--MTTLTLVLTAVGSVLLLLFLVMK-ARMHA
FLALMVVSMGAGLFSGMPLD-----------KIAATMEKGMGGTLGFLAVVVALGAMFGKILHETGAVDQIAVKMLKSFGHSR

AHYAIGLAGLVCALPLFF-EV---AIVLLISVAFSMARHT--
---------------------------GTNLVKLVIPLFAGVAAAAAFL--VPGPAPMLLASQM-------NADFGWMILIGL-CAAIPGMIIAGP
LWGNFISRYVE-----LH---
--IP-DDISEPHLGEGKMP-SFGFSLSLILLPLVLVGLKTIAARF
VPEGSTAYEWF-----------------EFIGHPFTAILVACLVAIYGLAMRQGMPKD---------------KVMEI-CGHAL-QPAGIILLVIG
AGGVFKQVLVDSGVGPALGEALTGM--------GLPIAITCFVLAAAVRIIQGSATVACLTAVGLVMPVIEQLNYSGA------------
--QMAALSICIAGGSIVVSHVNDAGFWLFGKFTGATE----AETLKTWTMMETILGTVGAIVGMIAFQLLS--------------------
------------------</seq-data>
</sequence>
<sequence seq-type="Protein">
<seq-name>O26881</seq-name>
<seq-info><length>1375</length></seq-info>
<seq-data>--

---MALAVYLVPLPGLRA--------SGHAALALLVFAVTMWATE
--AVPLAVTSLIILFAQPLIGVES------------------FENAVIGFANPILFLMIGGFIMAEAIRKSGLAQRFTYYLLGRLGTSPERGLF
VSIFSTGLLSAWI-ENVV-AFAMLLPIIKEIVDIMGCSEPEKGKS--
-------------------------NYAKAMILGASFGSLAGGFGTEIGTAPNLMAAAYTS----------IPFLNWMIFGL-PLSIAMLLVTWFL
LMRIFP--
---SEVRALENGDALIGERIRMMG-SMSRDEKLSAGILLFAILLWVTA--
---------------------------GFTGINSYSVSLIAAVMFIFA------------------------GVITW-KDAQK-NIDWGLVVFFGGALSLGSAL
LKTGAAAWIIDKLVGMLGS---DPSTVIVMLVLMAVAVIITQVMSNIALSAILVPLSVTLASAQHQ-----------------PIGVYAVP
VAIACSLSFMLPMADPTVAMAYGSG------YVKLRDIPRAGIPIIAVGIILTVLVITTLAAPFIS----------------------------------</
seq-data>
</sequence>
<sequence seq-type="Protein">
<seq-name>YFBS_ECOLI</seq-name>
<seq-info><length>1375</length></seq-info>
<seq-data>--

---MNGELIWVLSLLAVAIVLFATG--RVRM
DAVALFVIVAFALSGTLT------------------VPEVFSGFSDPNVVLIAALFIIGDGLVRTGVATVMGTWLVKVAGNSEIKMLVLL
MLTVAGLGAFM-SSTG-VVAIFIPVVLSVAMRMQT---
------------------------SPSRLMMPLSFAGLISGMMTLVATPPNLVVNSELLREG------YHGFSFFSVTPI-GLVVLVLGILYMLV
MRFMLKGDTQTPQREGWTRRTFRDLIREYRLTGRARRLAIRPGSPMIGQRLDDLKLRERYGANVIGVERWRRFRRVIVNV
NGVSEFRARDVLLIDMSAADVDLRQFCSEQLLEPMVL--------RGEYFSDQALDVGMAEISLIPESELIGKSVREIGFRTRYGL
NVVGLKRNGVALEGSLADEPLLLGDIILVVGNWKLIGMLAKQGRDFVALNLPEE-VSEASPAHSQAPHAIFCLVLMVALML
T----------------------------DEIPNPVAAIIACLLMGKF------------------------RCIDA-ESAYK-SIHWPSIILIVGMMPFAVALQ
KTGGVALAVKGLMDIGGG----YGPHMMLGCLFVLSAVIGLFISNTATAVLMAPIALAAAKTMGV-----------------SPYPFA
MVVAMAASAAFMTPVSSPVNTLVLGPG------NYSFSDFVKLGVPFTIIVMAVCVVMIPMLFPF------------------------------

------</seq-data>
</sequence>
<sequence seq-type="Protein">
<seq-name>Q9RUH5</seq-name>
<seq-info><length>1375</length></seq-info>
<seq-data>--

--MDPVTILLILFVVALVLFATE--WLPVDV
TALGLLSALLLFGLLK------------------PKEAFAGFGSDTVLTLASLFILTRVLLRAGVIEWIGHALARRARNANAMLRAML-GT
VAGVSSLT-SNTA-TTAVFLPVVSGLSRRAGI---
----------------PASRSLMPLAFASILGGTITVIGTSTNLVVSGALPATG------LKPLGFFELAWV-GVPVALVGLLYLFFVAPKLLP-
--------------DREAQLEDTLRAYLADLTVAPGSPLIGQSLRESGLGRDHGLTVVAVRRAG-PGGEGLTSYAPGA----DFIIQEGD
TLALEGPTERILAGKTNLGVVSRSEQKMLAAEGGTPGATVRLVEAVVLPGSPLLGRTLREAQFRERYGLSVLALHRRERTVE-
RLGQLRIRVGDVLLIQGQADRVAR---LGDRFAVLGD----LTDQQSDLKKAPLALLLFLGAVALGAT-------------------------------
GVVPLGVAVVVAAALSLIL-------------------------RLITP-EEAYG-SVEWPVIVLVACMLAFGTAFEQTGAAKVLTGALSGVLE
P----LGPYGLLGALFLVTVILTQPMSNQAAALVMLPLAIGTAKALGY------------------DPRPFVIGITVAASNSFITPLE-PSCML
VYGPG------HYTFMDFVRVGAGLTLLAFAVSMVVIPLVWPF--</seq-data>
</sequence>
<sequence seq-type="Protein">
<seq-name>Y640_SYNY3</seq-name>
<seq-info><length>1375</length></seq-info>
<seq-data>--

--MAFLQTWLADYQIPLTLAVIVFALVMFVLE--W
LPIDTTAILVAVILMVLGLVT-------------------PTEGIAGFSNSATVTIMAMFILSYGITRTGIIQIIRDSLIKFG-GDSLRRQLLLM
GFIVGPSSAFLNNTA-IVAIFLPIIEEWARQRQI--
------------------SVSKLLIPLSYATILGGMITLLGTSTNILASGLAEKLT------GQGFSIFQFTPL-GLLTFSVGLIYIVLAAPILLP------
--ARRNISDGNVAAEYQIKDYVSEIIIPPRSSLIGQTLRQSEIQRKFDIDVLEIIHNDTHFPQPLADRVLTMG---DILLVR--AGRE
DLLRIKDERGIEILAD-VQFVSAETNNAGPLESSEEKVAEVLILSNSRLIGSTLKDLRFRQRYNATVIAIRRGEELIRQRLGKVRLK
FGDLLLLQGPRESFLGLQT-TRELLVLEEKP--LDNLRLDKAKTAIAIVALVIVIAG---------------------------------LDILPISVTSWA
GVMAMVIS--------------------------GCLKP-GEIYG-AIRWDVIFLLAGLIPLGTAMENSGTTAWFASFLADAGGH----LSGYALL
LLFYLATALLTEILSNNATVVLMLPIAFQVAQSLGL------------------NPLAFMFVVTFAASNSFMSPIGYQTNTMVYGPG-----
-GYRFTDFARIGAPLTVILTLATPLLVMLIYGVQPTN------------------------------------</seq-data>
</sequence>
<column-score>
<colsco-name>coreblock</colsco-name>
<colsco-owner>0</colsco-owner>
<colsco-type>int</colsco-type>
<colsco-data>0 0 0 0 0 0 0 0 0 0 0 0 0 0 0 0 0 0 0 0 0 0 0 0 0 0 0 0 0 0 0 0 0 0 0 0 0 0 0 0 0 0 0 0 0 0 0 0 0 0 0 0 0 0 0 0 0 0 0 0 0
0 0 0 0 0 0 0 0 0 0 0 0 0 0 0 0 0 0 0 0 0 0 0 0 0 0 0 0 0 0 0 0 0 0 0 0 0 0 0 0 0 0 0 0 0 0 0 0 0 0 0 0 0 0 0 0 0 0 0 0 0 0 0 0 0 0 0 0 0

0 0 0 0 0 0 0 0 0 0 0 0 0 0 0 0 0 0 0 0 0 0 0 0 0 0 0 0 0 0 0 0 0 0 0 0 0 0 0 0 0 0 0 0 0 0 0 0 0 0 0 0 0 0 0 0 0 0 0 0 0 0 0 0
0 0 0 0 0 0 0 0 0 0 0 0 0 0 0 0 0 0 0 0 0 0 0 0 0 0 0 0 0 0 0 0 0 0 0 0 0 0 0 0 0 0 0 0 0 0 0 0 0 0 0 0 0 0 0 0 0 0 0 0 0 0 0 0
0 0 0 0 0 0 0 0 0 0 0 0 0 0 0 0 0 0 0 0 0 0 0 0 0 0 0 0 0 0 0 0 0 0 0 0 0 0 0 0 0 0 0 0 0 0 0 0 0 0 0 0 0 0 0 0 0 0 0 0 0 0 0 0
0 0 0 0 0 0 0 0 0 0 0 0 0 0 0 0 0 0 0 0 0 0 0 0 0 0 0 0 0 0 0 0 0 0 0 0 0 0 0 0 0 0 0 0 0 0 0 0 0 0 0 0 0 0 0 0 0 0 0 0 0 0 0 0
0 0 0 0 0 0 0 0 0 0 0 0 0 0 0 0 0 0 0 0 0 0 0 0 0 0 0 0 0 0 0 0 0 0 0 0 0 0 0 0 0 0 0 0 0 0 0 0 0 0 0 0 0 0 0 0 0 0 0 0 0 0 0 0
0 0 0 0 0 0 0 0 0 0 0 0 0 0 0 0 0 0 0 0 0 0 0 0 0 0 0 0 0 0 0 0 0 0 0 0 0 0 0 0 0 0 0 0 0 0 0 0 0 0 0 0 0 0 0 0 0 0 0 0 0 0 0 0
0 0 0 0 0 0 0 0 0 0 0 0 0 0 0 0 0 0 0 0 0 0 0 0 0 0 0 0 0 0 0 0 0 0 0 0 0 0 0 0 0 0 0 0 0 0 0 0 0 0 0 0 1 1 1 1 1 1 1 1 1 1 1 1
0 0 0 0 1 1 1 1 1 1 1 1 1 1 1 1 1 1 1 1 1 1 1 1 1 1 1 0 0 0 0 0 0 0 0 0 0 0 0 0 0 0 0 0 0 0 0 0 0 0 0 0 1 1 1 1 1 1 1 1 1 1 1 1
1 1 1 1 1 1 1 1 1 1 1 1 0 1 1 1 1 1 1 1 1 1 1 1 1 1 0 0 0 0 0 1 1 1 1 1 1 1 1 1 1 1 1 1 1 1 1 1 1 1 1 1 0 0 0 0 0 1 1 0 1 1 1
1 1 1 1 1 1 0 0 0 0 0 0 0 0 0 0 0 0 0 0 0 0 0 0 0 0 0 0 0 0 0 0 0 0 0 0 0 0 0 0 0 0 0 0 0 0 0 0 0 0 0 0 0 0 0 0 0 0 0 0 0 0 0 0
0 0 0 0 0 0 0 0 0 0 0 0 0 0 0 0 0 0 0 0 0 0 0 0 0 0 0 0 0 0 0 0 0 0 0 0 0 0 0 0 0 0 0 0 0 0 0 0 0 0 0 0 0 0 0 0 0 0 0 0 0 0 0 0
0 1 1 1 1 1 1 1 1 1 1 1 1 1 1 1 1 1 1 1 1 1 1 1 1 1 1 0 0 0 0 0 0 0 0 0 0 0 0 0 0 0 0 0 0 0 0 0 0 0 1 1 1 1 1 1 1 0 0 1 1 1 0 1
1 1 1 1 1 1 1 1 1 1 1 1 1 0 0 0 0 0 0 0 0 0 0 0 0 0 0 0 0 0 0 0 0 0 0 0 0 0 0 0 0 0 0 0 0 0 0 0 0 0 0 0 0 0 0 0 0 0 0 0 0 0 0 0
0 0 0 0 0 0 0 0 0 0 0 0 0 0 0 0 0 0 0 0 0 0 0 0 0 0 0 0 0 0 0 0 0 0 0 0 0 0 0 0 0 0 0 0 0 0 0 0 0 0 0 0 0 0 0 0 0 0 0 0 0 0 0 0
0 0 0 0 0 0 0 0 0 0 0 0 0 0 0 0 0 0 0 0 0 0 0 0 0 0 0 0 0 0 0 0 0 0 0 0 0 0 0 0 0 0 0 0 0 0 0 0 0 0 0 0 0 0 0 0 0 0 0 0 0 0 0 0
0 0 0 0 0 0 0 0 0 0 0 0 0 0 0 0 0 0 0 0 0 0 0 0 0 0 0 0 0 0 0 0 0 0 0 0 0 0 0 0 0 0 0 0 0 0 0 0 0 0 0 0 0 0 0 0 0 0 0 0 0 0 0 0
1 1 1 1 1 1 1 1 1 1 1 1 1 1 1 1 0 0 0 0 0 0 0 0 0 0 0 0 0 0 0 0 0 0 0 0 0 0 0 0 0 0 0 0 0 0 0 0 0 0 0 0 0 0 0 0 0 0 1 1 1 1 1 1
1 1 1 1 1 1 1 1 0 0 0 0 0 0 0 0 0 0 0 0 0 0 0 0 0 0 0 0 0 0 0 0 0 0 0 0 0 0 0 0 0 0 0 0 0 0 0 0 0 0 0 0 0 0 0 1 1 1 1 1 1 1 1 0
0 0 1 1 1 1 1 1 1 1 1 1 1 1 1 1 1 1 1 1 1 1 1 1 1 1 1 1 0 0 0 0 0 0 0 0 0 0 1 1 1 1 1 1 1 1 1 1 1 1 1 1 1 1 1 1 1 1 1 1 1 1 1 1
1 1 1 1 1 1 1 1 0 0 0 0 0 0 0 0 0 0 0 0 0 0 0 0 0 0 0 0 0 0 0 0 0 0 0 0 0 0 0 0 0 0 0 0 0 0 1 1 1 1 1 1 1 1 1 1 1 1 1 1 1 1 1 1
0 0 0 0 0 0 0 0 0 0 0 0 0 0 0 0 0 0 1 1 1 1 1 1 1 1 1 1 1 1 1 1 1 1 1 1 1 1 1 1 1 1 1 1 0 0 0 0 0 0 0 0 0 0 0 0 0 0 0 0 0 0 0 0
0 0 0 0 0 0 0 0 0 0 0 0 0 0 0 0 0 0 0 0 0 0 0 0 0 0 0 0 </colsco-data>
</column-score>
</alignment>
</macsim>
:::::::::::::
acr_core-block.xml
:::::::::::::
<?xml version="1.0"?>
<!DOCTYPE macsim SYSTEM "http://www-bio3d-igbmc.u-strasbg.fr/macsim.dtd">
<macsim>
<alignment>
<aln-name>1</aln-name>
<sequence seq-type="Protein">
<seq-name>O66977</seq-name>
<seq-info><length>1374</length></seq-info>
<seq-data>----------------------------MQKFFIERPVTAFMLMLSFVFLGLYSLKLVPIDRLPDVEFPVVRISADYPGADPYVV
DTNVTRIIEEAVST-VSGIESIISRS-YPGFASVVVTFTLDKDIDVATQEVSNAIQRAMRRFPKGVE-PPTVVKVDTSVAPIMVL
LLYSDT---------------------------------ADYQRLT-YYADKVIKRELEKLYGVGSVSLGGFRDNVLWVRLKVSELYGYELTPLDVL
REIDKNHQDIPAGNVYG------KRREYILRLYGKFKTPEELSNLYIK--ENLRLGDVARVY
FG-----------EDEERSLARFNGN-----RAVALLVFKQAKVNTVRVIDEIKSRMEFFR--ELLPPDIKLDYTFDASVYVKESIRAAFE
EIIIGVFLTAFTVYLFLGNLRLTLVPVFAIPVTILGTIFFLYQLGYSLNTITLLALAVAVGIVIDDAIVVMESIHRRR-------------EEG

LPPFEAGVKGTRVVMFALLASTSVLIVVFLPVVFLK---GVLGTFLKN--FALTLIIAIGLSYLVAITFTPSATVRLITQRPREN------
---FFMKLYKRFEGSFDKALRFSLDHKFIVLLLSFFFVGLS
VYLF-PKVKKEFVPHVDEGRFMVRMRMPVGTSFERVKEKAKEVERILLK---NPHIAKVGMVVGEGVGG-----AGVHSGLFF
VYLKERSQRPH---------------QMVVMSEVRRELSE------------------LKNVKVSVEPPHGIGASAGRTTDLQYIVRGERIEVL
KKIAKSMEEHF-SNVE-GFRD----VDTSLELSAPYVKISVKREKLSHLGVSVEDLGLTLSLLF---GKYRVGTYELG--SESYDFYIKA
EEDFVKEIHNLNRVYIRARNGE-----LIPITELIDFSIEAGPFSLTRYNRQYSFVFYANL---EGIDLATARSLVEDWLKKN----------
------LPFGYTYDTAGRVKEFK-KAFSGFVLSLAMSLIGVYMILASLFESLKHPFTVLLMVPLSALGVFGFMYLT-----------DTSL
NVSSYFGIILLVGIIVRDAVLFIERIIQLRK----------EGYAVREAILEARRERLRPILMTTITVVSALLPVAL-GLTAGSEQRKPLAI
AVIGGIASGLPLSLFLLPVLYELMEWKRK---------------------------</seq-data>
</sequence>
<sequence seq-type="Protein">
<seq-name>O68962</seq-name>
<seq-info><length>1374</length></seq-info>
<seq-data>---------------------------MYKTAINRPITTLMFALAIVFFGTMGFKKLSVALFPKIDLPTVVVTTTYPGASAEIIES
KVTDKIEEAVMG-IDGIKKVTSTS-SKNVSIVVIEFELEKPNEEALNDVVNKISSV-RFDDSNIK-KPSINKFDTDSQAIISLFVSSS
S------------------------------VPATTLN-DYAKNTIKPMLQKINGVGGVQLNGFRERQIRIYADPTLMNKYNLTYADLFSTLK
AENVEIDGGRIVN------SQRELSILINANSYSVADVEKIQV---GNHVRLGDIAKIEIG----
-------LEEDNTFASFKDK-----PGVILEIQKIAGANEIEIVDRVYEALKHIQ--AISPR-YEIRPFLDTTSYIRTSIEDVKFDLILGAILAV
LVVFAFLRNGTITLVSAISIPISIMGTFALIQWMGFSLNMLTMVALTLAIGIIIDDAIVVIENIHKKL------------EMGMNKRKA
SYEGVREIGFALVAISAMLLSVFVPIGNMK---GIIGRFFQS--FGITVALAIALSYVVVVTIIPMVSSVVVNPR------------------
--HSRFYVWSEPFFKALESRYTRLLQWVLNHKLIISIAVVLVFVGSLF
VA-SKLGMEFMLKEDRGRFLVWLKAKPGVSIDYMTQKSKIFQKAIEK---HAEVEFTTLQVGYGTT------QNPFKAKIFVQLK
PLKERKK----------ESELGQFELMSALRKELKS----------------MPEAKGLDTINLSEVSLLGGGGDSSPFQTFVFSHSQEAVD
KSVANLKKFL-LESP-ELKGKIEGYHTSTSESQPQLQLKILRQNANKYGVSAQTIGAVVSSAF--SGTSQASVFKED--GKEYDMI
IRVPDDKRVSVEDIKRLQVRNKYDK-----LMFLDALVEITETKSPSSISRYNRQRSVTVLAQP--KAGISLGEILTQVSKNTKEW-
--------------LVEGANYRFTGEADNAK-ETNGEFLVALATAFVLIYMILAALYESILEPFIIMVTMPLSFSGAFFALGLV---------
--HQPLSMFSMIGLILLIGMVGKNATLLIDVANEERK----------KGLNIQEAILFAGKTRLRPILMTTIAMVCGMLPLAL-ASG
DGAAMKSPIGIAMSGGLMISMVLSLLIVPVFYRLLAPIDDKIKRFYQNQKALE----------------</seq-data>
</sequence>
<sequence seq-type="Protein">
<seq-name>O25328</seq-name>
<seq-info><length>1374</length></seq-info>
<seq-data>---------------------------MYKTAINRPITTLMFALAIVFFGTMGFKKLSVALFPKIDLPTVVVTTTYPGASAEIIES
KVTDKIEEAVMG-IDGIKKVTSTS-SKNVSIVVIEFELEKPNEEALNDVVNKISSV-RFDDSNIK-KPSINKFDTDSQAIISLFVSSS
S------------------------------VPATTLN-DYAKNTIKPMLQKINGVGGVQLNGFRERQIRIYANPTLMNKYNLTYADLFSTLK
AENVEIDGGRIVN------SQREFSILINANSYSVADVEKIQV---GNHVRLGDIAKIEIG----
-------LEEDNTFASFKDK-----PGVILEIQKIAGANEIEIVDRVYEALKRIQ--AISPN-YEIRPFLDTTGYIRTSIEDVKFDLVLGAILA
VLVVFAFLRNGTITLVSAISIPISIMGTFALIQWMGFSLNMLTMVALTLAIGIIIDDAIVVIENIHKKL------------EMGMSKRK
ASYEGVREIGFALVAISAMLLSVFVPIGNMK---GIIGRFFQS--FGITVALAIALSYVVVVTIIPMVSSVVVNPR------------------
---HSRFYVWSEPFFKALESRYTKLLQWVLNHKIIISIAVVLVFVGSLF
VA-SKIGMEFMLKEDRGRFLVWLKAKPGVSIDYMTQKSKIFQKAIEK---HAEVEFTTLQVGYGTT------QNPFKAKIFVQLKP
LKERKK----------EHQLGQFELMSVLRKELRS----------------LPEAKGLDTINLSEVTLIGGGGDSSPFQTFVFSHSQEAVDKS

VENLKKFL-LESP-ELKGKVESYHTSTSESQPQLQLKILRQNANKYGVSAQTIGSVVSSAF--SGTSQASVFKED--GKEYDMIIR
VPDDKRVSVEDIKRLQVRNKYDK-----LMFLDALVEITETKSPSSISRYNRQRSVTVLAEPNRNAGVSLGEILTQVSKNTKEW-
---------------LVEGANYRFTGEADNAK-ESNGEFLVALATAFVLIYMILAALYESILEPFIIMVTMPLSFSGAFFALGLV----------
--HQPLSMFSMIGLILLIGMVGKNATLLIDVANEERK-----------KGLNIQEAILFAGKTRLRPILMTTIAMVCGMLPLAL-ASG
DGAAMKSPIGIAMSGGLMISMVLSLLIVPVFYRLLAPIDDKIKRFYQNQKTLE-----------------</seq-data>
</sequence>
<sequence seq-type="Protein">
<seq-name>YEGO_ECOLI</seq-name>
<seq-info><length>1374</length></seq-info>
<seq-data>---------------------------MKFFALFIYRPVATILLSVAITLCGILGFRMLPVAPLPQVDFPVIIVSASLPGASPETMA
SSVATPLERSLGR-IAGVSEMTSSS-SLGSTRIILQFDFDRDINGAARDVQAAINAAQSLLPSGMPSRPTYRKANPSDAPIMIL
TLTSDT---------------------------------YSQGELY-DFASTQLAPTISQIDGVGDVDVGGSSLPAVRVGLNPQALFNQGVSLDD
VRTAVSNANVRKPQGALED------GTHRWQIQTNDELKTAAEYQPLIIH--YNNGGAVRL
GDVATVTDS-----------VQDVRNAGMTNAK-----PAILLMIRKLPEANIIQTVDSIRAKLPELQ--ETIPAAIDLQIAQDRSPTIRA
SLEEVEQTLIISVALVILVVFLFLRSGRATIIPAVSVPVSLIGTFAAMYLCGFSLNNLSLMALTIATGFVVDDAIVVLENIARHL---
----------EAGMKPLQAALQGTREVGFTVLSMSLSLVAVFLPLLLMG---GLPGRLLRE--FAVTLSVAIGISLLVSLTLTPMMCG
WMLKAS---KPREQKRLRGFGRMLVALQQGYGKSLK
WVLNHTRLVGVVLLGTIALNIWLY-ISIPKTFFPEQDTGVLMGGIQADQSISFQAMRGKLQDFMKIIRD---DPAVDNVTGFT
GGSRV---------NSGMMFITLKPRDERSE---------------TAQQIIDRLRVKLAK-----------------EPGANLFLMAVQDIRVGGRQ
SNASYQYT--LLSDDLAALREWEPKIRKKL-ATLP-ELAD----VNSDQQDNGAEMNLVYDRDTMARLGIDVQAANSLLNNAF
---GQRQISTIYQP--MNQYKVVMEVDPRYTQDISALEKMFVINNEGK-----AIPLSYFAKWQPANAPLSVNHQGLSAASTISF
NL--PTGKSLSDASAAIDRAMTQL---------------GVPSTVRGSFAGTAQVFQ-ETMNSQVILIIAAIATVYIVLGILYESYVHPLTI
LSTLPSAGVGALLALELF------------NAPFSLIALIGIMLLIGIVKKNAIMMVDFALEAQRH----------GNLTPQEAIFQACLLRF
RPIMMTTLAALFGALPLVL-SGGDGSELRQPLGITIVGGLVMSQLLTLYTTPVVYLFFDRLRLRFSRKPKQTVTE---------------
---</seq-data>
</sequence>
<sequence seq-type="Protein">
<seq-name>YEGN_ECOLI</seq-name>
<seq-info><length>1374</length></seq-info>
<seq-data>------------------MQVLPPSSTGGPSRLFIMRPVATTLLMVAILLAGIIGYRALPVSALPEVDYPTIQVVTLYPGAS
PDVMTSAVTAPLERQFGQ-MSGLKQMSSQS-SGGASVITLQFQLTLPLDVAEQEVQAAINAATNLLPSDLPNPPVYSKVNP
ADPPIMTLAVTSTA---------------------------------MPMTQVE-DMVETRVAQKISQISGVGLVTLSGGQRPAVRVKLNAQAI
AALGLTSETVRTAITGANVNSAKGSLDG------PSRAVTLSANDQMQSAEEYRQLIIA--Y
QNGAPIRLGDVATVEQG-----------AENSWLGAWANKE-----QAIVMNVQRQPGANIISTADSIRQMLPQLT--ESLPKSVK
VTVLSDRTTNIRASVDDTQFELMMAIALVVMIIYLFLRNIPATIIPGVAVPLSLIGTFAVMVFLDFSINNLTLMALTIATGFVV
DDAIVVIENISRYI-------------EKGEKPLAAALKGAGEIGFTIISLTFSLIAVLIPLLFMG---DIVGRLFRE--FAITLAVAILISAVVSL
TLTPMMCARMLSQE---SLRKQNRFSRASEKMFDRIIA
AYGRGLAKVLNHPWLTLSVALSTLLLSVLLW-VFIPKGFFPVQDNGIIQGTLQAPQSSSFANMAQRQRQVADVILQ---DPA
VQSLTSFVGVDG-----TNPSLNSARLQINLKPLDERDD---------------RVQKVIARLQTAVDK-----------------VPGVDLFLQPT
QDLTIDTQVSRTQYQFT--LQATSLDALSTWVPQLMEKL-QQLP-QLSD----VSSDWQDKGLVAYVNVDRDSASRLGISMA
DVDNALYNAF---GQRLISTIYTQ--ANQYRVVLEHNTENTPGLAALDTIRLTSSDGG-----VVPLSSIAKIEQRFAPLSINHLDQF
PVTTISFNV--PDNYSLGDAVQAIMDTEKTL---------------NLPVDITTQFQGSTLAFQ-SALGSTVWLIVAAVVAMYIVLGILY

ESFIHPITILSTLPTAGVGALLALLIA------------GSELDVIAIIGIILLIGIVKKNAIMMIDFALAAERE----------QGMSPREAIYQA
CLLRFRPILMTTLAALLGALPLML-STGVGAELRRPLGIGMVGGLIVSQVLTLFTTPVIYLLFDRLALWTKSRFARHEEEA------
-----------</seq-data>
</sequence>
<sequence seq-type="Protein">
<seq-name>y895_haein</seq-name>
<seq-info><length>1374</length></seq-info>
<seq-data>---------------------MYEEIRMKFTDIFIRRPVLAVSISLLMIILGLQAISKLAVREYPKMTTTVITVSTAYPGADANLI
QAFVTSKLEESIAQ-ADNIDYMSSTS-APSSSTITIKMKLNTDPAGALADVLAKVNAVKSALPNGIE-DPSVS-SSSGGSGIMYI
SFRSKK--------------------------------LDSSQVT-DYINRVVKPQFFTIEGVAEVQVFGAAEYALRIWLDPQKMAAQNLSVPT
VMSALSANNVQTAAGNDNG------YYVSYRNKVETTTKSVEQLSNLIIS--SNGDDLVRLR
DIATVELN-----------KENDNSRATANGA-----ESVVLAINPTSTANPLTVAEKIRPLYESIK--TQLPDSMESDILYDRTIAINSSIH
EVIKTIGEATLIVLVVILMFIGSFRAILIPILAIPISLIGVLMLLQSFNFSINLMTLLALILAIGLVVDDAIVVLENIDRHI-------------K
AGETPFRAAIIGTREIAVPVISMTIALIAVYSPMALMG---GITGTLFKE--FALTLAGAVFISGVVALTLSPMMSSKLLKSN------
---AKPTWMEERVEHTLGKVNRVYEYMLDLVMLNRKSM
LAFAVVIFSTLPFLF-NSLSSELTPNEDKGAFIAIGNAPSSVNVDYIQNAMQPYMKNVME---TPEVSF-----GMSIAG----APT
SNSSLNIITLKDWKERSR---------------KQSAIMNEINEKAKS-----------------IPEVSVSAFNIPEIDTGEQGPPVSIVLK---TAQ
DYKSLANTAEKFLSAM-KASG-KFIY----TNLDLTYDTAQMTISVDKEKAGTYGITMQQISNTLGSFL---SGATVTRVDVD--G
RAYKVISQVKRDDRLSPESFQNYYLTASNGQ-----SVPLSSVISMKLETQPTSLPRFSQLNSAEISAVP--MPGISSGDAIAWLQ
QQATDN----------------LPQGYTFDFKSEARQLV-QEGNALAVTFALAVIIIFLVLAIQFESIRDPMVIMISVPLAVSGALVSLNI
LSFFSIA------GTTLNIYSQVGLITLVGLITKHGILMCEVAKEEQLN----------HGKTRIEAITHAAKVRLRPILMTTAAMVAGLIP
LLY-ATGAGAVSRFSIGIVIVAGLSIGTIFTLFVLPVVYSYVATEHKPLPVFDENKTTH------------------</seq-data>
</sequence>
<sequence seq-type="Protein">
<seq-name>Q9ZDZ3</seq-name>
<seq-info><length>1374</length></seq-info>
<seq-data>---------------------------MLLSTICIKRPIFAIVLSLMIVALGGIFFTKLQIRGAPDISVPIINVEAHYAGADALYMEQ
EITTRIEKVLKT-VKNLDYITSQS-STGSSSITLSFRLSTDIELALNDVRSKISDISYMFPQDMK-APSVTKLDTDNFPSLFISIESDQ
---------------------------------YSDLELT-QIVSDNIQAQLDKLESVGKSQIYGGREYIMRIEPDFKKLYQYKISLLEIEAAIKEQNK
DYPAGMIKT------KSNNFIVTLTGSLSAPEEFGNIILK---VQNGCIIKLRDITKISLT-----------
SPDEDIIFRYNGK-----NSIALGLIKESKANIIDLSNEVTKELERIK--ASIPKGISISIAYDGAKPLKASIYAVFKTMFEALILVVLVTY
LFLASAKISLIPFVTIPVSLIGTFSIMYAFGFSINVFTLLAMILAIGLVVDDAIVMLENIFRYN-------------EMGYKPMEAAMLA
SKEIGFAIITMTITLAAVFVPIGFIE---GFIGKLFIE--FAWTLAFCVLFSGFVAVTLTPMMSSHMVTKH--------------------------
---NTNLAKFLVRFHDMLQFVQNRYIYYLKLALDNKKKFVIIILSSFIVLIISF-KLT
KKTFVPQEDDGFLQVFLKGPEGASLESSIKVVKEAEQILAN---YKDIFGYLMVIGVG---------GSENVFGFIPLKDWSARSR---
------------SQKTIKNMLNKQFSK-----------------IPGMSVFAMEPRSMISVNATSPIEFTIQ---TNLEYKDLDKISQQFIDIM-R
ANP-IFLN----VNRNLQSAMPTINIEVNRDKAYLYGMDLANIGKTLQYLI---AGQQIGAFRMG--NNLYNVILQFNQKDRQD
ISDLSKILIRTKNNN-----MLPLESIANIREKISLKSYYHYNNSKSVTISSDL--APDVKINDAINIINKIAAQL---------------LDPNN
TIIEYIGEIKQMK-EVDNNILITFVFSLVFIYLVLAAQFESFTDPLLILLAVPFSITGGGLTLWLA------------GNSLNMYSNIGLITL
IGLVTKNAIMIVEFANQLRA-----------KGLNVREAIIESSKLRLRPILMTTVAAIVGALPLVF-ADGAGAASRNSIGFVIVGGLSI
GTIFTIFAIPVIYQTFKKD--------------------------------</seq-data>
</sequence>

<sequence seq-type="Protein">
<seq-name>p73998</seq-name>
<seq-info><length>1374</length></seq-info>
<seq-data>----------------------------MFVDFFIKRPVFSSVCAIIILLVGTISIFSLPIAQFPEVAPTTIQVSSNYSGANAEVVERA
VTDILERQING-VQGMRYISSTSSNDGTSSITVTFDRSQNKDIAAVDVQNRVALAEPQLPEAVR-RTGIRVNKESNALLLGIGI
TSPDG-------------------------------EYDNVFLS-NYADRYLVDPIRRLEGVGDVRIFGERLYAMRLWVDPMKLAAQQLTMAD
LSRALQEQNLQVGAGQIGAEPAPPGQEYQLDLLASSQLVEVKDFEDLIVK--SGASGSVV
RFKDIGRVELG-----------AQNYNSFLRFRGD-----EAVGLGIYQLLDSNALEVARLVKDEMARLA--QNFPEGIEYSVAFDTTE
FVQESLSEVVETLLIAVVLVILVILVFLQDWRSALIPALTIPLALIGTFAFVKVFNFSINSLTLFGLTLATGLVVDDAIIVVEQISRFI
K------------VKHEDPQEAAQEAMGELTGAVIATSLVLMAVFIPVAFFP---GTTGALYQQ--FALTIAFSILLSTFLALTLTPSLCA
LLLREGQEP---PAFIA-GFFNWFNRVLDIIKNGYGNVLGK
LVNLRAWVIGVFVLLLGATAWLY-VTVPTAFLPEEDQGYFITIIQAPQGVSLQYTSRVMAQVEKELLA---VPEVTATFAVGG
FSFSG-----NSPNQGIIFTRLKPWGERTA------------PNQSVQAIIGQMFGKFSQ-----------------IPEANIIPINPPPIRGLGQF
GGFDFQLQDLRVNSELDTMVGTMGEILGAA-NQNP-ALTR----VFSTFQANNPQLIVNVNRNKAKSLGVPVDQIFQTMET
AL---GSSYVNDFVLQ--GRTYRVYLQADEQFRSSPEDINSLYVRSESGT-----MIPMANLVTVTQGVGAPIITHYNLFRSIAITG
SA--NFGVSTGQAMNAMAAIARQV----------------MPPGFDFQWSGISLEEM-GSQGQAPLIFGLGLLFVFLVLAAQYENYI
DPVIILLSVPLAILGALTAQSLR------------GFPNDVYCQIGLVMLIGLSSKNAILIVEFANQLRA-----------EGYPIAKAALEASK
DRLRPILMTALSTLFGIFPLAI-ATGAGAGSRQALGTAVFGGMLVATFLSLFVVPVLYIVVKTISGRLLAPKSTNPEPSPEIESHT
PAPASQRNS-</seq-data>
</sequence>
<sequence seq-type="Protein">
<seq-name>q55584</seq-name>
<seq-info><length>1374</length></seq-info>
<seq-data>-------------------------MLLSISNVFIKNPVLTTVCTIVIILLGAIALPLLPLAKLPDMAPKQVQVTTNYVGSDAQTA
VDNVTTVLERQING-TEQVIYMNSTTDNTGTSTINVYFPVEMDRNIAQVLVQNNVAIAASSLPEVVN-RQGVTTQTQSPSV
TIAYGVYSENDD--------------------------QGKPIYDDIFVS-NFVDRVLLDEIKRIDGVGSAILIGASEYAMRFWLDPDALAARD
LTAADVTNAIRSQNIQVGVGGVNLPPVTDQQRFQINARALSRFTTPEEAEQIVVK--VGD
DGTLIRIKDVGRATIG-----------TQNYIQTALFNNA-----PAVAFVIYQLPGTNALDTANMVKEKMAELR--PLFPPGLNAEVA
LDNTLFVTASLEEAALTLIEAILLVILVIFIFLQNWRTTLIPALAIPVSLIGAMAFALAFGFSLNQLTLFGVILATGLVVDDGILVVE
AIEVKL-------------DQGMKPFQAALDAMGELTGAVISTSLVLMAVFIPVTFFP---GTTGIVYKQ--FAVIMASAVAVSTFNAI
SFSPSMSAILMRPKKEVH--GPLAWFFNLFNRTFDWLK
ERYGNIITAILKVRLLAIPIFIGSLILTVIVY-NITPTGFIPEEDQGYFFMLGNSPAGVSIEYTKDVISQATEIVSA---RPEVEHVLG
MGGFSFLG-----NDSSKSLFFVKLKNWDERPG------------QKGSVFGLLAEINRELAQ----------------KIPDAQVFAVNAPPV
DGLSSTGGLDFYIQN-RGGMPLENFLDYVQQYMEKL-RQEP-ALNPRT--VFTQFTFNAPLLEIGVDREKANAQNVDISEVF
NTIGIYM---GSSYINQFVME--SRLYQVYAMADGQFRSNPRDIGRLYVRSRTGA-----LVQLSNLIDVKQTTYPPILTNFNIYP
AVDVQASP--AAGYSTGQAMATMERLSKEM----------------FPDSIGYAWYGTGYEEL-QSAGAAPIIFGLAFIMVFLVLSAQ
YESYVDPAIIMMTVPLAILGAIGAILLRANFMVATNMVWPTVNNNVYAQVALVMLIGLASKNAILIVEFGNQAMD---------
--LGMKIPQAAAFAAKERMRPILMTAISGLVGFWPLVI-ASGAGAMSRWSLGTAIFGGYLISTILSLFLVPVLYTLVKEAEARF
LKGEKGEGDSNPPPPSGEDNGEPVNNPS</seq-data>
</sequence>
<sequence seq-type="Protein">
<seq-name>p95422</seq-name>

<seq-info><length>1374</length></seq-info>
<seq-data>---------------------------MNFSQFFIQRPIFAAVLSLLILIGGAISLFQLPISEYPEVVPPTVVVRANFPGANPKVIGE
TVASPLEQAITG-VENMLYMSSQSTSDGKLTLTITFALGTDLDNAQVQVQNRVTRTEPKLPEEVT-RLGITVDKASPDLTMV
VHLTSPDN-------------------------------RYDMLYLS-NYAVLNVKDELARLDGVGDVQLFGLGDYSLRVWLDPNKVASRNLT
ATDVVNAIREQNRQVAAGTLGAPPAPNDTIFQLSINTQGRLVTEEEFENIIIR--AGANGEI
TRLRDIARVELG-----------SNQYALRSLLNNK-----PAVAIPIFQRPGSNAIEISNLVREKMAELK--HSFPQGMDYSIVYDPTIF
VRGSIEAVVHTLFEALVLVVLVVILFLQTWRASIIPLAAVPVSLIGTFAVMHMLGFSLNALSLFGLVLAIGIVVDDAIVVVENV
ERNI-------------GLGLKPVEATKRAMREVTGPIIATALVLCAVFIPTAFIS---GLTGQFYRQ--FALTIAISTVISAFNSLTLSPALA
AVLLKGHHEPKDRFSVFL---DKLLGSWLFRPFNRFFDRASHGYV
GTVNRVLRGSSIALLVYGGLMVLTYFGF-SSTPTGFVPQQDKQYLVAFAQLPDAASLDRTEAVIKQMSEIALA---QPGVADS
VAFPGLSING---FTNSPNSGIVFTPLKPFDERKD-------------PSQSAGAIAAALNAKYAD-----------------IQDAYIAIFPPPPVQ
GLGTIGGFRLQIED-RGNQGYEELFKQTQNIITKA-RALP-ELEPSS--VFSSYQVNVPQIDADIDREKAKTHGVAISDIFDTLQV
YL---GSLYANDFNRF--GRTYQVNVQAEQQFRLEPEQIGQLKVRNNLGE-----MVPLASFIKVSDTSGPDRVMHYNGFITAE
LNGAP--AAGYSSGQAQAAIEKLLKEE-----------------LPNGMTYEWTELTYQQI-LAGNTALFVFPLCVLLAFLVLAAQYESW
SLPLAVILIVPMTLLSAITGVILA-------------GSDNNIFTQIGLIVLVGLACKNAILIVEFAKDKQE------------EGMDRVAAVLEAC
RLRLRPILMTSIAFIMGVVPLVI-STGAGAEMRHAMGVAVFSGMIGVTFFGLLLTPVFYVLIRRFVENREARRAANDKGLPE
VHA-------------</seq-data>
</sequence>
<sequence seq-type="Protein">
<seq-name>o06471</seq-name>
<seq-info><length>1374</length></seq-info>
<seq-data>---------------------------MNISKFFIDRPIFAGVLSVIILLGGVIAMFLLPISEYPEVVPPSVIVKAQYPGANPKVIAE
TVASPLEEQING-VENMLYMQSQANSDGNMTITVTFKLGTDPDKATQLVQNRVNQALPRLPEDVQ-RLGITTVKSSPTLT
MVVHLISPDN-------------------------------RYDMTYLR-NYALINVKDRLSRIQGVGQVQLWGSGDYAMRVWLDPQKVAQ
RGLSAEDVVQAIREQNVQVAAGVIGASPSLPGTPLQLSVNARGRLQTEDEFGDIVVK--T
TPDGGVTHLRDIARIQLD-----------ASEYGLRSLLDNK-----PAVAMAINQSPGANSLQISDEVRKTMAELK--QDMPAGVD
YKIVYDPTQFVRSSIKAVVHTLLEAIALVVIVVIVFLQTWRASLIPLIAVPVSIIGTFSLLLAFGYSINALSLFGMVLAIGIVVDDAI
VVVENVERNI-------------ENGMNARQATYKAMQEVSGPIIAIALTLVAVFVPLAFMS---GLTGQFYKQ--FAMTIAISTVISA
FNSLTLSPALSAILLKGHGDKEDWLTRVM---NRVLG-GFFRGFN
KVFHRGAENYGRGVRGVLSRKTLMLGVYLVLVGATVLVS-KVVPGGFVPAQDKEYLIAFAQLPNGASLDRTEKVIRDMGSI
ALK---QPGVESAVAFPGLSVNG---FTNSSSAGIVFVTLKPFAERHG-------------KALSAGAIAGALNQKYGA-----------------M
KDSFVAVFPPPPVLGLGTLGGFKMQIED-RGAVGYAKLSDATNDFIKRA-QQAP-ELGP----LFTSYQINVPQLNVDLDRVKA
KQLGVPVTDVFNTMQVYL---GSLYVNDFNRF--GRVYQVRVQADAPFRQRADDILQLKTRNDKGE-----MVPLSSLVTVTP
TFGPEMVVRYNGYTAADINGGP--APGFSSGQAQAAVERIADET-----------------LPRGVRFEWTDLTYQQI-LAGDSAMW
VFPISVLLVFLVLAALYESLTLPLAVILIVPMSILSALTGVWLT-------------QGDNNIFTQIGLMVLVGLSAKNAILIVEFARELEH
------------DGRTPLEAAIEASRLRLRPILMTSIAFIMGVVPLVT-STGAGSEMRHAMGVAVFFGMLGVTLFGLI------------------
-----------------------------</seq-data>
</sequence>
<sequence seq-type="Protein">
<seq-name>q9zh24</seq-name>
<seq-info><length>1374</length></seq-info>
<seq-data>-----------------------------MARFFIDRPVFAWVISLLIVLAGVLAIRFLPVAQYPDIAPPVVNVSASYPGASAKVVE

EAVTAIIEREMNG-APGLLYTKATS-QHRQASLTLTFRQGVNADLAAVEVQNRLKIVESRLPESVR-RDGIYVEKAADSIQLIV
TLTSSSG------------------------------RYDAMELG-EIASSNVLQALRRVEGVGKVETWG-AEYAMRIWPDPAKLTSMNLSAS
DLVNAVRRHNARLTVGDIGNLGVPDSAPISATVKVDDTLVTPEQFGEIPLR--------------------------------------IRADGGAI
RLRDVARVEFG-----------QSEYGFVSRVNQM-----TATGLAVKMAPGSNAVATAKHPRHP-RRVS--RYFPEGVSYNIPYDTS
AFVEISIRKVVSTLLEAMLLVFAVMYLFMQNFRATLIPTLVVPVALLGTFTVMLGLGFSINVLTMFGMVLAIGILVDDAIIVVE
NVERLMA------------EEGLSPHDATVKPMRQISGAIVGITVVLVSVFVPMAFFS---GAVGNIYRQ--FAVTLAVSIGFSAFLAL
SLTPALCATLLRPIDAD--HHEKR-GFFGWFNRAFLRLTG
RYRNAVAGILARPIRWMLVYTLVIGVVALLF-VRLPQAFLPEEDQGDFMIMVMQPEGTPMAETMANVGDVERYLAE---H
EPVAYAYAVGGFSLYG-----DGTSSAMIFATLKDWSERRE------------ASQHVGAIVERINQRFAG----------------LPNRTVY
AMNSPPLPDLGSTSGFDFRLQD-RGGVGYEALVKARDQLLARA-AEDP-RLAN----VMFAGQGEAPKIRLDIDRRKAETLGV
SMDEINTTLAVMF---GSDYIGDFMHG--SQVRKVVVQADGAKRLGIDDIGRLHVRNEQGE-----MVPLATFAKAAWTLGP
PQLTRYNGYPSFNLEGQA--APGYSSGEAMQAMEQLMQG----------------LPEGIAHEWSGQSFEER-LSGAQAPALFALS
VLIVFLALAALYESWSIPLAVILVVPLGVLGALLGVSLR------------GLPNDIYFKVGLITIIGLSAKNAILIIEVAKDHYQ----------
EGMSLLQATLEAARLRLRPIVMTSLAFGFGVVPLAL-SSGAGSGAQVAIGTGVLGGIVTATVLAVFLVPLFFLVVGRLFRLRK
APRTGNSPQIPTEQA------------</seq-data>
</sequence>
<sequence seq-type="Protein">
<seq-name>q9zng8</seq-name>
<seq-info><length>1374</length></seq-info>
<seq-data>---------------------------MARFFIDRPVFAWVISLLIVLAGVLAIRFLPVAQYPDIAPPVVNVSATYPGASAKVV
EEAVTAIIEREMNG-APGLLYTKATS-STGQASLTLTFRQGVNANLAAVEVQNRLKIVESRLPESVR-RDGIYVEKAADSIQLIV
TLTSSSG------------------------------RYDAMELG-EIASSNVLQALRRVEGVGKVETWG-AEYAMRIWPDPAKLTSMNLSAS
DLVNAVRRHNARLTVGDIGNLGVPDSAPISATVKVDDTLVTPEQFGEIPLA--------------------------------------HPRDGGA
IRLRDVARVEFG-----------QSEYGFVSRVNQM-----TATGLAVKMAPGSNAVATAKRIRATLDELS--RYFPEGVSYNIPYDTS
AFVEISIRKVVSTLLEAMLLVFAVMYLFMQNFRATLIPTLVVPVALLGTFTVMLGLGFSINVLTMFGMVLAIGILVDDAIIVVE
NVERLMA------------EEGLSPHDATVKAMRQISGAIVGITVVLVSVFVPMAFFS---GAVGNIYRQ--FAVTLAVSIGFSAFLAL
SLTPALCATLLRPIDAD--HHEKR-GFFGWFNRAFLRLTG
RYRNAVAGILARPIRWMLVYTLVIGVVALLF-VRLPQAFLPEEDQGDFMIMVMQPEGTPMAETMANVGDVERYLAE---H
EPVAYAYAVGGFSLYG-----DGTSSAMIFATLKDWSERRE------------ASQHVGAIVERINQRFAG----------------LPNRTVY
AMNSPPLPDLGSTSGFDFRLQD-RGGVGYEALVKARDQLLARA-AEDP-RLAN----VMFAGQGEAPQIRLDIDRRKAETLG
VSMDEINTTLAVMF---GSDYIGDFMHG--SQVRKVVVQADRRKRLGIDDIGRLHVRNEQGE-----MGAAGDVRQGRLDPR
PAATDPLQRLSLVQPRGPG--RAGLQQREAMQAMEQLMQG----------------TARGIRPRVVRPVLRRTPVAGAQAPALFA
LSVLIVFLALAALYESWSIPLAVILVVPLGVLGALLGVSLR------------GLPNDIYFKVGLITIIGLSAKNAILIIEVAKDHYQ--------
---EGMSLLQATLEAARLRLRPIVMTSLAFGFGVVPLAL-SSGAGIRAQVAIGTGVLGGIVTATVLAVFLVPLFFLVVGRLFRLR
KAPRTGNSPQIPTEQA------------</seq-data>
</sequence>
<sequence seq-type="Protein">
<seq-name>o87936</seq-name>
<seq-info><length>1374</length></seq-info>
<seq-data>---------------------------MARFFIDRPVFAWVISLFIMLGGIFAIRALPVAQYPDIAPPVVSLYATYPGASAQVV
EESVTAVIEREMNG-VPGLLYTSATS-SAGQASLSLTFKQGVSADLAAVDVQNRLKIVEARLPEPVR-RDGISIEKAADNAQII
VSLTSEDG------------------------------RISGVELG-EYASANVLQALRRVEGVGKVQFWG-AEYAMRIWPDPVKMAALGLT

ASDIASAVRAHNARVTIGDVGRSAVPDSAPIAATVLADAPLTTPDAFGAIALR--------------------------------------ARADGS
TLYLRDVARIEFG-----------GNDYNYPSFVNGK-----TATGMGIKLAPGSNAVATEKRVRATMEELA--KFFPPGVKYQIPYET
ASFVRVSMSKVVTTLVEAGVLVFAVMFLFMQNFRATLIPTLVVPVALLGTFGAMLAAGFSINVLTMFGMVLAIGILVDDAI
VVVENVERLMV------------EEKLPPYEATVKAMKQISGAIVGITVVLTSVFVPMAFFG---GAVGNIYRQ--FAFALAVSIGFSA
FLALSLTPALCATLLKPVADD---HHEKD-GFFGWFNRFV
ARSTHRYTRRVGRVLERPLRWLVVYGALTAAAALLI-TKLPAAFLPDEDQGNFMVMVIRPQGTPLAETMQSVRRVEEYVR
T---HSPSAYTFALGGYNLYG-----EGPNGGMIFVTMKDWKERKR-----------ARDQVQAIIAEINAHFAG----------------TP
NTMVFAINMPALPDLGLTGGFDFRLQD-RGGLGYGAFVAAREKLLAEG-RKDP-VLTD----LMFAGTQDAPQLKLDIDRAK
ASALGVSMEEINATLAVMF---GSDYIGDFMHG--SQVRRVIVQADGRHRLDAADVTKLRVRNAKGE-----MVPLAAFATLH
WTMGPPQLTRYNGFPSFTINGAA--SAGHSSGEAMAAIERIAST-----------------LPAGTGYAWSGQSYEER-LSGAQAPML
FALSVLVVFLALAALYESWSIPFAVMLVVPLGVIGAVAGVTLR-----------GMPNDIYFKVGLIATIGLSAKNAILIVEVAKDLV
A-----------QRMSLADAALEAARLRLRPIVMTSLAFGVGVLPLAF-ATGAASGAQIAIGTGVLGGVISATLFAIFLVPLFFVCVG
RVFDVVPRRPGGAQAALEAK-------------</seq-data>
</sequence>
<sequence seq-type="Protein">
<seq-name>q51396</seq-name>
<seq-info><length>1374</length></seq-info>
<seq-data>----------------------------MSEFFIKRPNFAWVVALFISLGGLLVISKLPVAQYPNVAPPQITITATYPGASAKVLV
DSVTSVLEESLNG-AKGLLYFESTNNSNGTAEIVVTFEPGTDPDLAQVDVQNRLKKAEARMPQAVL-TQGLQVEQTSAGFL
LIYALSYKEGAQ----------------------------RSDTTALG-DYAARNINNELRRLPGVGKLQFFS-SEAAMRVWIDPQKLVGFGLS
IDDVSNAIRGQNVQVPAGAFGSAPGSSAQELTATLAVKGTLDDPQEFGQVVLR--------------------------------------ANED
GSLVPARRCRAPGTR-----------QGELQHFLATERH-----AHRGRGYPAVARGQRDPDPTLVKQRLAELS--AFFPEDMQYSV
PYDTSRFVDVAIEKVIHTLIEAMVLVFLVMFLFLENVRYTLIPSIVVPVCLLGTLMVMYLLGFSVNMMTMFGMVLAIGILVD
DAIVVVENVERIMA------------EEGISPAEATVKAMKQVSGAIVGITLVLSAVFLPLAFMA---GSVGVIYQQ--FSVSLAVSILF
SGFLALTFTPALCATLFKPIPEG---HHEKR-GFFGAFNRGF
ARVTERYSLLNSKLVARAGRFMLVYAGLVAMLGYFY-LRLPEAFVPAEDLGYMVVDVQLPPGASRVRTDATGEELERFLKS-
--REAVASVFLISGFSFSG-----QGDNAALAFPTFKDWSERG------------AEQSSAAEIAALNEHFAL----------------PDDGTV
MAVSPPPINGLGNSGGFALRLMD-RSGVGREALLQARDTLLGEI-QTNP-KFLY----AMMEGLAEAPQLRLLIDREKARALG
VSFETISGTLSAAF---GSEVINDFTNA--GRQQRVVIQAEQGNRMTPESVLELYVPNAAGN-----LVPLSAFVSVKWEEGPVQ
LVRYNGYPSIRIVGDA--APGFSTGEAMAEMERLASQ-----------------LPAGIGYEWTGLSYQEK-VSAGQATSLFALAILVVF
LLLVALYESWSIPLSVMLIVPIGAIGAVLAVMVS-----------GMSNDVYFKVGLITIIGLSAKNAILIVEFAKELWE-----------QG
HSLRDAAIEAARLRFRPIIMTSMAFILGVIPLAL-ASGAGAASQRAIGTGVIGGMLSATFLGVLFVPICFVWLLSLLRSKPAPIE
QAASAGE---------------</seq-data>
</sequence>
<sequence seq-type="Protein">
<seq-name>q51073</seq-name>
<seq-info><length>1374</length></seq-info>
<seq-data>----------------------------MAKFFIDRPIFAWVISIFIIAAGIFGIKSLPVSQYPSVAAPTITLHAIYPGASAQVMEG
SVLSVIERNMNG-VEGLDYMSTSADSSGSGSVSLTFTPDTDENLAQVEVQNKLSEVLRTLPATVQ-QYGVTVSKARSNFLMI
VMLSSDVQ--------------------------------STEEMN-DYAQRNVVPELQRIEGVGQVRLFG-AQRAMRIWVDPKKLQNYNLS
FADVGSALSAQNIQISAGSIGSLPAVRGQTVTATVTAQGQLGTAEEFGNVILR--------------------------------------ANTDG
SNIYLKDVAKVGLG-----------MEDYSSSTRLNGV-----NTTGMAVMLSNSGNAMATAKAVKERLAVLE--KYFPQGMSWKT

PYDTSKFVEISIEKVIHTLIEAMVLVFVVMYLFLQNIRYTLIPTIVVPISLLGGFAFISYMGMSINVLTMFAMILVIGIVVDDAIV
VVENVERIMA------------GEGLPPKEATKKAMGQISGAVIGITAVLISVFVPLAMFS---GAAGNIYKQ--FALTMASSIAFSAFL
ALTLTPALCATMLKTIPKG--HHEEKKGFFGWFNKKFDS
WTHGYEGRVAKVLRKTFRMMVVYIGLAVVGVFLF-MRLPTSFLPTEDQGFVMVSVQLPAGATKERTDATLAQVTQLAKS-
--IPEIENIITVSGFSFSG-----SGQNMAMGFAIFKDWNERTA------------SGSDAVAVAGKLTGMMMG----------------TLKD
GFGIAVVPPPILELGNGSGLSINLQD-RNNTGHTGIAGEGNELIQKM-RASG-LFDPST--VRAGGLEDSPQLKIDINRAAAAA
QGISFADIRTALASAL---SSSYVSDFPNQ--GRLQRVMVQADEDARMQPADILNLTVPNKSGV-----AVPLSTIATVSWENG
TEQSVRFNGYPSMKLSASP--ATGVSTGQAMEAVQKMVDE-----------------LGGGYSFEWGGQSSEEA-KGGSQTLILYGL
AVAAVFLVLAALYESWSIPLAVILVIPLGLIGAAAGVTGRNLFEGLLGSVP-SFANDIYFQVGFVTVMGLSAKNAILIIEFAKDL
QA-----------QGKSAVEAALEAARLRFRPIIMTSFAFILGVVPLYI-AAGASSASQRAIGTTVFWGMLVGTLLSVFLVPLFYVVV
RKFFKETAHEHEMAVRHASKAGITGSDDKQY----</seq-data>
</sequence>
<sequence seq-type="Protein">
<seq-name>ACRF_ECOLI</seq-name>
<seq-info><length>1374</length></seq-info>
<seq-data>----------------------------MANFFIRRPIFAWVLAIILMMAGALAILQLPVAQYPTIAPPAVSVSANYPGADAQT
VQDTVTQVIEQNMNG-IDNLMYMSSTSDSAGSVTITLTFQSGTDPDIAQVQVQNKLQLATPLLPQEVQ-QQGISVEKSSSS
YLMVAGFVSDNP------------------------------GTTQDDIS-DYVASNVKDTLSRLNGVGDVQLFG-AQYAMRIWLDADLLNK
YKLTPVDVINQLKVQNDQIAAGQLGGTPALPGQQLNASIIAQTRFKNPEEFGKVTLR---------------------------------------V
NSDGSVVRLKDVARVELG-----------GENYNVIARINGK-----PAAGLGIKLATGANALDTAKAIKAKLAELQ--PFFPQGMKVL
YPYDTTPFVQLSIHEVVKTLFEAIMLVFLVMYLFLQNMRATLIPTIAVPVVLLGTFAILAAFGYSINTLTMFGMVLAIGLLVDD
AIVVVENVERVMM------------EDKLPPKEATEKSMSQIQGALVGIAMVLSAVFIPMAFFG---GSTGAIYRQ--FSITIVSAMA
LSVLVALILTPALCATLLKPVSA---EHHENKGGFFGWFNT
TFDHSVNHYTNSVGKILGSTGRYLLIYALIVAGMVVLF-LRLPSSFLPEEDQGVFLTMIQLPAGATQERTQKVLDQVTDYYLK
NE-KANVESVFTVNGFSFSG-----QAQNAGMAFVSLKPWEERNG------------DENSAEAVIHRAKMELGK-----------------IR
DGFVIPFNMPAIVELGTATGFDFELID-QAGLGHDALTQARNQLLGMA-AQHPASLVS----VRPNGLEDTAQFKLEVDQEK
AQALGVSLSDINQTISTAL---GGTYVNDFIDR--GRVKKLYVQADAKFRMLPEDVDKLYVRSANGE-----MVPFSAFTTSHW
VYGSPRLERYNGLPSMEIQGEA--APGTSSGDAMALMENLASK-----------------LPAGIGYDWTGMSYQER-LSGNQAPAL
VAISFVVVFLCLAALYESWSIPVSVMLVVPLGIVGVLLAATLF------------NQKNDVYFMVGLLTTIGLSAKNAILIVEFAKDLM
EK----------EGKGVVEATLMAVRMRLRPILMTSLAFILGVLPLAI-SNGAGSGAQNAVGIGVMGGMVSATLLAIFFVPVFFV
VIRRCFKG----------------------------</seq-data>
</sequence>
<sequence seq-type="Protein">
<seq-name>acrb_ecoli</seq-name>
<seq-info><length>1374</length></seq-info>
<seq-data>----------------------------MPNFFIDRPIFAWVIAIIIMLAGGLAILKLPVAQYPTIAPPAVTISASYPGADAKTVQ
DTVTQVIEQNMNG-IDNLMYMSSNSDSTGTVQITLTFESGTDADIAQVQVQNKLQLAMPLLPQEVQ-QQGVSVEKSSSSF
LMVVGVINTDG------------------------------TMTQEDIS-DYVAANMKDAISRTSGVGDVQLFG-SQYAMRIWMNPNELNK
FQLTPVDVITAIKAQNAQVAAGQLGGTPPVKGQQLNASIIAQTRLTSTEEFGKILLK---------------------------------------VN
QDGSRVLLRDVAKIELG-----------GENYDIIAEFNGQ-----PASGLGIKLATGANALDTAAAIRAELAKME--PFFPSGLKIVYPY
DTTPFVKISIHEVVKTLVEAIILVFLVMYLFLQNFRATLIPTIAVPVVLLGTFAVLAAFGFSINTLTMFGMVLAIGLLVDDAIVVV
ENVERVMA------------EEGLPPKEATRKSMGQIQGALVGIAMVLSAVFVPMAFFG---GSTGAIYRQ--FSITIVSAMALSVLV

ALILTPALCATMLKPIAKG---DHGEGKKGFFGWFNRMFE
KSTHHYTDSVGGILRSTGRYLVLYLIIVVGMAYLF-VRLPSSFLPDEDQGVFMTMVQLPAGATQERTQKVLNEVTHYYLTKE-
KNNVESVFAVNGFGFAG-----RGQNTGIAFVSLKDWADRPG------------EENKVEAITMRATRAFSQ-----------------IKDA
MVFAFNLPAIVELGTATGFDFELID-QAGLGHEKLTQARNQLLAEA-AKHPDMLTS----VRPNGLEDTPQFKIDIDQEKAQA
LGVSINDINTTLGAAW---GGSYVNDFIDR--GRVKKVYVMSEAKYRMLPDDIGDWYVRAADGQ-----MVPFSAFSSSRWE
YGSPRLERYNGLPSMEILGQA--APGKSTGEAMELMEQLASK-----------------LPTGVGYDWTGMSYQER-LSGNQAPSLY
AISLIVVFLCLAALYESWSIPFSVMLVVPLGVIGALLAATFR------------GLTNDVYFQVGLLTTIGLSAKNAILIVEFAKDLMDK
----------EGKGLIEATLDAVRMRLRPILMTSLAFILGVMPLVI-STGAGSGAQNAVGTGVMGGMVTATVLAIFFVPVFFVVV
RRRFSRKNEDIEHSHTVDHH---------------</seq-data>
</sequence>
<sequence seq-type="Protein">
<seq-name>YHIV_ECOLI</seq-name>
<seq-info><length>1374</length></seq-info>
<seq-data>-----------------------------MANYFIDRPVFAWVLAIIMMLAGGLAIMNLPVAQYPQIAPPTITVSATYPGADAQ
TVEDSVTQVIEQNMNG-LDGLMYMSSTSDAAGNASITLTFETGTSPDIAQVQVQNKLQLAMPSLPEAVQ-QQGISVDKSS
SNILMVAAFISDNG-------------------------------SLNQYDIA-DYVASNIKDPLSRTAGVGSVQLFG-SEYAMRIWLDPQKLNK
YNLVPSDVISQIKVQNNQISGGQLGGMPQAADQQLNASIIVQTRLQTPEEFGKILLK---V
QQDGSQVLLRDVARVELG-----------AEDYSTVARYNGK-----PAAGIAIKLAAGANALDTSRAVKEELNRLS--AYFPASLKTV
YPYDTTPFIEISIQEVFKTLVEAIILVFLVMYLFLQNFRATIIPTIAVPVVILGTFAILSAVGFTINTLTMFGMVLAIGLLVDDAIVV
VENVERVIA------------EDKLPPKEATHKSMGQIQRALVGIAVVLSAVFMPMAFMS---GATGEIYRQ--FSITLISSMLLSVFV
AMSLTPALCATILKAAP--EGGHKPNALFARFNTLFEKS
TQHYTDSTRSLLRCTGRYMVVYLLICAGMAVLF-LRTPTSFLPEEDQGVFMTTAQLPSGATMVNTTKVLQQVTDYYLTKE-K
DNVQSVFTVGGFGFSG-----QGQNNGLAFISLKPWSERVG------------EENSVTAIIQRAMIALSS-----------------INKAVVFP
FNLPAVAELGTASGFDMELLD-NGNLGHEKLTQARNELLSLA-AQSPNQVTG----VRPNGLEDTPMFKVNVNAAKAEAM
GVALSDINQTISTAF---GSSYVNDFLNQ--GRVKKVYVQAGTPFRMLPDNINQWYVRNASGT-----MAPLSAYSSTEWTYG
SPRLERYNGIPSMEILGEA--AAGKSTGDAMKFMADLVAK-----------------LPAGVGYSWTGLSYQEA-LSSNQAPALYAISL
VVVFLALAALYESWSIPFSVMLVVPLGVVGALLATDLR------------GLSNDVYFQVGLLTTIGLSAKNAILIVEFAVEMMQK--
--------EGKTPIEAIIEAARMRLRPILMTSLAFILGVLPLVI-SHGAGSGAQNAVGTGVMGGMFAATVLAIYFVPVFFVVVEHL
FARFKKA--------------------------</seq-data>
</sequence>
<sequence seq-type="Protein">
<seq-name>ACRD_ECOLI</seq-name>
<seq-info><length>1374</length></seq-info>
<seq-data>-----------------------------MANFFIDRPIFAWVLAILLCLTGTLAIFSLPVEQYPDLAPPNVRVTANYPGASAQTLE
NTVTQVIEQNMTG-LDNLMYMSSQSSGTGQASVTLSFKAGTDPDEAVQQVQNQLQSAMRKLPQAVQ-NQGVTVRKTG
DTNILTIAFVSTDG-------------------------------SMDKQDIA-DYVASNIQDPLSRVNGVGDIDAYG-SQYSMRIWLDPAKLNS
FQMTAKDVTDAIESQNAQIAVGQLGGTPSVDKQALNATINAQSLLQTPEQFRDITLR---V
NQDGSEVRLGDVATVEMG-----------AEKYDYLSRFNGK-----PASGLGVKLASGANEMATAELVLNRLDELA--QYFPHGLE
YKVAYETTSFVKASIEDVVKTLLEAIALVFLVMYLFLQNFRATLIPTIAVPVVLMGTFSVLYAFGYSVNTLTMFAMVLAIGLLV
DDAIVVVENVERIMS------------EEGLTPREATRKSMGQIQGALVGIAMVLSAVFVPMAFFG---GTTGAIYRQ--FSITIVAA
MVLSVLVAMILTPALCATLLKPLKKG---EHHGQK-GFFA
WFNQMFNRNAERYEKGVAKILHRSLRWIVIYVLLLGGMVFLF-LRLPTSFLPLEDRGMFTTSVQLPSGSTQQQTLKVVEQIE

KYYFTHE-KDNIMSVFATVGSGPGG-----NGQNVARMFIRLKDWSERDS------------KTGTSFAIIERATKAFNQ-------------
---IKEARVIASSPPAISGLGSSAGFDMELQD-HAGAGHDALMAARNQLLALA-AENP-ELTR----VRHNGLDDSPQLQIDIDQ
RKAQALGVAIDDINDTLQTAW---GSSYVNDFMDR--GRVKKVYVQAAAPYRMLPDDINLWYVRNKDGG-----MVPFSAF
ATSRWETGSPRLERYNGYSAVEIVGEA--APGVSTGTAMDIMESLVKQ-----------------LPNGFGLEWTAMSYQER-LSGAQ
APALYAISLLVVFLCLAALYESWSVPFSVMLVVPLGVIGALLATWMR------------GLENDVYFQVGLLTVIGLSAKNAILIVEF
ANEMNQ-----------KGHDLFEATLHACRQRLRPILMTSLAFIFGVLPMAT-STGAGSGGQHAVGTGVMGGMISATILAIYFV
PLFFVLVRRRFPLKPRPE-----------------------</seq-data>
</sequence>
<sequence seq-type="Protein">
<seq-name>MEXB_PSEAE</seq-name>
<seq-info><length>1374</length></seq-info>
<seq-data>----------------------------MSKFFIDRPIFAWVIALVIMLAGGLSILSLPVNQYPAIAPPAIAVQVSYPGASAETVQ
DTVVQVIEQQMNG-IDNLRYISSESNSDGSMTTTVTFEQGTDPDIAQVQVQNKLQLATPLLPQEVQ-RQGIRVTKAVKNFL
MVVGVVSTDG------------------------------SMTKEDLS-NYIVSNIQDPLSRTKGVGDFQVFG-SQYSMRIWLDPAKLNSYQL
TPGDVSSAIQAQNVQISSGQLGGLPAVKGQQLNATIIGKTRLQTAEQFENILLK---------------------------------------VNPD
GSQVRLKDVADVGLG-----------GQDYSINAQFNGS-----PASGIAIKLATGANALDTAKAIRQTIANLE--PFMPQGMKVVYP
YDTTPVVSASIHEVVKTLGEAILLVFLVMYLFLQNFRATLIPTIAVPVVLLGTFGVLAAFGFSINTLTMFGMVLAIGLLVDDAIV
VVENVERVMA------------EEGLSPREAARKSMGQIQGALVGIAMVLSAVFLPMAFFG---GSTGVIYRQ--FSITIVSAMALSV
IVALILTPALCATMLKPIEKG--DHGEHKGGFFGWFNRM
FLSTTHGYERGVASILKHRAPYLLIYVVIVAGMIWMF-TRIPTAFLPDEDQGVLFAQVQTPPGSSAERTQVVVDSMREYLLE
KE-SSSVSSVFTVTGFNFAG-----RGQSSGMAFIMLKPWEERPG------------GENSVFELAKRAQMHFFS-----------------FKD
AMVFAFAPPSVLELGNATGFDLFLQD-QAGVGHEVLLQARNKFLMLA-AQNP-ALQR----VRPNGMSDEPQYKLEIDDEK
ASALGVSLADINSTVSIAW---GSSYVNDFIDR--GRVKRVYLQGRPDARMNPDDLSKWYVRNDKGE-----MVPFNAFATGK
WEYGSPKLERYNGVPAMEILGEP--APGLSSGDAMAAVEEIVKQ-----------------LPKGVGYSWTGLSYEER-LSGSQAPALY
ALSLLVVFLCLAALYESWSIPFSVMLVVPLGVIGALLATSMR------------GLSNDVFFQVGLLTTIGLSAKNAILIVEFAKELHE--
---------QGKGIVEAAIEACRMRLRPIVMTSLAFILGVVPLAI-STGAGSGSQHAIGTGVIGGMVTATVLAIFWVPLFYVAVSTL
FKDEASKQQASVEKGQ----------------</seq-data>
</sequence>
<sequence seq-type="Protein">
<seq-name>o52248</seq-name>
<seq-info><length>1374</length></seq-info>
<seq-data>----------------------------MSKFFIDRPIFAWVIALVIMLVGALSILKLPINQYPSIAPPAIAIAVTYPGASAQTVQD
TVVQVIEQQLNG-IDNLRYVSSESNSDGSMTITATFEQGTNPDTAQVQVQNKLNLATPLLPQEVQ-QQGIRVTKAVKNFLL
VIGLVSEDG------------------------------SMTKDDLA-NYIVSNMQDPISRTAGVGDFQVFG-AQYAMRIWLDPAKLNKFQL
TPVDVKTAVAAQNVQVSSGQLGGLPALPGTQLNATIIGKTRLQTAEQFESILLK---------------------------------------VNKD
GSQVRLGDVAQVGLG-----------GENYAVSAQFNGK-----PASGLAVKLATGANALDTAKALRETIKGLE--PFFPPGVKAVFP
YDTTPVVTESISGVIHTLIEAVVLVFLVMYLFLQNFRATIITTMTVPVVLLGTFGILAAAGFSINTLTMFAMVLAIGLLVDDAIV
VVENVERVMS------------EEGLPPKEATKRSMEQIQGALVGIALVLSAVLLPMAFFG---GSTGVIYRQ--FSITIVSAMGLSVL
VALIFTPALCATMLKPLKKG--EHHTAKGGFFGWFNRNF
DRSVNGYERSVGTILRNKVPFLLAYALIVVGMIWLF-ARIPTAFLPEEDQGVLFAQVQTPAGSSAERTQVVVDQMREYLLKD
E-ADTVSSVFTVNGFNFAG-----RGQSSGMAFIMLKPWDERS-------------KENSVFALAQRAQQHFFT-----------------FRD
AMVFAFAPPAVLELGNATGFDVFLQD-RGGVGHAKLMEARNQFLAKA-AQSK-ILSA----VRPNGLNDEPQYQLTIDDERA

SALGVTIADINNTLSIAL---GASYVNDFIDR--GRVKKVYIQGEPSARMSPEDLQKWYVRNGAGE-----MVPFSSFAKGEWTY
GSPKLSRYNGVEAMEILGAP--APGYSTGEAMAEVERIAGE-----------------LPSGIGFSWTGMSYEEK-LSGSQMPALFALS
VLFVFLCLAALYESWSIPIAVVLVVPLGIIGALIATSLR------------GLSNDVYFLVGLLTTIGLAAKNAILIVEFAKELHE-----------
QGRSLYDAAIEACRMRLRPIIMTSLAFILGVVPLTI-ASGAGAGSQHAIGTGVIGGMISATVLAIFWVPLFFVAVSSLFGSKEP
EKDVTPENPRYEAGQ-----------</seq-data>
</sequence>
<sequence seq-type="Protein">
<seq-name>o31100</seq-name>
<seq-info><length>1374</length></seq-info>
<seq-data>----------------------------MSRFFIDRPIFAWVLAIVAMLAGALSLAKMPISQYPNIAAPAVSIQVSYPGASAQTV
QDTVVQVIEQQLSG-LDGFRYMSAESASDGSMTIIVTFEQGTDPDIAQVQVQNKLQLATPRLPEEVQ-RQGLRVVKYQMN
FFLVMSLVDRSG-------------------------------KLDNFDLG-NLIASQLQDPISRIPGVGDFQLFG-SPYAMRIWLDPGKLNSYQ
LTPTDVASAIREQNVQVSSGQLGGLPTRSGVQLNATVLGKTRMTTPSQFDEILVK---------------------------------------VNP
DGSQVRVKDVGRAELG-----------ADSFAISAQYKDS-----PTASLALRLSTGGNLLETVDAVKKLMEQQK--AYLPDGVEVIYP
YDTTPVVEASIESVVHTIFEAVVLVFLVMYLFLQSFRATLIPTLAVPVVLLATFALLPYFGLNINVLTMYAMVLAIGLLVDDAIV
VVENVERLMH------------DEGLSPLEATRKSMDQISGALVGIGMVLSAVFVPMAFFG---GSAGIIYQQ--FAITIVVCMGLSIL
VALVFTPALCVTILKAPEGN---SHHERK-GFFGWFNRIFD
RGTRRFERGVGAMLKGRGRYLLAFLLITGGTGYLF-TQIPKAFLPNEDQGLMMIEVRTPANASAERTEGVLQEVRDYLAND
E-GALVEHFMTVNGFNFAG-----RGQNSGLVLITFKDWKERHG------------AGQDVFSIAQRANQHFAK-----------------IKD
ASVMAFVPPAILEMGNAMGFNLYLQD-NLGLGHEALMAARNQFLQLA-SQNP-KLQA----VRPNGKDDEPQFQVNIDDE
KARALQVSIASINETMSAAW---GSMYVNDFIDR--GRVKRVYVQGEDISRISPEDFDKWYVRNSLGQ-----MVPFSAFATGE
WVNGSPKLERYGGISSLNILGEP--APGYSTGDAMIAIAEIMQQ-----------------LPAGIGLSYTGLSYEEI-QTGDQAPLLYAL
TVLIVFLCLAALYESWSVPVSVIMVVPLGILGAVLATLWR------------DLTADVYFQVGLMTTVGLSAKNAILIVEFAKELYEK-
---------EGYPIVKAAIEAAKLRLRPILMTSLAFTFGVLPMAI-ASGAGAGSQHSIATGVVGGMITATVLAVFFVPLFYVVVVKL
FEGLMKRKPNAVKEVTHEV-------------</seq-data>
</sequence>
<sequence seq-type="Protein">
<seq-name>Q9RBY8</seq-name>
<seq-info><length>1374</length></seq-info>
<seq-data>----------------------------MVRFFIDRPIFAWVIAIAVSLLGLLAILILPVDRYPQIAPPTITIRATYTGASSQTVENA
VTQVIEQSQQS-LDHLMYMTSTSASDGSAQVNLVFATGTNPDTAQVQVQNQLQAAMATLPQAVQ-QNGLTITKSSGSIF
EVLSFTSEDG-------------------------------SMDNFDVA-NFMEARIDDQISRVSGVGNIQPIG-QEYAMRIWLDPEKMRQYA
LMPWDIETALQAQNTDVSAGELGGQPALKGQQLDATVTARSRLHTPEQFAQVVLK---------------------------------------A
DANGSVVRLGDVAKIGLG-----------PESYDSISTFNGK-----PSASLGIELNAGANAIAVSKAIDARLQQLQ--KYWPHGYTAH
VAFTTTPFVTISLKEVVITLIEAIILVVLVMYLFLQNWRATLIPTIAVPVVLLGTFGVLAAFGYSINTLTMFALVLAIGLLVDDAIV
VVENVERVMT------------FEGLAPKPATLKAMGQITGALVGIVLVLTAVFLPMAFFS---GVTGVIYRQ--FSVTIAAAMILSVL
VAMTITPALCGSILHQIPKGGHPHGDHG--GEPSLLG-KFFIWFN
HRFERTSNGLRHRVDRFLGRRTLGVLFYLVLSVATGLLL-WHLPGAFLPDEDQGMLNALVKLPAGSTLEQTRAVMDRLSAV
AVK---DDGVLSIQATAGFSVTG-----SGQNVGQAFIRLKDWDDRK---------------DDADTIAARLTRAMAS-----------------V
PDAQVFITSPPAILGLGDAGGFTLELQD-EGGAGHAAAVAARNTLLKEA-AKDP-KLVN----VRYASLEDAPVYAVKVDDAK
AQAMGVNPQDVNDTLNAAL---GGDFVNNFIYK--GRIKKVFIQGTAEARMQPQDIERWSVRNQAGQ-----MVPLSSLIST
HWTSAPAAVQRYNGISAMEITGQP--APGVSSGEAMAEIARLADT-----------------LPEGFSHAWSDMAYQEQ-LSGNQA

PMLYAISLLFVFLCLAALYESWAVPFAVMLAVPVGMFGAVLMMNLR------------GLNNDVYFQVGLLTTIGLAAKNGILIVE
FARILEQ-----------QGKSTREAILQAVYLRLRPIVMTSLAFLMGVLPLVF-ATGAGSAARRSLGTGVAGGTVASMVLGMFFV
PLFYLLVRRLFPGRAPADATVPETSP----------------</seq-data>
</sequence>
<sequence seq-type="Protein">
<seq-name>o68441</seq-name>
<seq-info><length>1374</length></seq-info>
<seq-data>-----------------------------MARFFIDRPIFAWVIAIIMGLGVLSIMRLPISQYPSIAGPSVVIGATYPGASAETVAD
TVVQIIEKEMTG-LDGLRYIDSSTTSTGRATVTLTFNLGTDPDIAQVQVQNKLSQAEASLPSEVT-RQGVTVEKSTTGFLMVI
ALISDDG-------------------------------ARSAIDLA-DYLNSYMVEPVSRLKGVGKVEVFG-SEYAMRIWLDPQKLKYYGLSPTTV
IHAISAQNAQISAGSFGAMPAPEGQQLNATVTAQSLLKTPQDFERIVLR--------------------------------------ADTDGGLVL
LRDVARAELG-----------TENYEISSFYNGK-----PSSGVAIQLASGANALETAELVKAKMADLG--AFLPAGVSYVIPYDTTPFV
SLSIEAVIHTLIEAIVLVVFVMLIFLHNFRATLIPTLAVPVVLLGTFGIMAALGFSINTLTMLAMVLAIGLLVDDAIVVVENVERI
MR------------DEHLDPVAATKNRWEKSRARSWASPWWFPPSSCRWRFSA---VQQGEMYKQ--FSVTIVAAMALSVLVALI
FTPALCATMLKAHDHNAK---SGLASRFSNWFERNFSWL
TTRYGNVVRASAKRPLRMFLVYLLLVGAMVFLY-QKTPTSFLPDEDQGTLMTIIQTPPGSTAQNTEAVLGKVENYYMNAE-
AQNVESVFSVSWFLLAG-----QGQNMGMMFVKLKDWEERKG------------KENSAQAIAARAFGPLMG----------------GIK
EAIVVPLVPPAVTELGNSNGFTAFLQA-RSGQSHEQLLEARNMLLGLA-AQSP-KLMA----VRPNGVEDASQFELNIDWGK
AGAVGLSAADVGSFLTTVW---SSSYVNDFLYE--GRMKRVYVQGEPLARTGPEDLALWRVPNANGD-----FVDLSTIASQN
WVFGPQQVIRYDALPAMSIEGSA--APGFSSGEALAEIEALAAK-----------------LPPGFSLQWTGMSLEEK-DAGAGALPLY
GLALATMFLCLAALYESWTIPVAVLLAMPVGILGALLGAWIG------------GQSNGVYFQVGLLTVVGLTGKNGIMIVEFARE
RMAQ----------LGESAFEAVCQAAKLRFRPILMTSLAFGLGVIPLVI-STGAGAGARQAIGYATFFGTVTGTLLAIVFVPVFFVL
ISGIFSRRSVKAAEPKTA------------------</seq-data>
</sequence>
<sequence seq-type="Protein">
<seq-name>YBDE_ECOLI</seq-name>
<seq-info><length>1374</length></seq-info>
<seq-data>-------------------------MIEWIIRRSVANRFLVLMGALFLSIWGTWTIINTPVDALPDLSDVQVIIKTSYPGQAPQI
VENQVTYPLTTTMLS-VPGAKTVRGFS-QFGDSYVYVIFEDGTDPYWARSRVLEYLNQVQGKLPAGV--SAELGPDATGVG
WIYEYALVDRSG-------------------------------KHDLADLR-SLQDWFLKYELKTIPDVAEVASVGGVVKEYQVVIDPQRLAQY
GISLAEVKSALDASNQEAGGSSIEL------AEAEYMVRASGYLQTLDDFNHIVLK--------------------------------------ASENG
VPVYLRDVAKVQIG-----------PEMRRGIAELNGEG----EVAGGVVILRSGKNAREVIAAVKDKLETLK--SSLPEGVEIVTTYDR
SQLIDRAIDNLSGKLLEEFIVVAVVCALFLWHVRSALVAIISLPLGLCIAFIVMHFQGLNANIMSLGGIAIAVGAMVDAAIVMI
ENAHKRLEEWQHQHPDAT--LDNKTRWQVITDASVEVGPALFISLLIITLSFIPIFTLE---GQEGRLFGP--LAFTKTYAMAGAA
LLAIVVIPILMGYWIRGKI---PPESSNPLNRFLIRVY
HPLLLKVLHWPKTTLLVAALSVLTVLWPL-NKVGGEFLPQINEGDLLYMPSTLPGISAAEAASMLQKTDKLIMS---VPEVAR
VFGKTGKAETAT--DSAPLEMVETTIQLKPQEQWR-------------PGMTMDKIIEELDNTV------------------RLPGLANLWVPP
IRNRIDMLSTGIKSPIGIKVSGTVLADIDAMAEQIEEVA-RTVP-GVAS----ALAERLEGGRYINVEINREKAARYGMTVADVQ
LFVTSAV---GGAMVGETVEG--IARYPINLRYPQSWRDSPQALRQLPILTPMKQ-----QITLADVADIKVSTGPSMLKTENAR
PTSWIYIDA---RDRDMVSVVHDLQKAIAEKV--------------QLKPGTSVAFSGQFELLE-RANHKLKLMVPMTLMIIFVLLYLA
FRRVGEALLIISSVPFALVGGIWLLWWM------------GFHLSVATGTGFIALAGVAAEFGVVMLMYLRHAIEAVPSLNNPQTF
SEQKLDEALYHGAVLRVRPKAMTVAVIIAGLLPILW-GTGAGSEVMSRIAAPMIGGMITAPLLSLFIIPAAYKLMWLHRHRV

RK-------------------------</seq-data>
</sequence>
<sequence seq-type="Protein">
<seq-name>o67202</seq-name>
<seq-info><length>1374</length></seq-info>
<seq-data>--------------------------MERFTEILLRNRLPILFISIFLLFYGYYSLKKTPIDAIPDLSDVQVIIYSEWIGQIPQVIEDQL
TYPLVTNMMG-VPKVKTVRGYS-FPNFSLVFVIFEDGTDLYWARSRVLEKLSSIRSQLPQDA--RIELGPDATGVGWVYKYVL
VSK--------------------------------KRTLEELW-ALQNFYIKYALLSIPDVAEVASVGGFEKEYRILVKPEKLYQYGISLGELYRAIK
RTNVETGGKYVEI------NEKEYLIRAVGYVKNREEIEKTVVA--ERDGVPIRLEDVAKVVEV
-----------PAFRMGVADFNGMG----DTVAGIVVMRFGADAYKVIQRVKEKLEEVK--KGLPEDVQVLTAYDRSTLIESAIENLK
RKLIEESVVVLLIVGIFLFHVRSALVIIIFLVLSILTTFAVMNHLGITSNIMSLGGIAIAIGTMVDAAIVLVENIHRRL-------------E
EGEKLWDAIVHSAKDVGKPIFFALLIVTVSFVPLFALE---GQAGRLFEP--LVATKTLSMLIASIISVIVVPVLAYYFIRGRI--------
--PGEEKNPLVKLLIKVYDPVFHISIKLRYLLLILFIFMGVA
TFYLY-EKLGREFMPPLNEGTLMYMPTTVPSVSRQEMLRILNIQDRILKE---FPEVESVLGKAGRANTPT--DPAPMSMIETFI
TLKPQEEWRKVKVERFYSD-WEIPEFIKDILRKVFPEERPL--TYAELIREMDKAISLPGLSNMWTMPIKGRIDMITTGIQTPL
GIKVYGDDVNKLNEVAQEIEKLL-KDVP-GIMS----VFGERSANALYVEIIPKREELDRYGLTVGDVNEAILTLF---ANKKISTVIL
G--RERYSVTLGIPLDYRYELETLT-IPMKDK--------FVPLSAVADVVVKESPSSIKSENGLLVSYVFVTP--DPEVDMGTVIERA
EKVLKENL--------------KLPKGYYYEWSGQFEYWK-KALENLKVIVPSVILLIILLVWFTFNKLFETFLVLITLPIATFGGVLLMYL
L------------NYNISIASIAGFLALLGIAAEMGIVMVVYIQNALKESEIR------SKKDAFEAIYKGAVKRIRPKFMTFGAILIGLLPI
ML-GHGTGSEVMSRIAAPMVGGILSTIVIVLIIVPALYAIYVDFRRGNI---------------------------</seq-data>
</sequence>
<sequence seq-type="Protein">
<seq-name>q9zjq5</seq-name>
<seq-info><length>1374</length></seq-info>
<seq-data>-------------------------MIEKIIDLSVKNKLLTTLVTLLIFLASLWAIKSVRLDALPDLSPAQVVVQITYPNQSPKIVQ
EQVTYPLVSTFMS-IANIDTVRGIS-SYESGLIYIIFKDGVNLYWARDRVLEQLNRVS-NLPKDA--KVEIGSDSTSIGWAYQYAL
SSD--------------------------------SKNLSDLK-VLQDFYYRYALLGVDGVSEVASVGGFVKDYEVTLQNDSLIRYNLSLEQVAN
AIKNSNNDTGGGVILE------NGFEKIIRSHGYIQSLNDLEEIVVK--KEGAIPLKIKDIASVRL
A-----------PKPRRGAANLNGDK----EVVGGIVMVRYHADTYKVLKAIKEKIATLQ--ASNPD-VKITSVYDRSELIEKGIDNLIHT
LIEESVIVLVIIAIFLLHFRSALVVIITLPLSVCISFLLMRYFNIEASIMSLGGIAIAIGAMVDAAIVMVENAHKHLQHIDT--------R
DNTQRVNAIMQGVKHVGGAIFFALMIIVVSFLPIFALT---GQEEKLFAP--LAYTKTFAMLVGALLSITIVPVLMVWLIKGRI---
---LEESKNPINAFFMKIYGVSLKVVLKFRYAFLIASVL
GLGGLVVAY-KKLNWEFIPQINEGVIMYMPVTINGVGIDTALEYLKKSNAAIKQ---LDFVKQVFGKVGRANTST--DAAGLG
MIETYIELKPQNEWK-------------EKLSYKEVRDKLEKTLQ-----------------LKGLTNSWTYPIRGRTDMLLTGIRTPLGIKLYG
NDTDKLQELAILMEQQL-KTLK-ESLS----VFAERSNNGYYITLDLNDENLARYGINKNAVLDTIKFAL---GGATLTTMIKG--V
ESYPISLRLEDTERNTIEKLKNLYIKTAYN------YMPLRELAHVYYDNSPAVLKSEKGLNVNFIYIVP--QNGISSDTYRQLAQKT
LEKI---------------QLPSGYYYEFSGESQYLE-EAFKTLQYIVPVSVFIIFILIVFALKNLTNSLLCFFTLPFAFLGGLIFMNLM-------
-----GFNMSVAALVGFLALLGVASETAIVMIIYLEDAFQKFIKTPLKER-TTAALKETIMHGAVLRVRPKLMTFFSILASLIPIMY-
SHGTGSEIMKSIAAPMLGGMMSSVVLTLFIIPTAYFVIKNARIRKREY-------------------------</seq-data>
</sequence>
<sequence seq-type="Protein">
<seq-name>o25622</seq-name>

<seq-info><length>1374</length></seq-info>
<seq-data>------------------------MMLASIIEFSLRQRVIVIVGAILILFFGTYSFINTPVDAFPDISPTQVKIILKLPGSSPEEMEN
NIVRPLELELLG-LKGQKSLRSVS-KYSISDITIDFDDSVDIYLARNIVNERLSSVMKDLPVGV--EGGMAPIVTPLSDIFMFTIDG
----------------------------------NITEIEKR-QLLDFVIRPQLRMISGVADVNSIGGFSRAFVIVPDFNDMARLGVSISDLESAVRV
NLRNSGAGRVDR------DGETFLVKIQTASLSLEDIGKITVS--TNLG-HLHIKDFAKVISQ---
--------SRTRLGFVTKDGVG----ETTEGLVLSLKDANTKEIITQVYQKLEELK--PFLPNGVSINVFYDRSEFTQKAIATVSKTLIEA
VVLIIITLFLFLGNLRASVAVGVILPLSLSVAFIFIKFSDLTLNLMSLGGLVIAIGMLIDSAVVVVENAFEKLSA-----------NTKTTK
LHAIYRSCKEIAVSVVSGVVIIIVFFVPILTLQ---GLEGKMFRP--LAQSIVYALLGTLVLSITIIPVVSSLVLKA-----------------------
---TPHSETFLTRFLNRIYAPLLEFFVHNPKKVILGAFVFLIASLSLF-PF
VGKNFMPVLDEGDVVLSVETTPSISLDQSRDLMLNIESAIKKH--VKEVKSIVARTGSDELGL--DLGGLNQTDTFISFIPKKEW
S-------------VKTKDELLEKIMDSLKD-----------------FKGINFSFTQPIEMRISEMLTGVRGDLAVKIFGDGISELNELSFQIAQ
AL-KGIK-GSSE----VLTTLNEGVNYLYVTPNKESMADVGITSDEFSKFLKSAL---EGLVVDVIPTG--ISRTPVMIRQESDFASSI
TKIKSLALTSKYGV-----LVPITSIAKIEEVDGPVSVVRENSMRMSVVRSNV---VGRDLKSFVEEAKKVIAQNI-------------KLP
PSYYITYGGQFENQQ-RANKRLSTVIPLSILAIFFILFFTFKSIPLALLILLNIPFAVTGGLIALFAV------------GEYISVPASVGFIAL
FGIAVLNGVVMIGYFKELLL-----------QGKSVEECVLLGAKRRLRPVLMTACIAGLGLLPLLF-SHSVGSEVQKPLAIVVLGGL
VTSSALTLLLLPPMFMLIAKKIKIV----------------------------</seq-data>
</sequence>
<sequence seq-type="Protein">
<seq-name>o66770</seq-name>
<seq-info><length>1374</length></seq-info>
<seq-data>-----------------------------MFRAVLKYRLLVLIALVGTVIYGVYSFKKLPIDAFPDPTPVQVNIYTEAPGLSAEEVET
LITIPVESVMNG-IKDVELVRSVS-LPGLSYVSVYFKEGTDVYFARRLVMEKLPDAKERIPEGF--NPVMGPNSTGLGNVLVYV
LYDTSG------------------------------KYTPQELKSIFQQWVARPLIMSAGGVEEIVQFG-PELAYVIVPDFEKLIAYGITLEDLLN
ALERNNLLVGGGFYQS------AEGDLVVRGLGRVYSKEDLLRIPVK--------------------------------------VDEEKGVSVYLRDVA
KVVEGE----------VPNRRGAFTMNGQ-----EVQGNIVVKRIYENTKEVVDKVKEKLKEIQ--KILPEGVKIEHLYDQAYLTEKAV
STIQKALFSGMLLVAIVTAFLMGNFRTSLIVISSLPLTLLIAFIVMKETGMSGNLMTLGGLAIGIGMFVDSSVVVVENVYRHLT
E-----------NKSATKFSIILDSLKEVWRPVLFAVLIIVIVFSPIFVFE---SVEGKFFKP--LAMTLIIALLASLFVAFTFIPALSYYFLKPG
K--ETYSKLMRAIHKVYEKVLDFTFKLRTLLYLG
VFISFVFSLFLL-TRIGTEFAPELEEGAVIVKAFLDPNVSLEEGKRVATAIEREAMK---FPEVIRTFSTVGRAE-KG--EAADVNYI
ETWILLKPQSEWET-------------FKTREEFNEILRKRLE------------------WLPASLGFTQPIKMRIDELLSGVRADIAIKIFGQDP
EVLNELADKVKEIA-ESVP-GAVD----VQKEIQAGRLQLRIYPKWEVLERYGITVEEVLDVVKYNL---GGAPVGELQKD--TLLF
PIVMTLPEDFRGDVSKLAKIPLFERHGK-----ILTFGDVADLKVEPGLFVIRRENNIRFALVMLNV---EGRDTGSFVKELQSRIE
NEV--------------KLPTGYFITYGGQFENQQ-RAMKKLSIAVPVSIFLIFVMLYLNFNSVRDALVVMLNVPFAVIGGIIALYIS----
--------GFNLSVPSAIGFIAIFGVATLNGVVLVSYIRQLLQ-----------EELEIREAVKRAAILRIRPILITAITTLIGLIPLLV-IRDIGSE
VQRPLATVVVGGIFTSTLLTLIILPSVYELVYRKFPRA----------------------------</seq-data>
</sequence>
<sequence seq-type="Protein">
<seq-name>ncca_alcxx</seq-name>
<seq-info><length>1374</length></seq-info>
<seq-data>-------------------------MIESILSGAVRFRWLVLFLTAVVGAIGAWQLNLLPIDVTPDITNKQVQINTVVPTLSPVE
VEKRVTYPIETAIAG-LNGVENMRSLS-RNGFSQVTVIFKESSNLYFMRQQVTERLAQARPNLPAGV--EPQMGPVSTGLGE
VFHYSVEYEFPDGKGAKVKDGEPGWQSDGSFLTERGERLTDRVSKLAYLR-TVQDWIIRPQLRTTAGVADVDSLGGYVKQ

FVVEPDAAKMAAYGISFEELAQALEDANLSVGANFIRR------SGESYLVRADARIKSADEIARAVIA----------------------------
-------------QRQGVPITVGQVANINVG-----------GELRSGAASRNGY-----ETVVGSALMLVGANSRTVAQAVGDKLEEIK--K
TLPPGVVIVPTLNRSQLVMATIKTVAKNLVEGAALVVVILFALLGNWRAAVIAALVIPLSLLISAIGMNGLNISGNLMSLG--A
LDFGLIIDGAVIIVENSLRRLAERQHHEGRL---LTLKERLEEVILSSREMVRPTVYGQLVIFMVFLPCLTFQ---GVEGKMFSP--
MVITLMLALASAFVLSLTFVPAMVAVLLRKK--V
SEKEVRVIAVTKERYRPLLERAVARPMPFLGAALVTLALAAMAF-TFVGREFMPTLDEQNLNLSSVRIPSTSIDQSVAIDLPLE
RAVLS---LPEVQTVYSKAGTASLAA--DPMPPNASDNYIILKPKSEWPE------------GITTKEQVIERIREKTAP-----------------
MVSNNYDVTQPIQMRFNELIGGVRSDVAVKIYGENLDDLASTAQKIAAVL-RKTP-GATD----TRVPLTGGFPTFDIVFDRA
AIARYGLTVKEVADTVAAAM---AGRPSGQIFDG--DRRYDIVIRLPGQQRENLDVLGALPVMLPAVEGQPRASVPLRQLVQ
FRFTQGLNEVSRDNGKRRVYVEANV---GGRDLGSFVDDAAKRIAAEV--------------KLPPGMYIEWGGQFQNLQ-AATQR
LAIIVPLCFILIAATLYMAIGSAALTATVLTAVPLALAGGVFALVLR------------DIPFSISASVGFIAVSGVAVLNGLVLISAIRKRL
E-----------DGAAPNEAVIEGAMERVRPVLMTALVASLGFVPMAI-ATGTGAEVQKPLATVVIGGLITATVLTLFVLPAVCGM
VLRRQKKLEKPGGELLEA------------------</seq-data>
</sequence>
<sequence seq-type="Protein">
<seq-name>CNRA_ALCEU</seq-name>
<seq-info><length>1374</length></seq-info>
<seq-data>-------------------------MIESILSGSVRYRWLVLFLTAVVAVIGAWQLNLLPIDVTPDITNKQVQINSVVPTMSPV
EVEKRVTYPIETAIAG-LNGVESTRSMS-RNGFSQVTVIFKESANLYFMRHEVSERLAQARPNLPENV--EPQMGPVSTGLGE
VFHYSVEYQYPDGTGASIKDGEPGWQSDGSFLTERGERLDDRVSRLAYLR-TVQDWIIRPQLRTTPGVADVDSLGGYVKQF
VVEPDTGKMAAYGVSYADLARALEDTNLSVGANFIRR------SGESYLVRADARIKSADEISRAVIA-----------------------------
-------------HGKMSHHVGQVARVKIG-----------GELRSGAASRNGN-----ETVVGSALMLVGANSRTVAQAVGDKLEQIS--K
TLPPGVVIVPTLNRSQLVIATIETVAKNLIEGALLVVAILFALLGNWRAATIAALVIPLSLLVSAIGMNQFHISGNLMSLG--ALD
FGLIIDGAVIIVENSLRRLAERQHREGRL---LTLDDRLQEVVQSSREMVRPTVYGQLVIFMVFLPSLTFQ---GVEGKMFSP--
MVITLMLALASAFVLSLTFVPAMVAVMLRKK---
VAETEVRVIVATKESYRPWLEHAVARPMPFIGAGIATVAVATVAF-TFVGREFMPTLDELNLNLSSVRIPSTSIDQSVAIDLPL
ERAVLS---LPEVQTVYSKAGTASLAA--DPMPPNASDNYIILKPKSEWPE------------GVTTKEQVIERIREKTAP----------------
-MVGNNYDVTQPIEMRFNELIGGVRSDVAVKVYGENLDELAATAQRIAAVL-KKTP-GATD----VRVPLTSGFPTFDIVFDR
AAIARYGLTVKEVADTISTAM---AGRPAGQIFDG--DRRFDIVIRLPGEQRENLDVLGALPVMLPLSEGQARASVPLRQLVQ
FRFTQGLNEVSRDNGKRRVYVEANV---GGRDLGSFVDDAAARIAKEV--------------KLPPGMYIEWGGQFQNLQ-AATKR
LAIIVPLCFILIAATLYMAIGSAALTATVLTASPLALAGGVFALLLR------------GIPFSISAAVGFIAVSGVAVLNGLVLISAIRKRL
D-----------DGMAPDAAVIEGAMERVRPVLMTALVASLGFVPMAI-ATGTGAEVQKPLATVVIGGLVTATVLTLFVLPALCG
IVLKRRTAGRPEAQAALEA------------------</seq-data>
</sequence>
<sequence seq-type="Protein">
<seq-name>czca_alcsp</seq-name>
<seq-info><length>1374</length></seq-info>
<seq-data>-------------------------MFERIISFAIQQRWLVLLAVFGMAGLGIFSYNRLPIDAVPDITNVQVQVNTSAPGYSPL
ETEQRVTYPIEVVMAG-LPGLEQTRSLS-RYGLSQVTVIFKDGTDVYFARQLVNQRIQEAKDNLPEGV--VPAMGPISTGLGE
IYLWTVEAEE----GARKADG---------------------TAYTPTDLR-EIQDWVVRPQLRNVPGVTEINTIGGFNKQYLVAPSLERLAS
YGLTLTDVVNALNKNNDNVGAGYIER------RGEQYLVRAPGQVASEDDIRNIIVG---TAQ
GQPIRIRDIGDVEIG-----------KELRTGAATENGK-----EVVLGTVFMLIGENSRAVSKAVDEKVASIN--RTMPEGVKIVTVYD

RTRLVDKAIATVKKNLLEGAVLVIVILFLFLGNIRAALITATIIPLAMLFTFTGMVNYKISANLMSLG--ALDFGIIIDGAVVIVEN
CVRRLAHAQEHHGRP---LTRSERFHEVFAAAKEARRPLIFGQLIIMIVYLPIFALT---GVEGKMFHP--MAFTVVLALLGAMIL
SVTFVPAAVALFIGER--VAEKENRLMLWAKRR
YEPLLEKSLANTAVVLTFAAVSIVLCVAIA-ARLGSEFIPNLNEGDIAIQALRIPGTSLSQSVEMQKTIETTLKAK--FPEIERVFAR
TGTAEIAS--DPMPPNISDGYIMLKPEKDWPE------------PKKTHAELLSAIQEEAGK-----------------IPGNNYEFSQPIQLRF
NELISGVRSDVAVKIFGDDNNVLSETAKKVSAVL-QGIP-GAQE----VKVEQTTGLPMLTVKIDREKAARYGLNMSDVQDAV
ATGV---GGRDSGTFFQG--DRRFDIVVRLPEAVRGEVEALRRLPIPLPKGVDARTTFIPLSEVATLEMAPGPNQISRENGKRRI
VISANV---RGRDIGSFVPEAEAAIQSQV--------------KIPAGYWMTWGGTFEQLQ-SATTRLQVVVPVALLLVFVLLFAMFN
NIKDGLLVFTGIPFALTGGILALWIR------------GIPMSITAAVGFIALCGVAVLNGLVMLSFIRSLRE-----------EGHSLDSAVR
VGALTRLRPVLMTALVASLGFVPMAI-ATGTGAEVQRPLATVVIGGILSSTALTLLVLPVLYRLAHRKDEDAEDTREPVTQTH
QPDQGRQPA--------</seq-data>
</sequence>
<sequence seq-type="Protein">
<seq-name>Q9RLI8</seq-name>
<seq-info><length>1374</length></seq-info>
<seq-data>-------------------------MFERIIQFAIEQRWLVLLAVLGMAGVGIGSYQKLSIDAVPDITNVQVQINTAAPGYSPL
EVEQRITYPVETVMAG-LPGLQETRSLS-RPGISQVTVIFEEGTDIYFARQQVNERLSTAREQLPEDI--SPTLGPISTGLGEIYL
WTVEAEE----GATKEDG--------------------SAYTPTDLR-TIQDWIIRPQLRNVKGVAEINTIGRYAKQFLIAPDPKKLAAYKLT
LGDLQNAVLRNNENVGAGYIER------RGEQLLIRAPGQVKDMDDIRGIIVS---NVDGVPI
RIRDVAEVGLG-----------KELRTGRATENGR-----EVVLGTVFMLIGENSREVAQAVGQRLEEIN--RTLPKGVKAITVYDRTTL
VDKAVATVKKNLVEGAALVIAVLFLFLGNIRAALITATIIPLSMLFTFTGMVGNRVSANLMSLG--ALDFGIIVDGAVVIVENAI
RRLAHAQAHHGRQ---LTRAERFHEVFAASREARRALIFGQIIIMVVYLPIFALT---GVEGKMFHP--MAFTVVTALLGAMILS
VTFVPAAIALFITGK--VKEEENFVMRRARLAYE
PALRWVLGHRALVVGGALGVILLTALVA-SRMGSEFIPSLSEGDFAMQGLRVPGTSLTQSVEMQQTLEKKLMGK--FPEID
GFFARTGTAEIAS--DLMPPNASDSYVMLKPQSQWPD------------PKKSREALLEELQAAALE-----------------VPGSVYEFSQ
PIQLRFNELISGVRSDVAVKVFGDDMQVLNDTAEKISKVL-QGID-GASE----VKVEQTTGLPVLTVDIDRDKAARFGLNVGD
IQDTVATAL---GGRNAGTLFEG--DRRFDIVIRLPETLRADLPALSNLLIPLPPNNLARIDFIPLSDVARLDLSPGPNQISRENGK
RRIVVSANV---RGRDIGSFVLEAQQKLQDGV--------------KIPAGYWTTWGGQFEQLQ-SAAKRLQVVVPVALLLVFTLLFA
MFNNVKDGLLVFTGIPFALTGGVLALWLR------------GIPLSISAAVGFIALSGVAVLNGLVMISFIRNLLQ-----------EGRSLD
QAVWEGAITRLRPVLMTALVASLGFVPMAL-ATGTGAEVQRPLATVVIGGILSSTMLTLLVLPVLYRWTHGRDAGPREETP
AAG--------------------</seq-data>
</sequence>
<sequence seq-type="Protein">
<seq-name>hela_legpn</seq-name>
<seq-info><length>1374</length></seq-info>
<seq-data>-------------------------MLEKIIRFSLKHRWFVLLFTLVIAILGVYNFQRLPIDAVPDITNVQVQINTQASGYSPFEV
EQRITFPIELAMSG-LPSLDYTRSLS-RYGLSQVTVVFKDGTNIYFARQLINERLQEVKDKLPPGV--ETTLGPISTGLGEIFMYT
VTNKP----NVPIS----------------------QHYNPTELR-TIQDWIIKPQLRNVEGVAEVNTIGGYEKQFHITPDPSKLVRYRLSLND
VVEALERNNANVGAGYIET------NGEQNLIRVPGQVQNMADIENIVIA---SFEGTPVRIR
DVAEVALG-----------KELRTGAATENSK-----EVVLGTVFILMGENSRTVSERVAAKMKDIN--KTLPEGVEAITVYNRTTLVN
ATINTVKNNLLEGALLVCVILFLFLGNIRAALITAMVIPLSMLLTITGMVENQISANLMSLG--ALDFGLIVDGAVIIVENCIKHL
AEQQHALHRV---LNLEERLKVISYATTEVIRPSIFGVFIITVVYLPILTLT---GVEGKMFLP--MAQTVIIALLASMLFALTFVPAA

VAIFLRGH---LQEKENWLVHYLSLGYAKVLRRCF
HARRVVISAAVALVVVSLGIA-FHLGGEFIPSLDEGDIAMHAMRIPGTSLTQAITMQDLVEKRIRQ---FSEVKNVFAKLGTAE
VAT--DPMPPNVADTFIILKSRKKWTN------------PKKTKPGLVQEIESAVQQ-----------------IPGNNYEFTQPIQMRFNELIS
GVRSDVAVKVFGDDMDTLLKTAEAISAQL-KQVP-GAAD----VKVEQVSGLPLLTVEINRDVLARYGLQIGTVQEAVVIAT---
GGKKGGELFEG--DKRFDIVVRLPESLRSDPNVLRQIFIPLPLSKDGEQHFIPLSEVASLIRSESPNQISRENGKRRVVVTANV--
-RNRDLSSFVSEAKKRIDGQV--------------KLPSGYWITWGGQFEQLQ-SAYQRLQIVVPITLLGIFLLLFISFGKVRDALLVFT
GIPLALTGGVFALWLR------------GIPLSISAGVGFIALSGVAVLNGLVMITFINKLREQ----------KKVYLKDAVLQGSLARLRP
VLMTALVASLGFVPMAL-ATGTGSEVQRPLATVVIGGIISSTFLTLLVLPGLYYVFHGRRKKGQSKSEPQEQIM----------------
-</seq-data>
</sequence>
<sequence seq-type="Protein">
<seq-name>q55935</seq-name>
<seq-info><length>1374</length></seq-info>
<seq-data>---------------------MLNTLLNQILKTSIAQRWFIVIAAIGITLWGIISVGQMPLDVFPEFAPPQVDIHTEAPGLAP
EEVETQITVPIESAVNG-LPGVTTVRSSS-KVGLSMVSVVFDQDADVYKARQTVTERLQQVTNQLPEGSH-PPEISPLVSPLG
TIVQYAFTIKDGG------------------------------SSNLMDLR-RLLETTVGNQLLSVPGVSQVTLYGGDERQEQVLVDPAKLRALK
VSLNEVTQASAEANSNAPGGFLIG------GGQELLVRGLGQMQSIEDLRRSVVK--VVDG
KPILLEDVAEVKTG-----------SALKRGDGSFNGQ-----PAIVMMVNKQPDVDTPTVTKAVEAVVESLK--PTFPADVQIAQTF
RQANFIDSAIRNVSTSLLEGIVIVSVIMLIFLMNWRTAAITLTAIPLSLLIGLMFMKAWGLGINTMTLGGLVVAIGSVVDDSIV
DMENCYRGLRTNQAE-------GNPKHPLRVVYETSVEVRLAVIFSTVIIVVVFAPIFSLT---GVEGRIFAP--MGLAYLLCIGASTL
VAMTVSPALCGILLAN--QRLPQEGTFVSRWAE
RLYRPLLNFSLRAPQVILSIALIAVIASVSLV-PSLGRVFLPEFREKSMVNSMVLFPGVSLDMTNRAGMALFNNLKD---NPLYE
WVQIRAGRAPGDA--DGAGVSMAHVDVEL-SDEALKD----------------REASVKELRKAFNQ-----------------LPGVASNMG
GFISHRMDEVLSGVRSAIAVKIFGPDLKELRAIGEQVQEAM-KTVP-GIVD----LQLEPQLPIRQVQIHYDRAAAAQYGLRMA
DISAVVETAL---NGRIVSQVPED--QQLVNVVVMLPETERNSLDAMGAIPISTPTGQ-----MITLGDVAKIDYGMGANVVNR
EDVSRLIVVSANV---AERDLGSVVEDVQAQIKEKV--------------QLPQGYFIEYGGQFESEQ-RATNSLLLFSFVAALVIGILMF
FSVKSLPATIAIMINLPLALIGGLLSVVFT------------GGVISIASLVGFITLFGVAVRNGLLLVDNYNQKFA-----------QGMKLK
ETIFKGSMERVNAILMTALTSALGMLPLAT-ASSAGNEILQPLAIVVLGGLCTSTALTLLVLPALYAKFGKWLMPKQPTNGD
HIAPFVRNDRVSTQKSHF----</seq-data>
</sequence>
<sequence seq-type="Protein">
<seq-name>O31501</seq-name>
<seq-info><length>1374</length></seq-info>
<seq-data>-------------MTSQSIKNVMEDMMNHVINFVLKNKFAVWLMTIIVTAAGLYAGMNMKQESIPDVNMPYLTIS
TTYPGATPSQVADEVTKPVEQAVQN-LDGVSVVTSTS-YENASSVMIEYDYEKDMDKAKTEAAEALENV--NLPDDAK-DPE
ISRYSLNSFPILTLSVSSDK---------------------------------DNLQELT-KQVEDSLVSKLEGIEGVASVQVSGQQVEEVEFSFKED
KLKEYGLDEDTVKQVIQGSDVTTPLGLYTF------GNEEKSVVVSGDIETIKDLKNMRIPTASASSAGSSAASQAGAQSAQAA
QSAQAAAQV-----QQSASTAVPTVKLSDIATIKD------------VKKAESVSRTNGK-----DSIGINIVKANDANTVEVADDVKAEL
KKFK--EDHK-GFNYSATLDMAEPITQSVDTMLSKAIFGAIFAIVIILLFLRDIKSTLISVVSIPLSLLIALLVLQQLDITLNIMTLGA
MTVAIGRVVDDSIVVIENIYRRMRLK----------DEPLRGKALVREATKEMFKPIMSSTIVTIAVFLPLALVG---GQIGELFIP--F
ALTIVFALAASLVISITLVPMLAHSLFKKSLTGA---PIK
AKEHK-PGRLANIYKKVLNWALSHKWITSIIAVLMLLGSLFLV-PLIGASYLPSEEEKTMQLTYSPEPGETKKEAENEAEKAEKI

LLD---RKHVDTVQYSLGSGSPLA-----GGDSNGALFYIKYESDTP-------------------DFDKEKDNVL--------------------KEIQKQ
SDRGEWKSQDFSSSGNNNELTYYVYGDSENDIKDTVKDIEKIM-KDEK-DLKN----VNSGLSSTYDEYTFVADQEKLSKLGLT
ASQISQALMSQT---SQEPLTTVKKD--GKELDVNIKTEKDEYKSVKDLENKKITSATGQ-----EVKIGDVAKVKEGSTSDTVSKR
DGKVYADVTGEV---TSDNVTAVSAAIQKKIDKL---------------DHPDNVSIDTGGVSADIA-DSFTKLGLAMLAAIAIVYLVLVI
TFGGALAPFAILFSLPFTVIGALVGLYVS------------GETISLNAMIGMLMLIGIVVTNAIVLIDRVIHKEA-----------EGLSTREA
LLEAGSTRLRPILMTAIATIGALIPLALGFEGGSQVISKGLGVTVIGGLISSTLLTLLIVPIVYEVLAKFRKKKPGTEEE--------------
--------</seq-data>
</sequence>
<sequence seq-type="Protein">
<seq-name>O51165</seq-name>
<seq-info><length>1374</length></seq-info>
<seq-data>----------------------------MLVKRIVGKPITMLILFSLLLMISLYTFSRLKVDLLPGIDIPQISIHTVYPGASPREVEES
VSRVLESGLSS-VKNLKNIYSVS-SKESSTVSLEFYHGTDLDLVLNEIRDALELVKSSLPSKSQ-TPRIFRYNLKNIPVMEIVINSV--
--------------------------------RPVSELK-RYADEIIKPGLERLDGVAIVTVNGGSKKRVLIEVSQNRLESYGLSLSRISSIIASQNLEL
SAGNILE------NNLEYLVEVSGKFKSIEEIGNVVIAYKIPDISSGINLS--------------------------------PIEIKLKDIANIKTD-----------
FEDLSEYVEYNGL-----PSISLSVQKRSDSNSIAVSNVVMNEIEKLK--LSMPKDMKLEIASDSTDFIKASISTVVNSAYFGAMLA
IFVIFFFLRSFRATIIIGISIPIAIVLTFCLMYFVNISLNIMSLAGLALGIGMVVDCSIVVIDNIYKYR-------------QKGAKLISSSILG
AQEMMLPITSSTFTSICVFGPFLIFKSELGVYGDFFKD--FTFTIVISLGVSLLVAIFLVPVLSSHYVGLYTSFQ---------------------
---KNIKNAFIRKIDAFFASIYYFLEFLYINLLNIVLNHKLIFGLIVFFSFIGSLLLG-LLL
DVTTFTRGKENSITINLNFPHKTNLEYAKFYSNRFLEIVKS--EAKGYKSIIATL-RADR---------ITFNVLFPLKEESRDNL---------
--TQSVDYDSIKYKIMNRIG-------------------NLYPEFNIEPS-ISGNALGGGD--SIKIKISANDFEYIKDYGKILVSMLKKEIP-ELV
N----PRLSISDFQLQIGVEIDRALVYNYGIDMNTILNELKANI---NGVVAGQYVEK--GLNYDIVLKLDRMDVKNLKDLEKIFIT
NSSGV-----KIPFSSIATFEKTNKAESIYRENQALTIYLNAGI--SPDDNLTQVTAKVVDFINNKV--------------PHKEGITLKVEG
EYNEFS-NIMNQFKIIIMMAIIVVFGIMASQFESFLKPFIIIFTIPLTAIGVVLIHFLA------------GEKLSIFAAIGMLMLVGVVVN
TGIVLVDYTGLLIK-----------RGFGLREAIIESCRSRLRPILMSSLTSIIGLIPMAF-SSGSGNELLKPIAFTFIGGMTASTFLTLFFIP
MLFEIFPTCFKFQI---------------------------</seq-data>
</sequence>
<sequence seq-type="Protein">
<seq-name>Q9WYK5</seq-name>
<seq-info><length>1374</length></seq-info>
<seq-data>---------------------------MKIAEISSKRPIAIFMLLMAVVFLGFISFQHLKLDLLPQIEYPYAVVITTYMGAGTEEVE
ELVTKPLEGYISS-TPGVRRFTSMS-MDSVSFILVEFDWNVKTSEAVGRLSRYVDLATRDLPEGA--NPVVVEFDPSLLPVYAF-
-STED---DYED--VVSKIKRLPEVASVEVMGKPEKIVEVILDQEKIKRFDVDP-SLIETILSGNL
VYPFGYVSDS-----EGNIYPVSVDGRFENIEDLKNTIVGFRGVKYQSLLQGKV---------------------------PNILVPVRLRNIAEV
NIT-----------NESVQGEVRVNGK-----KASLIAIYKRSGANTVKTVREIKRILRDSK--------INYIEAMNQAYYTERAIDNLLKNL
LYGFIAAFVVVLIFLKDFTSTIVTSFSIPLSLTITFVLLYAFGLNLDTLTLGGLIMALGMLVDNSIVVFENIYRHR-------------SLGD
PIPDAARKGAGEVWLAILASTLTTVSVFLPIVFFT---GFVVRLFKY--FAIAFVFALSSSLFVSTVVVPAGTRWIKPRRG----------
---KLTEALQKHYRKALEWCLKRKKLVLVISFALVAISV
IYL-LSKPLGFLPSFQTNLMVIDVKLPSQSSYEKTAEAVKQIEDYLNEHRNEYKISSFYAEVGITNVYSQIMGRGQNEARIMVN
LGGSRREFA------------------KNREKLKNAISQL---------------NIPEAKIEVLEQS--QQIYEILGY--PVTIEVRGDDLSLIQEKAKE
IFERL-RKLQ-EVKK----IQIRNSFEKEVLHVKIDRNKALMNGVVAGQVFLDLQTYL---LGKKIGTLRTE--EGTLPIVLKKAKFLS-
-PEDLSKISLTGLKG------DVPLGVVSNISRETLPSIVEHSDGERVVYVDLVS---VEGSIGEVAKKVEKLLNEI---------------DLS-

GVKVEITGQKSFMD-EAFSELQYVVYIAIALVYMILAAQFESFVLPFVIFFTIPFAVVGVAFAVFVN------------GYSLNVPVLV
GLLTLSGTVVNNAIVMLTRIEQIR------------QEKGLLEATLEGAQSRLRPILMTTFTTIVALLPTAL-SHAEGSEIESPISWVVIF
GMLISMLFTLFVIPVIYAAIRRKVRV----------------------------</seq-data>
</sequence>
<sequence seq-type="Protein">
<seq-name>o69238</seq-name>
<seq-info><length>1374</length></seq-info>
<seq-data>---------------------------MGIVRFALRLPHTFYVLAALILFLGGIAIRSMPTDIFPEIRIPVVTVIWRYTGLSTPEME
QRVSTYSQYSISANVTGIKNIEAQT-LNGLSVQKIYFQPDVNLDLAISQIVSATNAIRALMPPGIQ-PPIIVQFNASSVPVLQLS
LESNS----------------------------------LNEQQLY-DFGIYRVRQQLAPVPGVTLPTPAGGKYRQIMVDIDPNKLLSRGLTPLDIV
NAVNTQNLTLPTGTTKI------GDTQYTVRTNATPATIQDLNMIPVK--FANGATIFLKDV
AQVRDG-----------AQVQQNIVREDGH-----RAVLLSVIKNGNASTLAVVNGVKAALTSIR--ASAPAGLKINELFDQSIFVTHS
VNGVLREGAIAAGLTALMILVFLGSWRSTLVVLISIPLAMLSSLVVLYFLGETLNTMTLGGLALAVGILVDDSTVTIENTPALD
RRGHA--------VVGSDPARSRRDRYSDVGLHARDQLRVHLGRIS---------GRPGQIFVQRRLASPWCSRCWRRTDCRGRST
PITIGLLLKGERRHGEGERSSN---IFARASAAFERGFEHLRDGY
SDLLLTLLRRRVIVPVVAVLVLALGATMF-VYVGRDFYPLIDGGQIQLHVRAPAGTRIERTEAIFQAVEDKIRE--VIPKRDRALI
VDNIGLPAR--------PYNLAFTDGSTIGVNDGTILVSLK---DGHKPTADYIRKLRQVLPS----------------AFPEDTFYFQAAD-IV
TQILNFGLPAQIDVRTVGYGSNNL-AVAKELQKSL-AAIP-GVVD----AHLQQEVDAPAFYADIDRTRAAQLGLNASTVATNI
NVSLSSSAQVSPNFWTDPTSGIPYYLAVQTPEYRANSLNSLGNTPVSASLAASGQTVPGLLSNVATFKRGTVPTNLNQANV
QPVFDVYASV---QGRDLGSVASDIDKVTSTLQK-------------QLQPGNSIQVLGQIQSMH-QSFRDLGIGLLFAAILVYLLMV
VNYQNFGDPFVVIMALPATFCGIVTMLFVT------------GTTLNVPSLMGAIMAVGVASANSILLVTFARDEQL-----------KG
HSAFQAALSAGRTRIRPVLKTAAAMIVGMIPMAI--GGPGEEQNAALARAVIGGLLFATPTTLLIVPYLFAMLRKGNDGKPR
HGVFEEQPE-----------------</seq-data>
</sequence>
<sequence seq-type="Protein">
<seq-name>p74461</seq-name>
<seq-info><length>1374</length></seq-info>
<seq-data>--------------------MSSPNSVFSLSGLAIRRHIATLMLTLAIIVLGVFAVFSLPVDLLPSITYPRIGVRLDAPGVSPEV
AVDEITRPLEAALSA-TEGVVQVYSQT-REGQISLDLFFEPGGNIDQALNDATATFNRARNQLPDDLE-TPRLFKFDPSQLPV
YEFAVTSPE---------------------------------LSGPSLR-VFAEEELARELGVVPGVASVNVSGAAQEEVRINVDLQRLQRSGVSLT
QVLDALQSRNVDISGGRIVG------TESEPLTRTVGRFASAQEIEDLVVGTVSGLEDQS-----------------------------------AQKV
YLRDVATVIDG-----------TEEERIFVTLNGN-----PAVKVSVQKQPEANTIEVVDGVKKRLEELRTEGIIPQAAELTPTLDDSVFI
RNSVNNVVVSGLIGTVLAAIAVLLFLGSLRQTLIIVLAIPLATMAAIIVMKAFGLSLNVFSLGGLALGVGIVVDNSIVMLETIAE
GAGMTPGKVNPLP--LTKGEMRNQAIASSQTVESALVASTSTNLVAVLPFLMIG---GFIALIFNE--LILTISFAVAASILVAVTL
VPMAASRLLAIRRRSGLGN--WLFFREFNRRFAGATAA
YARFLSILIRHRLVAVASIFIVFGGSSLWMIGQIPQEILPRINTGQASMFAQFPPGTPLEENQRLMAIVDDILIN---QPETEYAF
TTVGGFLFGSN-VNANALRSSSTITLKPNTDVE---------------AFTERVTAELEALNL------------------VDIRLRMAPGQLRGLI
LSNSPLRNVDVDVVLQGNDADVLDEAGRAVLAEL-GEKV-TLAR----FRPDADPRQPEVQIRPDWQRATELGLTTQAIGQT
VQTAL---DGAVPTQLQRE--NRLVDVRVKLDNDLLSGPGDLAQIPLFIDGDR-----PIRLGDVATIDQGRAPGEIQRINQRPVF
LIAGTL--VEGASLSEALTEVDQVLSAME---------------FPPGVSRLPSTAAASNE-QLQSSLVILGGLAAFLVFVVMAVQYNSL
LDPLVIMFTLPLALAGGILGLYVT------------QTAIGATVIVGTVLLVGIVVNNAIIMVELANQIWAE----------EGISREAAILRA
APQRLRPILMTTITTVLGMFPLAL-GIGQGSELLQPLGIVVFSGLSLATLLTLFLIPCLYVLLHQWEGVDIEKVKTDLESRLTEED

VPTKIQ------</seq-data>
</sequence>
<sequence seq-type="Protein">
<seq-name>Q9RWC9</seq-name>
<seq-info><length>1374</length></seq-info>
<seq-data>--MSTHFDEAEFRSGGTLPDGTPEPQIHPLVRFSVKNYVFSIGIFVMVVLLGLVATFRLGVELLPNFEVPVLAVS
TSYPGANPDQVDREVSRRVEDAVST-LSGVTDINTTS-VSNQSAVVITFSDSTNIDSAANSVSQAVAAIRGTLPDGAE-APVV
QKFDPNAQPILTLALLGGA---------------------------------ARPSEVT-TFAEDTLVPRLQRVEGVADVTVTGGPERQVQVLLD
PARLQGFDLAPARISGAIGSSALDLPAGTLDR------GGSTTSYSTRNTPRSAADVARIVVD-------------------------------------
-----PSTGLRVSDVATVRDA-----------SAAANSYARVNGQ-----PAVLLGVRKASGTNSVAVTDNVRAAMEAQK----LPPGY
RLTLASDTTTSTRATVNDTFREFLIAVGAVGLICLLFLGRLNTVFAVILAIPISISAAPLLFGTLGFTFNIITLLAIIVAIGIVVDDSIV
VAENVQRYR-------------DLGYSPLRSVLLGGSEVFSAVTAASFSLLAVLIPLSLMP---GILGQFFKQ--FGLGIAAAIVLSWLESL
LFLTVRMAYTREPARITWADLPAVLRRLPLTFRESLSGVKTFWGLLGLALAGAATYFGLHRAGLPAAAAGVLCVLLAPVVLT
VVRYLLTVLYALLEALTETLHTLTLSGVNRAAKAYARSLAGALRRPGVVMLVAGLFLLSAPLAL-RGLGFAFVPASDSGIATISL
TLPVGTPLAVTDELTRQVEDKLLA---HREVKLVQTASGSSGVLGG-ANANTSDLTLTLIPKAERPG------------------IDELLER
YRRELAP-------------------LVARYPGTELLVAGQAVGPGDSSDISLALTAPSQSLLEERNRAVVRLL-SADP-NIRT----VKSSLS
ATRQERTFVPDPLRLSGTGLSASDVAQALRTYN---DGTVAGQVRDG--DRSVDIVVRLDPALVSGEQSLLSQTLYSQALGA--
--NVPLSSLGRFEVAQAPATLRRFNKAYTATLDINLV-SGGPNPFAYQKDVQQRVEKAGLLA----------GGVTLGNANSF-GS
AGLTG-DLLFYGPILMVAAVLLTYLVLGSQFNSFRYPIYLLLPIPLAIVGALWTLHLF------------GVNLDVITVLGMVILLGLSTK
NSILYLEFVTERA------------RVLPLHEALLEAAELRFRPILMTTLTVLVISIPLIL-GHGEGAEFRRGLGIVILGGVVTSTLLTFYVV
PSVFWQFEKKRMATPQPKLEPVAGD-----------------</seq-data>
</sequence>
<sequence seq-type="Protein">
<seq-name>o66916</seq-name>
<seq-info><length>1374</length></seq-info>
<seq-data>----------------------MYGLAGRLANFFIDSKL-TPILILVSLALGLFAVLTTPREEEPQIVVPMIDIFIPYPGATPEEVE
ARVVFPLEKILWE-LKDIEYIYSAS-TNDMGIVTARFYVGTDPVKALVDLNSKMMSALDKAPPGVILPPVVRLMSIDDVPILTL
TLWGEN---------------------------------YDWYELR--RLAATLQNEIKKIENVANVYLIGGRPRQIRIILDPAKMAAYGISPLYIDQ
LLRSANAQAISGKINK------GNMEYLVKTGPFFKSVRDVENVVVG--VRNGRVIFLKDVA
KIVDGPSEIKDYVFFGIGEAHEYKGIEGKPGELYPAVTIAVAKRKGTNAVVVAEQVIEFVESLK-GKVIPSDVNITVTRNYGET
AQEKALELLEHLFIATFSVVLLIAVSLGV-REAIVVGLAVPVTLSIALFLNEMYGFTLNRVTLFALIFSIGILVDDAIVVVENIHRW
FE------------MQKLPPRDAIVHATDEVGNPTILATFTVIAALMPMAFVT---GMMGPYMRP--IPINASVAMFFSLLVAFIVTP
WAAYHILKKE---REHEEINIRKTLFWKLYCHIMYPLL
ASKLKRYAFYLFVLALMGGSVGLLFTKTVLVKMLPYDNKSELEIVLDMPEGTTLEDTLKVAKEIADYVGR---YSIVTDYQIYAG
TAAPFT------FNGLVRHYYLRQASNLAE---------------IQVNLVGKHDRDLQS------HEFAKLIRPKVHEIASKYGAKYVAVVE
VPPGPPVLSPIVAEVYAPDMKVQREFAWRVLKIF-KDSP-AITD----EGIYLEYPAPMIRFEVKEDKVKLAGLTKQEVVYTLKA
LI--GGWQAGIMQTTD--TEHVPIIIRLDEKYRV-PELLKNVKIPNREGK-----LIPLSELVEIKEGPIPETIYHKNLRRVIYVIGDVA-
GREEAPIYGILDVRDKILNIENPYGEVGELWMSLPVLEDRVYVKWDGEMHITL-EVFRDLGLAFAVAVFVMYVLVLGWFKD
FRIPGIILAPIPLTLVGIIPGHWLMDKIMG-------NVFFTATSMIGFIALGGIIVRNSILLVDFAEQRLK-----------EGLPVHVAVV
EAGVIRTRPILLTAAAVIIGAFVIIF------DPIFNGLAISLIFGTIGSTTLTLVLIPVMYYASKIKRMPPEACPTPEMIKRDLMAD---
---------</seq-data>
</sequence>

<column-score>
<colsco-name>coreblock</colsco-name>
<colsco-owner>0</colsco-owner>
<colsco-type>int</colsco-type>
<colsco-data>0 0 0 0 0 0 0 0 0 0 0 0 0 0 0 0 0 0 0 0 0 0 0 0 0 0 0 0 0 0 0 0 0 0 0 0 0 0 1 0 1 1 1 1 1 1 1 1 1 1 1 1
1 1 1 1 1 1 1 1 1 1 1 1 1 1 1 1 1 1 1 1 1 1 1 1 1 1 1 1 1 1 1 1 1 1 1 1 1 1 1 1 1 1 1 1 1 1 1 0 0 0 0 0 0 0 0 0 0 0 0
0 0 0 0 1 1 1 1 1 1 1 1 1 1 1 1 1 1 1 1 1 1 1 1 1 1 1 1 1 1 1 0 0 0 0 0 1 1 1 1 0 0 0 0 0 0 0 0 0 0 0 0 0 0 0 0 0 0 0
0 0 0 0 0 0 0 0 0 0 0 0 0 0 0 0 0 0 0 0 0 0 0 0 0 0 0 0 0 0 0 0 0 0 0 0 0 0 0 0 0 0 0 0 0 0 0 0 0 0 0 0 0 0 1 1 1 1 1
1 1 1 1 1 1 1 1 1 1 1 1 0 0 0 0 0 0 1 1 1 1 1 1 1 1 1 1 1 1 1 1 1 1 1 1 1 1 1 1 1 1 1 1 1 1 1 1 1 1 1 1 0 0 0 0 0 0 0
0 0 0 0 0 0 0 0 1 1 1 1 1 1 1 1 1 1 1 1 1 1 1 1 1 1 1 0 0 0 0 0 0 0 0 0 0 0 0 0 0 0 0 0 0 0 0 0 0 0 0 0 0 0 0 0 0 0 0
0 0 0 0 0 0 0 0 0 0 0 0 0 0 0 1 1 1 1 1 1 1 1 1 1 1 0 0 0 0 0 0 0 0 0 0 0 0 0 0 0 0 0 0 0 1 1 1 1 1 1 1 0 0 0 0 0 1 1
1 1 1 1 1 1 1 1 1 1 1 1 1 1 1 1 1 1 1 1 1 1 1 1 1 1 1 0 0 0 0 0 0 0 0 0 1 1 1 1 1 1 1 1 1 1 1 1 1 1 1 1 1 1 1 1 1 1 1
1 1 1 1 1 1 1 1 1 1 1 1 1 1 1 1 1 1 0 1 1 1 1 1 1 1 1 1 1 1 1 1 1 1 1 1 1 1 1 1 1 1 1 1 1 1 1 1 1 1 1 1 1 1 1 1 1 1 1
1 1 1 1 1 1 1 1 1 1 1 1 1 1 1 1 0 0 0 0 0 0 0 0 0 0 0 0 0 0 0 0 0 0 0 0 0 0 0 0 0 0 0 0 0 0 0 0 0 0 0 0 0 0 0 0 0 0 0
0 0 0 0 0 0 0 0 0 0 0 0 0 0 1 1 1 1 1 1 1 0 0 0 0 1 1 1 1 1 1 1 1 1 1 1 1 1 1 1 1 1 1 1 1 1 1 1 1 1 1 1 1 0 0 0 0 0 0
0 0 0 0 0 0 0 0 0 0 0 0 0 0 0 0 0 0 0 0 0 0 0 0 0 0 0 0 0 0 0 0 0 0 0 0 0 0 0 0 0 0 0 0 0 0 0 0 0 0 0 0 0 0 0 0 0 0 0
0 0 0 0 0 0 0 0 0 0 0 0 0 0 0 0 0 0 0 0 0 0 0 0 0 0 0 0 0 0 0 0 1 1 1 1 1 1 1 1 1 1 1 1 1 1 1 1 1 1 1 1 0 0 0 0 0 0 0
0 0 0 0 0 0 0 0 0 0 0 0 0 0 0 1 1 1 1 1 1 1 1 1 1 1 1 1 1 1 1 1 1 1 1 1 1 1 1 1 1 1 1 1 1 1 1 1 1 1 1 1 1 1 0 0 0 0 0
0 0 0 0 0 0 0 0 0 0 0 0 0 0 0 0 0 0 0 0 0 0 0 0 0 0 0 0 0 0 0 0 0 0 0 0 0 0 0 0 0 0 0 0 0 0 0 0 0 0 0 0 0 0 0 0 0 0 0
0 0 0 0 0 0 0 0 0 0 0 0 0 0 0 0 0 0 0 0 0 0 0 0 0 0 0 0 0 0 0 0 0 0 0 0 0 0 0 0 0 0 0 0 0 0 0 0 0 0 0 0 0 0 0 0 0 0 0
0 0 0 1 1 1 1 1 1 1 1 1 1 1 1 1 1 1 1 1 0 0 0 0 0 0 0 0 0 0 0 0 0 0 0 0 0 0 0 0 0 0 0 0 1 1 1 1 1 1 1 1 1 1 1 1 1 1 1
1 1 1 1 1 1 1 1 1 1 1 1 1 1 1 0 0 0 0 0 0 0 0 0 0 0 0 0 0 0 0 0 1 1 1 1 1 1 1 1 1 1 0 0 0 0 0 0 0 1 1 1 1 1 1 1 1 1 0
0 0 0 0 0 0 0 0 0 1 1 1 1 1 1 1 1 1 1 1 1 1 1 1 1 1 1 1 1 1 1 1 1 1 1 1 1 1 1 1 1 0 0 0 0 0 0 0 0 0 0 0 0 0 0 0 0 0 0
0 0 0 0 0 0 0 0 0 0 0 0 0 0 0 0 0 0 0 0 0 0 0 0 0 1 1 1 1 1 1 1 1 1 1 1 1 0 0 0 0 1 1 1 1 1 1 1 1 1 1 1 1 1 1 1 1 1 1
1 1 1 1 1 1 1 1 1 1 1 1 1 1 1 1 1 1 1 1 1 1 1 1 1 1 1 1 1 1 1 1 1 0 0 0 0 0 0 0 0 0 0 0 0 0 0 0 1 1 1 1 1 1 1 1 1 1 1
1 1 1 1 1 1 1 1 1 1 1 1 1 1 1 1 1 1 0 0 0 0 0 0 0 0 0 0 0 0 0 0 0 1 1 1 1 1 1 1 1 1 1 1 1 1 1 1 1 1 1 1 1 1 1 1 1 1 1
1 1 1 1 1 1 1 1 1 0 0 0 0 0 0 1 1 1 1 1 1 1 1 1 1 1 1 1 1 1 1 1 1 1 1 1 1 1 1 1 1 1 1 1 1 1 1 1 1 0 0 0 0 0 0 0 0 0 0
0 0 0 0 0 0 0 0 0 0 0 0 0 0 0 0 0 0 0 0 0 0 0 0 </colsco-data>
</column-score>
</alignment>
</macsim>
::::::::::::::
dtd_core-block.xml
::::::::::::::
<?xml version="1.0"?>
<!DOCTYPE macsim SYSTEM "http://www-bio3d-igbmc.u-strasbg.fr/macsim.dtd">
<macsim>
<alignment>
<aln-name>1</aln-name>
<sequence seq-type="Protein">
<seq-name>Q54756</seq-name>
<seq-info><length>1354</length></seq-info>

<seq-data>---
---MLLGQSQLRWLPGLRSLLHYRRA--WLRGDVLAGVTVAAYLIP
QCMAYGQLA---GLPAIVGLWAILIPLFLYTFFGSSPQLSVGPESSTAIMTAVAIAPVAAQTDL---------------------------------
---------------------SYSLLAAVMALLVGIVFLVAYSLRLGFLADLLSKPILIGYMAGIGLVMISGQLGKTSGIPI----------TATKPL
EEFQQFFAGL-------GQCHWPTVGVSILVLLFLFGVQKK----------------------------FRTAPGPLLAVLLATLFVALFQ----------
-LDQQGVQVIGTIPA-GLPRWQWPTLP--------------WQQWPTLTASAIGVALVGYSDNILTARAFAVR-HRYEIDANQELLA
LGIANVGNSFFQCFPISGSTSRTVIGDA-LGSRTQLFSLVSLGTVLLVLWFFRPVLAMFPQAALGAIVIYAATKLIDL---REFYR
LRRYRPSEFWLALITAAGVLGTN-MLIGVGVAVSLSVIDLFARVARPHAAILGEIPGMAGLHDIEDW-------------------------
---PQAQTFPGLVIFRYDAQ-LCFANAEDFKRRVLLAIA-------------------------
--TAPQP-VQWLLLNAEAIIN--LDVTAAEKLLELLRELQQR----GVTLTIARA-
KQELIAELDRVGLV-EQIGGE-HFYPTLPTAIAAFQQFRQSQIADEPPLTAS--

---</seq-data>
</sequence>
<sequence seq-type="Protein">
<seq-name>P71997</seq-name>
<seq-info><length>1354</length></seq-info>
<seq-data>---
---MIPTMTSAGWAPGVVQFREYQRR--WLRGDVLAGLTVAAYLI
PQAMAYATVA---GLPPAAGLWASIAPLAIYALLGSSRQLSIGPESATALMTAAVLAPMAAGDLR-----------------------------
-------------------------RYAVLAATLGLLVGLICLLAGTARLGFLASLRSRPVLVGYMAGIALVMISSQLGTITGTSV----------EG
NEFFSEVHSFATSV-------TRVHWPTFVLAMSVLALLTMLTRW----------------------------APRAPGPIIAVLAATMLVAVM
S-----------LDAKGIAIVGRIPS-GLPTPGVPPVS--------------VEDLRALIIPAAGIAIVTFTDGVLTARAFAAR-RGQEVNANAE
LRAVGACNIAAGLTHGFPVSSSSSRTALADV-VGGRTQLYSLIALGLVVIVMVFASGLLAMFPIAALGALVVYAALRLIDL---S
EFRRLARFRRSELMLALATTAAVLGLG-VFYGVLAAVALSILELLRRVAHPHDSVLGFVPGIAGMHDIDDY--------------------
--PQAKRVPGLVVYRYDAP-LCFANAEDFRRRALTVVD------------------
--QDPGQ-VEWFVLNAESNVE--VDLTALDALDQLRTELLRR----GIVF
AMARV-KQDLRESLRAASLL-DKIGED-HIFMTLPTAVQAFRRR---

---</seq-data>
</sequence>
<sequence seq-type="Protein">
<seq-name>P72770</seq-name>
<seq-info><length>1354</length></seq-info>
<seq-data>---
--MIKQIFPIFQWLPNYHPS--WLKADVVAGLTLAAYAIPVAL
AYGSLA---GLPSEVGLYCYMLGAVGYAFFGTSRQLALGPTSAISILVGVSLAPLANDDAG-------------------------------------
-----------------RYLILASSTAILVAIICLLAWLLKLSQIVNFISEPILTGFKAGAALQIASTQLPKLFGVPS----------GGSNFFSRIW
DLFHHW-------QEIQPATLLVGGLALVLLVMGDRL----------------------------WPSKPISLMVVILAIVVMGITN-----------LL
EQGVKVVGEIPQ-GLPSFGMPHWS--------------FSDLDSLLPLALACFLLSYIEGISTARSFAIK-HHYRINPEQELLAIGAANLA
AGLGQGYPIAGGLSQSAVNEK-AGAKTPLAIIITACIIAIVLLFFTGLFSNLPEAILGSVVLVAVKGLINI---PELQHLKKIAPLEFK
VSLIALFGVLCFG-VLQGVLLAAIASILFLIHIISYPSSAVLGRIPGSDQFSDLERH---

-------------------------------PENLVISGVLIYRINGP-ILYFNINNIESDLFNHLA--
--------------------------QQQEP-VELVIFEMGTSPG--IDTPAARWFKTLSQSLNQQ----GITLKLVNA-SGFVRDRLRAEGLE-G
EVGTF-RRLDTVDSLISEFNCQRYSDLPHTEND---

--</seq-data>
</sequence>
<sequence seq-type="Protein">
<seq-name>O04722</seq-name>
<seq-info><length>1354</length></seq-info>
<seq-data>---MKERDSESFESLSH
QVLPNTSNSTHMIQMAMANSGSSAAAQAGQDQPDRSKWLLDCPEPPSPW-HELKRQVKGSFLTKAKKFKSLQKQPFPK
QILSVLQAIFPIFGWCRNYKLT--MFKNDLMAGLTLASLCIPQSIGYATLA---KLDPQYGLYTSVVPPLIYALMGTSREIAIGPV
AVVSLLISSMLQKLIDPETDPL---GYKKLVLTTTFFAGIFQASFGLFRLGFLVDFL
SHAAIVGFMGGAAIVIGLQQLKGLLGITN--------FTTNTDIVSVLRAVWRSCQ------QQWSPHTFILGCSFLSFILITRFIGKKY
KK----------------------LFWLPAIAPLIAVVVSTLMVFLTK-----------ADEHGVKTVRHIKG-GLNPMSIQDLDFNT------------PH
LGQIAKIGLIIAIVALTEAIAVGRSFAGI-KGYRLDGNKEMVAIGFMNVLGSFTSCYAATGSFSRTAVNFA-AGCETAMSNIV
MAVTVFVALECLTRLLYYTPIAILASIILSALPGLIN---INEAIHIWKVDKFDFLALIGAFFGVLFAS-VEIGLLVAVVISFAKIILISIR
PGIETLGRMPGTDTFTDTNQY--PMTVKTPGVLIFR
VKSALLCFANASSIEERIMGWVDEE--EEEENTKSNAKRK-ILFV
VLDMSSLIN--VDTSGITALLELHNKLIKT----GVELVIVNP-KWQVIHKLNQAKFV-DRIGG--KVYLTIGEALDACFGLKV-------
----
---</s
eq-data>
</sequence>
<sequence seq-type="Protein">
<seq-name>SUT3_STYHA</seq-name>
<seq-info><length>1354</length></seq-info>
<seq-data>--
------------MSSLGTEQFSERSQWVLNSPNPPPLTKKFLGPLKDNKFFTSSSSK-------KETRAVSFLASLFPILSWIRTYSAT--KF
KDDLLSGLTLASLSIPQSIGYANLA---KLDPQYGLYTSVIPPVIYALMGSSREIAIGPVAVVSMLLSSLVPKVIDPDAHPN-------
---DYRNLVFTVTLFAGIFQTAFGVLRLGFLVDFLSHAALVGFMAGAAIVIGLQQLKG
LLGLTH--------FTTKTDAVAVLKSVYTSLHQQITSSENWSPLNFVIGCSFLIFLLAARFIGRRNKK----------------------FFWLPAI
APLLSVILSTLIVFLSK-----------GDKHGVNIIKHVQG-GLNPSSVHKLQLNG------------PHVGQAAKIGLISAIIALTEAIAVGR
SFANI-KGYHLDGNKEMLAMGCMNIAGSLTSCYVSTGSFSRTAVNFS-AGCKTAVSNIVMAVTVLLCLELFTRLLYYTPMAIL
ASIILSALPGLID---IGEAYHIWKVDKFDFLACLGAFFGVLFVS-IEIGLLIALSISFAKILLQAIRPGVEVLGRIPTTEAYCDVAQY--
--PMAVTTPGILVIRISSGSLCFANAGFVRERILKWVE
D---EEQDNIEEAAKGR-VQAIIIDMTDLTN--VDTSGILALEELH
KKLLSR----GVELAMVNP-RWEVIHKLKVANFV-DKIGKE-RVFLTVAEAVDACLSSRFANSA--------------------------------------
----
--</seq-data>
</sequence>
<sequence seq-type="Protein">

<seq-name>O64434</seq-name>
<seq-info><length>1354</length></seq-info>
<seq-data>---
--MGIELQNHQSHHEEASPAEEPMSRWLINTPEPPSMWQELIGYIR-TNVLAKKKHKRNKTKNSSSNLVYSCLKSAFPILSW
GRQYKLN--LFKKDLMAGLTLASLCIPQSIGYANLA---GLDPEYGLYTSVVPPLIYSTMGTSRELAIGPVAVVSLLLSSMVRDL
QDPVTDPI--AYRKIVFTVTFFAGAFQAIFGLFRLGFLVDFLSHAALVGFMAG
AAIVIGLQQLKGLFGLTH--------FTNKTDVVSVLSSVFHSLH------HPWQPLNFVIGSSFLIFILLARFIGKRNNK---------------
-----LFWIPAMAPLISVVLATLIVYLSN----------AESRGVKIVKHIKP-GFNQLSVNQLQFKS-----------PHLGQIAKIGLISAIIA
LTEAIAVGRSFATI-KGYRLDGNKEMMAMGFMNIAGSLSSCYVATGSFSRTAVNFS-AGCETVVSNIVMAITVMISLEVLTR
FLYFTPTAILASIILSALPGLID---VSGALHIWKLDKLDFLVLIAAFFGVLFAS-VEIGLLLAVGISFARIMLSSIRPSIEALGRLSKTD
IFGDINQY--PMANKTAGLLTLRISSPLLCFANANFIR
DRILNSVQEI--EGEENEQEVLKENGLQVVILDMSCMMG--VD
TSGVFALEELHQELASN----DIRLVIASP-RWRVLHKLKRAKLD-EKIKTE-NIYMTVGEAVDIYVRARSTSHELC----------------
----
--</seq-data>
</sequence>
<sequence seq-type="Protein">
<seq-name>Q43482</seq-name>
<seq-info><length>1354</length></seq-info>
<seq-data>---MPRT
VSDGGEDFDGDVCSQTASQRHTD--STHHHHGYKVGFPPAKGVFAEFAEGVKETFFADDPLR-EYKDQPRSKKLWLSLVHL
FPVLDWSRSYTFG--KFKGDLVAGLTIASLCIPQDIGYAKLA---NLQPHVGLYSSFVPPLIYALMGSSRDIAIGPVAVVSLLLGTL
LQEEIDPVKNPL--EYSRLAFTATFFAGITQAMLGFFRLGFIIEFLSHAAIVGFM
AGAAITIALQQLKGLLGIAK--------FTKKSDIISVMESVWGNVQ------HGWNWQTILIGSSFLAFLLTTKYIAKKNKK---------
-----------LFWVSAIAPLISVVISTFCVYITR----------ADKQGVAIVKNIKQ-GINPPSFDLIYWSG-----------PYLAKGFRIGVV
SGMVALTEAIAIGRTFAAM-KDYQIDGNKEMVALGTMNIVGSMTSCYVATGSFSRSAVNYM-AGCKTAVSNVVMAIVV
MLTLLLITPLFKYTPNAILASIIINAVVNLVD---YETAYLIWKVDKMDFVALLGAFFGVVFAS-VEYGLLIAVAISLGKILLQVTRP
RTALLGNLPRTTIYRNVEQY---PEATKVPGVMIVRV
DSA-IYFTNSNYVKERILRWLR---DEEEQQQEQKLSK-TEFLIV
ELSPVTD--IDTSGIHALEELLKALEKR----KIQLILANP-GPAVIQKLRSAKFT-DLIGDD-KIFLSVGDAVKKFAPKSSLNV--------
----
--</seq
-data>
</sequence>
<sequence seq-type="Protein">
<seq-name>Q9XGB5</seq-name>
<seq-info><length>1354</length></seq-info>
<seq-data>---MPRT
VSDGGEDFDGDVSSQTGSHRHTDSNSTHHHHGYKVGFPPARGLFAEFADGVKETFFADDPLR-EYKDQSRSKKLWLSLVH
LFPVLDWARSYKFS--MFKGDFIAGLTIASLCIPQDIGYAKLA---FLPAHVGLYSSFVPPLVYAMMGSSRDIAIGPVAVVSLLL
GTLLQEEIDPVKSPY---EYSRLAFTATFFAGITQAMLGFFRLGFIIEFLSHAAIIG
FMAGAAVTIALQQLKGFLGIKK--------FTKKSDIISVMESVWGNVH------HGWNYQTILIGASFLAFLLTTKYIAKKNKK------

--------------LFWVSAIAPLISVVISTFCVYITR-----------ADKQGVAIVKDIKQ-GINPPSFHLIYWSG------------PYLAKGFRIG
VVAGMVALTEAIAIGRTFAAM-KDYQIDGNKEMVALGTMNIVGSMTSCYVATGSFSRSAVNYM-AGCKTAVSNVVMAIV
VMLTLLLITPLFKYTPNAILASIIINAVVSLVD---YETAYLIWKVDKMDFVALLGAFFGVVFAS-VEYGLLIAVAISLGKILLQVTR
PRTALLGNLPRTTIYRNVEQY---PEAAKVPGVMIVR
VDSA-IYFTNSNYVKERILRWLR---DEEDQQQEQKLSK-TEFLI
VELSPVTD--IDTSGIHALEELLKALEKR----KIQLILANP-GPAVIQKLRSAKFT-ELIGDD-KIFLSVGDAVKKFAPKSALNV-------
----
--</se
q-data>
</sequence>
<sequence seq-type="Protein">
<seq-name>Q9XGB6</seq-name>
<seq-info><length>1354</length></seq-info>
<seq-data>---MSRT
MSDGSEDFNGNISR-TGSHRQ------TDHHGYKVRFPPAKGLFTELAEGVKETFFANDPLR-EYKDQPRSKKLWLSLAHLFPV
LDCPTSYTFG--MFKGDFVAGLTIASLCIPQDIGYAKLA---FLPAHVGLYSSFVPPLIYAAMGTSRDIAIGPAAVLSLLLGTLLQE
EIDPVKNPY--EYSRLAFTATFFAGVTQAMLGFFRLGFIIEFLSHAAIVGFMAG
AAITIGLQQLKGFLGIAK--------FTKKSDIISVMESVWGNVH------HGCNWQTILIGASFLAFLLTTKYIAKKNKK--------------
------LFWVSAIAPLISVIISTFCVFITR-----------ADKQGVAIVS------INPPSFHLIYWTG------------PYLVKGFRIGVVAGMVG
LT-AIAIGRTFAAL-KDYQIDGNKEMLALGTMNIVGSMTSCYVGTGSMSRSAVNYM-AGCKTAISNVVMAIVVMLTLLLVT
PLFKYTPNAILASIIINAVVSLVD---YETAYLIWKVDKMDFMALLGAFFGVVFAS-VEYGLLIAVAISLGKILLQVTRPRTALLGN
LPRTTIYTNVEQY---PEATKVPGVMIVRVDSA-IYFTN
SNYVKERILRWLR---DEEEQQQEQKLSK-TEFLIVELSPVTD--I
DTSGIHALEELLKALEKR----KIHLILANP-GPAVIQKLRSAKFT-ELIGDD-KIFLSVDDAVKKFAPKSALNV-----------------------
----
--</seq-data>
</sequence>
<sequence seq-type="Protein">
<seq-name>SUT2_STYHA</seq-name>
<seq-info><length>1354</length></seq-info>
<seq-data>--MSQRVSD
QAMAEVIAETRTNSSSRRHGGG---DDTPSLPYMHKVGAPPKQTLFQEIKHSFNETFFPDKPFG-NFKDQSGSRKFVLGLQY
IFPILEWGRHYDLK--KFRGDFIAGLTIASLCIPQDLAYAKLA---NLDPWYGLYSSFVAPLVYAFMGTSRDIAIGPVAVVSLLLG
TLLSNEISNTKSH--DYLRLAFTATFFAGVTQMLLGVCRLGFLIDFLSHAAIVG
FMAGAAITIGLQQLKGLLGIKD--------FTKNSDIVSVMHSVWSNVH------HGWNWETILIGLSFLIFLLITKYIAKKNKK-------
--------------LFWVSAISPMICVIVSTFFVYITR-----------ADKRGVTIVKHIKS-GVNPSSANEIFFHG------------KYLGAGVRVG
VVAGLVALTEAMAIGRTFAAM-KDYSIDGNKEMVAMGTMNIVGSLTSCYVTTGSFSRSAVNYM-AGCKTAVSNIVMAIV
VLLTLLVITPLFKYTPNAVLASIIIAAVVNLVN---IEAMVLLWKIDKFDFVACMGAFFGVIFKS-VEIGLLIAVAISFAKILLQVTR
PRTAVLGKLPGTSVYRNIQQY---PKAEQIPGMLIIRV
DSA-IYFSNSNYIKERILRWLI---DEGAQRTESELPE-IQHLIVE
MSPVTD--IDTSGIHAFEELYKTLQKR----EVQLMLANP-GPVVIEKLHASNLA-ELIGED-KIFLTVADAVATYGPKTAAF-------
----

--</se
q-data>
</sequence>
<sequence seq-type="Protein">
<seq-name>SUT1_STYHA</seq-name>
<seq-info><length>1354</length></seq-info>
<seq-data>---MSQRVSD
QVMADVIAETRSNSSSHRHGGGGGGDDTTSLPYMHKVGTPPKQTLFQEIKHSFNETFFPDKPFG-KFKDQSGFRKLELGL
QYIFPILEWGRHYDLK--KFRGDFIAGLTIASLCIPQDLAYAKLA---NLDPWYGLYSSFVAPLVYAFMGTSRDIAIGPVAVVSLL
LGTLLSNEISNTKSH--DYLRLAFTATFFAGVTQMLLGVCRLGFLIDFLSHAAI
VGFMAGAAITIGLQQLKGLLGISNNN------FTKKTDIISVMRSVWTHVH------HGWNWETILIGLSFLIFLLITKYIAKKNKK---
-----------------LFWVSAISPMISVIVSTFFVYITR-----------ADKRGVSIVKHIKS-GVNPSSANEIFFHG------------KYLGAGVR
VGVVAGLVALTEAIAIGRTFAAM-KDYALDGNKEMVAMGTMNIVGSLSSCYVTTGSFSRSAVNYM-AGCKTAVSNIVMSI
VVLLTLLVITPLFKYTPNAVLASIIIAAVVNLVN---IEAMVLLWKIDKFDFVACMGAFFGVIFKS-VEIGLLIAVAISFAKILLQVT
RPRTAVLGKLPGTSVYRNIQQY---PKAAQIPGMLIIR
VDSA-IYFSNSNYIKERILRWLI---DEGAQRTESELPE-IQHLITE
MSPVPD--IDTSGIHAFEELYKTLQKR----EVQLILANP-GPVVIEKLHASKLT-ELIGED-KIFLTVADAVATYGPKTAAF---------
----
--</seq
-data>
</sequence>
<sequence seq-type="Protein">
<seq-name>O22277</seq-name>
<seq-info><length>1354</length></seq-info>
<seq-data>--
---MSGTINPPDGGGSGA--RNPPVVRQRVLAPPKAGLLKDIKSVVEETFFHDAPLR-DFKGQTPAKKALLGIQAVFPIIGWAR
EYTLR--KFRGDLIAGLTIASLCIPQDIGYAKLA---NVDPKYGLYSSFVPPLIYAGMGSSRDIAIGPVAVVSLLVGTLCQAVIDPK
KNPE---DYLRLVFTATFFAGIFQAGLGFLRLGFLIDFLSHAAVVGFMGGAAITI
ALQQLKGFLGIKT--------FTKKTDIVSVMHSVFKNAE------HGWNWQTIVIGASFLTFLLVTKFIGKRNRK--------------------
LFWVPAIAPLISVIISTFFVFIFR-----------ADKQGVQIVKHIDQ-GINPISVHKIFFSG------------KYFTEGIRIGGIAGMVALTE
AVAIARTFAAM-KDYQIDGNKEMIALGTMNVVGSMTSCYIATGSFSRSAVNFM-AGVETAVSNIVMAIVVALTLEFITPLFK
YTPNAILAAIIISAVLGLID---IDAAILIWRIDKLDFLACMGAFLGVIFIS-VEIGLLIAVVISFAKILLQVTRPRTTVLGKLPNSNVY
RNTLQY---PDAAQIPGILIIRVDSA-IYFSNSNYVRERA
SRWVR--EEQENAKEYGMPA-IRFVIIEMSPVTD--IDTSGIHS
IEELLKSLEKQ----EIQLILANP-GPVVIEKLYASKFV-EEIGEK-NIFLTVGDAVAVCSTEVAEQQT--------------------------------
----
--</seq-data>
</sequence>
<sequence seq-type="Protein">
<seq-name>O49307</seq-name>
<seq-info><length>1354</length></seq-info>
<seq-data>--

-----------------------MEVHKVVAPPHKSTVAKLKTKLKETFFPDDPLR-QFRGQPNRTKLIRAAQYIFPILQWCPEYSFS--LLK
SDVVSGLTIASLAIPQGISYAKLA---NLPPIVGLYSSFVPPLVYAVLGSSRDLAVGPVSIASLILGSMLRQQVSPVDDPV---------
---LFLQLAFSSTFFAGLFQASLGILRLGFIIDFLSKATLIGFMGGAAIIVSLQQLKGLLGIT
H--------FTKHMSVVPVLSSVFQHT-------NEWSWQTIVMGVCFLLFLLSTRHLSMKKPK--------------------LFWVSAGAPL
LSVIVSTLLVFVFR-----------AERHGISVIGKLPE-GLNPPSWNMLQFHG------------SHLALVAKTGLVTGIVSLTEGIAVGRTF
AAL-KNYHVDGNKEMIAIGLMNVVGSATSCYVTTGAFSRSAVNNN-AGAKTAVSNIVMSVTVMVTLLFLMPLFEYTPNVV
LGAIIVTAVIGLID---LPAACHIWKIDKFDFLVMLCAFFGVIFLS-VQNGLAIAVGLSLFKILMQVTRPKMVIMGNIPGTDIYRD
LHHY---KEAQRIPGFLVLSIESP-VNFANSNYLTERTS
RWIE---ECEEEEAQEKHSS-LQFLILEMSAVSG--VDTNGVSFF
KELKKTTAKK----DIELVFVNP-LSEVVEKLQRADEQKEFMRPE-FLFLTVAEAVASLSLKGPSLSNV----------------------------
----
--</seq-data>
</sequence>
<sequence seq-type="Protein">
<seq-name>O04001</seq-name>
<seq-info><length>1354</length></seq-info>
<seq-data>---MVG
MRVPYGGSYTNNGSNESQPPGAAPEVPAMVEVHKVVPPPPQSTASKLKTRLKETLFPDDPFR-GFQGQPARVQWVLAV
KYLFPILDWLPAYSLS--LFKSDLIAGLTIASLAIPQGISYAKLA---NLPPLIGLYSSFVPPLVYAVLGSSRDLAVGPVSISSLIMGPC
CASRQPHCGAD---AVPAARLHATLFAGIFQASLGILRLGFIIDFLSKATLVGF
MAGAAIIVSLQQLKALLGIVH--------FTTEMGIVPVMASVFHHT-------KEWSWQTILMGVCFLVFLLVARHVSIRWPR-----
---------------LFWVSACAPLVSVIISTLVVFLFK-----------AQNHGISIIGQLKC-GLNRPSWDKTNIDT------------TYLGLTMK
TGLVTGIISLTEGIAVGRTFASL-KEYQIDGNKEMMAIGLMNVVGSCTSCYVTTGAFSRSPVNHN-AGCKTAMSNVIMALT
VMVTLLFLMPLFVYTPNVVLGAIIIAAVIGLID---IPAVYHIWKMDKMDFLVCVCAFAGVLFIS-VQEGLAIAVGISVFRVLLQI
TRPKITVQGNIMGTDIYRNLHQY---KDAQRIPGFLIL
ATEAP-INFANSNYLNERIKRWIE---EESSAQTKQTE---LRFVIL
DLSAVPA--IDTSGVAFLIDIKKSIEKR----GLELVLVNP-TGEGHGKNTASERGTQAFQVGIACILTTGEAVASLSALAKMASP-
----
---<
/seq-data>
</sequence>
<sequence seq-type="Protein">
<seq-name>O23250</seq-name>
<seq-info><length>1354</length></seq-info>
<seq-data>---
--------MGTEDYTFPQGAEELHRRHHTVEAPQPQPFLKSLQYSVKETLFPDDPFR-QFKNQNASRKFVLGLKYFLPIFEWAPR
YNLK--FFKSDLIAGITIASLAIPQGISYAKLA---NLPPILGLYSSFVPPLVYAVLGSSRDLAVGTVAVASLLTGAMLSKEVDAEKD
PK--LYLHLAFTATFFAGVLEASLGIFRLGFIVDFLSHATIVGFMGGAATVVSL
QQLKGIFGLKH--------FTDSTDVISVMRSVFSQT-------HEWRWESGVLGCGFLFFLLSTRYFSIKKPK--------------------FF
WVAAMAPLTSVILGSLLVYFTH-----------AERHGVQVIGDLKK-GLNPLSGSDLIFTS------------PYMSTAVKTGLITGIIALAE
GIAVGRSFAMF-KNYNIDGNKEMIAFGMMNIVGSFTSCYLTTGPFSRSAVNYN-AGCKTAMSNIVMAIAVMFTLLFLTPLF
HYTPLVVLSAIIISAMLGLID---YQAAIHLWKVDKFDFLVCMSAYVGVVFGS-VEIGLVVAVAISIARLLLFVSRPKTAVKGNIP

NSMIYRNTEQY--PSSRTVPGILILEIDAP-IYFANASY
LRERIIRWID--EEEERVKQSGESS-LQYIILDMSAVGN--IDTS
GISMMVEIKKVIDRR----ALKLVLSNP-KGEVVKKLTRSKFIGDHLGKE-WMFLTVGEAVEACSYMLHTFKTEPASKNEPWN
NV--
---</seq-
data>
</sequence>
<sequence seq-type="Protein">
<seq-name>O04289</seq-name>
<seq-info><length>1354</length></seq-info>
<seq-data>--
-----------------MSSKRASQYHQVEIPPPQPFLKSLKNTLNEILFADDPFRRIRNESKTSKKIELGLRHVFPILEWARGYSLE--YL
KSDVISGITIASLAIPQGISYAQLA---NLPPILGLYSSLVPPLVYAIMGSSRDLAVGTVAVASLLTAAMLGKEVNAVVNPK------
--LYLHLAFTATFFAGLMQTCLGLLRLGFVVEILSHAAIVGFMGGAATVVCLQQLK
GLLGLHH--------FTHSTDIVTVLRSIFSQS-------HMWRWESGVLGCCFLIFLLTTKYISKKRPK--------------------LFWISAM
SPLVSVIFGTIFLYFLH-----------DQFHGIQFIGELKK-GINPPSITHLVFTP------------PYVMLALKVGIITGVIALAEGIAVGRSF
AMY-KNYNIDGNKEMIAFGMMNILGSFSSCYLTTGPFSRSAVNYN-AGCKTALSNVVMAVAVAVTLLFLTPLFFYTPLVVLS
SIIIAAMLGLVD---YEAAIHLWKLDKFDFFVCLSAYLGVVFGT-IEIGLILSVGISVMRLVLFVGRPKIYVMGNIQNSEIYRNIEH
Y--PQAITRSSLLILHIDGP-IYFANSTYLRDRIGRWID-
---EEEDKLRTSGDIS-LQYIVLDMSAVGN--IDTSGISMLEELNKI
LGRR----ELKLVIANP-GAEVMKKLSKSTFI-ESIGKE-RIYLTVAEAVAACDFMLHTAKPD--SPVPEFNNV---------------------
----
--</seq-data>
</sequence>
<sequence seq-type="Protein">
<seq-name>O67306</seq-name>
<seq-info><length>1354</length></seq-info>
<seq-data>--
---MKRAEEVPGITVSFSIKFPFLMWFKNYSKE--GFIRDLIAGITVAAVYVP
QSMAYAMLA---GMPPIHGLYVAFIATIVAAIFGSSRYLNTGPVAMTCLLSASVLYGIGFEPQTP-------------------------------
----------------------EWIKYMALLALMVGLIRLTVGLFKLGFIVDLISNSVVVGFTAAGALVIALSQFKHFFGYEV---------KSST
HIFEVVMDLVSKI-------EMTNPYTLAIGVLAYFLIWGSRRI----------------------------SVYLPGALIAVVVTSLLVYWYK-------
----LYDKGVAIVGEVPQ-GLPSPEPPPLD-------------FAMMSKMWGGAFVVAFFGLIEAVAIAKTLAIR-VGDKWDPNQEL
IGQGLANVAVSFFKGFPAGGSFSRSSLNFA-LGAVSPLASVISGALVGLTLFLFAPAFYYLPKATLAAIVLSAVVNLIRP---QDIL
KLYRINKIDGVVAGLTFLSVFFMD-LWVAITLGVLLSLGSFVYKTMYPRIVTLTRDPVTRTFVNAEK----------------------------
--RGLPECPQIMFIRPNMS-IYFGNAQYVVYDYIMNKVE------------------------
--DALFNGRP-LKFVLIDMEAVNY--VDATGAETIVRLVKDIKQK----GVEVAFA
NI-GCDVYPILENAGFD-EVVNQD-LVFNAKGEAIGKLFEKLDHDYCREKCPYAVFDECLEVKPPEKVQELKKAS--------------
----
---</seq-data>
</sequence>
<sequence seq-type="Protein">

<seq-name>Q9U6Q2</seq-name>
<seq-info><length>1354</length></seq-info>
<seq-data>---
-----------------------MGAATAWDPRDQGVYTHCSGKKVKPTVSTLDCTRSWLQDCQRRTFNRKTLHKRLPILGWLPKYNS
Q--DAVGDLVAGITVGLTVIPQALAYAGIA---GLPVAYGLYASFVGCFVYIFLGSCKDVPMGPSAIVALLTYQAAQ-------------
---GSWQKSVLLCLLSGIVELLMGLFGLGFLIDFVSGPVSSGFTSAVSLIILTSQIQS
VLGITA---------KGNT-FVEIWTQVFHNI-------EHTRAGDTVLGLTCIVILLLMRSLSSCRIGPVDEKE----CSSFQRAVNKILWI
VGTARNAILVVVCCIMGYMLHT---------EEHGAPFRVVGEIPP-GLPSIQLPPTSLTANETSNGVAEGFVEMVHSMGSGLV
VIPLISLMENIAICKAFA---NGKPVDASQELIAIGTANIFNSFVQXFPGTGALSRGAVNNA-SGVRTPLSNIYSGGLVMTALLF
LTPYFYFIPRPTLAAIIISAVVFMIEV---KVVKPMWRSKKSDLVPGVGTFVACLVLP-LEWGILIGVGLNVIFILYHAARPKLTTE
LLTT--QSGVEYLMIT-PDRCLIFPSVD
YVRNLVN--KQSIRQ-NVPVVIDASHVYG--ADFTTATV
IDSLISDFNQR----GQLLFFYNL-KPSICSIFEHVSAA-QFVV---YYQEQQLDELLKERNYVQKRLETA-----------------------------
----
---</seq-data>
</sequence>
<sequence seq-type="Protein">
<seq-name>Q9URY8</seq-name>
<seq-info><length>1354</length></seq-info>
<seq-data>--MRAWGWVRNKFSSED
DYNDGASNKDYPDRFNEFDNSQNDHNDYTQNNAQFQNAQTTTFGRTISRVKAYYEIPEDDELDELASIPQWFKKNVTSNI
FKNFLHYLKSLFPIIEWLPNYNPY--WLINDLIAGITVGCVVVPQGMSYAKVA---TLPSEYGLYSSFVGVAIYCFFATSKDVSIG
PVAVMSLITAKVIANVMAKDETY--TAPQIATCLALLAGAITCGIGLLRLGFII
EFIPVPAVAGFTTGSALNILSGQVPALMGYKNK--------VTAKATYMVIIQSLKHL--------PDTTVDAAFGLVSLFILFFTKYMC
QYLGKRYPRW------------QQAFFLTNTLRSAVVVIVGTAISYAICKH---------HRSDPPISIIKTVPR-GFQHVGVPLIT-----------
---KKLCRDLASELPVSVIVLLLEHISIAKSFGRV-NDYRIVPDQELIAMGVTNLIGIFFNAYPATGSFSRSAIKAK-AGVKTPIAGI
FTAAVVILSLYCLTDAFYYIPNAILSAVIIHAVTDLILP--MKQTILFWRLQPLEACIFFISVIVSVFSS-IENGIYVSVCLAAALLLLRI
AKPHGSFLGKIQAANKY-GSDNIANV---RDIYVPLEMKEENPNLEIQS
PPPGVFIFRLQES-FTYPNASRVSTMISRRIKDLTRRGIDNIYVKDIDRPWNVPRQRKKKEN-------------------------------------
----------SEIEDLRPLLQAIIFDFSAVNN--LDTTAVQSLIDIRKELEIYA-NETVEFHFTNIRSGWIKRTLVAAGFGK--PKGHAVD
ASVCVEVAAPLRDANLSAESSRNLSRIITPI--YDDEEGNVSGHIYE-----------------LDGKNNSDLSMHCQKGSNQVEIEFVEF
NSRKYPFFHVDVASAVVDLQHRLLSPQKSDSFGSLKEGGTTTIKKIEN---
---</seq-data>
</sequence>
<sequence seq-type="Protein">
<seq-name>SULH_SCHPO</seq-name>
<seq-info><length>1354</length></seq-info>
<seq-data>---MSFIKKIKHYFADPADLPQDEAPDS
VPGLVPPPSNFVPVYPEPTYSDTVSKKGTIVRVVRHTASKLTKHGDSSQSLSTLPNNDISVGEYVEPLPSVPGWLKQNIFSHF
GTRLLHYLRSLFPIMNWLPRYNWN--WLVYDFIAGITVGCVVVPQGMSYAKVA---TLPAQYGLYSSFVGVAIYCIFATSKDV
SIGPVAVMSLVTSKVIANVQAKDPNY---DAAQIGTTLALLAGAITCGLGLLRL
GFIIEFIPVPAVAGFTTGSALNIMAGQVSSLMGYKSR-------VHTNAATYRVIIQTLQNL--------PHTKVDAAFGLVSLFILYLV

RYTCQHLIKRYTKF------------QRVFFLTNVLRSAVIIIVGTAISYGVCKH---------RRENPPISILGTVPS-GFRDMGVPVIS------
--------RKLCADLASELPVSVIVLLLEHISIAKSFGRV-NDYKVIPDQELIAMGATNLIGVFFHAYPATGSFSRSAINAK-SGVRTP
LGGIFTAGVVVLALYCLTGAFYYIPNAVLSAVIIHSVFDLIIP--WRQTLLFWRMQPLEALIFICAVFVSVFSS-IENGIYTAVCLS
AALLLFRIAKPSGSFLGILKIANKFDDDENSIDVV--RDIYVPLNQKGM
NPNLTVRDPPAGVLIFRLQES-FTYPNAGHVNSMLTSKAKTVTRRGNANIYKKASDRPWNDPAPRKKKNA-----------------
----------------------------PEVEDTRPLLRAIILDFSAVNH--IDTTGVQALVDTRKELEIYA-GDEVEFHFTDINNDWIKRTLVAA
GFGKARDATKYTSRSIEVGSAAPLRDIE-TPMAPGN-SRIVMPSSVRVRPFDEEEAIESSIPAIVSEDGADSDTISVSDDKDKK
VEGHRPSQDPTFSHHEYYPVISTTYPFFHVDVTSAVVDIQYRHVLDVNYKPKIVTNEVENENIEHSDGAA---------------------
----
</seq-data>
</sequence>
<sequence seq-type="Protein">
<seq-name>CY14_NEUCR</seq-name>
<seq-info><length>1354</length></seq-info>
<seq-data>---
--------------MASTSTGETVKKFLGIPADERLNDTACYVDGSFVESEPTTQDFLNEIRPTVQGTLNYLRELFPFVNWIFHYNLT
--WLLGDFIAGVTVGFVVVPQGMAYAKLA---NLAPEYGLYTSFVGFVLYWAFATSKDITIGAVAVMSTIVGNIIANVQKDHP
DF--DAGDIARTLAFISGAMLLFLGLIRFGFIVEFIPIVAISAFMTGSAISIAAG
QVSTLMGIPN--------INSREETYKVIINTLKGL--------PNTHLDAH-GLDRAIWAVLYPLVLYQMGKRYPRQ------------QRA
WFFVSTLRMVFIIILYILVSWLVNRHV-------KDPKKAHFKILGHVPS-GFQHKGAPRLD--------------NEILSAISGDIPTTILVL
LIEHIAISKSFGRV-NNYIINPSQELVAIGFTNLLGPFLGALPRYRIILENGHQGQ-SRCSHASRWHLYRRPRPARSLCAHLRLLL
HPQQRSRRHDHPRRRPDHSS---KGVYKFWLTSPLEVVIFFAGVFVSIFTS-IENGIYVTVAASGAVLLWRIAKSPGKFLGQTEI
YTAPRELVRGSKDSGRSRKACCRSRST---TAFLSLDRDDLSNPELQISTPWPGIFVY
RFGEG-LNYVNSAKHLDNLTIHVFKHTRRTELNKFEKLGDRPWNDPGPRRPSLPNR---
-RARFAPDPAAHHPRLLRRQLHR--CDRRP-RRLQDLRNQFDRYAHPDKVEWHFAGVSNRWTKRALVASGFGV----DSLRT
AKVQRENHKGGVQEVDQGPLVAIGPSVSASDIEAVVRSGSGSTDEKRPEGEGGATNGGMEKGSANGEDISTVPTATSAD
ACACGKGRWREEVGAGVWD--
---</seq-data>
</sequence>
<sequence seq-type="Protein">
<seq-name>SUL1_YEAST</seq-name>
<seq-info><length>1354</length></seq-info>
<seq-data>---MSRKSSTEYVHNQEDADIEV
FESEYRTYRESEAAENRDGLHNG------DEENWKVNSSKQKFGVTKNELSDVLYDSIPAYEESTVTLKEYYDHSIKNNLTAKSA
GSYLVSLFPIIKWFPHYNFT--WGYADLVAGITVGCVLVPQSMSYAQIA---SLSPEYGLYSSFIGAFIYSLFATSKDVCIGPVAV
MSLQTAKVIAEVLKKYPEDQTEV---TAPIIATTLCLLCGIVATGLGILRLGFLVELIS
LNAVAGFMTGSAFNIIWGQIPALMGYNSL-------VNTREATYKVVINTLKHL--------PNTKLDAVFGLIPLVILYVWKWWCG
TFGITLADRYYRNQPKVANRLKSFYFYAQAMRNAVVIVVFTAISWSITRN--------KSSKDRPISILGTVPS-GLNEVGVMKIP--
------------DGLLSNMSSEIPASIIVLVLEHIAISKSFGRI-NDYKVVPDQELIAIGVTNLIGTFFHSYPATGSFSRSALKAK-CNVRT
PFSGVFTGGCVLLALYCLTDAFFFIPKATLSAVIIHAVSDLLTS--YKTTWTFWKTNPLDCISFIVTVFITVFSS-IENGIYFAMCW
SCAMLLLKQAFPAGKFLGRVEVAEVLNPTVQEDIDAVISSNELPNEL-----------------------------NKQVKSTVEVLPAPEYKF
SVKWVPFDHGYSRELNINTTVRPPPPGVIVYRLGDS-FTYVNCSRHYDIIFDRIKEETRRGQLITLRKKSDRPWNDPGEWKM

PDSLKSLFKFKRHSATTNSDLPISNGSS---------------------NGETYEKPLLKVVCLDFSQVAQ--VDSTAVQSLVDLRKAVNRY
A-DRQVEFHFAGIISPWIKRSLLSVKFGT--TNEEYSDDSIIAGHSSFHVAKVLKDDVDYTDEDSRIST---------------------------
--------------------SYSNYETLCAATGTNLPFFHIDIPDFSKWDV---
---</seq-data>
</sequence>
<sequence seq-type="Protein">
<seq-name>SUL2_YEAST</seq-name>
<seq-info><length>1354</length></seq-info>
<seq-data>--MSREGYPNFEEVEIPDFQETNNTVPD
LDDLELEYDQYKNNENNDTFNDKDLESNSVAKHNAVNSSKGVKGSKIDYFNPSDVSLYDNSVSQFEETTVSLKEYYDHSIRS
HLTVKGACSYLKSVFPIINWLPHYNFS--WFTADLIAGITIGCVLVPQSMSYAQVA---TLPAQYGLYSSFIGAYSYSFFATSKDV
CIGPVAVMSLQTAKVIADVTAKYPDGDSAI---TGPVIATTLALLCGIISAAVGFLR
LGFLVELISLNAVAGFMTGSAFNILWGQVPALMGYNSL-------VNTRAATYKVVIETLKHL--------PDTKLDAVFGLIPLFLLY
VWKWWCGTYGPRLNDRYNSKNPRLHKIIKWTYFYAQASRNGIIIIVFTCIGWAITRG--------KSKSERPISILGSVPS-GLKEV
GVFHVP--------------PGLMSKLGPNLPASIIVLLLEHIAISKSFGRI-NDYKVVPDQELIAIGVSNLLGTFFNAYPATGSFSRSALK
AK-CNVRTPLSGLFSGSCVLLALYCLTGAFFYIPKATLSAVIIHAVSDLLAS--YQTTWNFWKMNPLDFICFIVTVLITVFAS-IED
GIYFAMCWSCAMLILKVAFPAGKFLGRVEVAEVTDAYVRPDSDVVSYVSENNNGISTLEDGGEDDKESSTKYVTNSSKKIET
NVQTKGFDSPSSSISQPRIKYHTKWIPFDHKYTRELNPDVQILPPPDGVLVYRLSES-YTYLNCSRHYNIITEEVKKVTRRGQLI
RHRKKSDRPWNDPGPWEAPAFLKNLKFWKKRENDPESMENAPSTSV---------------------DVERDDRPLLKILCLDFSQVA
Q--TDATALQSLVDLRKAINQYA-DRQVEFHFVGIISPWVKRGLISRGFGT--LNEEYSDESIVAGHTSYHVARVPQGEENPEK
Y---SVYTASGTNLPFFHIDIPDFAKWDI------------------------------------
----
-------------</seq-data>
</sequence>
<sequence seq-type="Protein">
<seq-name>DTD_MOUSE</seq-name>
<seq-info><length>1354</length></seq-info>
<seq-data>--MSSENKEQ
HDLSPRDLPEEAFGFPSELPLETQRRSGTDLRQSETGHGRRAFRRIHMELREKPDTDIKQFVIRELQKSCQCSAAKVRDGAF
DFFPVLRWLPKYDLK-KNILGDVMSGLIVGILLVPQSIAYSLLA---GQEPIYGLYTSFFASIIYFLFGTSRHISVGIFGILCLMIGEV
VDRELHKACPDTDATSSSIAVFSSGCVVVNHTLDGL--CDKSC----------------------YAIKIGSTVTFMAGVYQVAMGFFQV
GFVSVYLSDALLSGFVTGASFTILTSQAKYLLGLSLP------RSHGVGSVITTWIHIFRNI-------RNTNICDLITSLLCLLVLVPSKEL
NEHFKDK--------------------LKAPIPVELIVVVAATLASHFGK----------LNGNYNSSIAGHIPT-GFMPPKAPDW--------------
--SLIPNVAVDAIAISIIGFAITVSLSEMFAKK-HGYTVKANQEMYAIGFCNIIPSFFHCITTSAALAKTLVKES-TGCQTQLSAIVT
ALVLLLVLLVIAPLFYSLQKCVLGVITIVNLRGALLK--FRDLPKMWRLSRMDTVIWFVTMLSSALLS-TEIGLLVGVCFSMFCVI
LRTQKPKNSLLGLEEESETFESISTY---KNLRSKSGIKV
FRFIAP-LYYINKECFKSALYKKALNPVLVKAAWKKAAKRKLKEEMVTFRG---DPDEVS
MQLSHDPLEVHTIVIDCSAIQF--LDTAGIHTLKEVRRDYEAV----GIQVLLAQC-NPSVRDSLARGEYC-KKEEET-LLFYSLSEA
VAFAEDSQNQKGVCVVNGLSLSGD---

-------------------------------</seq-data>
</sequence>

<sequence seq-type="Protein">
<seq-name>DTD_RAT</seq-name>
<seq-info><length>1354</length></seq-info>
<seq-data>---MSSENKEQ
HNLSPRDLPEEAYGFPPELPLGAQRGSSTDLRQFEPSDRRRAFRRIHMELHEKPDTNIRQLVMRKLQKSCQCNATKIRNRIF
DFFPVLRWLPKYDLK-KNILGDMMSGLIVGILLVPQSIAYSLLA---GQEPIYGLYTSFFASIIYFLFGTSRHISVGIFGILCLMIGE
VVDRELHKACPDIDTTSSSIAMFSNGCVVVNHTLDGL--CDKSC-----------------------YAIKIGSTVTFMAGVYQVAMGFFQ
VGFVSVYLSDALLSGFVTGASFTILTSQAKYLLGLSLP------RSNGVGSVITTWIHIFRNI-------HKTNICDLITSLLCLLVLVPTK
ELNEYFKSK---------------------LPAPIPTELIVVVAATLASHFGK----------LNENYNSSIAGQIPT-GFMPPQAPDW------------
----SLIPNVAVDAIAISIIGFAITVSLSEMFAKK-HGYTVKANQEMYAIGFCNIIPSFFHCITTSAALAKTLVKES-TGCQTQLSAIV
TSLVLLLVLLLIAPLFYSLQKCVLGVITIVNLRGALLK--FRDLPKMWRLSRMDTVIWFVTMLSSALLS-TEIGLLVGVCFSMFCV
ILRTQMPKISLLGLEEESEIFESISTY--KNLRSKSGIKV
FRFIAP-LYYINKECFKSALYKKTLNPVLVKAAWKKAAKRKLKEETVTFHG---DPDEVS
MQLSHDPLELHTVVIDCSAIQF--LDTAGIHTLKEVRRDYEAI----GIQVLLAQC-NPSVRDSLAKGEYC-KKEEEN-LLFYSLSEA
VAFAEESQKEKGVCVVNGLSLSGD--

------------------------------</seq-data>
</sequence>
<sequence seq-type="Protein">
<seq-name>DTD_HUMAN</seq-name>
<seq-info><length>1354</length></seq-info>
<seq-data>---MSSESKEQH
NVSPRDSAEGNDSYPSGIHLELQRESSTDFKQFETNDQCRPYHRILIERQEKSDTNFKEFVIKKLQKNCQCSPAKAKNMILGF
LPVLQWLPKYDLK-KNILGDVMSGLIVGILLVPQSIAYSLLA---GQEPVYGLYTSFFASIIYFLLGTSRHISVGIFGVLCLMIGETV
DRELQKAGYDNAHSAPSLGMVSNGSTLLNHTSDRI--CDKSC-----------------------YAIMVGSTVTFIAGVYQVAMGFFQV
GFVSVYLSDALLSGFVTGASFTILTSQAKYLLGLNLP------RTNGVGSLITTWIHVFRNI-------HKTNLCDLITSLLCLLVLLPTKE
LNEHFKSK---------------------LKAPIPIELVVVVAATLASHFGK----------LHENYNSSIAGHIPT-GFMPPKVPEW--------------
--NLIPSVAVDAIAISIIGFAITVSLSEMFAKK-HGYTVKANQEMYAIGFCNIIPSFFHCFTTSAALAKTLVKES-TGCHTQLSGVV
TALVLLLVLLVIAPLFYSLQKSVLGVITIVNLRGALRK--FRDLPKMWSISRMDTVIWFVTMLSSALLS-TEIGLLVGVCFSIFCVI
LRTQKPKSSLLGLVEESEVFESVSAY--KNLQTKPGIKI
FRFVAP-LYYINKECFKSALYKQTVNPILIKVAWKKAAKRKIKEKVVTLGG---IQDEMS
VQLSHDPLELHTIVIDCSAIQF--LDTAGIHTLKEVRRDYEAI----GIQVLLAQC-NPTVRDSLTNGEYC-KKEEEN-LLFYSVYEA
MAFAEVSKNQKGVCVPNGLSLSSD--

-------------------------------</seq-data>
</sequence>
<sequence seq-type="Protein">
<seq-name>SAT1_RAT</seq-name>
<seq-info><length>1354</length></seq-info>
<seq-data>---
----------------------MDASPEPPQKGGTLVLVRRQPPVSQGLLETLKARLKKSCTCSMPCAQALVQGLFPVIRWLPQYRLK-E
YLAGDVMSGLVIGIILVPQAIAYSLLA---GLQPIYSLYTSFFANLIYFLMGTSRHVNVGIFSLLCLMVGQVVDRELQLAGFDPS

QDSLGPGNNDSTLNNTATLTVGLQDCGRDC-----------------------HAIRIATALTLMAGLYQVLMGILRLGFVSTYLSQPLLD
GFAMGASVTILTSQAKHLLGVRIP------RHQGLGMVIHTWLSLLQNV-------GQANLCDVVTSAVCLAVLLTAKELSDRYRH
Y----------------------LKVPVPTELLVIVVATIASHFGQ----------LHTRFGSSVAGNIPT-GFVAPQIPDP----------------KIMWSV
ALDAMSLALVGSAFSISLAEMFARS-HGYSVSANQELLAVGCCNVLPAFFHCFATSAALSKTLVKIA-TGCQTQLSSVVSAAV
VLLVLLVLAPLFHDLQRCVLACIIVVSLRGALRK--VKDLPQLWRLSPADALVWVATAATCVLVS-IEAGLLAGVFFSLLSLAGR
TQRPRAALLARIGDSTFYEDAAEF---EGLLPPPEVRV
FRFTGP-LYYANKDFFLRSLYSLTGLDAGYSATRKDRGTEVGVSNRSLVDRKDLG--SVSS
GDGLVVPLAFGFHTVVIDCAPLLF--LDVAGMATLKDLRKNYRAL----DITLLLACC-SPSVRDTLRKGGFLGEDQGTAEELLFP
SVHSAVETACARREELMAADSAL--

----------------------------------</seq-data>
</sequence>
<sequence seq-type="Protein">
<seq-name>DRA_HUMAN</seq-name>
<seq-info><length>1354</length></seq-info>
<seq-data>---
-------------------MIEPFGNQYIVARPVYSTNAFEENHKKTGRHHKTFLDHLKVCCSCSPQKAKRIVLSLFPIASWLPAYRLK-E
WLLSDIVSGISTGIVAVLQGLAFALLV---DIPPVYGLYASFFPAIIYLFFGTSRHISVGPFPILSMMVGLAVSGAVSKAVPDRNA
TTLGLPNNSNNSSLLDDER----------------------------------VRVAAAASVTVLSGIIQLAFGILRIGFVVIYLSESLISGFTTAAAVH
VLVSQLKFIFQLTVP------SHTDPVSIFKVLYSVFSQI-------EKTNIADLVTALIVLLVVSIVKEINQRFKDK---------------------LP
VPIPIEFIMTVIAAGVSYGCD----------FKNRFKVAVVGDMNP-GFQPPITPDV----------------ETFQNTVGDCFGIAMVAFA
VAFSVASVYSLK-YDYPLDGNQELIALGLGNIVCGVFRGFAGSTALSRSAVQES-TGGKTQIAGLIGAIIVLIVVLAIGFLLAPLQ
KSVLAALALGNLKGMLMQ--FAEIGRLWRKDKYDCLIWIMTFIFTIVLG-LGLGLAASVAFQLLTIVFRTQFPKCSTLANIGRT
NIYKNKKDY---YDMYEPEGVKIFRCPSP-IYFANIGFF
RRKLIDAVGFSPLRILRKRNKALRKIRKLQKQGLLQVTPKGFICTVDTIKDS--DEELDNNQIEVLDQPINTTDLPFHIDWNDDL
PLNIEVPKISLHSLILDFSAVSF--LDVSSVRGLKSILQEFIRI----KVDVYIVGT-DDDFIEKLNRYEFF-DGEVKSSIFFLTIHDAVLH
ILMKKDYSTSKFNPSQEKDGKIDFTINTNGGLRNRVYEVPVETKF---

--</seq-data>
</sequence>
<sequence seq-type="Protein">
<seq-name>Q9WVC8</seq-name>
<seq-info><length>1354</length></seq-info>
<seq-data>---
-------------------MIEAIGNQYVVARPVYSTKTFGEEFKKTHRHHKTFLDHLKGCCSCSSQKAKKIALSLFPIASWLPAYKIK-E
WLLSDIVSGISTGLVAVLQGLAFALLV---NIPPAYGLYAAFFPVITYFFLGTSRHISVGPFPVLSMMVGVVVTRVVSD--PNAS
SELSSSSTENDSFIE---------------------------------------EKVMVAASVTVLSGIIQLLLGVLQVGFVVIYLSESLISGFTTAAAIHVL
VSQLKFMLQLPVP------AYSDPFSIFKVLESVFTQI-------QKTNIADLVTSVIILVVVFVFKEINQRYRSK---------------------LP
VPIPIELIMTVIATGVSYGCN----------FEDRFGVAVVGNMSL-GFQPPITPSV----------------EVFQDTIGDSFGIAIVGFAVAF
SVASVYSLK-YDYPIDGNQELIALGVSNIFTGAFKGFAGSTALSRSGVQES-TGGKTQVAGLLSAVIVLIVIVAIGFLLQPLQKSV
LAALALGNLKGMLMQ--FAEIGRLWKKDKYDCLIWIMTFIFAIVLG-LGLGLAASVAFQLLTIVFRTQFPKCSTLANVGRSNIY
KNKKNY---AEVYEPEGVKIFRCPSP-IYFANIGFFKQK

LIDAVGFSPLRILRKRNKALKKIRKLQKRGLIQMTPKGFICTSDGFKDS--DEELDNNQIEELDQPINTTDLPFDIDWNGDLPL
NITIPKISLHSLILDFSAVSF--LDVSSMRGLRTILQEFIRI----KVDVYIVGT-DDDFIDKLARCEFF-DDEVTDSIFFLTIHDAILHIL
MKKDYSTSKFNSSQEKERKFDFTINTNGGLRNRECQVPVETKF---

---------------------------------------</seq-data>
</sequence>
<sequence seq-type="Protein">
<seq-name>PEND_HUMAN</seq-name>
<seq-info><length>1354</length></seq-info>
<seq-data>---
--------MAAPGGRSEPPQLPEYSCSYMVSRPVYSELAFQQQHERRLQERKTLRESLAKCCSCSRKRAFGVLKTLVPILEWLPK
YRVK-EWLLSDVISGVSTGLVATLQGMAYALLA---AVPVGYGLYSAFFPILTYFIFGTSRHISVGPFPVVSLMVGSVVLSMA---
-PDEHFLVSSSNGTVLNTTMIDTAARDT------------------------------ARVLIASALTLLVGIIQLIFGGLQIGFIVRYLADPLVGGF
TTAAAFQVLVSQLKIVLNVSTK------NYNGVLSIIYTLVEIFQNI-------GDTNLADFTAGLLTIVVCMAVKELNDRFRHK-------
-------------IPVPIPIEVIVTIIATAISYGAN----------LEKNYNAGIVKSIPR-GFLPPELPPV----------------SLFSEMLAASFSIAV
VAYAIAVSVGKVYATK-YDYTIDGNQEFIAFGISNIFSGFFSCFVATTALSRTAVQES-TGGKTQVAGIISAAIVMIAILALGKLL
EPLQKSVLAAVVIANLKGMFMQ--LCDIPRLWRQNKIDAVIWVFTCIVSIILG-LDLGLLAGLIFGLLTVVLRVQFPSWNGLGS
IPSTDIYKSTKNY--KNIEEPQGVKILRFSSP-IFYGNV
DGFKKCIKSTVGFDAIRVYNKRLKALRKIQKLIKSGQLRATKNGIISDAVSTNNAFEPDEDIED-LEELD--IPTKEIEIQVDWNS
ELPVKVNVPKVPIHSLVLDCGAISF--LDVVGVRSLRVIVKEFQRI----DVNVYFASL-QDYVIEKLEQCGFF-DDNIRKDTFFLTV
HDAILYLQNQVKSQEGQGSILETITLIQDCKDTLELIETELTEEELDVQDEAMRTLAS---
----
--</seq-data>
</sequence>
<sequence seq-type="Protein">
<seq-name>YLDM_CAEEL</seq-name>
<seq-info><length>1354</length></seq-info>
<seq-data>---
----------------------------------MSLRQLFSDRPPLNAVLQEKAQKLRY--ACSPSKCIHSLLSFLPIITWLPKYDWS-HSFFGDLSG
GLTMAVFSVPQGIALASIT---GVPPVYGLYTAIFPSFLYIFFGTSKHNALGGFAVLSLMTHGAIEKVMLRTATSYNATAYVNH
TLDELLDKENETALISNTTLMQILGNETSFVEEVTMEMWTEGVTPVKQIHVATTIIFLAGVIQVFMGVFRLQYLTSLFSEQV
MSGFVVGGGIHVFFAQIGNMLGIELP------RRSGPGYLYYRIWDLVENL-------DNVHIPTVCISLSSFLFLVFGKEYLAPWLN
SA--------------------FNYPVPFELVLLTHYLINR-------------------------------FPPPSLPRF----------------DLIRHIGLNAAAIAIT
AVAIHITVAKVVEKR-YKYKINHGQELYALGFVGVLSSFFPVFPVTSGFARSVVGAA-VGGSTQLTCLFSSLALLSVILCIGPALE
YLPQCILSAMIIFAQKGMLEK--FGELKSLWPVFKIDFTIWLMSFFLTVCYD-MGEGLLMAIGFAVLTTIIRTQRPKWHFLSRD
DDTENYKETKKR--DLERIQGNVCIFRMDAP-LIFTSS
DRFTMSVWQCVKKWERCKSESFVTIEQMNSDRSADIFDSKLKSARRRWKR---DQKSEN
-RCKLVIDCDGFPY--VDYLGLSTLKSVYVDLQAA----GIQCFFVVQ-KSDLKKLFRATDFY-EVVDES-KVFNKVGDAVKAAEQ
HISSPKTTKEILTALASIATTDTVLIDEESSDSNDNDDAEIQERITEESENSEEVMSETSVSIEDATSLTSSRNSINSEE------------
----
--</seq-data>
</sequence>

<sequence seq-type="Protein">
<seq-name>O17951</seq-name>
<seq-info><length>1354</length></seq-info>
<seq-data>---MIH
VEEEEYDQLRSFY----AKKRRA-----PMNQVEYDEKYGYQKRLKDGGKFKKRSTKVASRYYVPFTSVTNFKIFLLNLFPIFGWL
PKYDWK-NSLTSDVVGGITVGVLQIPQGIAYAILS---RQDPIVGLYTSIYPVFLYIFFGTSKHASLGTFAVVALMTGLAVEREAF
IPSDNLNSTLLPG--DEALP-----------------------------------SPIEVSCALVLGVGLIQFLMGVFRLQFLTTYLSDQLIAGFTT
GSAVHVLVSQFKELFGLRGLV-----KHSGPGYLIRNVYDTVTNL-------PKANFMCCAISLATMILLHCGKEYINPIMKRKMK--
---------------SNIPIPWELVAVIISTIFVALID----------ANELYNVKIVNKIPT-GLPELSLPNP---------------NLIPRVLPDAISIA
VVVVAVHLSLSKMLAKK-YEYEIDAGQELYALSFTAIGGSFFPTFPTSIGLGRTMVGVE-SGVKTQVATFFSCLFVLSVSLYFGR
FLETLPMCVLSAIIVIALKSMLWK--LRDLKGIWKLSKIDCCIWMVAFFATVLVD-VSEGLLIAIFFALFTTILREQYPKWHLLAN
VKDTDEFSDTQQY---QETIFYKGICIFKFDAP-LLFHN
VECFKKCIEKVYDEWKKSSEFNFVKEPNAGKGSKFTFEGMHRIAPITEIPLH-----------------------------------PGINRDPILP
RHFVIDCSGFTF--IDLMGVSALKEVFSDLRKK----RVQVYFAST-KVPVREMFEKCSFF-DFVSKE-NFYPTLRDATGIARLRQT
E---

----</seq-data>
</sequence>
<sequence seq-type="Protein">
<seq-name>O17950</seq-name>
<seq-info><length>1354</length></seq-info>
<seq-data>--MAPPGRINH
ARHLSEAEEEAENYLEHFYNKSEPNKRRVSFVQRGAMNQAQFDEKFDYNKPHLENELKKQAKKFVRRFYEPFTSFFALKLFI
FDLIPILKWFPEYKWK-TDLSLDIIGGITVGVMQVPQGIAYALLA---KQPAINGLYTSLFPPLIYMLFGTSRHASLGTFAVVSL
MTGLSVEKLAAPTDYDPSSFNETDIDLVKLP-----------------------------------SPTEVSCAITITMGLILFVMGVLRLQFLTT
YLSDQVIAGFTVGSSVHVLVSQLKTLLGIRGLP-----RHSGPFYLFRHLYDLVMSI-------TRANPVSCGISIVSIIVLHFGKEFINP
FIKRKTK-----------------SNIPIPWELVIVILSTVFVAVTG----------VDTEAKVQVVNKIPV-GVPNFSLPSF---------------YLIP
QVLPDAISITVVSISVWLSISKMLAKR-YNYELDSGQELFALSFASISSSFIPSIPNSCSLSRTLVAVG-AGCTTQLSIFFSSLIVFSV
VIFLGTLLETLPMAALSAIICVALQGMFRK--FADLIDLWKVSKIDFTIWVVSCVSTILLD-VSTGLIISVCFALFTTILREQYPKW
HLLASVKGTQDFRDAERY---GETVYFKGICIFRFDAP-
LLFHNVECFKKSIEKAYTEWQKSHEFYVLREERETILNTKLDGSDESIDGKMFQTAQST-------------------------------LNT
HSPDILSRHFVIDCSGFTF--IDLMGVSALKEIFSDMRKR----GILVYFANA-KAPVREMFEKCHFF-NFVSKE-NFYPTMRDAT
SIARQRQLELGFKDTGYVPEHDRLTEVLSSHPM---

------------------------------</seq-data>
</sequence>
<sequence seq-type="Protein">
<seq-name>Q19447</seq-name>
<seq-info><length>1354</length></seq-info>
<seq-data>--
------------------------------MKQEEYDQMYSFNREGNETWVKKKINNYCTFLSKDGIRKIILRRVPVIDWIGSYQIN--NFASD
FIAGLTMGVYNVPQAMSYSILA---GLPPVYGLYASFFPPFLYSIFGSAKHSSIGVFSITCLMVDKCVKKMLKFRNENPESEL---

---ISIFSDKLYNFWAQNSPAVMAIFRCDKPMKFLGAPAISAITFSACFFGVVSQIPKL
CGFSVPS-----RNEHWFSLVHSILDIFENC-------HKSNTATLCISASALVFLIGSRIFIEPFFKNHKKL--------------------QSIPFPKE
LITIVIATSASYFFD----------FEHRFGVKTLHTVPR-GFPYPDVPRI------------------DIWPYIFQDALSIAVVAYAVTMAMGQEF
ATK-HRYRIDSNQELLALGFINIGSSFFSVFPTSASFSRTLVNER-SGAKTQLSGITSACFMALVITTIGPYLASLPSCILSAIVIVVL
ESMLRK--CTVLPGLWRCSKHDFWIWIITAVVTLSSD-IAQGVAAGIFTAIFTIAIQSQQPTIKLLGQI-RPNDFRPMNHY------
---SLAKETS-FRIIRFDAP-LIFANVEKFLDNVRDSTSATTKK
RS---ASCDSQEDIEWSAIILDCHTWIY--TDSMGIAAVKQINEEL
RQI----RILLLFANL-KSSLRRQYGNAGLIGEGNIGRNQMYPSIQDALDSAHELIKNHKMFFDCEKSMPPEKIVP-----------------
----
---</seq-data>
</sequence>
<sequence seq-type="Protein">
<seq-name>Q23454</seq-name>
<seq-info><length>1354</length></seq-info>
<seq-data>--
---MKEVYQPCTTTDQDIVEKKECSP--FRKKLQKYIPILEWLPNYQWK-DHFHGDVIAGL
TVGIMHVPQGMAYASLA---GVPPVYGMYSSFFASTIYMFFGTARHISIGVFAVASMMVGAARLRLAPDIPISNSSDINPSV
YPLGEYV---DPLVFTSALTLLVGVVQIIMGILRLGFLTTYLSDSLVSGFTTGAAVHVFTSQLN
KVFGIKLP------RHEGIGMIVRMYRDMIMSL-------GSVNFVALGISIFGILFLDLGRTYINPIVKKF-----------------------SPIPPPL
ELILVIFGIVISMIFN----------LDAEYHVKTVYEIPR-GFPLPSIPRL------------------NFLPALLSDAIPIAVVCYMFVMSMGKLFA
KK-HKYKTDATQELYAIGIASALSSFFPVYPVGASLSRSSVCEM-SGANTQLYTIFSSFLLLTVILLLGPFLEPLPMCILACIVIVSL
KSLFMQ--VKELPRLYRISKYDFAIWLVACLSTIFTD-VTTGLVISLAFSLYTLVLRQQWPSFSTVLHDET-----------------------------
---PRQNVPENVKIVKFAGS-LHFANVTAFQDDMGEAIG-------------
---KLPEEDPLIDERTIILDASSVAF--IDIMGVDALRDVFKDALKL----G
VHVFYCGL-PEDVLSVLSNDENF-LSVVPPSTFFPSIDSCLISFVHQQSV---

---</seq-data>
</sequence>
<sequence seq-type="Protein">
<seq-name>Q9S6X8</seq-name>
<seq-info><length>1354</length></seq-info>
<seq-data>--
---MNNSFTLKQQWFGN---IRKDILAGILVALALIPEAIGFSII
A---GVDPMVGLYASFCIAIISIFGGRPGMISAATGSMAVVMVSLVAD--
----HGLQYLFAATILTGIIQVILGISKIARLMKFIPRSVMIGFVNALAILIFSAQLPQFEG--ASW
SMYAMLAGSLVIIYVLPRFT------------------------------------TAVPSPLVAIIVMTIIAVTFHVDV------------RTVGDMGNISS-SLPHF
LIPDVPF--------------TFETLQIIFPYSIALAFVGLLESLLTAQIIDEM-TDTDSDKNKESRGQGIANIVTGFFGGMAGCAMIGQS
VINTK-AGGRGRLSAFVAGAFLMFLIAVLSHVVVKIPMAALVAVMVMVSVGTFDWSSL---KGLKKAPLTDSIVMVVTVVT
VVVTDDLSKGVFVGVLLSAVFFVAKISKLKIVSHA---
--------------------EDQKLRTYQVKGQ-IFFASVTDLTNAFIYQE--
--------------DIERVVIDLTEAHV--WDDSGAAALDKIVAKFKEQ----GIEAELKGL-NKASKSLMKQMA------------------------------
----

----</seq-data>
</sequence>
<sequence seq-type="Protein">
<seq-name>Q9RBV5</seq-name>
<seq-info><length>1354</length></seq-info>
<seq-data>--
--MLHSLKQTWLSN---IRGDILAGLVVALALIPEAIAFSIIA
---GVDPKVGLYASFCIAVVIAFVGGRPGMISAATGAMALLMVTLVKN---
----HGLEYLLAATLLCGVLQIAAGYLKLGSLMRFVSRSVVTGFVNALAILIFMAQLPELTN-------------------------------------V
TWHVYAMTAAGLGIIYLFPYVPKIG-------------------------KLIPSPLVCIIVLTAVAMSVGLDI-----------RTVGDMGELPD-T
LPIFLWPDVPL------------TFETLAIIFPYSAALAVVGLLESMMTATIVDDL-TDTPSDKNRECKGQGVANIASGLIGGMAGCA
MIGQSIINVK-SGGRSRLSSLAAGVFLLLMVVFLGDWLKQIPMAALVAVMIMVSIGTFSWDSL---RNLKKHPLSTNIVMVV
TVVVVVATHNLAFGVLAGVLLAAMFFANKVGHYMAISSSL--
--------------------------DEAGEHRSYNVTGQ-VFFSSADKFVAAFDFKE---
--------------------------ALNKVTIDLNRAHF--WDITAVAALDKVVIKFRRE----GTEVEVLGL-NEASATIVDRFGVHDKPDAI
DQLMGH--

-----------------------------</seq-data>
</sequence>
<sequence seq-type="Protein">
<seq-name>O07488</seq-name>
<seq-info><length>1354</length></seq-info>
<seq-data>--
---MLLSSTRKDWLGN---VRGDVLAGIVVALALIPEAIAFSII
A---GVDPQVGLYSAFCIPLVMAFFGGRPAMISSSTGAMALLMVTLVKD---
------HGLQYLLAASILTGVFQLIAGYLKLGGLMRFVSRSVVTGFVNALAILIFMAQLPELTN-------------------------------------
VTWHVYAMTAAGLGIIYLFPYIN----------------------------KTIPSPLVCIVVLTGIAMWLHLDV-----------RTVGDMGKLPD-
SLPVFLLPDVPL------------NLQTLLIILPYSAGLAVVGLLESMMTATIVDDM-TDTPSDKNRECKAQGIANICTSFIGGMAGC
AMIGQSVINVK-SGGRGRLSTLTAGVVLLCLIVFLRDWVSQIPMAALVAVMIMVSIGTFSWRSI---ANLRTHPLSTSVVMLA
TVAVVVATHNLAFGVLTGVLIASLNFATKVSRFMRVTSVL--
--------------------------EGTSRTYTVTGQ-VFFASADRFTSHFDFRE---
---------------------AIENVVIDVSHAHF--WDITSVSALDKVVIKFRRE----GAGVEIRGM-NEATRTIVDRFGVHDKPEEVEKL
MGGH--

------------------------</seq-data>
</sequence>
<sequence seq-type="Protein">
<seq-name>P73474</seq-name>
<seq-info><length>1354</length></seq-info>
<seq-data>--
--MINWTILKREWFRN---TKEDLLAGAVVGLALIPEAIAFSI

IA---GVDPKVGLYASFTIAVLTAFFGGRSGSISAATGAMALLMVDLVKD--
-----HGLQYLLAATVLTGIFQVLLGVFKVAKQMRYVPRAVVLGYINALAVLIFLAQLPQLDPTKVS------------------------------
---QYWLVYLILACSLAIIYILPTFT----------------------------KVFPSPLVALFVMTNATIFLQLDV-----------PTVGDMGELPG-S
LPIFLLPQIPF------------NWQTLQIILPYSLTLAIVGLLASFLTASLVDEL-TDTPSDKNREAKGQGIANIVTGFFGGMAGCGMI
GQSVINVQ-SGGRGRLSTFAAGIFLLIAILGLKDWVQQMPMATLVAVMIMVSIGTFRWSSL---RDFRKIPQSENIVMFTTM
GLIIITRNFALGVAAGIIMSTIFFTRKIAQLVFVDRVL---
----------------TEDENHRIYNVSGQ-IFFVSKEEFLEAFDFDE---
-------------LVDRVTIDLTHAHL--WDQGAVGTVEQIMTKFRRN----GIDVELVGL-NGASATLWQKLIKEPELKLINSSAEN--
----

--------------</seq-data>
</sequence>
<sequence seq-type="Protein">
<seq-name>Q9X8E9</seq-name>
<seq-info><length>1354</length></seq-info>
<seq-data>---
---MTPTTPAARLRALKPDWLNDPKVWRTEILAGLVVALALIPE
AISFSIIA---GVDPAIGLFASFTMAVTISIVGGRRAMISAATGAVALVIAPMNRE---
------------HGLGYLIATVILAGVFQIVLGAVGVAKLMRFVPRSVMTGFVNALAVMIFMAQVPEMHD-----------------------
-------------VPWPVYPLIVGGLALMVFFPKVT----------------------------RVVPAPLVSIVILTVITVAAGIAV-----------PTVGD
KGELPS-SLPVPGLPDVPF------------TLDTLTTVAPYALAMALVGLMESLMTARLVDDI-TDTHSSKTRESIGQGVANIVTGF
FGGMGGCAMIGQTMINVKVSGARTRVSTFLAGSFLMVLCIVFGPVVSDIPMAALVAVMVMVAFGTFDWHSVAP-KTLK
RMPAGEIAVMIITVIVVVATDNLAIGVVVGTVTAMVVFARRVAHLAQVTAVT---
---DPDGTTVVYRVTGE-LFFASSNDLVGQFDYAG--------------------------------------
---DPDRVVIDLSAAHI--WDASSVAALDAIETKYGQR----GKSVEITGL-NTPSADLH
GRLTGELTGSH--

--</seq-data>
</sequence>
<sequence seq-type="Protein">
<seq-name>Q50443</seq-name>
<seq-info><length>1354</length></seq-info>
<seq-data>---
---MSITLPGLRAPSSGNGLASLRSPSV---LRTEVLAGLVVALALIPEAI
AFSVIA---GVDPRVGLFSTVTMAITIAFTGGRPAMITAATAAVALVVAPVARE--
------------FGVGYLVATILLAGVIQMLLALFGVAKLMRFIPRSVMVGFVNALAILVFVSQLQHLVD-----------------------------
--------VPWLVYALTAVGIAVMFLLPKVT----------------------------TAVPAPLVVVLVLTVAVVAFGWDV-----------PDVGD
QGALPD-SLPVPGLPHVPL------------TLHTLQVIAPYAFGAALVGLLESLMTAKLVDDI-TDTPSNKTREACGQGTANVVTAL
FGGMGGCAVVGQTMMNVKVAGARTRLSTLLTGVFVLILVLALGDLVGRIPMAALVAVMIVVSFATFDWHSITP-RTLKVL
PRSETLIMLITVACTVATGNLAIGVSVGVLAAMVAFARRVAHFTSVSAE---
--PGPDDSRTYRVRGD-LFFVSSNDLVHQFDYAG--
--DPDEVVIDMSGVSL--WDASSVATLDAIRLKYSSR----GKTVAFTGL-DGESLDRLNRM

SGRLGV---

--</seq-data>
</sequence>
<sequence seq-type="Protein">
<seq-name>O06984</seq-name>
<seq-info><length>1354</length></seq-info>
<seq-data>--
--MRFSGRFKGYNLQ--KFQKDLIAGIVVGVVAIPLGMAF
AIAS---GVEPEYGLYTVVIAGICISLFGGSKYQIGGPTGAFVPILFGIIMQ---
----YGLENLLIAGFMAGVMLVLFGLFKLGKIMKFVPKPVIVGFTAGIAVLIFTEQIANFLGLRNV--------EKHENFHHNMFEIV
QQL-------GTFNVYAILTAVIGLVILLVSAKVM-------------------------------PKVPGALLALLISTVVAVVFFP---------DRMATIG
STYGEIPR-HLPEFQFPEL--------------TLDKMVMLFPAALVIALLGGLESILSAMVADNM-KGSKHDSNKELVGQGIANMA
APLFGGIPATGAIARTATNIK-NGAVSPVSGVVHGVVVLLVLLVFAPYASHVPLASMAPILMVVAWNMSERK---EVANML
RLKNADSFILAATFALTVLFD-LIIGVATGLLLAFVFFIRRMSEATRIHNQET--
-------------------------------------HPVLAKREDPSVSMYAIEGP-LFFGSIDSLESSLLEHVQKK--
--PKTLILLMNKVHY--MDTSAEAVLGNIMNRIKRH----NGKLMIVGL-QSQPKELLHK
TGLF-HKIGKQ-HFFDHHDEITG--

--</seq-data>
</sequence>
<sequence seq-type="Protein">
<seq-name>YCHM_ECOLI</seq-name>
<seq-info><length>1354</length></seq-info>
<seq-data>--
--MPFRALIDACWKEKYTAA--RFTRDLIAGITVGIIAIPLAMAL
AIGS---GVAPQYGLYTAAVAGIVIALTGGSRFSVSGPTAAFVVILYPVSQQ--
-------FGLAGLLVATLLSGIFLILMGLARFGRLIEYIPVSVTLGFTSGIGITIGTMQIKDFLGLQM--------AHVPEHYLQKVGALF
MAL------PTINVGDAAIGIVTLGILVFWPRLGIRLP----------------------------GHLPALLAGCAVMGIVNLLGGHVA-TIGSQFH
YVLADGSQGNGIPQ-LLPQLVLPWDLPNSEFTL------TWDSIRTLLPAAFSMAMLGAIESLLCAVVLDGM-TGTKHKANSEL
VGQGLGNIIAPFFGGITATAAIARSAANVR-AGATSPISAVIHSILVILALLVLAPLLSWLPLSAMAALLLMVAWNMSEAHK--
VVDLLRHAPKDDIIVMLLCMSLTVLFD-MVIAISVGIVLASLLFMRRIARMTRLA---
--PVVVDVPDDVLVLRVIGP-LFFAAAEGLFTDLESRL------------------------------------
---EGKRIVILKWDAVPV--LDAGGLDAFQRFVKRLPE-----GCELRVCNV-EFQPL
RTMARAGIQ-PIPGRL-AFFPNRRAAMADL---

---</seq-data>
</sequence>
<sequence seq-type="Protein">
<seq-name>O84864</seq-name>
<seq-info><length>1354</length></seq-info>
<seq-data>--

--MVKVSLSFKHLVPKLVTCLKEGYSFN--TLKKDFTAGITAGILAFPLA
IAIAIGI---GVSPLQGLLASIIGGFLASALGGSRVLISGPTSSFISILYCIGVK--
----YGEDGLFTITLMAGIFLIIFGLAGLGTFIKYMPYPVVTGLTTGIAVIIFSSQIRDFLGLQMG-------DGVPLDFIGKWAAYW
DYL-------WTWDSKTFAVGLFTLLLMIYFRNYK----------------------------PRYPGVMISIIIASTLVWILKIDI----------PTIGSR
YGTLPS-SLPGPVFPHI--------------SITKMLQLMPDALTISVLSGIETLLAAVVADGM-TGWRHQSNCQLIGQGIANIGTSLF
AGMPVTGSLSRTTASIK-CGASTPIAGIIHAICLSFILLLLAPLTIKIPLTCLAAVLILIAWNMSEIH---HFIHLFTAPKKDVVVLLTV
FILTVMTT-ITSAVQVGMMLAAFLFMKQMSDLSDVISTAKYFDESEQPQNDL---
-----------------------------LFSKNEVPPFTEIYEINGP-FFFGIADRLKNLLNEIEKP--
----------------------------------PKIFILCMTRVPT--IDASAMHALEEFFLECDRQ----GTLLLLAGV-KKTPLSDLRRYHVD-ELIG
VD-HIFPNIKGALLFAKALIKLESKSSQ---

--</seq-data>
</sequence>
<sequence seq-type="Protein">
<seq-name>Q9Z6P5</seq-name>
<seq-info><length>1354</length></seq-info>
<seq-data>--
---MKVPWAFKNFIPKLYTSIKEGYSFN--TFKKDFQAGITVGILAFPFA
IAIAIGV---GVSPIQGLLASIIGGLLASAMGGSNVLISGPSSAFISILYCLSAK---
-------YGAEALFTVTLLAGVFLIAFGLTGLGTFIKYMPYPVVTGLTTGLAIIIFSSQIKDFLGLQMG-------ANIPADFLPKWIAY
WDHL-------WTWDSKSFAVGLFTLLIMIYFRNYK----------------------------PRYPGVMIAIVTATTLVWLLKIDI----------PTI
GSRYGTLPT-AIPLPKIPQL--------------SITKILQLMPDALTIAVLSGLETLLSAVVADGM-TGWRHQSNCQLVAQGVANIGT
SLFSGIPVTGSLSRTAASIK-SEATTPIAGIVHSIFICFILLLLAPLTVKIPLTCLAAVLILIAWNMSEIH---HFIHLFTAPKKDIVVLLT
VFILTVMTT-ITAAVQVGMMLAAFLFMKQMSDLSDVISTAKYFDKDSDFLSKA--
-----------------------------------EVPQNTEIYEINGP-FFFGIADRLKNLLNDIEKP---
---------------------------------PKIFILCMTRVPT--IDASAMHALEEFFLECDRQ----GTLLLLAGV-KKTPLADLKRYHLD-ELIG
VD-HIFSNIKSALLFAQALTNLESKTSTRHLV---

---</seq-data>
</sequence>
<sequence seq-type="Protein">
<seq-name>O33206</seq-name>
<seq-info><length>1354</length></seq-info>
<seq-data>--
---MLQR---IARELLSGVAVAIVALPLAIAFGITATG
TSQGALIGLYGAIFAGFFAAVFGGTPGQVTGPTGPITVVATATIAE---
HGLEGAFFAFILAGVFQILFGACRLGSLIRYVPHPVISGFMGGIAILIIMTQLD---QVRSSS
LLVLVTVVLLLASGRFI----------------------------KAIPPSLLVLVLVSSVLPLAAPWLRDLRAGPVSINRTVDYIGEIPQ-AMPSF
DFPQV--------------ANSTMLQVLLSAVAIALLGSLDSLLTSLVMDNI-RGTRHRSNKELIGQGIGNIAAGLFGGLSGAGATVR
SVVNVR-NGGQTALSAATHSVVLFVFVAGLGAVVQYIPLAVLSGILILVAVGMFDWHAM---RKAHVSPRGDVIVMFTTMI
ITVVVD-LTIAVMVGIALSLLVHRLRSRQRKAKVT--
-----------------QDDTGTYRIDGP-LSFLSVDGVFGSLR---

----------DGREDVSLDLQHVTY--LDTSGARALLYFIDHSEKD----GVAVSIKRI-PPRLESQLTALADNEQRDKLRTVLESA------
----

----------</seq-data>
</sequence>
<sequence seq-type="Protein">
<seq-name>Q55814</seq-name>
<seq-info><length>1354</length></seq-info>
<seq-data>---
---MATTLLSPTRFVHGLRFD--NLRGDIFGGITAAIVALPLALAF
GVAS---GAGPIAGLYGAICTGFFAALFGGTPAQISGPTGPMTVVMASTFANLTASNPE--
----------------NGWAMALTVVMFGGIFQVVLGLLGLGKYITFMPYTVISGFMSGIGVIIVILQIAPLLGHQTAP--------GVVPS
LQAIPTYLND---------VNFAALGLGLIALAIVFFSPPRLN-----------------------------RILPAPLIALVLGTIISMIFFGNV-----------
-SLPRIGEIPS-GLPSLHLPYL--------------SWGSLQQMLLYSIMLAVLGSLDSLLTSLVADNI-SRSHHDSDRELVGQGIGNLVS
GLCGGLPGAGATMRTVININ-SGGKTPIAGMIHALILLMIVLKAGQLTEPIPHAVLAGILIKVGIDIVDWS---FLKRAHQLSTK
GAGLMYLVLGLTVFVD-LITAVAVGVFVANLFTVKNLTDLQLESIKSVTRADGEDWLLEE---------------------------------------
--EQQILRAANGRIFMFHLSGP-MSFGAAKSIARRLAIVEN-----------------------------------
---YDVLILDLATVPR--LGVTALLAIETMLKDALAK----QREVFLVGA-KGQVQAR
LQRLPVLAQLSPHHRLESRLLALQMAAVCINVD--

---</seq-data>
</sequence>
<sequence seq-type="Protein">
<seq-name>Q55415</seq-name>
<seq-info><length>1354</length></seq-info>
<seq-data>---
---MQITNKIHFR--NLQGDLFGGVTAAVIALPMALAFGI
AS---GAGATAGLWGAVIVGFFAALFGGTPTLISEPTGPMTVVQTAVIASLVAADPD--
------------NGLAMAFTVVMMAGLFQIAFGLLKLGKYVTMMPYTVISGFMSGIGIILVILQLAPFLG-QASP------KGGVIGTL
QALPNLVSN---------VRPVETLLLALMTVGIIWFMPSRWK-----------------------------KFAPPQLVALVLGTIISITLFGDL-------
-----DIRRIGEIQA-GLPALQLPVF--------------QADQLQRMLIDAAVLGMLGCIDALLTSVVADSL-TRTEHNSNKELVGQGI
GNVMSGLFGGLGGAGATMGTVVNIQ-SGGRTALSGLIRAMVLLVVILGAAKLAATIPLAVLAGIAFKVGVDIIDWG---FLKR
AHHVSIKGALIMYAVIVLTVLVD-LIAAVGIGVFIANILTIDRMSALQSKAVKSISDADDEILLSAN--------------------------------
---EKRWLDEGNGRVLLFQLSGP-MIFGVAKAIAREHNAIQE---------------------------
---CAAIVFDLSDVPH--LGVTASLALENAIEEAAEK----GRAVYIVGA-TGQ
TKRRLEKLQVFRFVPESNCYDDRSEALKDAVLALGPHESEDSPSSSSVQTTY---

---</seq-data>
</sequence>
<sequence seq-type="Protein">
<seq-name>P96878</seq-name>
<seq-info><length>1354</length></seq-info>

<seq-data>--
--MTIPRSQHMSTAVNSCTEAPASRSQWMLA--NLRHDVPASLVVFLV
ALPLSLGIAIAS---GAPIIAGVIAAVVGGIVAGAVGGSPVQVSGPAAGLTVVVAELIDE---
-------------------LGWPMLCLMTIAAGALQIVFGLSRMARAALAIAPVVVHAMLAGIGITIALQQIHVLLGGTS--------HSSA
WRNIVALPDGILHH---------ELHEVIVGGTVIAILLMWSKLPAKV--------------------------RIIPGPLVAIAGATVLALLPVLQ--
--TERIDLQGNFFDAIG-LPK---LAEMSPGGQP-----------WSHEISAIALGVLTIALIASVESLLSAVGVDKLHHGPRTDFNRE
MVGQGSANVVSGLLGGLPITGVIVRSSANVA-AGARTRMSTILHGVWILLFASLFTNLVELIPKAALAGLLIVIGAQLVKLA---
HIKLAWRT--GNFVIYAITIVCVVFLN-LLEGVAIGLVVAIVFLLVRVVRAPVEVKPVG---
--GEQSKRWRVD-IDGTLSFLLLPRLTTVLSKL---------------------------------
--PEG-SEVTLNLNADY---IDDSVSEAISDWRRAHETR----GGVVAIVET-SPAKLHHA
HARPPKRHFASD-PIGLVPWR-------------------------------------SARGKDRGSAS----------VLDRIDEYHRNGAAVLHPHI
AGLT-DSQDPYELFLTCADSRILPNVITASGPGDLYTVRNLGNLVP---TDPDDRSVDAALDFAVNQLGVSSVVVCGHSSCAA
MTALLEDDPANTTTPMMRWLENAHDSLV------VFRNHHPARRSAESA-----------GYPEADQLSIVNVAVQVERLTRHPIL
ATAVAAADLQVIGIFFDISTARVYEVGPNGIICPDEPADRPVDHESAQ</seq-data>
</sequence>
<sequence seq-type="Protein">
<seq-name>Q9X927</seq-name>
<seq-info><length>1354</length></seq-info>
<seq-data>--
--MSACVPARADSARTKHVHPPHSPPPHGPRRFRV---AGADVSAAIAVFLI
ALPLSLGIALAT---GAPLQAGLVAAAVGGIVAGRIGGCPLQVSGPAAGLTVVTADLIQH--------------------------------------
----------------------YGWRTTCAITVFAGIAQLGLGCLRVARSALAVSPAIVHGMLAGIGVTIAVAQLHIVLGGTP--------QSS
VPDNLVALPAQLANL---------HPADLAVSVLTLTLLLLWPRIPGRAGQAL----------------------GKLPAALIAVAGATAVAAL
TGLR----LPKVDLPSWSSHALASLPD----GPVLGLV--------------AAVLTTTLVCSVQSLLGAVAVDKLVAARPSLTG-RVKRSD
LDRELLGQGAANIVSGSLGGLPIAGVAVRSSANVA-AGAVSRNSTMLHGVLVVIAALLMVPALELIPLASLAALVMAVGLK
MVSLN---HIRTVTRH--REVLVYAVTTCGVVFLG-VLEGVALGIAVAVGVALHRLTRTRITHDETE------------------------------
---GVHHVHVRGQ-LTFLAVPRLSRVL-HQV----------------------
---PHG-ADAVVELDGSF---MDHAAYETLQDWQKTHTAQ----GGSVDI
TGR-RPGTRISEPAEEDGCRCR---PWTPWRNHQCERPPTAPPRGTAGRSATDGSPAGNLGTSTGPNTPGTRDATGARGA
TGGSSAPDGPTGHELARGISAFHRNTGPLMRRELARLAREGQQPSQLFLTCADSRVVTSMITSSGPGDLFVVRNVGNFVP
LPGEESGDDSVAAAIEYAVDVLKVRSITVCGHSGCGAMQALLAAEPGGARTPLQRWLRHGLPSLERALPDVDVDVDLNE
QEQKRGAGRARPRLAGRGAADTAERLCLANVVQQLEHLRAHDSVARALGAGDLELHGMYFHVGEAQSYLLSEDAEGSV
FELVRET--NLTA-</seq-data>
</sequence>
<sequence seq-type="Protein">
<seq-name>SULX_YEAST</seq-name>
<seq-info><length>1354</length></seq-info>
<seq-data>---MTSNNSLLGRGRMSY
SSTAPPRFKRSVDQRDTFSDNFDYDKDSSNRGRTYIAASNSTTGVPPPNNSRSGCTNNTNNTNNTSNTSNTNNNDSVDE
NTVFETLPYYLPCFSWLPEYTFN--KLWGDVIAGISVASFQIPLALSYTTSI--AHVPPLCGLYSLAISPFVYGILGSVPQMIVGPE
SAISLVVGQAVESITLHKENV---SLIDISTVITFVSGTILLFSGISRFGFLGNVLSK
ALLRGFISSVGLVMIINSLISELKLDKFLVSLPQHYHTPFEKILFLIDYAP---------AQYHIPTAIFSGCCLIVLFLTRLLKRKLMKYH

K-------------------SAIFFPDILLVVIVTILISMKFNL---------KHRYGISIIGDFSMDNFDELKNPLT-RP-----------RRKLIPDLFSA
SLIVAMLGFFESTTASKSLGTT-YNLTVSSNRELVALGFMNIVISLFGALPAFGGYGRSKINAL-SGAQSVMSGVFMGVITLIT
MNLLLQFVHYIPNCVLSVITTIIGISLLEEVPGDIKFHLRCGGFSELFVFAVTFCTTIFYS-IEAGICIGCVYSIINIIKHSAKSRIQILA
RVA-GTSNFTNLDD---YMMNMKRNSLDVEGTEEIEGCMIVRI
PEP-LTFTNSEDLKQRL-DRIERYG--SSKIHPGRKSLRSKDSIKYVIF
DLGGMTS--IDSSAAQVLEEIITSYKRR----NVFIYLVNV-SINDKVRRRLFKAGVAASVERAQANNNENNTSNTFSDAGETYS
PYFDSIDAALYEIEKMKIKGNNVPNNDSESFMSNTLFNSSLV---

----------------------------</seq-data>
</sequence>
<sequence seq-type="Protein">
<seq-name>O59782</seq-name>
<seq-info><length>1354</length></seq-info>
<seq-data>---
-------------MSSPSENHLLGPKTSFIDNRTSTSRPLHEIPSYQSLARRSSTWKRANIPQQKPSLVRRINYYIPVLHWLPNYSLR-
-NIIWDVLAGCSTACLSVPIALSFAQTF--LGVPPIYILTGTAIGPILYCLFTACPLISIGPEAGMCLLIAENIHQRVLSKADVPQE--
---TAILVTGLIAFIAGIINLAAGLFRLGFLDALVSPVLLRGCILSISMIIMINQGSVFF
GFSGV---KYKGSDFPIDKLMFLIRNMS----------KANIYTTILSCITISLLIGCRNLKSKLSAKYP-------------------RIVSIPDAVIIL
LLGSFLSKKFDW---------HSNYGIAILGEIKT-TILLPKLPLPEKN-----------KLHFITQSLQTGVMCSFLAFIDTVIAVKAISLQ-T
NNLIRSNRELISLGAANIGSSLFCGLPICGGYLRTKCNIM-SGARTQVATIACSVLILLATFFIMPVFSTVPTCMLASMVVSLGV
SLFADAAVEIFKLARIRVWWELGIIFSIATCTMMFG-LETGIIFGLSITVMQIIRHSTRSRIMFRSPTSNGTAEFILEDA----------
---ASTLSHRTNPSSTAVESAPRILVVRIPEP-LFFANVSQLEDRL-NRLEKY
G--HPRMHPGETPYRRIEDIEVVVFDMVGVSS--IDSSALFAFQRI
LKEYVEH----QVEVHLVSL-DPQVLHIFEKHGLL-DLIGGYDHVQDSIKKVDALCDIELGV---------------------------------------
----
--</seq-data>
</sequence>
<sequence seq-type="Protein">
<seq-name>YG35_YEAST</seq-name>
<seq-info><length>1354</length></seq-info>
<seq-data>MGRTIRRRRSNSSLSEAISVSLGINQDSSVNKMHRASVSAMSPPLCRSYMSGFFTGGNSPMINNLSDSKLPIS
NKQHPKVIHGSENLHRQTAQLSNEFCSSSVEENSPTIKDYMDIIGNGDRKDDQSMRTIEENIDEEYSDEYSRLLLSPASSNV
DDDRNRGLQNSSLPELEDGYAGGYQSLRPSHNLRFRPRNLWHMCTSFPSKFAHYLPAAVLG---LLLNILDALSYGMIIFPIT
EPVFSHL---GPTGISMFYISTIISQAVYSGGWSSFPSGIGSEMIEITPFYHTMALAIKEALAGNDDEIIT--------------------------
-------------------TTIFCYVISSMLTGVVFYALGKLRLGKIVGFFPRHILIGCIGGVGYFLIITGIEVTTRV----------AKFEYSWPF
FSGLF---------TDYDTLAKWLLPVLLTVVLIGTQRYFKNS------------------------LVLPSFYILTLVLFHFIVAIIPTLS------LDAL
RQAGWIFPIANSDSKWYDHYRLFNVHKV--------HWSLVLQQIPTMMALTFFGILHVPINVPALAMSLQMDKYDVDRELIA
HGYSNFFSGLLGSVQNYLVYTNSVLFIR-AGADSPFAGFLLIALTICIMIIGPVIISFIPICIVGSLIFLLGYELLVEALV---DTWNKL
NRFEYLTVVIIVFTMGIFD-FVLGIIVGILIACFSFLVDSTKLQTINGEYNGNVARSTVYRDY-------------------------------------
-------------------------------------VQTKFLDGIGEQIYVLKLQNL-LFFGTIISIEEKIERLLQIS------------------------------------
------------------------------------NKDATKRRIKYLILDFKNINADNIDYSAAEGFNRIKRFTETK----RIKLIISSI-KERDRIYNAF
NNVGLLND----VELFADLNSALEWCENEFLFQYKQLRKKAKERLEEGKQNNVVSAVIAATKNKKIDTIGNGLNRGSNGDTA

RNLMSLPTNTPRNYQILSVAQNVFVNDEQAVKNFKKEYKD-DEPVLPILLFALKQYRPDIISEVQKVREKEIKFWAQLCPYFT
RRRLASQSHLLHADNIFFLVETGMLKATYELPQGTLYEIFSNGTCFGKIIAPGNAMPREQKLTIETETDSVLWVIDSSSLNK-L
KEDNLALYVEVALMVMCIKDT--RFKELLGYTLVSA-------------------------------</seq-data>
</sequence>
<sequence seq-type="Protein">
<seq-name>YA7B_SCHPO</seq-name>
<seq-info><length>1354</length></seq-info>
<seq-data>--MPTHSLNQKRSLLYPSIDNGSVPDSSFLSQSFRDTNDIHLLSARLA
SVEVDPETLPSNSPETNDDVSNYDYMSISSYQRAHSLASTSRNDFNYRPSEINETRPLLPEREEHSSQLPPAQNQVLPFQSL
NAPSKKLRGRISLSKIYQFFFDDKGPILFILNIPAVIIG---LLLNILDALSYGLILFPISDPLFKNL---GADGLAIYYVSCVVSQLVYSL
GGSFFKGSVGSEMIEVIPFFHQIAFTILNRVGEDNPKSVIA---TTILAYCLSSILTGLVFF
ILGILRLGRLIEFFPRHILLGCIGGVGSFLVLTAVEVSSRL----------EGSVSFNWASLSALF----------QPMTFAKWSIPLFLSSAL
EFAQQRWPHP-------------------------FLIPSFFVIAPAIFYVLVWAIPGMS------LEYLRETGWVFSSTETNVPWYHFYSLFSL
RDT--------DWSALLATVPEMCALTFFGILHVPINVPALAISLGLDFVDTDKELIAHGVSNTLSGAVGSIQNYMTYTNSLMFIR
-SGGNNRLAGIMLALATVALLVIGPGIIAYIPVWTVGCLIYLLGIELLKESLW---DPIGITTKIEYFTICAIVFTMTVVD-FVVGIVI
GIIMACVFFVIQASSRSALRGIYSGGIVRSTVRRPM---N
QQRFLNEIGRQIQVCKLSGF-LFFGTINGVEKNIAGLIEEL---
---NVSNNPLRFLIIDFSLVNG--ADFSVVQAFLRIRRMLATM----NVQLCVCGL-DETRSSFKTLTTMCFGGDDNCGCQVFED
VNSSLEYCENMLLDDYDVYRTKLL----------------------------------HKAGYSHTLAVPGKHKQNISMAETFSPSPRHDLLRQV
AMSSVKAETKELSKFEKYAKYEQPFPLLMQVFGEITTKREDFWLGLCPFFKKAFLRKGDLLWRKGDTPSKLVILETGMVKAS
YQMDRESLSENITCLCVVGELPFFSKTCYNATVVAELDSAVWILDREGWETMLKSSDKAEVIENEMLYLTLKMTRDKFNVF
TNFALNLYR------------------------------</seq-data>
</sequence>
<sequence seq-type="Protein">
<seq-name>O22123</seq-name>
<seq-info><length>1354</length></seq-info>
<seq-data>--M
SYASLSVKDLTSLVSRSGTGSSSSLKPPGQTRPVKVIPLQHPDTSNEARPPSIPFDDIFSGWTAKIKRMRLVDWIDTLFPCFR
WIRTYRWS-EYFKLDLMAGITVGIMLVPQAMSYAKLA---GLPPIYGLYSSFVPVFVYAIFGSSRQLAIGPVALVSLLVSNALG
GIADTNEE---LHIELAILLALLVGILECIMGLLRLGWLIRFISHSVISGFTSASAI
VIGLSQIKYFLGYSI---------ARSSKIVPIVESIIAGA-------DKFQWPPFVMGSLILVILQVMKHVGKAKKE--------------------L
QFLRAAAPLTGIVLGTTIAKVFH-------------PPSISLVGEIPQ-GLPTFSFPRSF---------------DHAKTLLPTSALITGVPILESVGI
AKALAAK-NRYELDSNSDLFGLGVANILGSLFSAYPATGSFSRSAVNNE-SEAKTGLSGLITGIIIGCSLLFLTPMFKYIPQCALA
AIVISAVSGLVD---YDEAIFLWRVDKRDFSLWTITSTITLFFG-IEIGVLVGVGFSLAFVIHESANPHIAVLGRLPGTTVYRNIKQ
Y--PEAYTYNGIVIVRIDSP-IYFANISYIKDRLREYEVA
V---DKYTNRGLEVDR--INFVILEMSPVTH--IDSSAVEALKELYQ
EYKTR----DIQLAISNP-NKDVHLTIARSGMV-ELVGKE-WFFVRVHDAVQVCLQYVQSSNLEDKHLSFTRRYGGSNNNSSSS
NALLKEPLLSVEK---
---</seq-dat
a>
</sequence>
<column-score>

<colsco-name>coreblock</colsco-name>
<colsco-owner>0</colsco-owner>
<colsco-type>int</colsco-type>
<colsco-data>0 0 0 0 0 0 0 0 0 0 0 0 0 0 0 0 0 0 0 0 0 0 0 0 0 0 0 0 0 0 0 0 0 0 0 0 0 0 0 0 0 0 0 0 0 0 0 0 0 0 0 0
0 0 0 0 0 0 0 0 0 0 0 0 0 0 0 0 0 0 0 0 0 0 0 0 0 0 0 0 0 0 0 0 0 0 0 0 0 0 0 0 0 0 0 0 0 0 0 0 0 0 0 0 0 0 0 0 0 0 0
0 0 0 0 0 0 0 0 0 0 0 0 0 0 0 0 0 0 0 0 0 0 0 0 0 0 0 0 0 0 0 0 0 0 0 0 0 0 0 0 0 0 0 0 0 0 0 0 0 0 0 0 0 0 0 0 0 0 0
0 0 0 0 0 0 0 0 0 0 0 0 0 0 0 0 0 0 0 0 0 0 0 0 0 0 0 0 0 0 0 0 0 0 0 0 0 0 0 0 0 0 0 0 0 0 0 0 0 1 1 1 1 1 1 1 1 1 1
1 1 1 1 1 1 1 1 1 1 1 1 1 1 1 0 0 0 0 0 1 1 1 1 1 1 1 1 1 1 1 1 1 1 1 1 1 1 1 1 1 1 1 1 1 1 1 1 1 1 1 1 1 1 1 1 1 1 1
1 1 1 0 0 0 0 0 0 0 0 0 0 0 0 0 0 0 0 0 0 0 0 0 0 0 0 0 0 0 0 0 0 0 0 0 0 0 0 0 0 0 0 0 0 0 0 0 0 0 0 0 0 0 0 0 0 0 0
0 0 0 0 0 0 0 0 0 0 0 1 1 1 1 1 1 1 1 1 1 1 1 1 1 1 1 1 1 1 1 1 1 1 1 1 1 1 1 1 1 1 1 1 1 1 1 1 1 1 1 1 1 1 1 1 1 1 1
1 1 1 0 0 0 0 0 0 0 0 0 0 0 0 0 0 0 0 0 0 0 0 0 0 0 0 0 0 0 0 0 0 0 0 0 0 0 0 0 0 0 0 0 0 0 0 0 0 0 0 0 0 0 0 0 0 0 0
0 0 0 0 0 0 0 0 0 0 0 0 0 0 0 0 0 0 0 0 0 0 0 0 0 0 0 0 0 0 0 0 0 0 0 0 0 0 0 0 0 0 0 0 0 0 0 0 0 0 0 0 0 0 0 0 0 0 0
0 0 0 0 0 0 0 0 0 0 0 0 0 0 0 0 0 0 0 0 0 0 0 0 0 0 0 0 0 0 0 0 0 0 0 0 0 0 0 0 0 0 0 0 0 0 0 0 0 0 0 0 0 0 0 0 0 0 0
1 1 1 1 1 1 1 1 1 1 1 1 1 1 1 1 1 1 1 1 1 1 0 0 0 0 0 0 0 1 1 1 1 1 1 1 1 1 1 1 1 1 1 1 1 1 1 1 1 1 1 1 1 1 1 1 1 1 1
1 1 1 1 1 1 1 1 0 0 1 1 1 1 1 1 1 1 1 1 1 1 1 1 1 1 1 1 1 1 1 1 1 1 1 1 1 1 1 1 1 1 1 1 1 1 1 1 1 1 1 1 1 1 1 1 1 0 0
0 0 0 0 0 0 0 0 0 0 0 0 0 1 1 1 1 1 1 1 1 1 1 1 1 1 1 1 1 1 0 0 1 1 1 1 1 1 1 1 1 1 1 1 1 1 1 1 1 1 1 1 0 0 0 0 0 0
0 0 0 0 0 0 0 0 0 0 0 0 0 0 0 0 0 0 0 0 0 0 0 0 0 0 0 0 0 0 0 0 0 0 0 0 0 0 0 0 0 0 0 0 0 0 0 0 0 0 0 0 0 0 0 0 0 0 0
0 0 0 0 0 0 0 0 0 0 0 0 0 0 0 0 0 0 0 0 0 0 0 0 0 0 0 0 0 0 0 0 0 0 0 0 0 0 0 0 0 0 0 0 0 0 1 1 1 1 1 1 1 0 0 0 0 1 1
1 1 1 1 1 1 1 1 1 0 0 0 0 0 0 0 0 0 0 0 0 0 0 0 0 0 0 0 0 0 0 0 0 0 0 0 0 0 0 0 0 0 0 0 0 0 0 0 0 0 0 0 0 0 0 0 0 0 0
0 0 0 0 0 0 0 0 0 0 0 0 0 0 0 0 0 0 0 0 0 0 0 0 0 0 0 0 0 0 0 0 0 0 0 0 0 0 0 0 0 0 1 1 1 1 1 1 1 1 1 1 1 0 0 0 0 1 1
1 1 1 1 1 1 1 1 1 1 1 1 1 0 0 0 0 0 0 0 0 0 1 1 1 1 1 0 0 0 0 0 0 0 0 0 0 0 0 0 0 0 0 0 0 0 0 0 0 0 0 0 0 0 0 0 0 0 0
0 0 0 0 0 0 0 0 0 0 0 0 0 0 0 0 0 0 0 0 0 0 0 0 0 0 0 0 0 0 0 0 0 0 0 0 0 0 0 0 0 0 0 0 0 0 0 0 0 0 0 0 0 0 0 0 0 0 0
0 0 0 0 0 0 0 0 0 0 0 0 0 0 0 0 0 0 0 0 0 0 0 0 0 0 0 0 0 0 0 0 0 0 0 0 0 0 0 0 0 0 0 0 0 0 0 0 0 0 0 0 0 0 0 0 0 0 0
0 0 0 0 0 0 0 0 0 0 0 0 0 0 0 0 0 0 0 0 0 0 0 0 0 0 0 0 0 0 0 0 0 0 0 0 0 0 0 0 0 0 0 0 0 0 0 0 0 0 0 0 0 0 0 0 0 0 0
0 0 0 0 0 0 0 0 0 0 0 0 0 0 0 0 0 0 0 0 0 0 0 0 0 0 0 0 0 0 0 0 0 0 0 0 0 0 0 0 0 0 0 0 0 0 0 0 0 0 0 0 0 0 0 0 0 0 0
0 0 0 0 0 0 0 0 0 0 0 0 0 0 0 0 0 0 0 0 0 0 0 0 0 0 0 0 0 0 0 0 0 0 0 0 0 0 0 0 0 0 0 0 0 0 0 0 0 0 0 0 0 0 0 0 0 0 0
0 0 0 0 </colsco-data>
</column-score>
</alignment>
</macsim>
:::::::::::::
ion_core-block.xml
:::::::::::::
<?xml version="1.0"?>
<!DOCTYPE macsim SYSTEM "http://www-bio3d-igbmc.u-strasbg.fr/macsim.dtd">
<macsim>
<alignment>
<aln-name>1</aln-name>
<sequence seq-type="Protein">
<seq-name>P92960</seq-name>
<seq-info><length>2009</length></seq-info>
<seq-data>--

---------------------------------------MSTTTTEARSPLPLLLRRGRSSTAL------------SASTAEARSPLSILQFRRRSSKDVRNI
TSV----------------SSSLLPAFGTFIEDD-----------------------NPSSKPFIVLHFDR-RYRLWELFLVILVGYSAWASLFELAFEK
AAEG---ALLTIDLVVDFFFAVDIILTFFVSYLDN-----TTYLNVTDHKLIAKRY
LKSVAFVMDVASTLPIQFIYKTITGDVGRGQAFG-----------------------FLNLLRLWRLRRVAELFKRLEKDAHFNYF--------
VIRVIKLLCVTIFWIHLAGCILYWIAYHYPRPTDT-------WIGSQVEDFK---ERSV
WLGYTYSMYWSIVTLTTVGYGDLH-----------------------------AVNSREKTFNMFYMLFNIGLTSYIIGIMTNLVVHGALRT
FAMRSAINDILRYTSKNRLPDTMREQMLAHMQLKFKTAE---LRQEEVLQDLPKAIRSSINQHLFRSIIEEAYLFKGFPEGLLV
QLVSQIQAEYFPPKMEIILQNEIPTDFYVIVSGGVDIIASKGVSEQVLAKLGPGSMAGEIGVVFNI---PQPFTVRTRRLSQVIRI
GHHKFKEMVQSDNDVDAKMIIANFMTYLKGLNDELKKEIPFLRDLLDDADAQVQETVQSEETPQSNDEEIVTVSRHENG
QIEERRREGVPKRVIIHGQAPPNQDNKNNGDSNGRLIILPDSIQLLFDLAEKKLGKRGSTIAMADGAHVEQIDALRENDHLY
IF--

--</seq-data>
</sequence>
<sequence seq-type="Protein">
<seq-name>Q9SUU2</seq-name>
<seq-info><length>2009</length></seq-info>
<seq-data>---

---MGIEKRKKMVWFWPEKHEGGVIKEAEDVAAE
HISREGTMSHYSFSKGLLPPLGVGATARSSR---------------------HIKLRCFIVSPFDP-RYRAWDWFLVILVLYTAWASPFEF
GFLQT-PRA--PLSILDNVVNGFFAVDIVLTFFVAFLDK-----ATYLLVDDPKR
IAWRYTST-WLIFDVVSTVPYELFGSLLHNTI---QGYG-----------------------IFSMLRLWRLHRVSKCFARLEKDRKYNYF----
----WIRCTKLLLVSLFVVHCGACFCYSIAAHYPDPSMT-------FMALAEANWK---
--QKSLLIRYVTAMYWSITTFSTTGYGDIH-----------------------------GNNAEERAFILFYMIFNLGLLAYIIGNMTNLVVHVT
SRTRNFRDTIQAASAFAQRNNLPLGLQEQMVAHLSLRYRTDS-EGLQQQEIIDSLPKAIRSSISHYLFYEVVDKTYLFHGISND
LLFQLVSEMKAEYFPPKEDVILRNEAPSDFYIMVTGAVDIIARVNGVDQVVGEAQTGHVFGEVGVLCYR---PQLFTVRTKRL
SQLLRLNRTAFLNLVQANVGDGAIIMNNLLQHLKDSTDPVMKGILAETELMLAQG-KMDLPLSLCFAAARGDDLLLHQLLK
RGSNPNETDKNGRTALHIAASKGSQYCVVLLLEHGADPNIRDSEGSVPLWEAIIGRHEENAKLLSENGATLSFDTVGYFSCL
AVGQNNLNALKDIVKYGGDISLSDVNGTTALHRAVSEGNLEIVQFLLEKGADMDKPDVYGWTARALAEHQGHEDIKALFY
NQRPVERKTILVSGTPEIKPLMKHSSEPVMTHHHSREAMPPLARAVSQ---RRKLSNFKNSLFGIMSAAKTGDEGGASTRTG
ISEGVGGVYPARVTISGE---------ASSSGKVVKLPDS-LEELIEIGEKKLG-FVATKILSREGAEIDDIRIIRDGDFLLLLKVS---------
--</seq-data>
</sequence>
<sequence seq-type="Protein">

<seq-name>Q38998</seq-name>
<seq-info><length>2009</length></seq-info>
<seq-data>--

---MRGGALLCGQVQDEIEQLSRESSHFSL----------
------STGILPSLGARSNR-------------------------RVKLRRFVVSPYDH-KYRIWEAFLVVLVVYTAWVSPFEFGFLRK-PRP------
--PLSITDNIVNAFFAIDIIMTFFVGYLDK-----STYLIVDDRKQIAFKYLRS-WFLLD
LVSTIPSEAAMRISS------QSYG------------------------LFNMLRLWRLRRVGALFARLEKDRNFNYF--------WVRCAKLVCVT
LFAVHCAACFYYLIAARNSNPAKT-------WIGANVANFL---EESLWMRYVTSM
YWSITTLTTVGYGDLH------------------------------PVNTKEMIFDIFYMLFNLGLTAYLIGNMTNLVVHGTSRTRNFRDTIQA
ASNFAHRNHLPPRLQDQMLAHLCLKYRTDS-EGLQQQETLDALPKAIRSSISHFLFYSLMDKVYLFRGVSNDLLFQLVSEMK
AEYFPPKEDVILQNEAPTDFYILVNGTADLVDVDTGTESIVREVKAGDIIGEIGVLCYR---PQLFTVRTKRLCQLLRMNRTTFL
NIIQANVGDGTIIMNNLLQHLKEMNDPVMTNVLLEIENMLARG-KMDLPLNLCFAAIREDDLLLHQLLKRGLDPNESDNN
GRTPLHIAASKGTLNCVLLLLEYHADPNCRDAEGSVPLWEAMVEGHEKVVKVLLEHGSTIDAGDVGHFACTAAEQGNLKL
LKEIVLHGGDVTRPRATGTSALHTAVCEENIEMVKYLLEQGADVNKQDMHGWTPRDLAEQQGHEDIKALFREKLHERRV
HIETSSSVPILKTGIRFLGRFTSEPNIRPASREVSFRIRETRARRKTNNFDNSLFGILANQSVPKNGLATVDEGRTGN-----PVR
VTISCAEK-------DDIAGKLVLLPGSFKELL-ELGSNKFGIVATKVMNKDNNAEIDDVDVIRDGDHLIFATDS-------------------
---</seq-data>
</sequence>
<sequence seq-type="Protein">
<seq-name>Q38898</seq-name>
<seq-info><length>2009</length></seq-info>
<seq-data>--

---MDLKYSASHCNLSSDMKLRRFHQHRGKGREE
EYDASSLSLNNLSKLILPPLGVASYNQN------------------------HIRSSGWIISPMDS-RYRCWEFYMVLLVAYSAWVYPFEVA
FLNSSPKR--NLCIADNIVDLFFAVDIVLTFFVAYIDE-----RTQLLVREPKQIA
VRYLST-WFLMDVASTIPFDAIGYLITGTSTLNITCN------------------------LLGLLRFWRLRRVKHLFTRLEKDIRYSYF--------
WIRCFRLLSVTLFLVHCAGCSYYLIADRYPHQGKT-------WTDA-IPNFT---ETSL
SIRYIAAIYWSITTMTTVGYGDLH------------------------------ASNTIEMVFITVYMLFNLGLTAYLIGNMTNLVVEGTRRTM
EFRNSIEAASNFVNRNRLPPRLKDQILAYMCLRFKAES---LNQQHLIDQLPKSIYKSICQHLFLPSVEKVYLFKGVSREILLLLVS
KMKAEYIPPREDVIMQNEAPDDVYIIVSGEVEIIDSEMERESVLGTLRCGDIFGEVGALCCR---PQSYTFQTKSLSQLLRLKTS
FLIETMQIKQQDNATMLKNFLQHHKKLSNLDIGDLKAQQNGENTDVVPPNIASNLIAVVTTGNAALLDELLKAKLSPDITDS
KGKTPLHVAASRGYEDCVLVLLKHGCNIHIRDVNGNSALWEAIISKHYEIFRILYHFAAISDPHIAGDLLCEAAKQNNVEVMK
ALLKQGLNVDTEDHHGVTALQVAMAEDQMDMVNLLATNGADVV----------CVNTHNEFTPLEKLRVVEEEEEEERG------
---RVSIYRGHPLERRERSCNEAGKLILLPPSLDDLKKIAGE

KFGFDGSETMVTNEDGAEIDSIEVIRDNDKLYFVVNKII---
--------------------------------------</seq-data>
</sequence>
<sequence seq-type="Protein">
<seq-name>O24538</seq-name>
<seq-info><length>2009</length></seq-info>
<seq-data>---

--MENNFGSLNRKHHHHTSSSSFNLRNV----------
-------SKLILPPLGVSRE---------------------------NQVYSRWIISPMDS-RYRCWESFMVVLIAYSAWAYPFEVAFMHSSPNR--
--KLYIVDNIIDLFFAIDIVMTFFLAFIDG-----TTHLLVRDSKKIVVRYLST-WFI
MDVASTIPYEAIGFILTGKHKLGLPYF-------------------------LLGMLRFWRIRRVKQFFTRLEKDIRFSYF--------WVRCARLLS
VTLFSIHCAGCLYYMLADRYPHQGNT-------WIGAVIPNFR--ETSPRTRYISAI
YWSITTMTTVGYGDLH-------------------------------AVNTMEMIFIIFYMLFNLGLTAYLIGNMTNLVVEGTRRTMEFRNSIE
AASNFVCRNRLPPRLREQILAYMCLRFKAER---LNQHQLIEQLPKSICKSICQHLFFPTVEKVYLFKGVSKEILLSLVAKICAEYI
PPKEDVIMQNEAAEDVYIIVSGEVEIIDSVIEKERVLGTLTTGDMFGEVGALCCR---SQSYTYRTKTLTQLLRLKTGALIEAMQ
IKKEDNILILKNFLQHYKQLKDLSIKDLIVENVEEEDPN----MAVNLLTVAGTGNAAFVEELLRAGLDPDIGDSKGKTPLHIAA
SNGHEECVKVLLKHTCNIHIKDMNGNTALWYAIASKHYSIFRILYQLSALSDPYTAGNLLCLAAKRNDLTVMNELLKQGLNI
DSKDGHGMKPIQIAMTENLVDMVQLLVMNGASVLDMHIPEFSASTLNELLQKREIGHLINVNEEMPSEFVLKGENQEDQ
KQVWKRCNGVEFPRVSIYRGHPIVRREKGFIEAGKLIKLPDSLEKLKIIAGEKFEFDARDAMVTNEEGAEIDSIDVIRDNDKLY
IVE--
---</seq-data>
</sequence>
<sequence seq-type="Protein">
<seq-name>Q9SM12</seq-name>
<seq-info><length>2009</length></seq-info>
<seq-data>---

--MKKNNSSIESTGGGVGGSGGTVSGSGSGSF---
-----NLRNLSKVILPPLGGPSGGQSQS----------------------HGGSDKWVVSPLDS-RYRWWDTFMVVLVAYSAWVYPFEVA
FMNASPKG--GLEVADIVVDLFFAVDIVLTFFVAYIDG-----RTQLLVRDRK
KITLRYLST-FFIMDVASTIPFQGLAYLITGEVRENAVYS-------------------------MLGVLRLWRLRRVKQFFTRLEKDIRFSYF---
-----WIRSARLVAVTLFLVHCAGCLYYLIADRYPDRQKT-------WIGAVIPNFR--
QASLRIRYISSIYWSITTMTTVGYGDLH-------------------------------AQNNVEMIFNIFYMLFNLGLTAYLIGNMTNLVVEGT
RRTMEFRNSIRAASSFVGRNHLPPRLKQQILAYMCLKFRAES---LNQQQLMDQLPKSICKSICEHLFVPVVKDVYLFRGVSR
EMLLSLVTKMKPEYIPPKEDVIVQNEAPDDVYVVVSGEVEVILFDGIYEQVQATLGARDIFGEVSALSDR---AQAFTFRTRTL

SQLLRLKQATLKEAMQSRPEDSVVVIKNFLKHQVEMHGMKVEDLLGDNTGEHDD------DAIVLTVAAMGNSGLLEDLLR
AGKAADVGDAKGRTALHIAASKGYEDCVLVLLKHACNVNIRDAQGNTAMWNAIAAGHHKIFNLLYQFGRASNPRAGGD
VMCLAARRGHLGALQELLKLGLDVDSEDHDGATALRVAMAEGHADAARFLILNGASVDK---------ASLDDDGSGSGSGSG
AARLAMSPTELRELLQKRELGHSITIHDSPAVVPNGGSSGHSRPGRLQSTSSDSQRWP-----------------------------------RV
SVYKGHPFLRNR--TSEAGKLINLPGTMEEFKVIVGEKLKVDAEKALIVSDEGAEIDSIDVIRDNDKLFMVTEEDLRRLASMD
WLSCE---</seq-data>
</sequence>
<sequence seq-type="Protein">
<seq-name>Q9XH40</seq-name>
<seq-info><length>2009</length></seq-info>
<seq-data>--

---MEKKDSGSSLKNENEEKKEKDLEEKPHL-EEDY
ESPTFNLQNVSKVILPPLGASTYNDRNSS--------------------PHHPKTWIISPLDT-RYRCWESLMVVLVGYSAWVYPFEIGF
LMSSPTH--KLYIVDNIIDIFFGVDIVLTFFLAYIDS-----STQLLVQDSKQIAL
RYLST-WFVMDVASSIPYEALGYLFTGKHKVGLTFF------------------------LLGMLRLWRLRRVKQYFTRLEKDIRFSYF-------
-LIRCVRLLCVTFFIAHCGGCLYYLLADVYPHLGRT-------WIGTTNPSFK---GTSI
GIRYISAMYWSLTTMTTVGYGDFH------------------------------AVNPMEMAFTICYMFFNLGLTAYLIGNMTNLVVQGTC
RTMEFRNNIEAATNFVSRNIVSARLKEQIIAYMCLRYKAES---LNHHQLMEQLPKSIRKNICQHLFLPAVQKVYLFKGLSREIL
LSLAAKMKAEYIPPKEEVIMQGDAAEDVYIIVSGEVEIIGFAMDKERVLGILQRWDMFGEVGALCCK---PQSFTYRTKTLTQ
LLRLRTSDFTDTMQTKKDDNMQMLKNFLQHLKKLHNLNDKDLVIDWSRED----DPSMAVNLLHVASTGNSALLEELLKAG
LDSSVADSKGKTPLHIAALNGHEECVKVLLKHACNVHARDMNGDTALWDAIFSKHYSIFRILYQLCVQSDPHTAGDLLCKA
AKRNELTVMSELLKQGLNIESKDHHGCTAMQVAWQHNHVDMIQLLFMNGSDVVG---------ALNYDNNNNNQEERGQ
HNSIWGTYNNNNNSNNHRHIAS---RVSIFRGHPILRRQQGCI
EAGKMIRFPNSIKELKTIAGERFGFDAKDAMVTDEGGAEIDSIDLIRDNDKLFIVE--
---</seq-data>
</sequence>
<sequence seq-type="Protein">
<seq-name>O54852</seq-name>
<seq-info><length>2009</length></seq-info>
<seq-data>--

-----MPV---RRGHVAPQNTFLGTIIRKFEGQ-NKKFIIANARV-QNCAIIYCNDGFCEMTGFSRPDVMQKPCTCDFLHGPETK
--RHDIAQIAQALLGSEERKVEVTYYHKNG-----------------STFICNTHIIPVKNQEGVAMMFIINFEYVTDEDNAASPERVNP
ILPVKSVNRKLFGFKFPGLRVLTYRKQSLPQED-----------PDVVVIDSSKHSDDSVAMKHFKSPTKESCSPSEADDTKALIQPS
QCSPLVNISGPLDHSSPKRQWDRLYPDMLQSSSQLTHSRSRESLCSIRRASSVHDIEGFNVHPKNIFRDRHASEDNGRNVK
GPFNHIKSSLLGSTSDSNLNKYSTINKIPQLTLNFSDVKTEKKNTSPPSSDKTIIAPKVKERTHNVTEKVTQVLSLGADVLPEYK
LQ--------------------TPRINKFTILHYSP-FKAVWDWLILLLVIYTAIFTPYSAAFLLNDREE-QKRRECGYSCS--------------------

------------------------PLNVVDLIVDIMFIIDILINFRTTYVNQ------NEEVVSDPAKIAVHYFKG-WFLIDMVAAIPFDLLIFGSG
SDE----TTT------------------------LIGLLKTARLLRLVRVARKLDRYSEYGA----------AVLMLLMCIFALIAHWLACIWYAIGN
VERP---YLTDKI-GWLDSLGTQIGKRYNDSDSS---SGPSIKDKYVTALYFTFSSLTSVGFG
NVS------------------------------PNTNSEKIFSICVMLIGSLMYASIFGNVSAIIQRLYSGTARYHMQMLRVKEFIRFHQIPNPL
RQRLEEYFQHAWTYTN-G-IDMNMVLKGFPECLQADICLHLNQTLLQNCKAFRGASKGCLRALAMKFKTTHAPPGDTLVH
CGDVLTALYFLSRGSIEILK----DDIVVAILGKNDIFGEMVHLYAK----------PGKSNADVRALTYCDLHKIQREDLLEVLDMYP
EFSDHFLTN--LELTFNLRHESAKSQSIN--DSEGDTCKLRRRRLSFESEGDKDFSKENSANDADDSTDTIRRYQSSKKHFEEKK
SRSSSFISSIDDEQKPLFLGTVDSTPRMVKASRHHGEEAAPPSGRIHTDKRSHSCKDITDTHSWEREHARAQPEECSPSGLQ
RAAWGISETESDLTYGEVEQRLDLLQEQLNRLESQMTTDIQAILQLLQKQTTVVPPAYSMVTAGAEYQRPILRLLRTSHPRA
SIKTDRSFSPSSQCPEFLDLEKSKLKSKESLSSGKRLNTASEDNLTSLLKQDSDASSELDPRQRKSYLHPIRHPSLPDSSLSTVGIL
GLHRHVSDPGLPGK---
---------------------------------</seq-data>
</sequence>
<sequence seq-type="Protein">
<seq-name>O54853</seq-name>
<seq-info><length>2009</length></seq-info>
<seq-data>---

-----MPV---RRGHVAPQNTYLDTIIRKFEGQ-SRKFLIANAQM-ENCAIIYCNDGFCELFGYSRVEVMQRPCTCDFLTGPNTP
--SSAVSRLAQALLGAEECKVDILYYRKDA-----------------SSFRCLVDVVPVKNEDGAVIMFILNFEDLAQL--------------------
---L
AKSSSRSLTQRLLSHSFLGSEGSHSRPSGQGPGPGRGKYRTVSQIPQFTLNFVEFNLEKHRSGSTTEIEIIAPHKVVERTQNVT
EKVTQVLSLGADVLPEYKLQ---------------------APRIHRGTILHYSP-FKAVWDWLILLLVIYTAVFTPYSAAFLLSDQDESQR-
GTCGYTCS---PLTVVDLIVDIMFVVDIVINFRTTYVNT------NDEVVSHPRRIAVHYFK
G-WFLIDMVAAIPFDLLIFRTGSDE----TTT------------------------LIGLLKTARLLRLVRVARKLDRYSEYGA----------AVLFLL
MCTFALIAHWLACIWYAIGNVERP---YLEPKI-GWLDSLGAQLGKQY--NGSDPASGP
SVQDKYVTALYFTFSSLTSVGFGNVS------------------------------PNTNSEKVFSICVMLIGSLMYASIFGNVSAIIQRLYSGTA
RYHTQMLRVKEFIRFHQIPNPLRQRLEEYFQHAWSYTN-G-IDMNAVLKGFPECLQADICLHLHRALLQHCPAFRGASKGC
LRALAVKFKTTHAPPGDTLVHLGDVLSTLYFISRGSIEILR----DDVVVAILGKNDIFGEPASLHAR----------PGKSSADVRALT
YCDLHKIHRADLLEVLDMYPAFADTFWNK--LEVTFNLRDADGGLQSTPRQAPGHQDPQGFFLNDSQSGAAPSLSISDTSA
LWPELLQQMPPSPPNPRQDLDCWHRELGFKLEQLQAQMNRLESRVSSDLSRILQLLQHPQGRPSYILGASASSDLASFPET
SVTRSSESTLLVGHVPSAQTLSYGDLDDHIQTPRNFSPRTPHVAMAMDKTLVPSSEQEQPGGLLSPLASPLRPLEVPGLGGS
RFPSLPEHLSSVPKQLEFQRHGSDPGFTRS--

--------------</seq-data>
</sequence>
<sequence seq-type="Protein">
<seq-name>O19119</seq-name>
<seq-info><length>2009</length></seq-info>
<seq-data>---

-----MPV---RRGHVAPQNTFLDTIIRKFEGQ-SRKFIIANARV-ENCAVIYCNDGFCELCGYSRAEVMQRPCTCDFLL----------
--------GAEERKVEIAFYRKDG----------------SCFLCLVDVVPVKNEDGAVIMFILNFEVVMEKDMVGSPARDTNHRGPPTS
WLAPGRAKTFRLKLPALLALTARESSVRPGGAGGAGAPGAVVVDVDLTPAAPSSESLALDEVPAMDNHVAGLGPAEERR
ALVGSCSPPPPVSAPGPHPSLRAHSLNPDASGSSCSLARTRSRESCASVRRASSADDIE--------AMRAGALPPPPRHASTGA
MHPLRSGLLNSTSDSDLVRYRTISKIPQITLNFVDLKGDPFLASP-TSDREIIAPKIKERTHNVTEKVTQVLSLGADVLPEYKLQ-
-------------------APRIHRWTILHYSP-FKAVWDWLILLLVIYTAVFTPYSAAFLLKETEEGPPAPECGYACQ-------------------
------------------------PLAVVDLIVDIMFIVDILINFRTTYVNA------NEEVVSHPGRIAVHYFKG-WFLIDMVAAIPFDLLIFGSG
SEE-------------------------------LIGLLKTARLLRLVRVARKLDRYSEYGA----------AVLLLLMCTFALIAHWLACIWYAIGNME
QP---HMDSRI-GWLHNLGDQMGKPYNSSGL--GGPSIKDKYVTGLYFTFSSLTSVGF
GNVS------------------------------PNTNSEKIFSICVMLIGSLMYASIFGNVSAIIQRLYSGTARYHTQMLRVREFIRFHQIPNPL
RQRLEEYFQHAWSYTN-G-IDMNAVLKGFPECLQADICLHLNRSLLQHCKPFRGATKDCLRALAMKFKTTHAPPGDTLVHA
GDLLTALYFISRGSIEILR----GDVVVAILGKNDIFGEPLNLYAR----------PGKSNGDVRALTYCDLHKIHRDDLLEVLDMYPEF
SDHFWSS--LEITFNLRDTNMIPGSPGSTEWEGGFNRQRKRKLSFRRRTDKDTEQPGEVSALGPGRAGAGPSSRGRPGGP
WGESPSSGPSSPESSEDEGPGRSSSPLRLVPFSSPRPPGEPPGGEPLTEDCEKSSDTCNPLSGAFSGVSNIFSFWGDSRGRQ
YQELPRCPAPAPSLLNIPLSSPSRRPRGDVESRLDALQRQLNRLETRLSADMATVLQLLQRQMTLVPPAYSAVTTPGPGPTS
TSPLLPVSPFPTLTLDSLSQVSQFMACEELPPGAPELPQEGPTRRLSLPGQLGALTSQPLHRHGSDPGS----------------------
----
-------------------</seq-data>
</sequence>
<sequence seq-type="Protein">
<seq-name>O88877</seq-name>
<seq-info><length>2009</length></seq-info>
<seq-data>--

-----MPV---MKGLLAPQNTFLDTIATRFDGT-HSNFILANAQVAKGFPIVYCSDGFCELAGFARTEVMQKSCSCKFLFGVETN
--EQLMLQIEKSLEEKVEFKGEIMFYKKNG----------------APFWCLLDIVPIKNEKGDVVLFLASFKDITDT---------------------
----
---------------------------KVKITSEDKKED----RAKGRSRA-GSHFDSARRRSRAVLYHISGHLQRREKNKLKINNNVFVDKPA
FPEYKVS--------------------DAKKSKFILLHFST-FKAGWDWLILLATFYVAVTVPYNVCFIGNEDL-STTR---------------------
-----------------------------STTVSDIAVEILFIIDIILNFRTTYVSK------SGQVIFEARSICIHYVTT-WFIIDLIAALPFDLLYAFNVT
VVS-----------------------------LVHLLKTVRLLRLLRLLQKLDRYSQHST----------IVLTLLMSMFALLAHWMACIWYVIGKM
EREDNSLLKWEV-GWLHELGKRLESPYYGNNT--LGGPSIRSAYIAALYFTLSSLTSVGF
GNVS------------------------------ANTDAEKIFSICTMLIGALMHALVFGNVTAIIQRMYSRWSLYHTRTKDLKDFIRVHHLPQ
QLKQRMLEYFQTTWSVNN-G-IDSNELLKDFPDELRSDITMHLNKEILQ-LSLFECASRGCLRSLSLHIKTSFCAPGEYLLRQG
DALQAIYFVCSGSMEVLK----DSMVLAILGKGDLIGANLSIKDQ----------VIKTNADVKALTYCDLQCIILKGLFEVLGLYPEYA
HKFVEDIQHDLTYNLREGH-ESDVISRLSNKSTVPQAEPKGNGSIKKRLPSIVEDEEEEEVEEEETTSLSPIYTRGSSVSHSKKT
GSSKSYLGLSLKQLTSGTVPFHSPIRVSSANSPKTKQEADPPNHGTRKEKNLKVQLCSLGTAGTPELSPRIVDGIEDGNSSEET
QTFDFGSEQIRPEPRISPSLGESEIGAAFLFIKAEETKQQINKLNSEVTTLTQEVSQLGKDMRSIMQLLENILSPQQPSQFCSL
HPTSICPSRESFQTRVSWSAHQPCLHLQANGAHLYHGNVTSDIWSVDPSLVGSNPQRTEAHEQSPVDSELHHSPNLAYSP
SHCQVIQEGHLQFLRCISPHSDTTLTPLQSISATLSSSVCSSSETSLHLVLPSRSEEGSITHGPVSSFSLENLPGSWDREGMMS

ASTEPLENFPVEVVTSTADVKDSKAINV--</seq-data>
</sequence>
<sequence seq-type="Protein">
<seq-name>Q9UQ05</seq-name>
<seq-info><length>2009</length></seq-info>
<seq-data>--

-----MPV---MKGLLAPQNTFLDTIATRFDGT-HSNFLLANAQGTRGFPIVYCSDGFCELTGYGRTEVMQKTCSCRFLYGPETS
--EPALQRLHKALEGHQEHRAEICFYRKDG-----------------SAFWCLLDMMPIKNEMGEVVLFLFSFKDITQS-----------------
----
--------------------------------GSPGLGPQGGRGDSNHENSLGRRGATWKFRSARRRSRTVLHRLTGHFGRRGQGGMKAN
NNVFEPKPSVPEYKVA--------------------SVGGSRCLLLHYSV-SKAIWDGLILLATFYVAVTVPYNVCFSGDDDTPITSR------
--HTLVSDIAVEMLFILDIILNFRTTYVSQ------SGQVISAPRSIGLHYLAT-WFFIDLIA
ALPFDLLYIFNITVTS------------------------------LVHLLKTVRLLRLLRLLQKLERYSQCSA----------VVLTLLMSVFALLAHW
MACIWYVIGRREMEANDPLLWDI-GWLHELGKRLEVPYVNGSV--GGPSRRSAYIAA
LYFTLSSLTSVGFGNVC------------------------------ANTDAEKIFSICTMLIGALMHAVVFGNVTAIIQRMYSRRSLYHSRMK
DLKDFIRVHRLPRPLKQRMLEYFQTTWAVNS-G-IDANELLRDFPDELRADIAMHLNREILQ-LPLFGAASRGCLRALSLHIKT
SFCAPGEYLLRRGDALQAHYYVCSGSLEVLR----DNMVLAILGKGDLIGADIPEPGQEPGLGADPNFVLKTSADVKALTYCG
LQQLSSRGLAEVLRLYPEYGAAFRAGLPRDLTFNLRQGSDTSGLSRFSRSPRLSQPRSESLGSSSDKTLPSITEAESGAEPGGG
PRPRRPLLLPNLSPARPRGSLVSLLGEELPPFSALVSSPSLSPSLSPALAGQGHSASPHGPPRCSAAWKPPQLLIPPLGTFGPP
DLSPRIVDGIEDSGSTAEAPSFRFSRRPELPRPRSQAPPTGTRPSPELASEAEEVKEKVCRLNQEISRLNQEVSQLSRELRHIM
GLLQARLGPPGHPAGSAWTPDPPCPQLRPPCLSPCASRPPPSLQDTTLAEVHCPASVGTMETGTALLDLRPSILPPYPSEPD
PLGPSPVPEASPPTPSLLRHSFQSRSDTFH--
---</seq-data>
</sequence>
<sequence seq-type="Protein">
<seq-name>Q9UQ06</seq-name>
<seq-info><length>2009</length></seq-info>
<seq-data>--

-----MPA---MRGLLAPQNTFLDTIATRFDGT-HSNFVLGNAQVAGLFPVVYCSDGFCDLTGFSRAEVMQRGCACSFLYGPD
TS--ELVRQQIRKALDEHKEFKAELILYRKSG-----------------LPFWCLLDVIPIKNEKGEVALFLVSHKDISET--------------------
----
------------------------------KNRGGPDRWKETGGGRRRYGRARSKG--FNANRRRSRAVLYHLSGHLQKQPKGKHKLNKGVF
GEKPNLPEYKVA--------------------AIRKSPFILLHCGA-LRATWDGFILLATLYVAVTVPYSVCVSTARE-PSAARG------------
---------------------------------------PPSVCDLAVEVLFILDIVLNFRTTFVSK------SGQVVFAPKSICLHYVTT-WFLLDVIAALP
FDLLHAFKVNVYF------------------------------GAHLLKTVRLLRLLRLLPRLDRYSQYSA----------VVLTLLMAVFALLAHWVA
CVWFYIGQREIESSESELPEI-GWLQELARRLETPYYLVGRRPAGGNSSGQS-------------DNCSSS---SEANGTGLELLGGPSL
RSAYITSLYFALSSLTSVGFGNVS------------------------------ANTDTEKIFSICTMLIGALMHAVVFGNVTAIIQRMYARRFLY
HSRTRDLRDYIRIHRIPKPLKQRMLEYFQATWAVNN-G-IDTTELLQSLPDELRADIAMHLHKEVLQ-LPLFEAASRGCLRALS

LALRPAFCTPGEYLIHQGDALQALYFVCSGSMEVLK----GGTVLAILGKGDLIGCELPRREQ----------VVKANADVKGLTYCV
LQCLQLAGLHDSLALYPEFAPRFSRGLRGELSYNLGAGGGSAEVDTSSLSGDNTLMSTLEEKETDGEQGPTVSPAPADEPSS
PLLSPGCTSSSSAAKLLSPRRTAPRPRLGGRGRPGRAGALKAEAGPSAPPRALEGLRLPPMPWNVPPDLSPRVVDGIEDGC
GSDQPKFSFRVGQSGPECSSSPSPGPESGLLTVPHGPSEARNTDTLDKLRQAVTELSEQVLQMREGLQSLRQAVQLVLAPH
REGPCPRASGEGPCPASTSGLLQPLCVDTGASSYCLQPPAGSVLSGTWPHPRPG-PPPLMAPWPWGPPASQSSPWPRAT
AFWTSTSDSEPPASGDLCSEPSTPASPPPSEEGARTGPAEPVSQAEATSTGEPPPGSGGLALPWDPHSLEMVLIGCHGSGT
VQWTQEEGTGV---</seq-data>
</sequence>
<sequence seq-type="Protein">
<seq-name>O89047</seq-name>
<seq-info><length>2009</length></seq-info>
<seq-data>--

-----MPA---MRGLLAPQNTFLDTIATRFDGT-HSNFVLGNAQVAGLFPVVYCSDGFCDLTGFSRAEVMQRGCACSFLYGPD
TS--ELVRQQIRKALDEHKEFKAELILYRKSG-----------------LPFWCLLDVIPIKNEKGEVALFLVSHKDISET---------------------
----
------------------------------KNRGGPDNWKERGGGRRRYGRAGSKG--FNANRRRSRAVLYHLSGHLQKQPKGKHKLNKGV
FGEKPNLPEYKVA---------------------AIRKSPFILLHCGA-LRATWDGFILLATLYVAVTVPYSVCVSTARE-PSAARG-----------
--PPSVCDLAVEVLFILDIVLNFRTTFVSK------SGQVVFAPKSICLHYVTT-WFLLDVIAALP
FDLLHAFKVNVYV-------------------------------GAHLLKTVRLLRLLRLLPRLDRYSQYSA----------VVLTLLMAVFALLAHWV
ACVWFYIGQQEIENSESELPEI-GWLQELARRLETPYYLVSRSPDGGNSSGQS-------------ENCSSSGGGSEANGTGLELLGG
PSLRSAYITSLYFALSSLTSVGFGNVS-------------------------------ANTDTEKIFSICTMLIGALMHAVVFGNVTAIIQRMYARR
FLYHSRTRDLRDYIRIHRIPKPLKQRMLEYFQATWAVNN-G-IDTTELLQSLPDELRADIAMHLHKEVLQ-LPLFEAASRGCLR
ALSLALRPAFCTPGEYLIHQGDALQALYFVCSGSMEVLK----GGTVLAILGKGDLIGCELPQREQ----------VVKANADVKGLT
YCVLQCLQLAGLHESLALYPEFAPRFSRGLRGELSYNLGAGGVSAEVDTSSLSGDNTLMSTLEEKETDGEQGHTISPAPADE
PSSPLLSPGCTSSSSAAKLLSPRRTAPRPRLGGRGRPSRAGVLKPEAGPSAHPRTLDGLQLPPMPWNVPPDLSPRVVDGIE
DGCGSDQHKFSFRVGQSGPECSSSPSPGTESGLLTVPLVPSEARNTDTLDKLRQAVTELSEQVLQMREGLQSLRQAVQLIL
VPQGEGQCPRVSGEGPCPATASGLLQPLRVDTGASSYCLQPPAGSVLSGTWPHPRPGHPPPLMAPWPWGPPASQSSP
WPRATALWTSTSDSEPPGSGDLCSEPSTPASPPPPEEGARTGTPAPVSQAEATSTGEPPPGSGGRALPWDPHSLEMVLIG
CHGPGSVQWTQEEGTGV---</se
q-data>
</sequence>
<sequence seq-type="Protein">
<seq-name>CIKE_DROME</seq-name>
<seq-info><length>2009</length></seq-info>
<seq-data>--

-----MP--GGRRGLVAPQNTFLENIIRRSNSQPDSSFLLANAQIVD-FPIVYCNESFCKISGYNRAEVMQKSCRYVCGFMYGEL
TDKETVGRLEYTLENQQQDQFEILLYKKNNLQCGCALSQFGKAQTQETPLWLLLQVAPIRNERDLVVLFLLTFRDITAL------
----

--KQPIDSEDTKGVLG-----LSKFAK---LARSVTRSRQ--FSAHLPTLKDPTKQSNLAHM
MSLSADIMPQYRQE--------------------APKTPPHILLHYCA-FKAIWDWVILCLTFYTAIMVPYNVAFKNKTSEDV-----------
---SLLVVDSIVDVIFFIDIVLNFHTTFVGP------GGEVVSDPKVIRMNYLKS-WFIIDLLSC
LPYDVFNAFDRDEDGIGS--------------------------LFSALKVVRLLRLGRVVRKLDRYLEYGA----------AMLILLLCFYMLVAH
WLACIWYSIGRSDADNGIQY-----SWLWKLANVTQSPYSYIW----------------------------------SNDTGPELVNGPSRKSMY
VTALYFTMTCMTSVGFGNVA-----------------------------AETDNEKVFTICMMIIAALLYATIFGHVTTIIQQMTSATAKYH
DMLNNVREFMKLHEVPKALSERVMDYVVSTWAMTK-G-LDTEKVLNCCPKDMKADICVHLNRKVFDEHPTFRLASDGCL
RALAMHFMMSHSAPGDLLYHTGESIDSLCFIVTGSLEVIQ----DDEVVAILGKGDVFGDQFWKDSA----------VGQSAANV
RALTYCDLHAIKRDKLLEVLDFYSAFANSFARN--LVLTYNLRHRLIFRRVADVKREKELAERRKNEPQLPQNQDHLVRKIFSK
FRRTPQVQAGSKELVGGSGQSDVEKGDGEVERTKVLPKAPKLQASQATLARQDTIDEGGEVDSSPPSRDSRVVIEGAAVS
SATVGPSPPVATTSSAAAGAGVSGGPGSGGTVVAIVTKADRNLALERERQIEMASSRATTSDTYDTGLRETPPTLAQRDLV
ATVLDMKVDVRLELQRMQQRIGRIEDLLGELVKRLAPGASSGGNAPDNSSGQTTPGDEICAGCGAGGGGTPTTQAPPTS
AVTSPVDTVITISSPGASGSGSGTGAGAGSAVAGAGGAGLLDPGATVVSSAGGNGLGPLMLKKRRSKSGKAPAPPEQTLA
STAGTATAAPAGVAGSGMTSSAPASADQQQQHQSAADQSPTTPGAELLHLRLLEEDFTAAQLPSTSSGGAGGGGGGSGS
GATPTTPPPTIAGGSGSGTPTSTTATTTPTGSGTATRGKLDFL</seq-data>
</sequence>
<sequence seq-type="Protein">
<seq-name>O44164</seq-name>
<seq-info><length>2009</length></seq-info>
<seq-data>--

-----MPV--GKRGLVAPQNTFLENVIRRCNNA-DTSFILANAQVVD-YPIVYCNDGFSKLVGYTRAEIMQKPCSLAFMHGEH
GE--VGSLQKMQEALENARTEQAEIGLCKKNK----------------TPIWLLVHLAPIKNHKDAVVLYLCQFKDITPL---------------
----
---------------------------------KQPLDDENNKALF-----------------------------CVTGKANAVEEVTRVVMNLGGDMLPQYR
QE--------------------TPKTSPHIILHYSS-FKTIWDWSILALTFYTAFMVPFNIAFKNSSRENPGGGID---------------------------
---------------------SVALMDSIVDVIFFADILLNFHTTFVGP------GGEVVIEPSVIRQNYFKS-WFLIDLLSCLPYDIFYMFKRD
DERIGS--------------------------LFSALKVVRLLRLGRVARKLDNYLEYGA----------ATLLLLLCAYVIVAHWLACVWFWIGD
SEVRLKMDNLALPDGWLWKLSNDLRQHYNIPLSNKTTL------------------------------------VGGPSRTSAYISSLYYTMSCM
STVGFGNIA-----------------------------SNTDNEKIFGVCMMIISALLYAAIFGHMTTIIQQMTSSTVRYHEMISNVREFIKLQ
EIPKELAERVMDYVVSTWAMTK-G-IDTAKVLGYCPKDMKADICVHLNRKVFNEHSCFRLASDGCLRSLAMFLELNHAAPG
DLLYHTGESVDALWFVVSGSLEVIQ----DDEVVAILGKGDVFGDEFWKANGS---------TGQSAANVRALTYSDLHMIKKDK
LMDVLDFYKAFANSFARN--MTLTYNLTHRMKFRKVADVKREKELDAKRKNEK-LTLPNDHPIRKLLFRMRERHGPRIFPSP
MFADIEKGLKKSTEISRISSLHSMIDETGGGGSSYVKSPRSKPKRPPLMKRQTVDEDALSRTSWGMDKKDREWSSLSNIKTE
MKSKFDIIGERLTIIEQINSRLALLERVLIGNNGGANTPSTMPVGSFSALNESGNRLTLDAAPVARSVSWSEQHQPHWQRF
VEIYSETSTVPPLRELEAGEWEPPIREPTPNPSTSSSRVPHIQIDEDGEARPPTRTRI--

------------------</seq-data>
</sequence>
<sequence seq-type="Protein">
<seq-name>O95259</seq-name>

<seq-info><length>2009</length></seq-info>
<seq-data>---

-----MTMAGGRRGLVAPQNTFLENIVRRSN---DTNFVLGNAQIVD-WPIVYSNDGFCKLSGYHRAEVMQKSSTCSFMYGE
LTD--KDTIEKVRQTFENYEMNSFEILMYKKNR-----------------TPVWFFVKIAPIRNEQDKVVLFLCTFSDITAF----------------
----
-----------------------------------KQPIEDDSCKGWG---------KFARLTRALTSSRGVLQQLAPSVQKGENVHKHSRLAEVLQLG
SDILPQYKQE-------------------APKTPPHIILHYCV-FKTTWDWIILILTFYTAILVPYNVSFKTRQNNV--------------------------
-------------------------------AWLVVDSIVDVIFLVDIVLNFHTTFVGP------AGEVISDPKLIRMNYLKT-WFVIDLLSCLPYDVIN
AFENVDEVSAFMGDPGKIGFADQIPPPLEGRE-SQGISSLFSSLKVVRLLRLGRVARKLDHYIEYGA----------AVLVLLVCVFG
LAAHWMACIWYSIGDYEIFDEDTKTIRNNSWLYQLAMDIGTPYQFNGSGSGKW-------------------------------------EGGP
SKNSVYISSLYFTMTSLTSVGFGNIA-------------------------------PSTDIEKIFAVAIMMIGSLLYATIFGNVTTIFQQMYANTN
RYHEMLNSVRDFLKLYQVPKGLSERVMDYIVSTWSMSR-G-IDTEKVLQICPKDMRADICVHLNRKVFKEHPAFRLASDGC
LRALAMEFQTVHCAPGDLIYHAGESVDSLCFVVSGSLEVIQ----DDEVVAILGKGDVFGDVFWKEAT----------LAQSCANV
RALTYCDLHVIKRDALQKVLEFYTAFSHSFSRN--LILTYNLRKRIVFRKISDVKREEEERMKRKNEAPLILPPDHPVRRLFQRFR
QQKEARLAAERGGRDLDDLDVEKGNVLTEHASANHSLVKASVVTVRESPATPVSFQAASTSGVPDHAKLQAPGSECLGPK
GGGGDCAKRKSWARFKDACGKSEDWNKVSKAESMETLPERTKASGEATLKKTDSCDSGITKSDLRLDNVGEARSPQDRS
PILAEVKHSFYPIPEQTLQATVLEVRHELKEDIKALNAKMTNIEKQLSEILRILTSRRSSQSPQELFEISRPQSPESERDIFGAS---
----
--</seq-data>
</sequence>
<sequence seq-type="Protein">
<seq-name>CNG_ICTPU</seq-name>
<seq-info><length>2009</length></seq-info>
<seq-data>---

-------------------------MTGQAALERSVSSHRLSVRSRLEGEAERAESAISRTDGDDDTCSELQRVTALELPSAEMLEAFTQRR
PLARLVNLVLSLREWAHKSLVETEQRPDSFLERFRGPQAANDQSAAPADAPKKTF--------------------KERWEGFVVSQSD
D-IYYYWLFFIALASLYNWIMLVARACFDQLQDEN---FFLWVGLDYLCDVIYI
LDTCIRLRTGYLE-------QGLLVKDLAKLRDNYIRTLQFKLDFLSILPTELLFFVTG-----------------------------------YVPQLRFNR
LLRFSRMFEFFDRTETRTNYP------NAFRICNLILYILVIIHWNACIYYAISKALGLSSDT-------WVYSGQNK----------------------
------------------------------------TLSFCYVYCFYWSTLTLTTIGEMPP--------------------------------PVKDEEYVFVVFDFLVGVL
IFATIVGNVGSMIANMNATRAEFQTRIDAIKHYMHFRKVNRTLETRVIKWFDYLWTNKK-T-VDEQEVLKNLPDKLRAEIAI
NVHLDTLKKVRIFQDCEAGLLVELVLKLRPQVYSPGDYICRKGDIGKEMYIIKEGQLAVVADD--GVTQFALLTAGGCFGEISI
LNIQGS------KMGNRRTANIRSIGYSDLFCLSKDDLMEAVAEYPDAQKVLEERGREILRKQGLLDESVAAGGLGVIDTE-EKV
ERLDASLDILQTRFARLLGEFTSTQRRLKQRITALERQLCHTGLGLLSDNEAEGEHAGVPTHTHADIHAQPETHTRTSAETNS
EEET--

---</seq-data>
</sequence>
<sequence seq-type="Protein">
<seq-name>CNG1_HUMAN</seq-name>
<seq-info><length>2009</length></seq-info>
<seq-data>--

--MKNNIINTQQSFVTMPNVIVPDIEKEIRRMENGACSSFSEDDDSASTSEESENENPHARGSFSYKSLRKGGPSQREQYLP
GAIALFNVNNSSNKDQEPEEKKKKKKEKKSKSDDKNENKNDPEKKKKKKDKEKKKKEEKSKDKKE---------------------EEKKE
VVVIDPSGN-TYYNWLFCITLPVMYNWTMVIARACFDELQSDY---LEYWLIL
DYVSDIVYLIDMFVRTRTGYLE-------QGLLVKEELKLINKYKSNLQFKLDVLSLIPTDLLYFKLGW---------------------------------
--NYPEIRLNRLLRFSRMFEFFQRTETRTNYP------NIFRISNLVMYIVIIIHWNACVFYSISKAIGFGNDT-------WVYPDINDPE
---FGRLARKYVYSLYWSTLTLTTIGETPP---------------------------------PVRDSEY
VFVVVDFLIGVLIFATIVGNIGSMISNMNAARAEFQARIDAIKQYMHFRNVSKDMEKRVIKWFDYLWTNKK-T-VDEKEVL
KYLPDKLRAEIAINVHLDTLKKVRIFADCEAGLLVELVLKLQPQVYSPGDYICKKGDIGREMYIIKEGKLAVVADD--GVTQFV
VLSDGSYFGEISILNIKGS------KAGNRRTANIKSIGYSDLFCLSKDDLMEALTEYPDAKTMLEEKGKQILMKDGLLDLNIANA
GSDPKDLE-EKVTRMEGSVDLLQTRFARILAEYESMQQKLKQRLTKVEKFLKPLIDTEFSSIEGPGAESGPIDST-----------------
----

---</seq-data>
</sequence>
<sequence seq-type="Protein">
<seq-name>CNG2_MOUSE</seq-name>
<seq-info><length>2009</length></seq-info>
<seq-data>--

----------------------MMTEKSNGVKSSPANNHNHHPPPSIKANGKDDHRAGSRPQSVAADDDTSSELQRLAEMDTPRRGR
GGFRRIVRLVGIIRDWANKNFREEEPRPDSFLERFRGPELQTVTPHQGDGKGDKDGEGKGT----------------------KKKFELFV
LDPAGD-WYYRWLFVIAMPVLYNWCLLVARACFSDLQRNY---FVVWLVLD
YFSDTVYIADLIIRLRTGFLE-------QGLLVKDPKKLRDNYIHTLQFKLDVASIIPTDLIYFAVGI-----------------------------------HS
PEVRFNRLLHFARMFEFFDRTETRTSYP------NIFRISNLVLYILVIIHWNACIYYAISKSIGFGVDT-------WVYPNITDPE-------
--YGYLAREYIYCLYWSTLTLTTIGETPP---------------------------------PVKDEEYLFFIFD
FLIGVLIFATIVGNVGSMISNMNATRAEFQAKIDAVKHYMQFRKVSKDMEAKVIKWFDYLWTNKK-T-VDEREVLKNLPAK
LRAEIAINVHLSTLKKVRIFQDCEAGLLVELVLKLRPQVFSPGDYICRKGDIGKEMYIIKEGKLAVVADD--GVTQYALLSAGSC
FGEISILNIKGS------KMGNRRTGTIRSLGYSDLFCLSKDDLMEAVTEYPDAKKVLEERGREILMKEGLLDENEVAASMEV-D
VQ-EKLEQLETNMETLYTRFARLLAEYTGAQQKLKQRITVLETKMKQNHEDDYLSDGINTPEPAVAE--------------------------

----

--</seq-data>
</sequence>
<sequence seq-type="Protein">
<seq-name>CNG3_BOVIN</seq-name>
<seq-info><length>2009</length></seq-info>
<seq-data>---

--MAKISTQYSHPTRT
HPSVRTMDRDLDCIENGLSRTHLPCEETSSELQEGIAMETRGLAESRQSSFTSQGPTRLSRLIISLRAWSARHLHQEDQRPD
SFLERFRGAELQEVSSRESHVQFNVGSQEPPDRGRSAWPLARNNTNTCNNSEKDDKAKKEEKEKKEEKKENPKKE---------
------------EKKKDSVVMDPSSN-MYYHWLTVIAVPVFYNWCLLVCRACFDELQSEH---
-----------LMLWLVLDYSADILYGMDMLVRARTGFLE-------QGLMVMDASRLWKHYTQTLHFKLDVLSLVPTDLAYFKLG
M---------------------------------NYPELRFNRLLKLARLFEFFDRTETRTNYP------NMFRIGNLVLYILIIIHWNACIYFAISKFIG
FGTDS-------WVYPNVSNPE---YGRLSRKYIYSLYWSTLTLTTIGETPP-----------
---------------------PVKDEEYLFVVIDFLVGVLIFATIVGNVGSMISNMNASRAEFQAKIDSIKQYMQFRKVTKDLETRVIRWF
DYLWANKK-T-VDEKEVLKSLPDKLKAEIAINVHLDTLRKVRIFQDCEAGLLVELVLKLRPAVFSPGDYICKKGDIGREMYIIKE
GKLAVVAED--GITQFVVLGDGSYFGEISILNIKGS------KSGNRRTANIRSIGYSDLFCLSKDDLMEALTEYPEAKKALEEKGR
QILMKDNLIDEELAKAGADPKDIE-EKVEHLETSLDSLQTRFARLLAEYNATQMKVKQRLSQLESQVKMGLPPDGDAPQTE
ASQP--

---</seq-dat
a>
</sequence>
<sequence seq-type="Protein">
<seq-name>CNG_DROME</seq-name>
<seq-info><length>2009</length></seq-info>
<seq-data>---

--MRHFKVKAMVQSLDISAITGQQTDAEPSKRSKPSALRRTLQALRQRLTK
RNRPKPPDWFLEKFSNTTNTDKIRKGCPAMEDAALSSEIRGS--------------------SVLCNRLSVDPTLQ-SHYRWLAIVSLAV
LYNIIFVVGRAVFWEINKSA--PAFWYTLDYLCDFIYLLDTLVHMHEGFLD---
----QGLLVRDAFRLRRHYFHTKGWYLDVLSMLPTDLAYIWWPPETCSSLYLP------------------------CPVIVRLNRLLRINRL
WEWFDRTETATGYP------NAFRICKVVLAILVLIHWNACMYFAISYEIGFSSDS-------WVYNLNGTRN-----------------------
-----------------------------NTLQRQYIYSFYWSTLTLTTIGETPT------------------------------PENDVEYLFVVADFLAGVLIFA
TIVGNIGSMISNMNVARVEFQNRMDGVKQYMAFRRVGHELEARVIRWFAYTWSQSG-A-LDEERVLAALPDKLKAEIAIQ

VHMDTLKQVRIFHDTEPGLLEALVLKLKLQVFSPGDYICRKGDVGKEMYIVKRGKLSVVGDD--GITVLATLGAGSVFGEVSV
LEIAGN------RTGNRRTANVRSLGYSDLFCLAKRDLWETLSDYPEARSTLTQRGCQLLRKDGLLDEQIFADSQRVHDSIEGGI
EKLELSVENLNMRLARLLAEYTASQAKIKQRLAKLEMNGGPGTWRLECEPQSRARSGRLYSLQPKRRPRSRPDATAKSSDA
AKQNTL--

---</seq-data>
</sequence>
<sequence seq-type="Protein">
<seq-name>CNGX_RAT</seq-name>
<seq-info><length>2009</length></seq-info>
<seq-data>--

---MSQDGK
VKTTESTPPAPT---------------------KARKWLPVLDPSGD-YYYWWLNTMVFPIMYNLIIVVCRACFPDLQHSY----------------
--LVAWFVLDYTSDLLYLLDIGVRFHTGFLE-------QGILVVDKGMIASRYVRTWSFLLDLAS
LVPTDAAYVQLGP-----------------------------------HIPTLRLNRFLRVPRLFEAFDRTETRTAYP------NAFRIAKLMLYIFVVIH
WNSCLYFALSRYLGFGRDA-------WVYPDPAQPG--FERLRRQYLYSFYFSTLIL
TTVGDTPL---------------------------------PDREEEYLFMVGDFLLAVMGFATIMGSMSSVIYNMNTADAAFYPDHALVKKYM
KLQHVNKRLERRVIDWYQHLQINKK-M-TNEVAILQHLPERLRAEVAVSVHLSTLSRVQIFQNCEASLLEELVLKLQPQTYSP
GEYVCRKGDIGREMYIIREGQLAVVADD--GVTQYAVLGAGLYFGEISIINIKGN------MSGNRRTANIKSLGYSDLFCLSKED
LREVLSEYPQAQAVMEEKGREILLKMNKLDVNAEAAEIALQEATESRLKGLDQQLDDLQTKFARLLAELESSALKIAYRIERL
EWQTREWPMPEDMGEADDEAEPGEGTSKDGEGKAGQAGPSGIE--

--</seq-data>
</sequence>
<sequence seq-type="Protein">
<seq-name>Q9XFS1</seq-name>
<seq-info><length>2009</length></seq-info>
<seq-data>--

---MMNPQRNKFVRFNGNDDEFSTKTTRPSVSSV
MKTVRRSFEKGSEKIRTFKRPLSVHSNKNKENN----------------------KKKKILRVMNPNDS-YLQSWNKIFLLLSVVALAFDPLF
FYIPYVKPERFCLNLDKKL---QTIACVFRTFIDAFYVVHMLFQFHTGFITPSSSGFGRG
ELNEKHKDIALRYLGS-YFLIDLLSILPIPQVVVLAIVPRMRRPASLVAK------------------ELLKWVIFCQYVPRIARIYPLFKEVTR
TSGLVTET------AWAGAALNLFLYMLASHVFGSFWYLIS--IERKDRC-------WREACAKIQNCTHAYLYCSPTGEDNRLF-LN

GSCPLIDPEEITNSTVFNFGIFADALQSGVVESRDFPKKFFYCFWWGLRNLSALGQNLKT-------------------------------SAFE
GEIIFAIVICISGLVLFALLIGNMQKYLQSTTVRVEEMRVKRRDAEQWMSHRMLPDDLRKRIRKYEQYKWQETK-G-VEEEA
LLSSLPKDLRKDIKRHLCLKLLKKVPWFQAMDDRLLDALCARLKTVLYTEKSYIVREGEPVEDMLFIMRGNLISTTTY--GGRT
GFFNSVDLVAGDFCGD-LLT-------WALDPLSSQ-FPISSRTVQALTEVEGFLLSADDLKFVATQYRRLHSKQLRHMFRFYSV
QWQTWAACFIQAAWKRHCRRKLSKALREEEGKLHNTLQNDDSGGNKLNLGAAIYASRFASHALRNLRANAAARNSRFP
HMLTLLPQKPADPEFPMDET--

---</seq-d
ata>
</sequence>
<sequence seq-type="Protein">
<seq-name>CAB45784</seq-name>
<seq-info><length>2009</length></seq-info>
<seq-data>---

--MDMNDSQKKYSFLLMIFCLGSEIGFQREQT-------------RP
SLNTVLKNVRRGLKKPLSFGSHNKKRDSNS---------------------STTTQKNIINPQGS-FLQNWNKIFLFASVIALAIDPLFFYIPI
VDGERHCLNLHRNL--EIAASVLRTFIDAFYIIHIVFQFRTAYISPSSRVFGRGELVDDP
KAIAIKYLSS-YFIIDLLSILPLPQLVVLAVIPNVNKPVSLITK-----------------DYLITVIFTQYIPRILRIYPLYTEVTRTSGIVTET-----
-AWAGAAWNLSLYMLASHVFGALWYLIS--VEREDRC-------WREACEKIPEVCNFRFLYCDGNSSVRNDFLTTSCPFINPD
DITNSTVFNFGIFTDALKSGIVESDDFWKKFFYCFWWGLRNLSALGQNLNT-------------------------------SKFVGEIIFAVSICI
SGLVLFALLIGNMQKYLESTTVREEEMRVRKRDAEQWMSHRMLPDDLRKRIRRYEQYKWQETR-G-VEEENLLRNLPKDL
RRDIKRHFCLDLLKKVPLFEIMDEQLLDAVCDKLKPVLYTENSYAIREGDPVEEMLFVMRGKLMSATTN--GGRTGFFNAVY
LKPSDFCGEDLLT-------WALDPQSSSHFPISTRTVQALTEVEAFALAADDLKLVASQFRRLHSKQLQHTFRFYSVQWRTWG
ASFIQAAWRRHCRRKLARSLTEEEDRFRNAITKRERNAASSSSLVATLYASRFASNALRNLRTNNLPLLPPKPSEPDFSLRNP-
----

---</seq-data>
</sequence>
<sequence seq-type="Protein">
<seq-name>O65717</seq-name>
<seq-info><length>2009</length></seq-info>
<seq-data>---

---MNFRQEKFVRFQDWKSDKTSSDVEYSGKNEIQTGIFQRTI
SSISDKFYRSFESSSARIKLFKRSYKSYSFKEAVSK---------------------GIGSTHKILDPQGP-FLQRWNKIFVLACIIAVSLDPLFF

YVPIIDDAKKCLGIDKKM---EITASVLRSFTDVFYVLHIIFQFRTGFIAPSSRVFGRGVL
VEDKREIAKRYLSS-HFIIDILAVLPLPQMVILIIIPHMRGSSSLNTK-----------------NMLKFIVFFQYIPRFIRIYPLYKEVTRTSGI
LTET------AWAGAAFNLFLYMLASHVFGAFWYLFS--IERETVC-------WKQACERNNPPCISKLLYCDPETAGGNAFLNESC-
---PIQTPNTTLFDFGIFLDALQSGVVESQDFPQKFFYCFWWGLQNLSSLGQNLKT------------------------------STYIWEICF
AVFISIAGLVLFSFLIGNMQTYLQSTTTRLEEMRVKRRDAEQWMSHRLLPENLRKRIRRYEQYKWQETR-G-VDEENLLSNL
PKDLRRDIKRHLCLALLMRVPMFEKMDEQLLDALCDRLQPVLYTEESYIVREGDPVDEMLFIMRGKLLTITTN--GGRTGFL
NSEYLGAGDFCGEELLT-------WALDPHSSSNLPISTRTVRALMEVEAFALKADDLKFVASQFRRLHSKQLRHTFRYYSQQW
KTWAACFIQAAWRRYIKKKLEESLKEEENRLQDALAKEACGSSPSLGATIYASRFAANILRTIRRSGSVRKPRMPERMPPML
LQKPAEPDFNSDD--

---</seq-data>
</sequence>
<sequence seq-type="Protein">
<seq-name>O65816</seq-name>
<seq-info><length>2009</length></seq-info>
<seq-data>--

--MEREDRYVRFQDCKSEQSVISENAVSPRRH-------------CV
FRSLKERTGGLFAFLGNFLRSETLKRSMLE--------------------DRKSMQNVFHPQGP-FLQRWNKIFVLSCIFAVSVDPLFLYI
PVINDKNLCWYLDRKM---KITASVLRSFTDIFYILHIIFQFRTGFITSSSTNFGRGVLVE
DRYAIAKRYLST-YFLIDVCAVLPLPQVVIWIVLPTLQVSQFMKAK-----------------NILMLIVICQYVPRVIRIRPLYLQITRSAG
IITET------AWAGAAFNLIIYMLASHVLGAVWYLLS--IQRKDAC-------WKQNCSLTRDCNPAYLYCGNGGTNAGNAFLQN
VC--VPNITKDNLPDPLFGIFVPAI-NNVSQSTDFFAKLFYCVWWGLQNLSSLGQNLKT------------------------------STYAW
ENLFAVFVSISGLVLFSLLIGNMQTYLQSATLRIEETRVKSRDTDQWMSYRLLPDNLKERIRRYEQYRWQETS-G-VDEEHLL
MNLPKDLRRAIKRHLCLSLLKRVPMFEKMDDQLLNALCDCLKPVLYTEGGCIVREGDPVNEMFFITRGNLMSMTTN--GG
KTGFFNSDVLKSGDFCGEELLT-------WALDPNSATSLPSSTRTVKSMSEVEAFALMAEDLKFVAMQFRRLHSKQLRHTFRF
YSQQWRTWAACFIQAAWHRRCRKKMEDALREKEERLQLAIVNDGSTSLSFGAAIYASRFARNMMRTLRRNATRKARLQ
ERVPARLLQKPAEPNFSAEEQ--

---</seq
-data>
</sequence>
<sequence seq-type="Protein">
<seq-name>CIK1_DROME</seq-name>
<seq-info><length>2009</length></seq-info>
<seq-data>--

--MAA

VAGLYGLGEDRQHRKKQQQQQQHQKEQLEQKEEQKKIAERKLQLREQQLQRNSLDGYGSLPKLSSQDEEGGAGHGFGG
GPQHFEPIPHDHDFCERVVINVSGLRFETQLRTLNQFPDTLLG---DPARRLRYF
DPLRNEYFFDRSRPSFDAILYYYQSGGRLRRPVNVPLDVFSEEIKFYELGDQAINKFREDEGFIK----------------------EEERPLP
DNEKQ-------------------------------------RKVWLLFEYPESSQAARVVAIISVFVILLSIVIFCLETLPEFKHYKVFNTTTNGTK--IE
ED--------------------------------------EVPDITDPFFLIETLCIIWFTFELTVRFLACP--------------------NKLNFCRDVMNVIDIIAIIPY
FITLATVVAEEEDTLNLPKAPVSPQDKS----------SNQAMSLAILRVIRLVRVFRIFKLSRHSKGLQILGRTLKASMRELGLLIFFL
FIGVVLFSSAVYFAEA--GSENSFFKSIPDAFWWAVVTM
TTVGYGDMT---------------------------------PVGFWGKIVGSLCVVAGVLTIALPVPVIVSNFNYFYHREADREE-MQSQN-FNH
VTSCSYLPGALGQHLKKSS-LSESSSDIMDLDDGIDATTPGLTDHTGRHMVPFLRTQQSFEKQQL-------------------------------
-----------QLQLQLQQQSQSPHGQQMTQQQQLGQNGLRSTNSLQLRHNNAMAVSIETDV-------------------------------------
----

---</seq-data>
</sequence>
<sequence seq-type="Protein">
<seq-name>CIK2_DROME</seq-name>
<seq-info><length>2009</length></seq-info>
<seq-data>--

-----MTMWQSGGMGGHGSQNNPWMKLMGIVHKERRHTENVQSQSGSNERNLNQSLPKLSSQDEEGGAGHGFGGGP
QHFEPIPHDHDFCERVVINVSGLRFETQLRTLNQFPDTLLG--DPARRLRYFDPL
RNEYFFDRSRPSFDAILYYYQSGGRLRRPVNVPLDVFSEEIKFYELGDQAINKFREDEGFIK----------------------EEERPLPDNE
KQ--------------------------------------RKVWLLFEYPESSQAARVVAIISVFVILLSIVIFCLETLPEFKHYKVFNTTTNGTK--IEED---
----------------------------------EVPDITDPFFLIETLCIIWFTFELTVRFLACP--------------------NKLNFCRDVMNVIDIIAIIPYFITL
ATVVAEEEDTLNLPKAPVSPQDKS----------SNQAMSLAILRVIRLVRVFRIFKLSRHSKGLQILGRTLKASMRELGLLIFFLFIG
VVLFSSAVYFAEA--GSENSFFKSIPDAFWWAVVTMTT
VGYGDMT---------------------------------PVGVWGKIVGSLCAIAGVLTIALPVPVIVSNFNYFYHRETDQEE-MQSQN-FNHVT
SCPYLPGTLGQHMKKSS-LSESSSDMMDLDDGVESTPGLTETHPGRSAVAPFLGAQQQQQQ-PVASSLSMSIDKQLQHPL
QQLTQTQLYQQQQQQQQQQQNGFKQQQQQTQQQLQQQQSHTINASAAAATSGSGSSGLTMRHNNALAVSIETDV--
----

--</seq-data>
</sequence>
<sequence seq-type="Protein">
<seq-name>CIK4_DROME</seq-name>
<seq-info><length>2009</length></seq-info>
<seq-data>--

---MAA
VAGLYGLGEDRQHRKKQQQQQQHQKEQLEQKEEQKKIAERKLQLREQQLQRNSLDGYGSLPKLSSQDEEGGAGHGFGG
GPQHFEPIPHDHDFCERVVINVSGLRFETQLRTLNQFPDTLLG---DPARRLRYF
DPLRNEYFFDRSRPSFDAILYYYQSGGRLRRPVNVPLDVFSEEIKFYELGDQAINKFREDEGFIK---------------------EEERPLP
DNEKQ-------------------------------------RKVWLLFEYPESSQAARVVAIISVFVILLSIVIFCLETLPEFKHYKVFNTTTNGTK--IE
ED------------------------------------EVPDITDPFFLIETLCIIWFTFELTVRFLACP-------------------NKLNFCRDVMNVIDIIAIIPY
FITLATVVAEEEDTLNLPKAPVSPQDKS----------SNQAMSLAILRVIRLVRVFRIFKLSRHSKGLQILGRTLKASMRELGLLIFFL
FIGVVLFSSAVYFAEA--GSENSFFKSIPDAFWWAVVTM
TTVGYGDMT--------------------------------PVGVWGKIVGSLCAIAGVLTIALPVPVIVSNFNYFYHRETDQEE-MQSQN-FNH
VTSCPYLPGTLGQHMKKSS-LSESSSDMMDLDDGVESTPGLTETHPGRSAVAPFLGAQQQQQQQPVASSLSMSIDKQLQ
HPLQHVTQTQLYQQQQQQQQQQQNGFKQQQQQTQQQLQQQQSHTINASAAAATSGSGSSGLTMRHNNALAVSIET
DV--

--</seq-data>
</sequence>
<sequence seq-type="Protein">
<seq-name>Q16968</seq-name>
<seq-info><length>2009</length></seq-info>
<seq-data>--

-------------------------MEVAMAGIEGNGGPAGYRDSYHSSQRPLLRSSNLPNSRSFPKLSEEDNANENGMGVPGSDYDCSC
ERVVINVSGLRFETQLKTLNQFPDTLLG---NPQKRNRYYDPLRNEYFFDRNRPSF
DAILYFYQSGGRLRRPVNVPLDVFSEEIKFYELGENAFERYREDEGFIK---------------------EEEKPLPQNEFQ------------------
------------------RRVWLLFEYPESSAAARLCAIFSVVIILLSIVIFCLETLPQFKHYRVVNSTANDSKESIEED-------------------------
-----------DIPKFNEPFFIIETCCIIWFTFELLVRFASCP-------------------EKLGFFKNIMNCIDIVAIIPYFITLGTVVADQSKS-----
--------------------NNQAMSLAILRVIRLVRVFRIFKLSRHSKGLQILGQTLKASMRELGLLIFFLFIGVILFSSAVYFAEA------------
---DADQTHFKSIPDAFWWAVVTMTTVGYGDMR-------------------
------------PIGVWGKLVGSLCAIAGVLTIALPVPVIVSNFNYFYHREGESTD--KGQ--YKHVQSCPNYPEKKDSLD------SECGS
DIMEMEEGNHSTPLTEKVKENHAIKANNPGSDYGLETDV--

--</seq-data>
</sequence>
<sequence seq-type="Protein">
<seq-name>CIK6_HUMAN</seq-name>
<seq-info><length>2009</length></seq-info>
<seq-data>--

---MRSEKSLTLAAPGEVRGPEGEQQDAGDFPEAGGGGGCCSSERLVINISGLRFE
TQLRTLSLFPDTLLG--DPGRRVRFFDPLRNEYFFDRNRPSFDAILYYYQSGGRLR
RPVNVPLDIFLEEIRFYQLGDEALAAFREDEGCLP-------------------EGGEDEKPLPSQPFQ--------------------------------------R
QVWLLFEYPESSGPARGIAIVSVLVILISIVIFCLETLPQFRVDGRGGNNGGVSRVSPVSRGSQEEEEDEDDSYTFHHGITPGE
MGTGGSSSLSTLGGSFFTDPFFLVETLCIVWFTFELLVRFSACP--------------------SKPAFFRNIMNIIDLVAIFPYFITLGTELV
QQQEQQPASGGGGQN---------------GQQAMSLAILRVIRLVRVFRIFKLSRHSKGLQILGKTLQASMRELGLLIFFLFIGVILFS
SAVYFAEA---DDDDSLFPSIPDAFWWAVVTMTTVGYG
DMY---------------------------------PMTVGGKIVGSLCAIAGVLTIALPVPVIVSNFNYFYHRETEQEE--QGQ--YTHVTCGQP---
APDLRAT---DNGLGKPDFPEANRE
RRPSYLPTPHRAYAEKRMLTEV--

--</seq
-data>
</sequence>
<sequence seq-type="Protein">
<seq-name>CIK2_HUMAN</seq-name>
<seq-info><length>2009</length></seq-info>
<seq-data>--

---MTVATGDPADEAAALPGHPQDTYDPEADHECCERVVINISGLRFETQL
KTLAQFPETLLG---DPKKRMRYFDPLRNEYFFDRNRPSFDAILYYYQSGGRLRRP
VNVPLDIFSEEIRFYELGEEAMEMFREDEGYIK----------------------EEERPLPENEFQ-------------------------------------RQVW
LLFEYPESSGPARIIAIVSVMVILISIVSFCLETLPIFRDENEDMHGSGVTFHTYSNSTIGYQQ-------------------------------STSFT
DPFFIVETLCIIWFSFEFLVRFFACP--------------------SKAGFFTNIMNIIDIVAIIPYFITLGTELAEKPEDAQQ---------------------
-GQQAMSLAILRVIRLVRVFRIFKLSRHSKGLQILGQTLKASMRELGLLIFFLFIGVILFSSAVYFAEA-------------------------------
---DERESQFFPSIPDAFWWAVVSMTTVGYGDMV--------------------------------PTTIG
GKIVGSLCAIAGVLTIALPVPVIVSNFNYFYHRETEGEE--QAQ--YLQVTSCPKIPSSPDLKKSRSA-STISKSDYMEIQEGVNN-
---SNEDFREENLKTANCTLANTNYVNITKMLTDV

--</seq-data>
</sequence>
<sequence seq-type="Protein">
<seq-name>CIK2_XENLA</seq-name>
<seq-info><length>2009</length></seq-info>

<seq-data>--

--MTVATGDLTDGSVGFAGHPQDSYDPEPDHECCERVVINISGLRFETQL
KTLSQFPETLLG--DPKKRMRYFDPLRNEYFFDRNRPSFDAILYFYQSGGRLRRP
VNVPLDIFSEEIRFYELGEEAMEIFREDEGFIK-----------------------EEERPLPDNEFQ---------------------------------------KQVWL
LFEYPESSGPARIIAIISVTVILISIVSFCLETLPVFRDENEDMHGSGGNYYSYPNSTVRFQK---------------------------------SNTFT
DPFFIVETLCIIWFSFEFLVRFLACP---------------------SKAVFFTNLMNIIDIVAIIPYFITLGTELAEKTEDGQQ----------------------
-GQQAMSLAILRVIRLVRVFRIFKLSRHSKGLQILGQTLNASMRELGLLIFFLFIGVILFSSAVFFAEA--------------------------------
---DERDSQFPSIPDAFWWAVVSMTTVGYGDMV----------------------------------PTTI
GGKIVGSLCAIAGVLTIALPVPVIVSNFNYFYHRETEGEE--QAQ--YLQVTSCPKIPSSPDLQKSRSA-STLSKSDYMEIQEGVN
H--SNEDFREKNLKTANCTLGNTNYVNITKMLT
DV--

--</seq-data>
</sequence>
<sequence seq-type="Protein">
<seq-name>CIK4_HUMAN</seq-name>
<seq-info><length>2009</length></seq-info>
<seq-data>--

---MEVAMVSAESSGCNSHMPYGYAAQARARERERLAHSRRAAARAVAA
ATAAVEGSGGSGGGSHHHHQSRGACTSHDPQSSRGSREEEATRSEKKKAHYRQSSFPHCSDLMPSGSEEKILRELSEEEED
EEEEEEEEEEGRFYYSEDDHGDECSYTDLLPQDEGGGGYSSVRYSDCCERVVINVSGLRFETQMKTLAQFPETLLG------------
--DPEKRTQYFDPLRNEYFFDRNRPSFDAILYYYQSGGRLKRPVNVPFDIFTEEVKFYQLGE
EALLKFREDEGFVR----------------------EEEDRALPENEFK--KQIWLLFEYPESSDPARGIAIVSV
LVILISIVIFCLETLPEFRDDRDLVMALSAGGHGGLLNDTSAPHLEN---------------------------SGHTIFNDPFFIVETVCIVWFSF
EFVVRCFACP--------------------SQALFFKNIMNIIDIVSILPYFITLGTDLAQQQGGGN---GQ------------------QQQAMSFAILR
IIRLVRVFRIFKLSRHSKGLQILGHTLRASMRELGLLIFFLFIGVILFSSAVYFAEA---
---------------------------DEPTTHFQSIPDAFWWAVVTMTTVGYGDMK----------------------------------PITVGGKIVGVLCAIAG
VLTIALPVPVIVSNFNYFYHRETENEE--QTQL-TQNAVSCPYLPSNLLKKFRSSTSSSLGDKSEYLEMEEGVKESLCAKE---------
---EKC--QAK-GDDSETDKNNCSNAKAVETDV-----------------

--</seq-data>
</sequence>
<sequence seq-type="Protein">
<seq-name>CIK5_HUMAN</seq-name>

<seq-info><length>2009</length></seq-info>
<seq-data>---

---MEIALVPLENGGAMTV
RGGDEARAGCGQATGGELQCPPTAGLSDGPKEPAPKGRGAQRDADSGVRPLPPLPDPGVRPLPPLPEELPRPRRPPPEDE
EEEGDPGLGTVEDQALGTASLHHQRVHINISGLRFETQLGTLAQFPNTLLG--D
PAKRLPYFDPLRNEYFFDRNRPSFDGILYYYQSGGRLRRPVNVSLDVFADEIRFYQLGDEAMERFREDEGFIK---------------
----EEEKPLPRNEFQ-------------------------------RQVWLIFEYPESSGSARAIAIVSVLVILISIITFCLETLPEFRDERELLRHP
PAPHQPPAPAPGANGSGVMAPPSGPTVAPLL-------------PRTLADPFFIVETTCVIWFTFELLVRFFACP----------------
SKAGFSRNIMNIIDVVAIFPYFITLGTELAEQQPGGGGGGQN---------------GQQAMSLAILRVIRLVRVFRIFKLSRHSKGL
QILGKTLQASMRELGLLIFFLFIGVILFSSAVYFAEA---DN
QGTHFSSIPDAFWWAVVTMTTVGYGDMR---------------------------PITVGGKIVGSLCAIAGVLTIALPVPVIVSNFNYF
YHRETDHEE-PAVLK-EEQGTQSQGPGLDRGVQRKVSGSRGSFCKAGGTLENADSARRGSCP----------------------------
---LEKCNVKAKSNVDLRRSLYALCLDTSRETDL-----------------------------------
----

--</seq-data>
</sequence>
<sequence seq-type="Protein">
<seq-name>CIK3_HUMAN</seq-name>
<seq-info><length>2009</length></seq-info>
<seq-data>---

---------------------------------MTVVPGDHLLEPEVADGGGAPPQGGCGGGGCDRYEPLPPSLPAAGEQDCCGERVVIN
ISGLRFETQLKTLCQFPETLLG--DPKRRMRYFDPLRNEYFFDRNRPSFDAILYYY
QSGGRIRRPVNVPIDIFSEEIRFYQLGEEAMEKFREDEGFLR------------------EEERPLPRRDFQ--------------------------
------RQVWLLFEYPESSGPARGIAIVSVLVILISIVIFCLETLPEFRDEKDYPASTSQDSFEAAGNSTSGSRAG------------------
-------ASSFSDPFFVVETLCIIWFSFELLVRFFACP----------------SKATFSRNIMNLIDIVAIIPYFITLGTELAERQGN--------
-------------GQQAMSLAILRVIRLVRVFRIFKLSRHSKGLQILGQTLKASMRELGLLIFFLFIGVILFSSAVYFAEA--------------
---DDPTSGFSSIPDAFWWAVVTMTTVGYGDMH---------------------
-------PVTIGGKIVGSLCAIAGVLTIALPVPVIVSNFNYFYHRETEGEE--QSQ--YMHVGSCQHLSSSAEELRKARSNSTLSKSEY
MVIEEGGM--NHSAFPQTPFKTGNSTATCTTNNNP
NSCVNIKKIFTDV--

--</seq-data>
</sequence>
<sequence seq-type="Protein">

<seq-name>CIK1_HUMAN</seq-name>
<seq-info><length>2009</length></seq-info>
<seq-data>---

---MTVMSGENVDEASAAPGHPQDGSYPRQADHDDHECCERVVINISGLRFE
TQLKTLAQFPNTLLG---NPKKRMRYFDPLRNEYFFDRNRPSFDAILYYYQSGGR
LRRPVNVPLDMFSEEIKFYELGEEAMEKFREDEGFIK-----------------------EEERPLPEKEYQ---------------------------------------R
QVWLLFEYPESSGPARVIAIVSVMVILISIVIFCLETLPELKDDKDFTGTVHRIDNTTVIYN--------------------------------------SNI
FTDPFFIVETLCIIWFSFELVVRFFACP--------------------SKTDFFKNIMNFIDIVAIIPYFITLGTEIAEQEGNQK-------------------
----GEQATSLAILRVIRLVRVFRIFKLSRHSKGLQILGQTLKASMRELGLLIFFLFIGVILFSSAVYFAEA-------------------------------
---EEAESHFSSIPDAFWWAVVSMTTVGYGDMY---------------------------------PVTI
GGKIVGSLCAIAGVLTIALPVPVIVSNFNYFYHRETEGEE--QAQ--LLHVSS-PNLASDSDLSR-RSS-STMSKYEYMEIEEDMN
NS--IAHYRQVNIRTANCTTANQNCVNKSKLLT
DV--

---</seq-data>
</sequence>
<sequence seq-type="Protein">
<seq-name>CIKB_HUMAN</seq-name>
<seq-info><length>2009</length></seq-info>
<seq-data>---

---MAEKAPPGLNRKTSRSTLSLPPEPVDIIRSKTCSRRVKINVGGLNHEVLW
RTLDRLPRTRLGKLRDCNTHE---SLLEVCDDYNLNENEYFFDRHPGAFTSILNFYRTG-KL
HMMEEMCALSFGQELDYWG-IDEIYLESCCQARYHQKKEQMNEELRREAETMRDGEGEEFDNTCCPDKR------------------
--------------------KKLWDLLEKPNSSVAAKILAIVSILFIVLSTIALSLNTLPELQETDEF--
--GQLNDNRQLAHVEAVCIAWFTMEYLLRFLSSP--------------------NKWKFFKGPLNVIDLLAILPYYVTIFLTESNKSVL-------
-------------------QFQNVRRVVQIFRIMRILRILKLARHSTGLQSLGFTLRRSYNELGLLILFLAMGIMIFSSLVFFAEK-------------
--DEDATKFTSIPASFWWATITMTTVGYGDIY--------------------------
------PKTLLGKIVGGLCCIAGVLVIALPIPIIVNNFSEFYKEQKRQEK-AIKRREALERAKRNGSIVS---MNLKDAFARSMELIDV
AVEKAGESANTKDSADDNHLSPSRWKWARKALSETSSNKSFENKYQEVSQKDSHEQLNNTFSSSPQHLSAQKLEMLYNEI
TKTQPHSHPNPDCQEKPERPSAYEEEIEMEEVVCPQEQLAVAQTEVIVDMKSTSSIDSFTSCATDFTETERSPLPPPSASHL
QMKFPTDLPGTEEHQRARGPPFLTLSREKGPAARDGTLEYAPVDITVNLDASGSQCGLHSPLQSDNATDSPKSSLKGSNPL
KSRSLKVNFKENRGSAPQTPPSTARPLPVTTADFSLTTPQHISTILLEETPSQGDRPCWALRFQRLVRDLPKGCPPGFPSRNC
SLSLQERGGASLK---

---</seq-data>

</sequence>
<sequence seq-type="Protein">
<seq-name>CIKB_CANFA</seq-name>
<seq-info><length>2009</length></seq-info>
<seq-data>--

---MAEKAPPGLNRKTSRSTLSLPPEPVDIIRSKTCSRRVKINVGGLNHEVLW
RTPDRLPRTRLGKLRDCNTHE--SLLEVCDDYNLGHNEYFFDRHPGAFTSILNFYRTG-KL
HMMEEMCALSVGQELDYWG-IDEIYLESCCQARYHQKKEQMNEELRREAETMRDGEGEEFDNTCCPEKR----------------
------------------KKSRDLLEKPNSSVAAKILAIVSNLFIVLSTIALSLNTLPELQEMDEF---
----GQPNDNPQLAHVEAVCNAWFTMEYLLRFLSSP------------------NKWKFFKGPLNVIDLLAILPYYVTIFLTESNKSVL----
--------------------QFQNVRRVVQIFRIMRILRILKLARHSTGLQSLGFTLRRSYNELGLLILFLAMGIMIFSSLVFFAEK----------
--DADATKFTSIPASFWWATITMTTVGYGDIY---------------------
---------PKTLLGKSVGGLCCIAGVLVIALPIPIIVPPFSEFYKEQKRQEK-AIKRREALERAKRNGSIVS---MNLKDAFARSMELI
DVAVEKAGESSSTKDSADDNHLSPSRWKWARKALSETSSNKSYENKYQEVSQKDSHEQLNNTSSSSPQHLSAQKLEMLYN
EITKTQPHPAPNPDGQEQPDRPSAYEEEIEMEEVVCPQEQLAVAQGEVIVDMKSTSSIDSFTSCATDFTETERSPLPPLSAS
HLQMRFPPDLAGTDEHQRARGPPFLMLARGKGPAGRDAILEYAPVDITVSLDASGSKVGSHGPLQPDSASESPKSSLKGS
NPLKSRSLRVNFKENRGSAPQTPPSTARPLPVTTADFSLTAPQLISTILLEETPSQGDRPCWAPRARHTVRDLPRGCPPGFPS
RNSFLSHQERGGASLK--

--</seq-data>
</sequence>
<sequence seq-type="Protein">
<seq-name>CIKA_HUMAN</seq-name>
<seq-info><length>2009</length></seq-info>
<seq-data>--

--MTKHGSRSTSSLPPEPMEIVRSKACSRRVRLNVGGLAHEVLWRTL
DRLPRTRLGKLRDCNTHD--SLLEVCDDYSLDDNEYFFDRHPGAFTSILNFYRTG-RLHM
MEEMCALSFSQELDYWG-IDEIYLESCCQARYHQKKEQMNEELKREAETLREREGEEFDNTCCAEKR------------------------
-----------KKLWDLLEKPNSSVAAKILAIISIMFIVLSTIALSLNTLPELQSLDEF---GQS
TDNPQLAHVEAVCIAWFTMEYLLRFLSSP------------------KKWKFFKGPLNAIDLLAILPYYVTIFLTESNKSVL------------------
---------QFQNVRRVVQIFRIMRILRILKLARHSTGLQSLGFTLRRSYNELGLLILFLAMGIMIFSSLVFFAEK---------------------
---DEDDTKFKSIPASFWWATITMTTVGYGDIY------------------------------PK
TLLGKIVGGLCCIAGVLVIALPIPIIVNNFSEFYKEQKRQEK-AIKRREALERAKRNGSIVS---MNMKDAFARSIEMMDIVVEK
NGENMGKKDKVQDNHLSPNKWKWTKRTLSETSSSKSFETKEQGSPEKARSSSSPQHLNVQQLEDMYNKMAKTQSQPIL
NTKESAAQSKPKEELEMESIPSPVAPLPTRTEGVIDMRSMSSIDSFISCATDFPEATRFSHSPLTSLPSKTGGSTAPEVGWRG
ALGASGGRFVEANPSPDASQHSSFFIESPKSSMKTNNPLKLRALKVNFMEGDPSPLLPVLGMYHDPLRNRGSAAAAVAGL

ECATLLDKAVLSPESSIYTTASAKTPPRSPEKHTAIAFNFEAGVHQYIDADTDDEGQLLYSVDSSPPKSLPGSTSPKFSTGTRSE
KNHFESSPLPTSPKFLRQNCIYSTEALTGKGPSGQEKCKLENHISPDVRVLPGGGAHGSTRDQSI--------------------------------
----
--</seq-data>
</sequence>
<sequence seq-type="Protein">
<seq-name>CIKB_DROME</seq-name>
<seq-info><length>2009</length></seq-info>
<seq-data>--

--MVGQLQGGQAAGQQQQQQQATQQ
QQHSKQQLQQQQQQQQQLQLKQHQQQQQDILYQQHNEAIAIARGLQAATPADIGDNQPYYDTSGNVDWERAMGA
GGAGAYGGIGIGSLPAAGGAAYHLGPANPAGLVSRHLDYGDGGHLAGPSAGLPAGAVGSGAGAGAGAGASVTGS----GS
GAGTGTGTGAGSGSGSGAAGKEVRYAPFPVASPTHSIPTTSQQIVGGVGGVGVGGASSQSISGGVPTHSQSNTTGALQR
THSRSMSSIPPPEPFMIAQSKAVNSRVSINVGGVRHEVLWRTLERLPHTRLGRLGECTTHE-------------------------------------
------AIVELCDDYSLADNEYFFDRHPKSFSSILNFYRTG-KLHIVDEMCVLAFGDDLEYWG-VDELYLESCCQHKYHQRKENV
HEEMRKEAESLRQRDEEEFGEGKFSEYQ-------------------------------------KYLWELLEKPNTSFAARVIAVISILFIVLSTIALTL
NTLPQLQHIDN--GTPQDNPQLAMVEAVCITWFTLEYILRFSSSP-----------------
--DKWKFFKGGLNIIDLLAILPYFVSLFLLETNKNATD-------------------------QFQDVRRVVQVFRIMRILRVLKLARHSTGLQS
LGFTLRNSYKELGLLMLFLAMGVLIFSSLAYFAEK--DEK
DTKFVSIPEAFWWAGITMTTVGYGDIC--------------------------------PTTALGKVIGTVCCICGVLVVALPIPIIVNNFAEFYKN
QMRREK-ALKRREALDRAKREGSIVSFHHINLKDAFAKSMDLIDVIVD-TGKQTNVVHPKGKRQSTPNIGRQTLDVQSAPG
HNLSQTDGNSTEGESTSGRNPATTGTGCYKNYDHVANLRNSNLHNRRGSSSEQDAVPPYSFDNPNARQTSMMAMESY
RRDEQALLQQQQQQQQQMLQMQQIQQKAPNGNGGATGGGVANNLAMVAASSAGTAVATATNASNASNTAPGSEG
AEGGVMEMGAVSMTTTFPRPRDCPSR---

---</seq-data>
</sequence>
<sequence seq-type="Protein">
<seq-name>CIKW_DROME</seq-name>
<seq-info><length>2009</length></seq-info>
<seq-data>--

---MNLINMDSENRVVLNVGGIRHETYKATLKKIPATR
LS---RLTEALANYDPILNEYFFDRHPGVFAQVLNYYRTG-KLHYPTDVCGPLFEEE
LEFWG-LDSNQVEPCCWMTYTQHRD-TQETLAVLDRLDLDTEKPSEEELARKFGFEEDYYKGTISWWQEMK----------------
-----PRIWSLFDEPYSSNAAKTIGVVSVFFICISILSFCLKTHPDMRVPIVRNITVKTANGSNGWFLDKT---------------------------
------QTNAHIAFFYIECVCNAWFTFEILVRFISSP-------------------NKWEFIKSSVNIIDYIATLSFYIDLVLQRFA------------------
------------SHLENADILEFFSIIRIMRLFKLTRHSSGLKILIQTFRASAKELTLLVFFLVLGIVIFASLVYYAEIRI-------------------------

---QPNPHNDFNSIPLGLWWALVTMTTVGYGDMA------------------------------
PKTYIGMFVGALCALAGVLTIALPVPVIVSNFAMYYSHTQARAK-----LPKKRRRVLPVEQPRQPRLPGAPGGVSGCGTPGS
GPHSGPMGSGGTGPRRMNNKTKDLVSPKSDMAFSFD---

---</seq-data>
</sequence>
<sequence seq-type="Protein">
<seq-name>CIKD_HUMAN</seq-name>
<seq-info><length>2009</length></seq-info>
<seq-data>--

--MGQGDESERIVINVGGTRHQTYRSTLRTLPGTRL
AWLAE--PDAHSHFDYDPRADEFFFDRHPGVFAHILNYYRTG-KLHCPADVCGPLY
EEELAFWG-IDETDVEPCCWMTYRQHRDAEEALDSFGGAPLDNSADDADADGPGDSGDGEDELEMTKRLALSDSPDGR
PGGFWRRWQ-------PRIWALFEDPYSSRYARYVAFASLFFILVSITTFCLETHERFNPIVNKTEIENVRNGTQVRYYR----------
-----------------------EAETEAFLTYIEGVCVVWFTFEFLMRVIFCP------------------NKVEFIKNSLNIIDFVAILPFYLEVGLSG
LSS-------------------------KAAKDVLGFLRVVRFVRILRIFKLTRHFVGLRVLGHTLRASTNEFLLLIIFLALGVLIFATMIYYAER
IGAQPNDPS---ASEHTHFKNIPIGFWWAVVTMTTLGYGDMY---
---------------------------PQTWSGMLVGALCALAGVLTIAMPVPVIVNNFGMYYSLAMAKQK-----LPKKKKKHIPRPPQLG
SPNYCKSVVNSPHHSTQSDTCPLAQEEILEINRAGRKPLRGMSI--

--</seq-da
ta>
</sequence>
<sequence seq-type="Protein">
<seq-name>CIKE_RAT</seq-name>
<seq-info><length>2009</length></seq-info>
<seq-data>--

---MGKIENNERVILNVGGTRHETYRSTLKTLPGTRLA
LLASSEPQGDCLTAAGDKLQPLPPPLSPPPRPPPLSPVPSGCFEGGAGNCSSHGGNGSDHPGGGREFFFDRHPGVFAYVL
NYYRTG-KLHCPADVCGPLFEEELAFWG-IDETDVEPCCWMTYRQHRDAEEALDIFETPDLIGGDPGDDEDLGGKRLGIED
AAGLGGPDGKSGRWRKLQ---------------PRMWALFEDPYSSRAARFIAFASLFFILVSITTFCLETHEAFNIVKNKTEPVINGT
SAVLQY-----------------------------------EIETDPALTYVEGVCVVWFTFEFLVRIVFSP------------------NKLEFIKNLLNIIDFV

AILPFYLEVGLSGLSS----------------------------KAAKDVLGFLRVVRFVRILRIFKLTRHFVGLRVLGHTLRASTNEFLLLIIFLALG
VLIFATMIYYAERVGAQPNDPS--ASEHTQFKNIPIGFWWAVVT
MTTLGYGDMY---------------------------------PQTWSGMLVGALCALAGVLTIAMPVPVIVNNFGMYYSLAMAKQK-----LPR
KRKKHIPPAPLASSPTFCKTELNMACNSTQSDTCLGKENRLLEHNRSVLSGDDSTGSEPPLSPPERLPIRRSSTRDKNRRGET
CFLLTTGDYTCASDGGIRKGYEKSRSLNNIAGLAGNALRLSPVTSPYNSPCPLRRSRSPIPSIL--------------------------------------
----

---</seq-data>
</sequence>
<sequence seq-type="Protein">
<seq-name>CIKG_HUMAN</seq-name>
<seq-info><length>2009</length></seq-info>
<seq-data>--

---MISSVCVSSYRGRKSGNKPPSKTCLKEEMAKGEASEKIIINVGGTRHETYRS
TLRTLPGTRLAWLADPDGGGRPETDG--------------------------------------GGVGSSGTSGGGGCEFFFDRHPGVFAYVLNYYR
TG-KLHCPADVCGPLFEEELTFWG-IDETDVEPCCWMTYRQHRDAEEALDIFESPDGGGSGAGPSDEAGDDERELALQRL
GPHEGGAGHGAGSGGCRGWQ----------PRMWALFEDPYSSRAARVVAFASLFFILVSITTFCLETHEAFNIDRNVTEILRVG
NITSVHFRR------------------------------------EVETEPILTYIEGVCVLWFTLEFLVRIVCCP--------------------DTLDFVKNLLNIIDF
VAILPFYLEVGLSGLSS-----------------------------KAARDVLGFLRVVRIVRILRIFKLTRHFVGLRVLGHTLRASTNEFLLLIIFLAL
GVLIFATMIYYAERIGARPSDPR--GNDHTDFKNIPIGFWWAVV
TMTTLGYGDMY---------------------------------PKTWSGMLVGALCALAGVLTIAMPVPVIVNNFGMYYSLAMAKQK-----LPK
KRKKHVPRPAQLESPMYCKSEETSPRDSTCSDTSPPAREEGMIERKRAGEIRGWEGKSLFPQWPREFPNGPQTLGFGMCF
VWGFPKHKDVPL--

--</seq-data>
</sequence>
<sequence seq-type="Protein">
<seq-name>CIKL_DROME</seq-name>
<seq-info><length>2009</length></seq-info>
<seq-data>--

--MASVAAWLPFARAAAIGWVPIATHPLPPPPMPKDRRKTDDEKLLINVSGRRF
ETWRNTLEKYPDTLLGSN---EREFFYDEDCKEYFFDRDPDIFRHILNYYRTG-KL
HYPKHECLTSYDEELAFFG-IMPDVIGDCCYEDYRDRKRENAERLMDDKLSENGDQNLQQLTNMR----------------------------

--------------QKMWRAFENPHTSTSALVFYYVTGFFIAVSVMANVVETVPCGHRPGRAGTL---------PCGERYKI--------------
----------------------VFFCLDTACVMIFTAEYLLRLFAAP-------------------DRCKFVRSVMSIIDVVAIMPYYIGLGITD-----------
---------------------NDDVSGAFVTLRVFRVFRIFKFSRHSQGLRILGYTLKSCASELGFLVFSLAMAIIIFATVMFYAEK-----------
---NVNGTNFTSIPAAFWYTIVTMTTLGYGDMV----------------------
---------PETIAGKIVGGVCSLSGVLVIALPVPVIVSNFSRIYHQNQRADK-----RKAQRKARLARIRIAKASSGAAFVSKKKAAE
ARWAAQESGIELDDNYRDEDIFELQHHHLLRCLEKTTM---

--</seq-data>
</sequence>
<sequence seq-type="Protein">
<seq-name>PKD2_MOUSE</seq-name>
<seq-info><length>2009</length></seq-info>
<seq-data>--

MVNSRRVQPQPPGDAGRSPAPRASGPGRLVAGGAGLAVPGGLGEQRGLEIEMERIRQAAARDPPAGASASPSPPLSSCS
RQAWSRDNPGFEAEEDDDDDEVEGEEGGMVVEMDVEWRPGSRRSASSSAVSSVGARGRGLG----SYRGAAHLSGRRR
RLEDQGAQCPSPAGGGDPLHRHLPLEGQPPRVAWAERLVRGLRGLWGTRLMEESNANREKYLKSVLRELVTYLFFLVVLC
ILTYGMMSSNVYYYTRTLSQLFIDTPVSKTEKTNFKTLSSMEDFWKFTEGSFLDGLYWKAQTSNHTQADNRSFIFYENLLLG
VPRLRQLRVRNGSCSIPQDLRDEIKECYDVYSVSSEDRAPFGPRNGTA--------WIYTSERELNGSSHWGIIASYS-GAGYYLD
LSRTREETAAQ------------------------------------LAGLRRNFWLDRGT-RAAFIDFSVYNANINLFCVVRLLAEFPATGGVVPS
WQFQ------------------------------------PVKLIRYVTAFDFFLAACEIIFCFFIIYYVVEEILEIRI------------------HRLSYFRSFWN
CLDVVIVVLSVVAMVINIYRMSNAEGLLQFLEDQNS----FPNFEHVAYWQIQFNNIAAVMVFLVWIKLFKFINFNRTMSQL
STTMSRCAKDLFGFTIMFSIIFLAYAQLAYLVFG---TQ
VDDFSTFQECIFTQFRIILGDINF---------------------------AEIEEANRVLGPLYFTTFVFFMFFILLNMFLAIINDSYSEVKSDLA
QQK--AEMELSDLIRKGCQKALVKLKLKRNTVDAISESLRQGGGKLNFDELRQDLKGKGHTDAEIEAIFTKYDQDGDQELTE
REHQQMRDDLEKEREDLDLEHSSLPRPMSSRSFPRSLDDSEEEDDEDSGHSSRRRGSISSGVSYEEFQVLVRRVDMEHSI
GSIVSKIDAVIVKLEIMERAKLKRREVLGRLLDGVAEDARLGRDSEIHREQMERLVREELERWESDDAASQTGHGVSTQVG
LGGQPHPRNSRPPSSQSAEGLEGGSGNGSANVHA--

---</seq-data>
</sequence>
<sequence seq-type="Protein">
<seq-name>Q9W4X5</seq-name>
<seq-info><length>2009</length></seq-info>
<seq-data>--

---MFAITVGIIIGCMICIAVF

AGFQHEDVKFKTMLVSIVIVLIFQFLIGDPIKFVILSIDRALWPPRIYIPPRCDKDEMDEKEERRDFLKQRLINKRANLMLTSRY
RNYKLNDQYKMIAHDLFIYGPYFLCLMCLVLVTRDQTLYHNTRIITDLFMYNHTDYTGLQEVYFLNQLFDFIESTLVLAFNSN
STESGSPGWAHAEQSVLLGVMRLRQLRVSNPQVGLGPPKFSDTYYMPDWQLPYRKLHYSDKYWRIYEPWIPI-KMGFEFL
DALLMNFDHVGSLNSYPELAGYVSLLARSAANSMKV---------------------------------------IDYLSENHWLTLNT-SAVFIDFTLY
NVDVNLFSICTLRVEKTPFGGIVPDVQVD--SAKLLENVDQMPYTGLLALLIYVLVFIQFAQ
TLAVKLW-------------------YEPHLLKSVWNKLDLFIFMVNVLVVVLVVLRESLVASMMKKVEGASKM--EFIDFRRPSRMHQ
LTTITIGFLICITTLRLWRVLQFASVFKLFTRTLYLAWSAVASTAIAIVIFLIGFCFAVVTING---
---NYSSNFNKLVKSMVMCMCYSFGFSAQVK---------------------------PSELFHGGKWLGI
LCYGVLAFVIIVLLINVFVSLINDYFTVAKTIRDSER-ENRITFFEFLVVEFSGVLGRFRKLPCWRRNYVRNGRTVAENVSRKLD
SMDRQRLMRRRLRQGRHHVSRPVSAEDALLEYKDRGDKMVNVHYILNIQLTLIRMFLFDEYVEWPQEQQKQPEAQDPD
PNLGPDGSPPQDKRQDEAEA---

--------</seq-data>
</sequence>
<sequence seq-type="Protein">
<seq-name>Q9WTN5</seq-name>
<seq-info><length>2009</length></seq-info>
<seq-data>---

-MSVILDDDVLLILTLDEELSAPLTPSNGLGQEDLPSKNGGGQSGPNSQVPSLVSGADSPPSSPPGHNWEMNYQEAAIYLQ
EGQNNDKFFTHPKDARALAAYLFVHNHFFYMMELLTALLLLLLSLCESPAVPALKLRTYVHATLELFALMVVVFELCMKLR
WL----GFHTFVRHKRTMVKTSVLVVQFIEAIVVLVRQTSHVRVTRALRCIFLVDCRYCGGVRRNLRQIFQSLPPFMDILLLLLF
FMIIFAILGFYLFSTNPSDPYFNTLENSIVNLFVLLTTANFPDVMMPSYSRNPWSCVFFIVYLSIELYFIMNLLLAVVFDTFNDI
EKHKFKSLLLHKRTAIQHAYHLLVSQRRPAGISYRQFEGLMRFYKPRMSARERFLTFKALNQSNTPLLSLKDFYDIYEVAALQ
WKAK-KNRQHWFDELP----------------------RTAFLIFKGINILV-NSKAFQYFMYLVVAVNGVWILVETFMLKGGNFISK------
--HVPWSYLVFLTIYGVELFMKVAGLG---------------------PVEYLSSGWNLFDFSLT
AFAFLGLLALTLNME---------------------------------PFYFIVVLRPLQLLRLFKLKKRYRNVLDTMFELL-PRMASLGLTLLTFYYSF
AIVGMEFFSGRLSPNCSTPARWGLTPIDSSITRLGNKTK--FGRKGYYYLNNFDNILNS
FVTLFELTVVNNWYIIME-------------------------------GVTSQTSHWSRLYFMTFYIVTMVVMTIIVAFILEAFVFR-MNYSRKS
QESEVDSGIVIEKEMSKEELLAILELHREARGTSSDITRLLDTLSQMEKYQQNSMVFLGRQSRTKSDLSLKMYQEEIQEWYEE
HAREQEQQQLRGSAPSPAAQQTPGSRQRSQTVT--

--</seq-data>
</sequence>
<sequence seq-type="Protein">
<seq-name>TRP7_HUMAN</seq-name>
<seq-info><length>2009</length></seq-info>

<seq-data>MEPSALRKAGSEQEEGFEGLPRRVTDLGMVSNLRRSNSSLFKSWRLQCPFGNNDKQESLSSWIPENIKKKEC
VYFVESSKLSDAGKVVCQCGYTHEQHLEEATKPHTFQGTQWDPKKHVQEMPTDAFGDIVFTGLSQKVKKYVRVSQDTPS
SVIYHLMTQHWGLDVPNLLISVTGGAKNFNMKPRLKSIFRRGLVKVAQTTGAWIITGGSHTGVMKQVGEAVRDFSLSSSY
KEGELITIGVATWGTVHRREGLIHPTGSFPAEYILDEDGQGNLTCLDSNHSHFILVDDGTHGQYGVEIPLRTRLEKFISEQTKE
RGGVAIKIPIVCVVLEGGPGTLHTIDNATTNGTPCVVVEGSGRVADVIAQVANLPVSDITISLIQQKLSVFFQEMFETFTESRI
VEWTKKIQDIVRRRQLLTVFREGKDGQQDVDVAILQALLKASRSQDHFGHENWDHQLKLAVAWNRVDIARSEIFMDEW
QWKPSDLHPTMTAALISNKPEFVKLFLENGVQLKEFVTWDTLLYLYENLDPSCLFHSKLQKVLVEDPERPACAPAAPRLQM
HHVAQVLRELLGDFTQPLYPRPRHNDRLRLLLPVPHVKLNVQGVSLRSLYKRSSGHVTFTMDPIRDLLIWAIVQNRRELAGI
IWAQSQDCIAAALACSKILKELS--KEEEDTDSSEE
MLALAEEYEHRAIGVFTECYRKDEERAQKLLTRVSEAWGKTTCLQLALEAKDMKFVSHGGIQAFLTKVWWGQLSVDNGL
WRVTLCMLAFPLLLTGLISFREKR-LQDVGTPAARARAFFTAPVVVFHLNILSYFAFLCLFAYVLMVD-----------------------
-----------------------------FQPVPSWCECAIYLWLFSLVCEEMRQLFYDP---------DECGLMKKAALYFSDFWNKLDVGAI
LLFVAGLTCRLIPA-----------------------TLYPGRVILSLDFILFCLRLMHIFTISKTLGPKIII-------VKRMMKDVFFFLFLLAVW
VVSFGVAKQAILIH---NERRVDWLFRGAVYHSYLTIFGQIP
GYIDGVNFNPEHCSPNGTDPYKPKCPESDATQQRPAFPEWLTVLLLCLYLLFTNILLLNLLIAMFNYTFQQVQEHTDQIWKF
QRHDLIEEYH-GRPAAPPPFILLSHLQLFIKRVVLKTPAKRHKQLKNKLEKNEEAALLSWEIYLKENYLQNRQFQQKQRPEQK
IEDISNKVDAMVDLLDLDPLKRSGSMEQRLASLEEQVAQTARALHWIVRTLRASGFSSEADVPTLASQKAAEEPDAEPGGR
KKTEEPGDSYHVNARHLLYPNCPVTRFPVPNEKVPWETEFLIYDPPFYTAERKDAAAMDPMGDTLEPLSTIQYNVVDGLR
DRRSFHGPYTVQAGLPLNPMGRTGLRGRGSLSCFGPNHTLYPMVTRWRRNEDGAICRKSIKKMLEVLVVKLPLSEHWAL
PGGSREPGEMLPRKLKRILRQEHWPSFENLLKCGMEVYKGYMDDPRNTDNAWIETVAVSVHFQDQNDVELNRLNSNLH
ACDSGASIRWQVVDRRIPLYANHKTLLQKAAAEFGAHY---

------------------------</seq-data>
</sequence>
<sequence seq-type="Protein">
<seq-name>O46129</seq-name>
<seq-info><length>2009</length></seq-info>
<seq-data>--

---------------MSKKPPRKRDLNNEERQFLGAVARGDVGSVKQALAEAEERFIDISCKDSLGRSSLVIAIENENDDLVSLLLNY
DVDLEDSLLHAIREEYVVAVEMILTHQLKKFGEDYLQIEFKSNTFTADITPIILAGHIDNYEIIKMLLDRGYRIPKPHDLTCHCD
DCLRGSLVDVLGHSRARINAYRALASPSLIALSSKDPILTAFELSWELRNLSEMENEFRAEYEELGITCQNFAVDLLEQIRGSK
ELEVILNHDCRSPDQDEEKELKKLSRLKLAIKY---

-----------KQKKFVAHPNCQQLLAAKWYEGLGNFRRKPILKQLTVAFGILFGFPIL-SGIYMIAPHSKFGKLCRKPFIKFLLHSA
SYLSFLVLLILVSQRIENPFFFLFPMK--EEKKISELRGSPATIVELMILGYVI
GLIW-------SEIKQIWMQGALEYIHDMWNIMDLVTNSLYMATFTLRLLAWLQVRRERAELNPYAFEERKHWDAYDPNLIA
EALFAGANIFSSLKLIYIFTVNAHLGPLQIS-------LGRMVSDIVKFISVYFLVLFSFACGLNHLYWYYAALRAEECEAGITHS-----
---CDIKYRSFANLFEILQSLYWAVYGLVDLEHA----------------------HLDEPHK
LTELIGKLMFGSYSMIAIIILLNLLIAMMSNSYQLIYSQADEEWKFARSRLWISYFAEGATVPPPFNIIPSPKSFTNMMTLFKSL
ILSHTAEQKQAKWTNVRSTVKNINKREIRYQYVIRNLISRYIMNKQRPQKDEMVSQDDINELKQDISTFRFELLTILSDNGFE
TPTVHQAKTSSRLDRMWKNLSAATEGQTETLMEEAGLDEE---

-------------------------</seq-data>
</sequence>
<column-score>
<colsco-name>coreblock</colsco-name>
<colsco-owner>0</colsco-owner>
<colsco-type>int</colsco-type>
<colsco-data>0 0 0 0 0 0 0 0 0 0 0 0 0 0 0 0 0 0 0 0 0 0 0 0 0 0 0 0 0 0 0 0 0 0 0 0 0 0 0 0 0 0 0 0 0 0 0 0 0 0 0 0
0 0 0 0 0 0 0 0 0 0 0 0 0 0 0 0 0 0 0 0 0 0 0 0 0 0 0 0 0 0 0 0 0 0 0 0 0 0 0 0 0 0 0 0 0 0 0 0 0 0 0 0 0 0 0 0 0 0 0
0 0 0 0 0 0 0 0 0 0 0 0 0 0 0 0 0 0 0 0 0 0 0 0 0 0 0 0 0 0 0 0 0 0 0 0 0 0 0 0 0 0 0 0 0 0 0 0 0 0 0 0 0 0 0 0 0 0 0
0 0 0 0 0 0 0 0 0 0 0 0 0 0 0 0 0 0 0 0 0 0 0 0 0 0 0 0 0 0 0 0 0 0 0 0 0 0 0 0 0 0 0 0 0 0 0 0 0 0 0 0 0 0 0 0 0 0 0
0 0 0 0 0 0 0 0 0 0 0 0 0 0 0 0 0 0 0 0 0 0 0 0 0 0 0 0 0 0 0 0 0 0 0 0 0 0 0 0 0 0 0 0 0 0 0 0 0 0 0 0 0 0 0 0 0 0 0
0 0 0 0 0 0 0 0 0 0 0 0 0 0 0 0 0 0 0 0 0 0 0 0 0 0 0 0 0 0 0 0 0 0 0 0 0 0 0 0 0 0 0 0 0 0 0 0 0 0 0 0 0 0 0 0 0 0 0
0 0 0 0 0 0 0 0 0 0 0 0 0 0 0 0 0 0 0 0 0 0 0 0 0 0 0 0 0 0 0 0 0 0 0 0 0 0 0 0 0 0 0 0 0 0 0 0 0 0 0 0 0 0 0 0 0 0 0
0 0 0 0 0 0 0 0 0 0 0 0 0 0 0 0 0 0 0 0 0 0 0 0 0 0 0 0 0 0 0 0 0 0 0 0 0 0 0 0 0 0 0 0 0 0 0 0 0 0 0 0 0 0 0 0 0 0 0
0 0 0 0 0 0 0 0 0 0 0 0 0 0 0 0 0 0 0 0 0 0 0 0 0 0 0 0 0 0 0 0 0 0 0 0 0 0 0 0 0 0 0 0 0 0 0 0 0 0 0 0 0 0 0 0 0 0 0
0 0 0 0 0 0 0 0 0 0 0 0 0 0 0 0 0 0 0 0 0 0 0 0 0 0 0 0 0 0 0 0 0 0 0 0 0 0 0 0 0 0 0 0 0 0 0 0 0 0 0 0 0 0 0 0 0 0 0
0 0 0 0 0 0 0 0 0 0 0 0 0 0 0 0 0 0 0 0 0 0 0 0 0 0 0 0 0 0 0 0 0 0 0 0 0 0 0 0 0 0 0 0 0 0 0 0 0 0 0 0 0 0 0 0 0 0 0
0 0 0 0 0 0 0 0 0 0 0 0 0 0 0 0 0 0 0 0 0 0 0 0 0 0 0 0 0 0 0 0 0 0 0 0 0 0 0 0 0 0 0 0 0 0 0 0 0 0 0 0 0 0 0 0 0 0 0
0 0 0 0 0 0 0 0 0 0 0 0 0 0 0 0 0 0 0 0 0 0 0 0 0 0 0 0 0 0 0 0 0 0 0 0 0 0 0 0 0 0 0 0 0 0 0 0 0 0 0 0 0 0 0 0 0 0 0
0 0 0 0 0 0 0 0 0 0 0 0 0 0 0 0 0 0 0 0 0 0 0 0 0 0 0 0 0 0 0 0 0 0 0 0 0 0 0 0 0 0 0 0 0 0 0 0 0 0 0 0 0 0 0 0 0 0 0
0 0 0 0 0 0 0 0 0 0 0 0 0 0 0 0 0 0 0 0 0 0 0 0 0 0 0 0 0 0 0 0 0 0 0 0 0 0 0 0 0 0 0 0 0 0 0 0 0 0 0 0 0 0 0 0 0 0 0
0 0 0 0 0 0 0 0 0 0 0 0 0 0 0 0 0 0 0 0 0 0 0 0 0 0 0 0 0 0 0 0 0 0 0 0 0 0 0 0 0 0 0 0 0 0 0 0 0 0 0 0 0 0 0 0 0 0 0
0 0 0 0 0 0 0 0 0 0 0 0 0 0 0 0 0 0 0 0 0 0 0 0 0 0 0 0 0 0 0 0 0 0 0 0 0 0 0 0 0 0 0 0 0 0 0 0 0 0 0 0 0 0 0 0 0 0 0
0 0 0 0 0 0 0 0 0 0 0 0 0 0 0 0 0 0 0 0 1 1 1 1 1 1 1 1 1 1 1 1 1 1 1 1 1 1 0 0 0 0 0 0 0 0 0 0 0 0 0 0 0 0 0 0 0 0 0
0 0 0 0 0 0 0 0 0 0 0 0 0 0 0 0 0 0 0 0 0 0 0 0 0 1 1 1 1 1 1 1 1 1 1 1 1 1 1 1 1 1 0 0 0 0 0 0 0 0 0 0 0 0 0 0 0 0 0
0 0 0 0 0 0 0 0 0 0 0 0 0 0 0 0 0 0 0 0 0 0 0 0 0 0 0 0 0 0 0 0 0 0 0 0 0 0 0 0 0 0 0 0 0 0 0 0 0 0 0 0 0 0 0 0 0 0 0
0 0 0 0 0 0 0 0 0 0 0 0 0 0 0 0 0 0 0 0 0 0 0 0 0 0 0 0 0 0 0 0 0 0 0 0 0 0 0 0 0 0 0 0 0 0 0 0 0 0 0 0 0 0 0 0 0 0 0
0 0 0 0 0 0 0 0 0 0 0 0 0 0 0 0 0 0 0 0 0 0 0 0 0 0 0 0 0 0 0 0 0 0 0 0 0 0 0 0 0 0 0 0 0 0 0 0 0 0 1 1 1 1 1 1 1 1 1
1 1 1 1 1 1 1 1 1 1 1 1 1 1 1 1 1 0 0 0 0 0 0 0 0 0 0 0 0 0 0 0 0 0 0 0 0 0 0 0 0 0 0 0 0 0 0 0 0 0 0 0 0 0 0 0 0 0 0
0 0 0 0 0 0 0 0 0 0 0 0 0 0 0 0 0 0 0 0 0 0 0 0 0 0 0 0 0 0 0 0 0 0 0 0 0 0 0 0 0 0 0 0 0 0 0 0 0 0 0 0 0 0 0 0 0 0 0
0 0 0 0 0 0 0 0 0 0 0 0 0 0 0 0 0 0 0 0 0 0 0 0 0 0 0 0 0 0 0 0 0 0 0 0 0 0 0 0 0 0 0 0 0 0 0 0 0 0 0 0 0 0 0 0 0 0 0
0 0 0 0 0 0 0 0 0 0 0 0 0 0 0 0 0 0 0 0 0 0 0 0 0 0 0 0 0 0 0 0 0 0 0 0 0 0 0 0 0 0 0 0 0 0 0 0 0 0 0 0 0 0 0 0 0 0 0
0 0 0 0 0 0 0 0 0 0 0 0 0 0 0 0 0 0 0 0 0 0 0 0 0 0 0 0 0 0 0 0 0 0 0 0 0 0 0 0 0 0 0 0 0 0 0 0 0 0 0 0 0 0 0 0 0 0 0
0 0 0 0 0 0 0 0 0 0 0 0 0 0 0 0 0 0 0 0 0 0 0 0 0 0 0 0 0 0 0 0 0 0 0 0 0 0 0 0 0 0 0 0 0 0 0 0 0 0 0 0 0 0 0 0 0 0 0
0 0 0 0 0 0 0 0 0 0 0 0 0 0 0 0 0 0 0 0 0 0 0 0 0 0 0 0 0 0 0 0 0 0 0 0 0 0 0 0 0 0 0 0 0 0 0 0 0 0 0 0 0 0 0 0 0 0 0
0 0 0 0 0 0 0 0 0 0 0 0 0 0 0 0 0 0 0 0 0 0 0 0 0 0 0 0 0 0 0 0 0 0 0 0 0 0 0 0 0 0 0 0 0 0 0 0 0 0 0 0 0 0 0 0 0 0 0
0 0 0 0 0 0 0 0 0 0 0 0 0 0 0 0 0 0 0 0 0 0 0 0 0 0 0 0 0 0 0 0 0 0 0 0 0 0 0 0 0 0 0 0 0 0 0 0 0 0 0 0 0 0 0 0 0 0 0
0 0 0 0 0 0 0 0 0 0 0 0 0 0 0 0 0 0 0 0 0 0 0 0 0 0 0 0 0 0 0 0 0 0 0 0 0 0 0 0 0 0 0 0 0 0 0 0 0 0 0 0 0 0 0 0 0 0 0
0 0 0 0 0 0 0 0 0 0 0 0 0 0 0 0 0 0 0 0 0 0 0 0 0 0 0 0 0 0 0 0 0 0 0 0 0 0 0 0 0 0 0 0 0 0 0 0 0 0 0 0 0 0 0 0 0 0 0

0 0 0 0 0 0 0 0 0 0 0 0 0 0 0 0 0 0 0 0 0 0 0 0 0 0 0 0 0 0 0 0 0 0 0 0 0 0 0 0 0 0 0 0 0 0 0 0 0 0 0 0 0 0 0 0 0 0 0 0 0 0 0 0
0 0 0 0 0 0 0 0 0 0 </colsco-data>
</column-score>
</alignment>
</macsim>
:::::::::::::
msl_core-block.xml
:::::::::::::
<?xml version="1.0"?>
<!DOCTYPE macsim SYSTEM "http://www-bio3d-igbmc.u-strasbg.fr/macsim.dtd">
<macsim>
<alignment>
<aln-name>1</aln-name>
<sequence seq-type="Protein">
<seq-name>MSCL_ECOLI</seq-name>
<seq-info><length>185</length></seq-info>
<seq-data>---------MSIIKEFREFAMRGNVVDLAVGVIIGAAFGKIVSSLVADIIMPPLGLLIGGIDFKQFAVTLRDAQ-------
GDIPAVVMHYGVFIQNVFDFLIVAFAIFMAIKLINKLN---RKKEE--PAAAP-APT----KEEVLLTEIRDLLKEQNNRS------------
-----------</seq-data>
</sequence>
<sequence seq-type="Protein">
<seq-name>MSCL_ERWCA</seq-name>
<seq-info><length>185</length></seq-info>
<seq-data>---------MSIIKEFREFAMRGNVVDLAVGVIIGALFGKIVSSLVSDIIMPPLGLLIGGVDFKQFALFLRNAQ-------G
GIPAVVMNYGAFIQNIFDFIIVAFAIFIAIKLMNKM----RCKQEDTPAAPP-KPS----AEEKLLAEIRDLLKEQQTRQ-------------
----------</seq-data>
</sequence>
<sequence seq-type="Protein">
<seq-name>MSCL_HAEIN</seq-name>
<seq-info><length>185</length></seq-info>
<seq-data>---------MNFIKEFREFAMRGNVVDMAVGVIIGSAFGKIVSSLVSDIFTPVLGILTGGIDFKDMKFVLAQAQ-----
--GDVPAVTLNYGLFIQNVIDFIIIAFAIFMMIKVINKV----RKPEEKKT------AP----KAETLLTEIRDLLKNK----------------------
----</seq-data>
</sequence>
<sequence seq-type="Protein">
<seq-name>MSCL_PSEFL</seq-name>
<seq-info><length>185</length></seq-info>
<seq-data>---------MGLLSEFKAFAVKGNVVDMAVGIIIGAAFGKIVSSFVGDVIMPPIGLLIGGVDFSDLAITLKAE--------G
DVPAVVLAYRKFIQTVLNFVIVAFAIFMGVKAINRL----KREEAVAPSEPP-VPS----AEETLLTEIRDLLKAQQNKS-------------
----------</seq-data>
</sequence>
<sequence seq-type="Protein">

<seq-name>Q9RH75</seq-name>
<seq-info><length>185</length></seq-info>
<seq-data>-MSGVDEKGRRMLKEFREFAMKGNVVDLAVGVIIRRAFGAIVTSLVGDVIMPLIGAVTGGLDFSNYFTPLSKA
VTATNLADAKKQGAVLAWGSFLTLTINFIIIAFVLFLVIRAINTL----KRKEEAAPAAPP-KPS----AEVELLTEIRDLLKKSCTRTL
RPNC------------------</seq-data>
</sequence>
<sequence seq-type="Protein">
<seq-name>MSCL_BACSU</seq-name>
<seq-info><length>185</length></seq-info>
<seq-data>-----------MWNEFKAFAMRGNIVDLAIGVVIGGAFGKIVTSLVNDIIMPLVGLLLGGLDFSGLSFTFG------------
--DAVVKYGSFIQTIVNFLIISFSIFIVIRTLNGL---RRKKEAEEEAAEEAVD-----AQEELLKEIRDLLKQQAKSPE------------------
----</seq-data>
</sequence>
<sequence seq-type="Protein">
<seq-name>MSCL_CLOPE</seq-name>
<seq-info><length>185</length></seq-info>
<seq-data>-----------MWKEFKEFAMKGNVIDLAIGVVIGGAFGKIVTSLVNDIIMPVVGSLVGKVDFSNLYINLSGQQFNS
LQEAQAAGAATINYGLFLNNLINFLIIAFSIFIVIKQINKLKNFTKKKEEVKVEATEKDC-----PYCYTKIDIKATRCPHCTSVLEEA
TN-----------------</seq-data>
</sequence>
<sequence seq-type="Protein">
<seq-name>MSCL_CLOHI</seq-name>
<seq-info><length>185</length></seq-info>
<seq-data>-----------MWKDFKEFAMKGNVVDLAVGVIIGGAFGKIVTSLVNDVIMPILGLILGGINFTSAKLTLHGLN-------
-SEKPLTLNYGQFIQNILDFLIISFSIFLFIRLINRF----KRKEEAVEEAKIPEIS----REEELLGEIRDLLKEKNK-------------------------
</seq-data>
</sequence>
<sequence seq-type="Protein">
<seq-name>MSCL_SYNY3</seq-name>
<seq-info><length>185</length></seq-info>
<seq-data>MVKSARQGAGGFWRDFKDFILRGNVVDLAVAVVIGGAFTSIVNAFVAWLMAVLLQPVL---DQAGVSQLQD
L---------------PLGLGELVIAIINFLIIAFVIFLIIKAIEKMQ---RKKAVEEEIVAEAQPDPVLEAQTNLTDSINRLITTLENQQSSSQ
-------------------</seq-data>
</sequence>
<sequence seq-type="Protein">
<seq-name>MSCL_STAAU</seq-name>
<seq-info><length>185</length></seq-info>
<seq-data>-----------MLKEFKEFALKGNVLDLAIAVVMGAAFNKIISSLVENIIMPLIGKIFGSVDFAKEWSFWG-------------
----IKYGLFIQSVIDFIIIAFALFIFVKIANTLM---KKEEAEEEAV---VE-----ENVVLLTEIRDLLREKK--------------------------</se
q-data>
</sequence>
<sequence seq-type="Protein">

<seq-name>MSCL_MYCTU</seq-name>
<seq-info><length>185</length></seq-info>
<seq-data>-----------MLKGFKEFLARGNIVDLAVAVVIGTAFTALVTKFTDSIITPLINRIGVNAQSDVGILRIGI-----------GG
GQTIDLNVLLSAAINFFLIAFAVYFLVVLPYNTL---RKKGEVEQPGD---------TQVVLLTEIRDLLAQTNGDSPGRHGGRGTPS
PTDGPRASTESQ</seq-data>
</sequence>
<sequence seq-type="Protein">
<seq-name>1msl</seq-name>
<seq-info><length>185</length></seq-info>
<seq-data>--------------------ARGNIVDLAVAVVIGTAFTALVTKFTDSIITPLINRIGVNAQSDVGILRIGI-----------GGGQTI
DLNVLLSAAINFFLIAFAVYFLVVLPYNTL---RKKGEVEQPGD---------TQVVLLTEIR--------------------------------</seq-da
ta>
</sequence>
<sequence seq-type="Protein">
<seq-name>MSCL_MYCLE</seq-name>
<seq-info><length>185</length></seq-info>
<seq-data>-----------MFRGFKEFLSRGNIVDLAVAVVIGTAFTALITKFTDSIITPLINRVGVNQQTNISPLRIDI-----------GG
DQAIDLNIVLSAAINFLLIALVVYFLVVLPYTTI---RKHGEVEQFDTDLIG-----NQVVLLAEIRDLLAQSNGAPSGRHVDTADLT
PTPNHEPRADT-</seq-data>
</sequence>
<sequence seq-type="Protein">
<seq-name>Q9RRR4</seq-name>
<seq-info><length>185</length></seq-info>
<seq-data>-----------MLNGFRDFILRGNVVDLAVGVVIGAAFNNVVAAFTKAFLDPLIRLATGGHGKVAGTFAVN----------
----GITFDWGTFVSTLINFLLTAAVLYFFVVMPMNA---ATERLKRSEKAAEAEPS----NEEKLLAEIRDAVRSRPL---------------
----------</seq-data>
</sequence>
<column-score>
<colsco-name>coreblock</colsco-name>
<colsco-owner>0</colsco-owner>
<colsco-type>int</colsco-type>
<colsco-data>0 0 0 0 0 0 0 0 0 0 0 0 0 0 0 0 0 0 0 0 0 0 0 1 1 1 1 1 1 1 1 1 1 1 1 1 1 1 1 1 1 1 1 1 1 1 1 1 1 1
1 1 1 1 1 1 0 0 0 0 0 0 0 0 0 0 0 0 0 0 0 0 0 0 0 0 0 0 0 0 0 0 0 0 0 0 0 0 1 1 1 1 1 1 1 1 1 1 1 1 1 1 1 1 1 1 1 1 1
1 1 1 1 1 0 0 0 0 0 0 0 0 0 0 0 0 0 0 0 0 0 0 0 0 0 0 0 0 0 0 0 0 0 0 0 0 0 0 0 0 0 0 0 0 0 0 0 0 0 0 0 0 0 0 0 0 0 0
0 0 0 0 0 0 0 0 0 0 0 0 0 0 0 </colsco-data>
</column-score>
</alignment>
</macsim>
::::::::::::::
photo_core-block.xml
::::::::::::::
<?xml version="1.0"?>

<!DOCTYPE macsim SYSTEM "http://www-bio3d-igbmc.u-strasbg.fr/macsim.dtd">
<macsim>
<alignment>
<aln-name>1</aln-name>
<sequence seq-type="Protein">
<seq-name>BACH_HALHP</seq-name>
<seq-info><length>387</length></seq-info>
<seq-data>--------------------MTAASTTATTMLQATQSDVLQEIQSNFLLNSSIWVNIALAGVVILLFVAMGRD---IESPRAK
LIWVATMLVPLVSISSYAGLASGLTVGFLQMPPGHALAG----------QEVLSPWGRYLTWTFSTPMILLALGLLA-------DTDIA
SLFTAITMDIGMCVTGLAAALITS-----SHLLRWVFYGISCAFFVAVLYVLLVQWPADA---EAAGTSEIFGTLKILTVVLW-LGY
PILWALGSEG-VALL-SVGVTSWGYSGLDILAKYVFAFLLLRWVAANEGAVSGSGMSIGS---GGAAPADD--------------------
-------------------------------------</seq-data>
</sequence>
<sequence seq-type="Protein">
<seq-name>BACH_HALHS</seq-name>
<seq-info><length>387</length></seq-info>
<seq-data>--------------------MTAVSTTATTVLQATQSDVLQEIQSNFLLNSSIWVNIALAGVVILLFVAMGRD---LESPRAK
LIWVATMLVPLVSISSYAGLASGLTVGFLQMPPGHALAG----------QEVLSPWGRYLTWTFSTPMILLALGLLA-------DTDIA
SLFTAITMDIGMCVTGLAAALITS-----SHLLRWVFYGISCAFFVAVLYVLLVQWPADA---EAAGTSEIFGTLKILTVVLW-LGY
PILWALGSEG-VALL-SVGVTSWGYSGLDILAKYVFAFLLLRWVAANEGTVSGSGMGIGS---GGAAPADD--------------------
-------------------------------------</seq-data>
</sequence>
<sequence seq-type="Protein">
<seq-name>BACH_HALVA</seq-name>
<seq-info><length>387</length></seq-info>
<seq-data>--------------------MPAASTAATTLLQASQSEVLGEIQSNFLLNSSLWVNIALAGVVILLFVAMGRE---LESSRAKL
IWVATMLVPLVSISSYAGLASGLTVGFLQMPPGHALAG----------QEVLSPWGRYLTWTFSTPMILLALGLLA-------DTDM
ASLFTAITMDIGMCITGLAAALVTS-----SHLLRWVFYGISCAFFIAVLYVLLVEWPADA---EAAGTSEIFGTLKLLTVVLW-LG
YPILWALGSEG-VALL-SVGVTSWGYSGLDILAKYVFAFLLLRWVAANEDTVTQAGMSLGS---GGAAPADD------------------
---------------------------------------</seq-data>
</sequence>
<sequence seq-type="Protein">
<seq-name>BACH_HALSS</seq-name>
<seq-info><length>387</length></seq-info>
<seq-data>---------------MIETAAADILAGGMVPLEMTQTQIFEAVQSDTLLASSLWINIALAGLSILLFVYMGRN---VEDPR
AQLIFVATLMVPLVSISSYTGLVSGLTVSFLEMPAGHALAG----------QEVLTPWGRYLTWALSTPMILIAVGLLA-------GSN
TTKLFTAVVADIGMCVTGLAAALTTS-----SYLLRWVWYAISCAFFVVVLYILLAEWAEDA---EIAGTADIFNTLKVLTVVLW-
LGYPIFWALGAEG-LAVL-DVAITSWAYSGMDIVAKYLFAFLLLRWVVNNERTVADVASGLGSGSRGGAAPADD------------
---</seq-data>
</sequence>
<sequence seq-type="Protein">
<seq-name>O93742</seq-name>

<seq-info><length>387</length></seq-info>
<seq-data>---------------MMETA-ADALASGTVPLEMTQTQIFEAIQGDTLLASSLWINIALAGLSILLFVYMGRN---LEDPR
AQLIFVATLMVPLVSISSYTGLVSGLTVSFLEMPAGHALAG----------QEVLTPWGRYLTWALSTPMILVALGLLA-------GSN
ATKLFTAVTADIGMCVTGLAAALTTS-----SYLLRWVWYVISCAFFVVVLYVLLAEWAEDA---EVAGTAEIFNTLKLLTVVLW-
LGYPIFWALGAEG-LAVL-DVAVTSWAYSGMDIVAKYLFAFLLLRWVVDNERTVAGMAAGLGAPL-ARCAPADD-----------
---</seq-data>
</sequence>
<sequence seq-type="Protein">
<seq-name>BACH_HALHM</seq-name>
<seq-info><length>387</length></seq-info>
<seq-data>--IALAGLSILLFVYMGRN---VEDPRAQLIFVATLMVPLVSISSY
TGLVSGLTVGFLEMPAGHALAGMGAGPE----GGVFTPWGRYLTWAFSTPMILIALGLLA-------GSNMSKLFTAVVADVG
MCITGLAAALTTS-----SYLLRWVWYGISCAFFVVVLYILLAEWAKDA---EVAGTADIFNTLKVLTVVLW-LGYPIFWALGAE
G-LAVL-DIAITSWAYSGM---</seq-d
ata>
</sequence>
<sequence seq-type="Protein">
<seq-name>BACH_NATPH</seq-name>
<seq-info><length>387</length></seq-info>
<seq-data>---------------MTETLPPVTESAVALQAEVTQRELFEFVLNDPLLASSLYINIALAGLSILLFVFMTRG---LDDPRAK
LIAVSTILVPVVSIASYTGLASGLTISVLEMPAGHFAEGSSVMLGGEEVDGVVTMWGRYLTWALSTPMILLALGLLA-------G
SNATKLFTAITFDIAMCVTGLAAALTTS-----SHLMRWFWYAISCACFLVVLYILLVEWAQDA---KAAGTADMFNTLKLLTV
VMW-LGYPIVWALGVEG-IAVL-PVGVTSWGYSFLDIVAKYIFAFLLLNYLTSNESVVSG--SILDVPS-ASGTPADD------------
--</seq-data>
</sequence>
<sequence seq-type="Protein">
<seq-name>BACH_HALSP</seq-name>
<seq-info><length>387</length></seq-info>
<seq-data>--------------------MSITSVPGVVDAGVLGAQSAAAVRENALLSSSLWVNVALAGIAILVFVYMGRT---IRPGRP
RLIWGATLMIPLVSISSYLGLLSGLTVGMIEMPAGHALAG----------EMVRSQWGRYLTWALSTPMILLALGLLA-------DVD
LGSLFTVIAADIGMCVTGLAAAMTTS-----ALLFRWAFYAISCAFFVVVLSALVTDWAASA---SSAGTAEIFDTLRVLTVVLW-
LGYPIVWAVGVEG-LALVQSVGVTSWAYSVLDVFAKYVFAFILLRWVANNERTVAVAGQTLGT------MSSDD---------------
---</seq-data>
</sequence>
<sequence seq-type="Protein">
<seq-name>O93741</seq-name>
<seq-info><length>387</length></seq-info>
<seq-data>MRSRTYHDQSVCGPYGSQRTDCDRDTDAGSDTDVHGAQVATQIRTDTLLHSSLWVNIALAGLSILVFLYMA
RT---VRANRARLIVGATLMIPLVSLSSYLGLVTGLTAGPIEMPAAHALAG----------EDVLSQWGRYLTWTLSTPMILLALGW
LA-------EVDTADLFVVIAADIGMCLTGLAAALTTS-----SYAFRWAFYLVSTAFFVVVLYALLAKWPTNA---EAAGTGDIFGT
LRWLTVILW-LGYPILWALGVEG-FALVDSVGLTSWGYSLLDIGAKYLFAALLLRWVANNERTIAVGQRSGRGAIGDPVED--
---</seq-data>

</sequence>
<sequence seq-type="Protein">
<seq-name>BACT_HALVA</seq-name>
<seq-info><length>387</length></seq-info>
<seq-data>---MATITTWFTLGLLGELLGTAVLAYGYT----LVPEETRKRYLLLIAIPGIAI
VAYALMALGFGSIQS--------------------EGHAVYVVRYVDWLLTTPLNVWFLALLA-------GASREDTVKLVVLQALTIVFGF
AGAVTPS-------PVSYALFAVGGALFGGVIYLLYRNIAVAAKSTLSDIEVSLYRTLRNFVVVLW-LVYPVVWLLGAAG-VGLM-
DVETATLVVVYLDVVTKVGFGVIALLAMIDLGSAGETAEEPTAVAGD--
-----</seq-data>
</sequence>
<sequence seq-type="Protein">
<seq-name>BACT_NATPH</seq-name>
<seq-info><length>387</length></seq-info>
<seq-data>---MVGLTTLFWLGAIGMLVGTLAFAWAGR----DAGSGERRYYVTLVGI
SGIAAVAYVVMALGVGWVPV--------------------AERTVFAPRYIDWILTTPLIVYFLGLLA-------GLDSREFGIVITLNTVVML
AGFAGAMVPG-------IERYALFGMGAVAFLGLVYYLVGPMTESASQ-RSSGIKSLYVRLRNLTVILW-AIYPFIWLLGPPG-VA
LL-TPTVDVALIVYLDLVTKVGFGFIALDAAATLRAEHGESLAGVDTDAPAVAD--
-----------</seq-data>
</sequence>
<sequence seq-type="Protein">
<seq-name>BACT_HALSA</seq-name>
<seq-info><length>387</length></seq-info>
<seq-data>--MALTTWFWVGAVGMLAGTVLPIRDC-----IRHPSHRRYDLVLAGIT
GLAAIAYTTMGLGITATTV--------------------GDRTVYLARYIDWLVTTPLIVLYLAMLA-------RPGHRTSAWLLAADVFVIA
AGIAAALTTG-------VQRWLFFAVGAAGYAALLYGLLGTLPRALG--DDPRVRSLFVTLRNITVVLW-TLYPVVWLLSPAG-IGI
L-QTEMYTIVVVYLDFISKVAFVAFAVLGADAVSRLVAADAAAPATAEPTPDGD--
------------</seq-data>
</sequence>
<sequence seq-type="Protein">
<seq-name>BACS_HALHA</seq-name>
<seq-info><length>387</length></seq-info>
<seq-data>---MDAVATAYLGGAVALIVGVAFVWLLYRS---LDGSPHQSALAPLAIIP
VFAGLSYVGMAYDIGTVIV--------------------NGNQIVGLRYIDWLVTTPILVGYVGYAA-------GASRRSIIGVMVADALMIA
VGAGAVVTDG-------TLKWALFGVSSIFHLSLFAYLYVIFPRVVP--DVPEQIGLFNLLKNHIGLLW-LAYPLVWLFGPAG-IGE
A-TAAGVALTYVFLDVLAKVPYVYFFYARRRVFMHSESPPAPEQATVEATAAD--
-----------</seq-data>
</sequence>
<sequence seq-type="Protein">
<seq-name>BAC3_HALVA</seq-name>
<seq-info><length>387</length></seq-info>
<seq-data>---MDAVAVVYGITAAGFAVGVAIVGYLYAS---LEGSEERSILAALALIPGF
AGISYVAMAFGIGTVTI--------------------GETTLVGFRYLDWVVTTPLLVGFVGYAA-------GASRRAIFGVMVADALMILT

GVGAVVADG-------TLKWVLFGVSTVFHVSLFAYLYLVFPRSVP--DDPQRIGLFSLLKNHIGLLW-IAYPLVWLAGPEG-LGL
A-TYVGVSITYAFLDLLAKVPYVYFFYARRQVFATKLLRDSGEVTATPAD--
------</seq-data>
</sequence>
<sequence seq-type="Protein">
<seq-name>O93743</seq-name>
<seq-info><length>387</length></seq-info>
<seq-data>--MTGAVTSAYWLAAVAFLIGVGITAALYAK---LEGSRARTRLAALAVIP
GFAGLSYVGMALGIGTVTV--------------------NGAELVGLRYVDWVVTTPLLVGFIGYNA-------GASRRAIAGVMIADALMI
VFGAAAVVSGG-------TLKWALFGVSALFHVSLFAYLYVIFPGGIP--DDPMQRGLFSLLKNHVGLLW-LAYPFVWLMGPAG
-IGFT-GAVGAALTYAFLDVLAKVPYVYFFYARRQAFIDVTDSRAAAKGDGPAVGGEAPVATGDDAPTAAD------------------
-----------------------------</seq-data>
</sequence>
<sequence seq-type="Protein">
<seq-name>BACS_HALSS</seq-name>
<seq-info><length>387</length></seq-info>
<seq-data>--MTGAVSAAYWIAAVAFLVGLGITAALYAK---LGESEDRGRLAALAVIP
GFAGLAYAGMALGIGTVTV--------------------NGAELVGLRYVDWIVTTPLLVGFIGYVA-------GASRRAIAGVMLADALMI
AFGAGAVVTGG-------TLKWVLFGVSSIFHVTLFAYLYVVFPRAVP--DDPMQRGLFSLLKNHVGLLW-LAYPFVWLMGPA
G-IGFT-TGVGAALTYAFLDVLAKVPYVYFFYARRQAFTDVVSAATADREDATDAVGDGAPTAAD----------------------------
--------------------------</seq-data>
</sequence>
<sequence seq-type="Protein">
<seq-name>BACR_HALHA</seq-name>
<seq-info><length>387</length></seq-info>
<seq-data>---------------------------MLELLPTAVEGVSQAQITGRPEWIWLALGTALMGLGTLYFLVKGMG---VSDPDAKK
FYAITTLVPAIAFTMYLSMLLGYGLTMVPFGG----------------EQNPIYWARYADWLFTTPLLLLDLALLV-------DADQGTILA
LVGADGIMIGTGLVGALTKV------YSYRFVWWAISTAAMLYILYVLFFGFTSKAES-MRPEVASTFKVLRNVTVVLW-SAYPV
VWLIGSEG-AGIV-PLNIETLLFMVLDVSAKVGFGLILLRSRAIFGEAEAPEPSAG-DGAAATSD----------------------------------
---------------------------</seq-data>
</sequence>
<sequence seq-type="Protein">
<seq-name>BAC3_HALSD</seq-name>
<seq-info><length>387</length></seq-info>
<seq-data>------------------------------MDPIALQAGYDLLGDGRPETLWLGIGTLLMLIGTFYFLVRGWG---VTDKDAREYY
AVTILVPGIASAAYLSMFFGIGLTEVTVGG----------------EMLDIYYARYADWLFTTPLLLLDLALLA-------KVDRVTIGTLVG
VDALMIVTGLIGALSHT------AIARYSWWLFSTICMIVVLYFLATSLRSAAKE-RGPEVASTFNTLTALVLVLW-TAYPILWIIG
TEG-AGVV-GLGIETLLFMVLDVTAKVGFGFILLRSRAILGDTEAPEPSAGADVSAAD--
-------------------</seq-data>
</sequence>
<sequence seq-type="Protein">
<seq-name>BAC1_HALS1</seq-name>

<seq-info><length>387</length></seq-info>
<seq-data>---------------------------MDPIALTAAVGADLLGDGRPETLWLGIGTLLMLIGTFYFIVKGWG---VTDKEAREYY
SITILVPGIASAAYLSMFFGIGLTEVQVGS----------------EMLDIYYARYADWLFTTPLLLLDLALLA-------KVDRVSIGTLVGV
DALMIVTGLVGALSHT------PLARYTWWLFSTICMIVVLYFLATSLRAAAKE-RGPEVASTFNTLTALVLVLW-TAYPILWIIG
TEG-AGVV-GLGIETLLFMVLDVTAKVGFGFILLRSRAILGDTEAPEPSAGAEASAAD---
-------------------</seq-data>
</sequence>
<sequence seq-type="Protein">
<seq-name>BAC2_HALS2</seq-name>
<seq-info><length>387</length></seq-info>
<seq-data>-----------------------------MDPIALQAGFDLLNDGRPETLWLGIGTLLMLIGTFYFIARGWG---VTDKEAREYYA
ITILVPGIASAAYLAMFFGIGVTEVELASG---------------TVLDIYYARYADWLFTTPLLLLDLALLA-------KVDRVTIGTLIGVDA
LMIVTGLIGALSKT------PLARYTWWLFSTIAFLFVLYYLLTSLRSAAAK-RSEEVRSTFNTLTALVAVLW-TAYPILWIVGTEG-
AGVV-GLGIETLAFMVLDVTAKVGFGFVLLRSRAILGETEAPEPSAGADASAAD--
--------------</seq-data>
</sequence>
<sequence seq-type="Protein">
<seq-name>BACR_HALHM</seq-name>
<seq-info><length>387</length></seq-info>
<seq-data>--GIGTLLMLIGTFYFIARGWG---VTDKKAREYYAITILVPGIASAA
YLSMFFGIGLTTVEVAGMA--------------EPLEIYYARYADWLFTTPLLLLDLALLA-------NADRTTIGTLIGVDALMIVTGLIGA
LSHT------PLARYTWWLFSTIAFLFVLYYLLTVLRSAAAE-LSEDVQTTFNTLTALVAVLW-TAYPILWIIGTEG-AGVV-GLGVE
TLAFMVLDVTA--</seq-data>
</sequence>
<sequence seq-type="Protein">
<seq-name>BACR_HALAR</seq-name>
<seq-info><length>387</length></seq-info>
<seq-data>---MPEPGSEAIWLWLGTAGMFLGMLYFIARGWG---ETDSRRQKFYIATIL
ITAIAFVNYLAMALGFGLTIVEFAG----------------EEHPIYWARYSDWLFTTPLLLYDLGLLA-------GADRNTITSLVSLDVLM
IGTGLVATLSPGSGVLSAGAERLVWWGISTAFLLVLLYFLFSSLSGRVAD-LPSDTRSTFKTLRNLVTVVW-LVYPVWWLIGT
EG-IGLV-GIGIETAGFMVIDLTAKVGFGIILLRSHGVLDGAAETTGTGATPADD---
---------------</seq-data>
</sequence>
<sequence seq-type="Protein">
<seq-name>BACR_HALHP</seq-name>
<seq-info><length>387</length></seq-info>
<seq-data>---IWLWLGTAGMFLGMLYFIARGWG---ETDSRRQKFYIATILITAIA
FVNYLAMALGFGLTIVEFAG----------------EEHPIYWARYSDWLFTTPLLLYDLGLLA-------GADRNTITSLVSLDVLMIGTG
LVATLSAGSGVLSAGAERLVWWGISTAFLLVLLYFLFSSLSGRVAD-LPSDTRSTFKTLRNLVTVVW-LVYPVWWLIGTEG-IG
LV-GIGIETAGFMVIDLTA--</seq-data
>
</sequence>

<sequence seq-type="Protein">
<seq-name>BACR_HALVA</seq-name>
<seq-info><length>387</length></seq-info>
<seq-data>--MPAPEGEAIWLWLGTAGMFLGMLYFIARGWG---ETDSRRQKFYIATIL
ITAIAFVNYLAMALGFGLTIVEIAG----------------EQRPIYWARYSDWLFTTPLLLYDLGLLA-------GADRNTISSLVSLDVLMI
GTGLVATLSAGSGVLSAGAERLVWWGISTAFLLVLLYFLFSSLSGRVAD-LPSDTRSTFKTLRNLVTVVW-LVYPVWWLVGT
EG-IGLV-GIGIETAGFMVIDLVAKVGFGIILLRSHGVLDGAAETTGAGATATAD---
---------------</seq-data>
</sequence>
<sequence seq-type="Protein">
<seq-name>BACR_HALHS</seq-name>
<seq-info><length>387</length></seq-info>
<seq-data>--LWLGTAGMFLGMLYFIARGWG---ETDGRRQKFYIATILITAIAF
VNYLAMALGFGLTFIEFGG----------------EQHPIYWARYTDWLFTTPLLLYDLGLLA-------GADRNTIYSLVSLDVLMIGTGV
VATLSAGSGVLSAGAERLVWWGISTAFLLVLLYFLFSSLSGRVAN-LPSDTRSTFKTLRNLVTVVW-LVYPVWWLVGSEG-L
GLV-GIGIETAGFMVIDLVA---</seq-dat
a>
</sequence>
<sequence seq-type="Protein">
<seq-name>BACR_HALSR</seq-name>
<seq-info><length>387</length></seq-info>
<seq-data>------------------------------------MLQSGMSTYVPGGESIFLWVGTAGMFLGMLYFIARGWS---VSDQRRQKFYI
ATIMIAAIAFVNYLSMALGFGVTTIELGG----------------EERAIYWARYTDWLFTTPLLLYDLALLA-------GADRNTIYSLVGL
DVLMIGTGALATLSAGSGVLPAGAERLVWWGISTGFLLVLLYFLFSNLTDRASE-LSGDLQSKFSTLRNLVLVLW-LVYPVLW
LVGTEG-LGLV-GLPIETAAFMVLDLTAKIGFGIILLQSHAVLDEGQTAS-EGAAVAD---
---------------------</seq-data>
</sequence>
<sequence seq-type="Protein">
<seq-name>O93740</seq-name>
<seq-info><length>387</length></seq-info>
<seq-data>---------------------------------MCCAALAPPMAATVGPESIWLWIGTIGMTLGTLYFVGRGRG---VRDRKMQEF
YIITIFITTIAAAMYFAMATGFGVTEVMVGD----------------EALTIYWARYADWLFTTPLLLLDLSLLA-------GANRNTIATLI
GLDVFMIGTGAIAALSST------PGTRIAWWAISTGALLALLYVLVGTLSENARN-RAPEVASLFGRLRNLVIALW-FLYPVVWI
LGTEGTFGIL-PLYWETAAFMVLDLSAKVGFGVILLQSRSVLERVATPTAAPT--
------------------</seq-data>
</sequence>
<sequence seq-type="Protein">
<seq-name>F123_CORVE</seq-name>
<seq-info><length>387</length></seq-info>
<seq-data>----------------------------MGNSALDLNPP-NATFHLSTHGSDWLWAAFSVFGVSLLTVVAWTF----TRPRGARL
FHQIAIVVLTTGSLAYFSMASDLGATPVPVEFRGE-------------GTRQIWFVRYIQWFITFPLLLLELLLAT-------GLSLSDIFTTL
FMSIVLVITGLVAALVPS-------TYKWGYYTFGVSALFYIWYVLLWHGPHTTFA-AGGVLRRGYILAAGYLSFLL-LLYPIAWAC

-AEG-GNVI-SVTSEMIWYGILDIFAGPIFLFFFLWELRGVDYATFGLHSGKYTDKSAYAPNTAQAAGTVPATTSTGAAGNV-
--------------------------------------</seq-data>
</sequence>
<sequence seq-type="Protein">
<seq-name>YRO2_YEAST</seq-name>
<seq-info><length>387</length></seq-info>
<seq-data>-----------------MSDYVELLKRGGNEAIKINPPTGADFHITSRGSDWLFTVFCVNLLFGVILVPLMF----RKPVKD
RFVYYTAIAPNLFMSIAYFTMASNLGWIPVRAKYNHVQTSTQ----KEHPGYRQIFYARYVGWFLAFPWPIIQMSLLG-------
GTPLWQIAFNVGMTEIFTVCWLIAACVHS-------TYKWGYYTIGIGAAIVVCISLMTTTFNLVKA-RGKDVSNVFITFMSVIM
FLWLIAYPTCFGI-TDG-GNVL-QPDSATIFYGIIDLLILSILPVLFMPLANYLGIERLGLIFDEEPAEHVGPVAEKKMPSPASFKS
SDSDSSIKEKLKLKKKHKKDKKKAKKAKKAKKAKKAQEEEEDVATDSE</seq-data>
</sequence>
<sequence seq-type="Protein">
<seq-name>YD33_YEAST</seq-name>
<seq-info><length>387</length></seq-info>
<seq-data>-----------------MSTFETLIKRGGNEAIKINPPTGADFHITSRGSDWFWTCFCCYLLFGLILTFLMF----RKPVNDR
FFYLTGIAPNFFMCIAYFTMASNLGWIPVKAKYNHVQTSTQ----KEHPGYRQIFYSRFVGWFLALPWPIIQICMLA-------GT
PFWQMAFNVCITEFFTVCWLIAACVHS-------TYKWGYYTIGLGAAIVVSISVMTTSYNLVKQ-RDNDIRLTFLVFFSIIMFL
WIIAYPTCFGI-TDG-GNVL-QPDSAGIFYGIIDLILMCFIPTLLVPIANHFGADKLGYHFGPSDAEAVMAPKAPVASPRPAAT
PNLSKDKKKKSKKSKKSKKSKKSEE------------------------</seq-data>
</sequence>
<sequence seq-type="Protein">
<seq-name>HS30_YEAST</seq-name>
<seq-info><length>387</length></seq-info>
<seq-data>------------------MNDTLSSFLNRNEALGLNPPHGLDMHITKRGSDWLWAVFAVFGFILLCYVVMFFIAENKGS
RLTRYALAPAFLITFFEFFAFFTYASDLGWTGVQAEFNHVKVSKSIT--GEVPGIRQIFYSKYIAWFLSWPCLLFLIELAASTTGE
NDDISALDMVHSLLIQIVGTLFWVVSLLVGS---LIKSTYKWGYYTIGAVAMLVTQGVICQRQFF-------NLKTRGFNALMLCT
CMVIVWLYFICWGL-SDG-GNRI-QPDGEAIFYGVLDLCVFAIYPCYLLIAVSRDGKLPRLSLTGGFSHHHATDDVEDAAPET
KEAVPESPRASGETAIHEPEPEAEQAVEDTA----------------------</seq-data>
</sequence>
<sequence seq-type="Protein">
<seq-name>O74870</seq-name>
<seq-info><length>387</length></seq-info>
<seq-data>-------------------------MNAKLSSSGMVLKELPEVALQKISSNYYWAVFAVFLLCAIVFPLVSIF---SLPQKQTYHR
FFSILS-LVSCLAYFTMACNYGLKNVFSSASFFREV----------SVRMVYYVRYIQWLINFPLIIVMLHWTV-------GVSILEIAYVV
CYVLFAIVCLLAAALTSS-------PYKWAYYGFSFVGYFIALAHSVVLHKKYASR-LETSARLGFLWSIVYLHVIW-FLYYACWIL-S
EG-LNVI-SPIGEAIFYSILDLFEFGFFGAAFSWMLDLVGIENFKSPQSIPLGACSPADDKFSMCPDMEAQNQADDLAVETRI
QISNLPSSPTKNNC-----------------------</seq-data>
</sequence>
<sequence seq-type="Protein">
<seq-name>Q9UW81</seq-name>
<seq-info><length>387</length></seq-info>

<seq-data>------MIHPEQVADMLRPTTSTTSSHVPGPVPTVVPTPTEYQTLGETGHRTLWVTFALMVLSSGIFALLSWN---
-VPTSKRLFHVITTLITVVASLSYFAMATGHATTFNCDTAWDHHKHVPD---TSHQVCRQVFWGRYVDWALTTPLLLLELCL
LA-------GVDGAHTLMAIVADVIMVLCGLFAALGEG----GNTAQKWGWYTIGCFSYLFVIWHVALHGSRTVTA-KGRGVSR
LFTGLAVFALLLW-TAYPIIWGI-AGG-ARRT-NVDTEILIYTVLDLLAKPVFGFWLLLSHRAMPETNIDLPGYWSHGLATEGRI
RIGEED--</seq-data>
</sequence>
<column-score>
<colsco-name>coreblock</colsco-name>
<colsco-owner>0</colsco-owner>
<colsco-type>int</colsco-type>
<colsco-data>0 0 0 0 0 0 0 0 0 0 0 0 0 0 0 0 0 0 0 0 0 0 0 0 0 0 0 0 0 0 0 0 0 0 0 0 0 0 0 0 0 0 0 0 0 0 0 0 0 0 0 0
0 0 0 0 0 0 0 0 0 0 0 0 0 0 0 0 0 0 0 0 0 0 0 0 0 0 0 1 1 1 1 1 1 1 1 1 1 1 1 1 1 1 1 1 1 1 1 1 1 1 1 1 1 1 1 1 1 1 0
0 0 0 0 0 0 0 0 0 0 0 0 0 0 0 0 0 0 0 0 0 0 0 0 1 1 1 1 1 1 1 1 1 1 1 1 1 1 1 1 1 1 1 1 1 1 1 1 0 0 0 0 0 0 0 0 0 0 1
1 1 1 1 1 1 1 1 1 1 1 1 1 1 1 1 1 1 1 1 1 1 1 1 1 0 0 0 0 0 0 0 0 0 0 1 1 1 1 1 1 1 1 1 1 1 1 1 1 1 1 1 1 1 1 0 0 0 0
0 0 0 0 0 0 0 0 0 0 0 1 1 1 1 1 1 1 1 1 1 1 1 1 1 1 0 0 0 1 1 1 1 1 1 1 0 1 1 1 0 0 0 0 0 0 0 0 0 0 1 1 1 1 1 1 1 1 1
1 1 1 1 1 1 1 1 1 1 1 1 1 0 0 0 0 0 0 0 0 0 0 0 0 0 0 0 0 0 0 0 0 0 0 0 0 0 0 0 0 0 0 0 0 0 0 0 0 0 0 0 0 0 0 0 0 0 0
0 0 0 0 0 0 0 0 0 0 0 0 0 0 0 0 0 0 0 0 0 0 0 0 0 0 0 0 0 0 0 0 0 0 0 0 0 0 0 0 </colsco-data>
</column-score>
</alignment>
</macsim>
:::::::::::::
ptga_core-block.xml
:::::::::::::
<?xml version="1.0"?>
<!DOCTYPE macsim SYSTEM "http://www-bio3d-igbmc.u-strasbg.fr/macsim.dtd">
<macsim>
<alignment>
<aln-name>1</aln-name>
<sequence seq-type="Protein">
<seq-name>Q57071</seq-name>
<seq-info><length>1088</length></seq-info>
<seq-data>--MWKKFFG
QLQRIGKALMLPVAILPAAGLLLALGNAFQGDALQSL----MPFIKAEGFQNVAKMMEGAGGIIFDN---LAIIFALGVAIGLAS
G-----DGVAAIAAFVGFIVL---NKTMGMFLGVTPEK-------------------AADAATGFANVLGIP-----TLQTGVFGGIIIGALAA
WCYNKFYNISLPSYLGFFAG---KRFVPIMMAT-CSFILAFPMAIIWPSIQGGLNAFSEGLL----ASNTGLAVFLFGFIKRLLIPFG
LHHIFHAPFWFEFGSYKNAA---GQIIHGDQRIFIEQIRDNVPL------------------
---TAGKFMQGEFPVMMFGL-PAAAL
AIYQTAKKENKKVVAGLMLSGALTSFLTGITEPLEFSFLFVAPLLFF-IHAVLDGLSFLILYLLD-----LHLGYTFS-GGFIDFFLLGIL
P----NKTQWWLVIPVGLVYAAIYYIIFRFLIVKFNFKTPGREDKEVKSSNVAAS-----------------------------ELPFKVLDAMGG
KANIKHLDACITRLRVEVNDKAKVDVQ-ELKDLGASGVLEVG-NNMQAIFGPKSDQIKHDMQQIMDGKITS----------PEET
TVTEEGDKETAEIAAAGGGVVYAPIKGEVVDISEVPDKVFSEKMMGDGIAIKPETGEVVAPFD-GVVK-MVFPTKHAIGLES
KDGIELLIHFGLETVKLEGKG--------FDILVKENDNIVLG-QPLMKVDLDYIKEHADSTITPIVVTNLNGRTMEVLQH---GEVK

QGDKVILVK---------------</seq-data>
</sequence>
<sequence seq-type="Protein">
<seq-name>Q53922</seq-name>
<seq-info><length>1088</length></seq-info>
<seq-data>--MKNLLKKFF
GQLQRIGKALMLPVAILPAAGILLTFGNAMHNEQILHF----APWMQHHYIQLISQIMEASGQVIFDN---LPLLFAMGTALG
LAGG-----DGVAGIAALVGYLIM---SATMGKIAGITIDDIFSYADGAKTL-------GQSAKDPAHALVLGIP-----TLQTGVFGGIII
GALAAWCYNKFYNIQLPQFLGFFAG---KRFVPIITSL-VAIVTGIVLSFVWPPVQDGLNNLSNFLL----GKNLALTTFIFGIIERS
LIPFGLHHIFYAPFWFEFGHYVNES---GNLVRGDQRIWMAQYQDGVPF-----
---TAGAFTTGKYPFMMFG
L-PAAAFAIYPQAKPERRKVVGGLMLSAALTSFLTGITEPLEFSFLFVAPILYV-AHVILAGTSFLIMHLLH-----VQIGMTFS-GG
FIDYILYGLLSW---DRSNALLVIPVGIAYALIYYFLFTFLIKKLNLKTPGREDKEVESKDVSVS----------------------------ELPFEV
LEAMGNKDNIKHLDACITRLRVEVRDKGLVDVE-KLKQLGASGVLEVG-NNMQAIFGPKSDQIKHDMQQIMDGKITS------
----PAETTVTEDGDVETAEIVAEGGAVIYAPITGEAVDLSEVPDKVFSAKMMGDGIAIKPETGEVVAPFD-GKVK-MIFPTKH
AIGLESKDGIELLIHFGLETVKLDGEG--------FEILVKENDNIVLG-QPLMKVDLNYIKEHADDTITPIIITNAGSANIEVLHT---G
KVEQGEKLLLVNN--------------</seq-data>
</sequence>
<sequence seq-type="Protein">
<seq-name>PTGA_BACSU</seq-name>
<seq-info><length>1088</length></seq-info>
<seq-data>---MFKALFG
VLQKIGRALMLPVAILPAAGILLAIGNAMQNKDMIQV----LHFLSNDNVQLVAGVMESAGQIVFDN---LPLLFAVGVAIGL
ANG-----DGVAGIAAIIGYLVM---NVSMSAVLLANGTIPSDSVERAKF---------FTENHPAYVNMLGIP-----TLATGVFGGIIV
GVLAALLFNRFYTIELPQYLGFFAG---KRFVPIVTSI-SALILGLIMLVIWPPIQHGLNAFSTGLV----EANPTLAAFIFGVIERSLI
PFGLHHIFYSPFWYEFFSYKSAA--GEIIRGDQRIFMAQIKDGVQL-------------
---TAGTFMTGKYPFMMFGL-PA
AALAIYHEAKPQNKKLVAGIMGSAALTSFLTGITEPLEFSFLFVAPVLFA-IHCLFAGLSFMVMQLLN-----VKIGMTFS-GGLI
DYFLFGILP----NRTAWWLVIPVGLGLAVIYYFGFRFAIRKFNLKTPGREDAAEETAAPGKTGEAG------------------------DLP
YEILQAMGDQENIKHLDACITRLRVTVNDQKKVDKD-RLKQLGASGVLEVG-NNIQAIFGPRSDGLKTQMQDIIAGRKPRP
EP---KTSAQEEVGQQVEEVIAEPLQNEIGEEVFVSPITGEIHPITDVPDQVFSGKMMGDGFAILPSEGIVVSPVR-GKIL-NVF
PTKHAIGLQSDGGREILIHFGIDTVSLKGEG--------FTSFVSEGDRVEPG-QKLLEVDLDAVKPNVPSLMTPIVFTNLAEGETV
SIKAS--GSVNREQEDIVKIEK-------------</seq-data>
</sequence>
<sequence seq-type="Protein">
<seq-name>YBFS_BACSU</seq-name>
<seq-info><length>1088</length></seq-info>
<seq-data>---MFKKAFQI
LQQLGRALMTPVAVLPAAGLLLRFGDKDLL---------------------NIPIIKDAGGVVFDN---LPLIFAVGVAIGLAGG-----EGVA
GLAAVIGYLIL---TVTLDNMG------------------------KLLGLQPPYEG-AEH-----LIDMGVFGGIIIGLLAAYLYKRFSSIELHPVL
GFFSG---KRFVPIITSV-SSLVIGVIFSFVWPLIQNGINAASSL------IADSTVGLFFYATIYRLLIPFGLHHIFYTPFYFMMGEYTD
PST---GNTVTGDLTRFFAGDP---

--TAGRFMMGDFPYMIFCL-PAVALAIIHTARPEKKKMISGVMIS
AALTSMLTGITEPVEFSFLFVAPVLYL-INSILAGVIFVVCDLFH-----VRHGYTFS-GGGIDYVLNYGL------STNGWVVIPVGIV
FAFIYYYLFRFAILKWNLKTPGRETDEDGQNEEKAPVAKD-------------------------QLAFHVLQALGGQQNIANLDACITRLR
VTVHQPSQVCKD-ELKRLGAVGVLEVN-NNFQAIFGTKSDALKDDIKTIMAGGV--------------PATAAALDTVTDKPLKPDS
DETFIYPIKGETVSLGDVPDQVFSEKMMGEGFAIIPSEGKVVAPAD-GEIV-SIFPTKHAIGFMSAGGTEILIHVGIDTVKLNGE
G--------FEAHVTSGQAVKQG-ELLLTFDLNYIKQHAASAITPVIFTNTSEEDLKHIQMK------------------------------</seq-da
ta>
</sequence>
<sequence seq-type="Protein">
<seq-name>PTGB_ECOLI</seq-name>
<seq-info><length>1088</length></seq-info>
<seq-data>--MFKNAFA
NLQKVGKSLMLPVSVLPIAGILLGVGSANFSW----------------LPAVVSHVMAEAGGSVFAN---MPLIFAIGVALGFTNN-----
DGVSALAAVVAYGIM---VKTMAVVA-------------------------PLVLHLPAEEIASKH-----LADTGVLGGIISGAIAAYMFNRFY
RIKLPEYLGFFAG---KRFVPIISGL-AAIFTGVVLSFIWPPIGSAIQTFSQWAA----YQNPVVAFGIYGFIERCLVPFGLHHIWNV
PFQMQIGEYTNAA---GQVFHGDIPRYMAGDP----------------------------------
---TAGK-LSGGFLFKMYGL-PAAAIAIWHSAKPE
NRAKVGGIMISAALTSFLTGITEPIEFSFMFVAPILYI-IHAILAGLAFPICILLG-----MRDGTSFS-HGLIDFIVLSGN------SSKL
WLFPIVGIGYAIVYYTIFRVLIKALDLKTPGREDATEDAKATGTS-----------------------------EMAPALVAAFGGKENITNLDA
CITRLRVSVADVSKVDQA-GLKKLGAAGVVVAG-SGVQAIFGTKSDNLKTEMDEYIRNH--
----
-----------------------------</seq-data>
</sequence>
<sequence seq-type="Protein">
<seq-name>PTGB_SALTY</seq-name>
<seq-info><length>1088</length></seq-info>
<seq-data>--MFKNAFA
NLQKVGKSLMLPVSVLPIAGILLGVGSANFSW----------------LPAVVSHVMAEAGGSVFAN---MPLIFAIGVALGFTNN-----
DGVSALAAVVAYGIM---VKTMAVVA-------------------------PLVLHLPAEEIAAKH-----LADTGVLGGIISGAIAAYMFNRFY
RIKLPEYLGFFAG---KRFVPIISGL-AAIFTGVVLSFVWPPIGTAIQAFSQWAA----YQNPVVAFGIYGFIERCLVPFGLHHIWN
VPSQMQIGEYTNAA---GQVFHGDIPRYMAGDP---------------------------------
---TAGM-LSGGFLFKMYGL-PAAAIAIWHSAK
PENRAKVGGIMISAALTSFLTGITEPIEFSFMFVAPILYI-IHAILAGLAFPICILLG-----MRDGTSFS-HGLIDFIVLSGN------SSK
LWLFPIVGAGYAIVYYTVFRVLIKAFDLKTPGREDTTDDAKAGATS-----------------------------EMAPALVAAFGGKENITNL
DACITRLRVSVADVAKVDQA-GLKKLGAAGVVVAG-SGVQAIFGTKSDNLKTEMDEYIRNH------------------------------------
----
----------------------------------</seq-data>
</sequence>
<sequence seq-type="Protein">
<seq-name>O32444</seq-name>
<seq-info><length>1088</length></seq-info>
<seq-data>--

--MLPIATLPVAALLLRLGQPDLL------------------------GIPFMAAAGDAIFSQ---LPLLFGLGIAIGLSKDG----SGAAGLAGAVAY
FVL---TATAKTID--A-----SINMSFFGGIFAGIIAGHSYNAFHATRLPEWLAFFAG---KRLVPI
MAGL-FALVAGALSGVIWPSIQGGLDALAHAV-----STSGALGQFVYGTLNRALIPVGLHHVLNSYFWFGMGTCQEILVSG
AQAAGQALPALEKLCVDPALAKTLVVGQEHTFAFKNAVTPEITAVVKEVT-EVVKSGDLHRFFGGDK------------------------------
--SAGVFMNGFFPVMMFGL-PGAALA
MYLAAPAERRHQVGGALFSVAFCSFLTGITEPLEFMFVFLAPALYA-MHAVFTGLSLVVANMFG-----TLHGFGFS-AGLIDF
LLNWGL------ATKPATLFVIGLAFFALYFFTFTFAIRAFNLKSPGREDEEVETETDSADASKG-----------------------------DLARQYL
KALGGHENLTSIDACITRLRLTLKDRSVADEA-VLKKLGAKGVVKLGENNLQVILGPLAEIVAGEMKAISPKEDLSDVKLP-----
----
--</seq-data>
</sequence>
<sequence seq-type="Protein">
<seq-name>O34521</seq-name>
<seq-info><length>1088</length></seq-info>
<seq-data>--MLSFL
QKLGKSFMLPIAVLPAVGIILALGREDVF------------------------NIPFVYQAGTAVFDH---LPLIFAIGIAIGISKDS----NGAAGLS
GAISYLML---DAATKTID--K-----TNNMAVFGGIIAGLIAGYTYNRFKDTKLPEYLGFFSG---
RRLVPILTAI-ITIILAGIFGVVWPPIQSCINSFGEWM-----LGLGGIGAGIFGLFNRLLIPLGLHHVLNNIFWFQFGEYN-----------
---GVTGDLARFFAKDP---
--TAGTYMTGFFPIMMFGL-PAACLAMVVTAKPSKRKATAGMMIGFALTA
FITGITEPIEFAFMFLSPLLYA-VHAVLTGLSLFIVNWLG-----IRSGFSFS-AGAIDYVLSYGI------AEKPLLLLLVGICYAAVYFIV
FYVLIKALNLKTPGREDDDVDEVLDENTVQD------------------------------VNENIMLKGLGGKENLQTIDHCATRLRLTVKDTAL
VDEA-LLKKAGAKGVVKSGGQSVQVIIGPNVEFAAEELRAAVK---

----</seq-data>
</sequence>
<sequence seq-type="Protein">
<seq-name>PTAA_ECOLI</seq-name>
<seq-info><length>1088</length></seq-info>
<seq-data>--MNILGFF
QRLGRALQLPIAVLPVAALLLRFGQPDLL------------------------NVAFIAQAGGAIFDN---LALIFAIGVASSWSKDS----AGAAA
LAGAVGYFVL---TKAMVTIN--P-----EINMGVLAGIITGLVGGAAYNRWSDIKLPDFLSFFG
G---KRFVPIATGF-FCLVLAAIFGYVWPPVQHAIHAGGEWI-----VSAGALGSGIFGFINRLLIPTGLHQVLNTIAWFQIGEFT
NAA---GTVFHGDINRFYAGDG---
---TAGMFMSGFFPIMMFGL-PGAALAMYFAAPKERRPMVGG
MLLSVAVTAFLTGVTEPLEFLFMFLAPLLYL-LHALLTGISLFVATLLG-----IHAGFSFS-AGAIDYALMYNLP---AASQNVWM
LLVMGVIFFAIYFVVFSLVIRMFNLKTPGREDKEDEIVTEEANSNTEEGLT------------------------QLATNYIAAVGGTDNLKAID
ACITRLRLTVADSARVNDT-MCKRLGASGVVKLNKQTIQVIVGAKAESIGDAMKKVVARGPVAAASAEAT------PATAAPV
AKPQAVPNAVSIAELVSPITGDVVALDQVPDEAFASKAVGDGVAVKPTDKIVVSPAA-GTIV-KIFNTNHAFCLETEKGAEIV
VHMGIDTVALEGKG--------FKRLVEEGAQVSAG-QPILEMDLDYLNANARSMISPVVCSNIDDFSGLIIKAQ--GHIVAGQTP
LYEIKK---------------</seq-data>
</sequence>

<sequence seq-type="Protein">
<seq-name>PTAA_KLEPN</seq-name>
<seq-info><length>1088</length></seq-info>
<seq-data>--MNILGFF
QRLGRALQLPIAVLPVAALLLRFGQPDLL---------------------NVPFIAQAGGAIFDN---LALIFAIGVASSWSKDN----AGSAA
LAGAVGYFVM---TKAMVTIN---P-----EINMGVLAGIITGLVAGAVYNRWAGIKLPDFLSFF
GG---KRFVPIATGF-FCLILAAIFGYVWPPVQHAIHSGGEWI-----VSAGALGSGIFGFINRLLIPTGLHQVLNTIAWFQIGEFT
NAA---GTVFHGDINRFYAGDG--
---TAGMFMSGFFPIMMFGL-PGAALAMYLAAPKARRPMVGG
MLLSVAITAFLTGVTEPLEFLFLFLAPLLYL-LHAVLTGISLFIATALG-----IHAGFSFS-AGAIDYVLMYSLP---AASKNVWMLL
VMGVVFFFVYFLLFSAVIRMFNLKTPGREDKAADVVTEEANSNTEEGLT----------------------QLATSYIAAVGGTDNLKAID
ACITRLRLTVGDSAKVNDA-ACKRLGASGVVKLNKQTIQVIVGAKAESIGDEMKKVVTRGPVAAAAAAPA----GNVATAAP
AAKPQAVANAKTVESLVSPITGDVVALEQVPDEAFASKAVGDGIAVKPTDNIVVAPAA-GTVV-KIFNTNHAFCLETNNGA
EIVVHMGIDTVALEGKG--------FKRLVEEGTDVKAG-EPILEMDLDFLNANARSMISPVVCSNSDDYSALVILAS--GKVVAG
QTPLYEIKGK------------</seq-data>
</sequence>
<sequence seq-type="Protein">
<seq-name>O51000</seq-name>
<seq-info><length>1088</length></seq-info>
<seq-data>---MSTSSASISIFTI
LQKIGKAFMLPIALLPAAGILLGIGGAFTNETMIQAYGLEEVLGKGTVANSILYLMKYTGEVIFAN---LPLMFAAAIPIGLAKV
E----KGTAALAGVVGFLVM---HQTINGILYIQGITPESASLKALLELGMPETA-ATAKSQEYTSVLGIF-----SLQMSVMGGLIA
GFVAVFLHNRFHNIQLPTFLAFFGG---TRFVPIVTTM-AMFVVGIFLTFIWPFIQGAMTSFGRIV-----EQSGLFGTFAYGAIK
RSLIPFGLHHIFYLPFWQTAVGGTLEIN--NELISGAQNIFFKQLADPNTVHFE
VA---KGTRFFSGEFVVMIF
GL-PGAALAMYHTSKPENKKNVASLLLSASFTSMLTGITEPLEFAFLFAAPALYYFIYVPLFGLAYLLTHLLN-----VGVGLTFS-
GGFIDMFLFGILQGN--SKTNWIAIPILGIFYFIGFYFIFKFAIMKFNLKTIGREDEEMEKTMMSSEKTSLS--------------------------
ETASKVLEGLGGKDNITYIDACASRLRVNLKQIEVIKSDAYFKNLGASGILKKG-NSVQIVFGGLSDNIKMEIDKLM-------------
----
--</seq-data>
</sequence>
<sequence seq-type="Protein">
<seq-name>O51590</seq-name>
<seq-info><length>1088</length></seq-info>
<seq-data>---MLKGFEQ
AQKFGRSFMLPIAILPAAGLLLGIGGSLSNPETVRT----YSFLNIFFLQSVFKIMSASGSIIFSN---LAPIFSIGIAVGLAKSD----K
GTSGIAAFIGYLVM---NATIGVLIDVSGRA--------------------ESFSSGAVGFVLGIK-----TLETGVFGGVVVGILTYYLHSRFNK
VDLPKVLGFFSG---SRFVPIIVSF-SSIFLAVIMFLVWPFVQSGINKVGGLV-----DSTGYIGTLIYGIFLRMLGPFGLHHIFYLPF
WTTGLGGSVIID--GKLIEGTQNIFFAELAAQGTDRFFI-----------------------------
---GTSRFMSGRFITMMFGL-PGAALALYYTAKRE
ERTKVFGLLMSSALTSFLTGITEPLEFSFLFVAPILYV-VHATFDGFAFMLAHILQ-----ITIGQTFS-GGFVDFILFGILQGN--SRT
NWLLVPVIGIVWFFLYYFTFIFLINKFDFKTPGRTQDLNSEDSPSSKSSEFEE------------------------NYATKVIIGLGGASNIVEL

DCCATRLRITVRDVLKVSEK-ILKKTGSKGVIIKG-NGVQVVYGPGVSVLKNEIEELLEA---
----
---------------------------</seq-data>
</sequence>
<sequence seq-type="Protein">
<seq-name>O51143</seq-name>
<seq-info><length>1088</length></seq-info>
<seq-data>---MLKLALERLSMINFIKII
NFSSLQKFSKALRVPISILPVSCLLIGIGSVFSNSSS-------IVYVDKIFIQNVLGLMKAIGNAILLN---MPLIFSIGISIGVARMG---
-QGTAALGGLIGYLTF---NITENYFIEAFSGLVEA----------------ETMSSVGRINFFGVQ-----TLNTGIAGSLAVGLLVGYLHNK
FYNMKLPKPFVFFSE---CHFVPIVIILPFCVFLAIFFCLIWSSFDDLIASLGLFV-----FRFEYFGSFLYGFLNRLLLPLGLHSILSFPF
EFTSLGGVEIVN---GDTVRGLKNIFYAQLLDPSLGKFSS---------------------------
--GFAKISSGFYLSIMFGL-PGAALGVYKGIVHED
KNKVAALLFSGALTAFLTGITEPLEFLFIFTAPLLYF-VHAAYSGFALLLANFFN-----VTIGNSFS-TGFLDFFMFGILQGN--SKT
NWISVLPLGAMFFALYYFTFSWLYRYFDFQIFVTDDPFFEGQEGKLESL---------------------------GIAHLLIQGLGGFDNITKL
DVCSTRLHVDVVNTELVDNN-LLKEAGVLKIGLVN-GKVQLFYGSNVYYIKNAIDTYSPKSLFEASVMVAVDNVKKGFKTYIE
MKEDKKLEKQGKSGKTYKLSELEED---
---</seq-data>
</sequence>
<sequence seq-type="Protein">
<seq-name>PTOA_ECOLI</seq-name>
<seq-info><length>1088</length></seq-info>
<seq-data>---MTAKTAPKVTL
WEFFQQLGKTFMLPVALLSFCGIMLGIGSSLSSHDVITL----IPVLGNPVLQAIFTWMSKIGSFAFSF---LPVMFCIAIPLGLAR
EN----KGVAAFAGFIGYAVM---NLAVNFWLTNKGILPTTDA--------------AVLKANNIQSILGIQ-----SIDTGILGAVIAGIIV
WMLHERFHNIRLPDALAFFGG---TRFVPIISSL-VMGLVGLVIPLVWPIFAMGISGLGHMI-----NSAGDFGPMLFGTGERLL
LPFGLHHILVALIRFTDAGGTQEVC---GQTVSGALTIFQAQLSCPTTHGFSES--
--ATRFLSQGKMPAFLGGL-
PGAALAMYHCARPENRHKIKGLLISGLIACVVGGTTEPLEFLFLFVAPVLYV-IHALLTGLGFTVMSVLG-----VTIGNTD--GNI
IDFVVFGILHG---LSTKWYMVPVVAAIWFVVYYVIFRFAITRFNLKTPGRDSEVASSIEKAVAGAPGKSG----------------------
YNVPAILEALGGADNIVSLDNCITRLRLSVKDMSLVNVQ-ALKDNRAIGVVQLNQHNLQVVIGPQVQSVKDEMAGLMHT
VQA--
---</seq-data>
</sequence>
<sequence seq-type="Protein">
<seq-name>P96159</seq-name>
<seq-info><length>1088</length></seq-info>
<seq-data>---MSKSNKSTIW
EFFESLGKTFMLPVALLAFSGILLGVGSSFSSSAIKEA----IPFLDNAPFEYLFMWMTKIGLVAFIY---LPVMFAVAIPLGLAREE
----KGVAGFAGFVGYAAF---NLAINFFLTVAGVLDSQ----------------SERVAYGVKNIIGIE-----SIDTGILGAVVVGVIVAKLH
QRFYTFKMPDALAFFGG---ARFVPIISAL-VLGVVGVFIPFVWPYFAAGINGIGHVI-----ESAGDFGPMLFGTGERLLLPIGLH
HILVALIRFTEAGGTMDVC---GQTVSGALNIFYSELSCADTTTFTPE-------------

--VTAFLSQGKMPTFLGGL-PGAALA
MYHCARPENRSKIKALLISGVVACIVGGITEPLEFLFLFVAPVLYV-VHAILTGLGFMVMGLLD-----VTIGNTD--GNLIDFLVF
GVLQG---TATKWYLVPLVAAIWFVVYYSVFKFAITKFNLKTPGREVDTAEVDKELEAAFINES----------------------GKGAAV
LAALGGKENITSLDNCITRLRLSVNDMSLVNDA-KLKAHGALGVVKLDQHNLQVVIGTQVHLVKNEMQAIMSAS-----------
----
--</seq-data>
</sequence>
<sequence seq-type="Protein">
<seq-name>Q9RL52</seq-name>
<seq-info><length>1088</length></seq-info>
<seq-data>---MQKTKAAL
WEFLQGLGKTFMLPVALLAFCGIMLGIGSSLSSDAVTDN----VAFLKGEGFHLVFTWMANTGLVAFTF---LPVLFAMAIPLG
LARED----KGVAAFSGFVGFVAM---NLAVNFYLTAKGVDFEDE--------------KAIEHYGIADVLGVQ-----SIDTGLLGAVAV
GIVVSLLHRRFRTQRMPDALAFFGG---LRFVPIVSAL-VLSVLGLLIPLVWPTFNGWISAVGRGI-----GHTGVFGPFFFGMG
EVLLRPIGLHHILVAMFRFTDVGGSGTVC---GDHVSGALNLFYAQLDCSAAPG
SAVTD---ATHFLSQGKMAA
YLGGL-PGAALAMYHCARRRMRPEIKALLVSGVVACVVGGITEPLEFLFLFVAPWLYL-VHAVLVGLGFLTAAVLG-----VFIG
NTD--GNVIDWLVFGVLQG---STTKWYLVPVIAAVWFAAYYFLFRWAITRFDLKTPGREDPPADDDHDEHDEHDGAHDA
EHAEEQVRAAPEPARELVAGKYDAAAMLDAIGGAGNIRSLDNCITRLRMTVADAEAVDEA-RLKKLGAVGVVKLDGHNV
QVVIGPQVQSVKDAIATMVPAGRP---
---</seq-data>
</sequence>
<sequence seq-type="Protein">
<seq-name>O07656</seq-name>
<seq-info><length>1088</length></seq-info>
<seq-data>---MKQKKAW
SFFQSLGKAFMYPIALLSVCGMMLGLGSGLASDDMAKL----IPFLAIPIIKTILDFIVSLGLFAFVN---LPVLFAIAIPLGLLKDKE
D--KAYGAFSGLIGFMAM---HLGTNFYLKQHDLLVVA----------------DQMSTHGQTIILGIQ-----SYNTSVLGGIVAGLLVAS
MYKKIVNLRIPESLGSYSG---PRLVPIITLI-VMSGFGLIIPFIWPPFFNLFMLIGHWI-----STSGPVGYFFYAVAERVTIPFGLN
HLVTSVFRFTPIGGSAVIG---GEEYYGTLNMFMAYVKENAVIPLD--------------
---LAGKMEQGKLMIQY-GL-AGAALA
MYRTAHAQNRKAIKALLISGVLTVIIGGVSEPIEFLFLFVSPLLFV-FHAFMNGFANMVLPYMG-----VKMG--FT-GDLIQFIS
FGVLRG---TRTGWPIAVCVEVAYFFIYYFVFRWTILKFNLMTVGREESSPVTLNAHEDTAIADIPTPDKSEL---------------QA
AEQMVKALGGKENIKSLDNCVTRLRLTIADMGLLDEA-AIKRAGGIAVVKLDQNTLQVIIGTKVIALRRDMDNYMGIY------
----
---</seq-data>
</sequence>
<sequence seq-type="Protein">
<seq-name>PTGA_MYCGE</seq-name>
<seq-info><length>1088</length></seq-info>
<seq-data>---MQISLVK------------IRNKFKQRN
RGSFRQWVGKLSNGLMIPIAVLPLAGIFLGIGDAISSNSSGIV-----------GVKFFGEFIKQGGNVVFAN---LPILFAVAIAITFS

QD-----AGVAGFSAFVFWATM---NAFMSSLIIPVDANNTA--------SGYNILYWKAVPQSAIASTLGLN-----SLSTSVFGGIIV
GALTAYLYNKFYAIRLPDVIGFFSG---TRFVPIICMT-IAIPVALLLLMVWPGVSILLNLIGTGLGILG-GRGYGANSLIFGYIERA
LIPFGVHHAFYAPLWYTSAGGSLQEIANQQVWIRAPG---SDYVTRVIGWEDFNTPGKWVIPAALANGTSGMMNGATTT
GQDSTSALSKYMSKESTNFLSWKELVDGLTRKGNFDELAKNGLLDGSNKIWIGLNQSGILGKKVLLSDGKDYTITFKTFANT
TPTFWSHGAHALLPISG------TPSAIT-NGVTVNGTANSKTYNVSQFTVAVPSLNPAQYSQGKFPFMLIGI-PAAGLAMILAA
PKGRRKEASSIIGSAAFTSFLTGITEPFEFTFLFLAPWLFYGIHAVLAAVSFWLMNLLS-----ANVGQTFS-GSFIDFILYGALPD
GRGWLANSYLVPIIGIFLALIYFPTFYFLTIRFNLATPGRGGKLITKKEYLAAKAAQKTDQTTNT--NFNQTQ---------IEAGMLL
RAYGGSENIAELGACITKLRVTVKNPELVNET-IIKDLGAAGVMRTTPTFFVAVFGTRAAVYKSAMQDIIQGKVNWTELQKV
LDKNDSTVEKPEIKPTP--VLKVQDEIVILSPVNGTLKPLTQVPDDTFKNRLVGDGIAILPSDGHFKAPGDVGVKTELAFPTGH
AFIFDV-DGVKVMLHIGIDTVKINADKKPGEQLEVFDVKTKQGEYTKLKSESVVEVDLKKLKRKYDPIT-PFIVMQESLDNFKL
VPIRQRGEIKVGQPLFKLIYKDKKS---------</seq-data>
</sequence>
<sequence seq-type="Protein">
<seq-name>PTGA_MYCPN</seq-name>
<seq-info><length>1088</length></seq-info>
<seq-data>--MQIKAQDTGQQKKSCLLSNIRNK
WKNRNRGSFRQWVGKLSNGLMIPIAVLPIAGIFLGVGDAIAGNAGDLT-----------GLRYFGLFIKNGGDVVFAN---LPILFAI
AIAITFSQD-----AGVAGFSAFVFWAAM---NGFMSSLILPFDKAGKIITDTSTPIAGFKVLYNKSVPVHAIATTLGLR-----TLST
SVFGGIIVGALTSVLYKKFYAIRLPDVIGFFSG---TRFVPIICFV-VAIPVALILLMIWPAVSIGLNAIGTGLGFLG-GKGYGANSLI
FGYIERSLIPFGVHHAFYAPLWYTSAGGSLQEIVNQQVWIRPDFHLSDNYVARVIGWVDPNNSSMYIIPGAL-NGQNGSST
GNTM-SKDLNGALSAYMSKESTAFLTWKDLVDGLTFKGNFDKMAENGLLDGSNKIWLGLNGSGILGKKLLLSDGNVYTITF
KTFANTTPIAWSKGAQAVLPLNASSTIVNNPTALAAATQSNNNTNNIKLYPVNSFRVAVESLNPAQYSQGKFPFMLFGI-P
AAGVAMILAAPKDRRKEAASIVGSAAFTSFLTGITEPFEFTFLFLAPWLFYGVHAVLAAVSFWLMNILG-----ANVGQTFS-G
SFIDFILYGALPDGRRWLANSYLVPIIGLFLAAIYFPTFYFLIKHFNLATPGRGGKLITKKEYLASKAAAKAEGVSGVAENFTQT
Q---------IEAGILLQAYGGKENIVELGACITKLRVTVKNPELVKEE-PIKELGAAGVMRTTPTFFVAVFGTRAAVYKSAMQDII
QGKVNWEALQKVINTDQLAVEPKETTPPKEVMPVVQDEIVILSPVNGTLKSLNQVPDETFKQKLVGEGVAIVPSDGHFKA
PGEAGVKTELAFPGGHAYIFDI-DGIKVMLHIGIDTVQINAKKQPGEPLEVFDIKTKQGEYTKEKSESVVEVDLKKLSKKYNPI
T-PFVVMKESLENFKLVPIRQRGEIKVGQPIFKLVYKKSQA---------</seq-data>
</sequence>
<sequence seq-type="Protein">
<seq-name>PTIB_BACSU</seq-name>
<seq-info><length>1088</length></seq-info>
<seq-data>---MMQKI
QRFGSAMFVPVLLFAFAGIIVGISTLFKNKTLMGP-----LADPDGFWYQCWYIIEQGGWTVFNQ---MPLLFAIGIPVALAKK
A----QARACLEALTVYLTF---NYFVSAILTVWGGAFGVDMNQ------------EVGGTSGLTMIAGIK-----TLDTNIIGAIFISSIVV
FLHNRYFDKKLPDFLGIFQG---STYIVMISFF-IMIPIALAVSYIWPMVQSGIGSLQSFL-----VASGAVGVWIYTFLERILIPTGL
HHFIYTPFIYGPAVAE---GGIVTYWAQHLGEYSQSAKPLKEL----------------
---FPQGGFALHGNSKIFGI-PGIALAFYV
TAKKEKKKLVAGLLIPVTLTAIVAGITEPIEFTFLFISPFLFA-VHAVLAATMSTVMYMAG-----VV-GNMG--GGLIEAVTLNWI
PLFGSHGMTYVYQILIGLSFTAIYFFVFRFLILKFNIATPGREKDEQQETKLYSKKEYRERKNKDETASAAETAD---------DTAFL
YIEALGGKDNITEVTNCATRLRVSVKDETKVEPDSVFRALGAHGVVRNG-KAFQVIIGLSVPQMRERVEKILNQ-------------
----

---</seq-data>
</sequence>
<sequence seq-type="Protein">
<seq-name>Q9S2H4</seq-name>
<seq-info><length>1088</length></seq-info>
<seq-data>--MSTATDTAAPAKKRG
SGLFQGLQKVGRSLQLPIAVLPAAGIMVRLGQDDIFGKDGL------------GWDKVAAVFNNAGGALTGS---LPILFCIGVAIG
FAKKA----DGSTALAAVVGFLVY---SKVLEAFPVTE---------------------------AVVQDGADVAAT-----YNDPGVLGGIIMGLLA
AVLWQRYHRKKLVDWLGFFNG---RRLVPIIMAF-VGIVVGVFFGLVWEPIGDGISNFGEWM-----TGLGSGGAALFGGVN
RALIPVGMHQFVNTVAWFQLGDFTNSA--GDVVHGDITRFLAGDP---------
---SAGIFQAGFFPIMMFG
L-PAAALAMAHTARPERRKAVLGMMISLAATSFVTGVTEPIEFSFMFIAPVLYV-LHAVLTAISMAITWGLG-----VHAGFNF
S-AGFIDYALNWHL------ATKPWLIIPIGLVFAAIYYVTFRFAIVKFNLKTPGREPEEEVEDLTKA-------------------------------------
----

---</seq-data>
</sequence>
<sequence seq-type="Protein">
<seq-name>Q9S2H5</seq-name>
<seq-info><length>1088</length></seq-info>
<seq-data>--MSADSAVSPARAR
WNTAFQGLQKMGRSLQLPIAVLPAAGILNRLGQPDVFGDDGL------------GWTNVSKVMTGAGGALLDGSLGLPLLFCV
GVAIGMAKKA----DGSTALAAVAGFLVY---YNVLRQFPEDCPEGSKVVPQIGCQ-----------VTDGSGEVTAFV-----FQNPG
VFGGIVMGLLTAYFWQRFHRTRLVDWLGFFNG---RRLVPIIMAF-VAIVFAALCLWVWPPIGDALESFSDWL-----NGIGA
WGAGVFGLANRALLVIGLHQFLNVPMWFQFGSYTKPD--GTVVHGDINM
FLAGDP--DAGQF
TTGFFPIMMFAL-PAAALAITHCAKPHRRKEVGGLMLSVGLTSFVTGITEPLEYSFLFIAPLLYA-AHAVLTGVSMAVTWALG
-----VKDGFSFS-AGLIDYVINWNL------ATKPWLIIPIGLCFAVVYYVVFRFAITKFDLKTPGREPEEDVEDVTKV-----------------
----

----------------------</seq-data>
</sequence>
<sequence seq-type="Protein">
<seq-name>q46072</seq-name>
<seq-info><length>1088</length></seq-info>
<seq-data>--MASKLTTTSQHILENLGGPDNITSMTHCATRLRFQVKDQSIVDQQEIDSDPSVLGVVPQGSTGMQVVMG-
GSVANYYQEILKLDGMKHFADGEATESSSKKEYGGVR---GKYSWIDYAFEFLSDTFRPILWALLGASLIITLLVLADTFGLQDF
RA------PMDEQPDTYVFLHSMWRSVFYF---LPIMVGATAARKL--------GANEWIGAAIPAALL---TPEFL----------------------
-------ALGSAGDTVTVFGLP--MVLNDYSGQVFPPLIAAIGLYWVEKGLKKIIPEAVQ---------MVFVPFFS-LLIMIPATAFLLG
PFGIGVGNGISNLLEAIN-NFSPFILSIVIPLLYPFLVPLGLHWPLNAIMIQNINT---

----LGYDFIQGPMGAWNFAC-FGLVTGVFLLSIKERNKAMRQVSLGGMLAGLLGGISEPSLYGVLLRFKKTYF-RLLPGCLAG

GIVMGIF------DIKAYAFVFTSLLTIPAM-----------DPWLGYTIGIAVAFFVSMFLVLALDYRSNEERDEARAKVAADKQAEE
DLKAEANATPAAPVAAA--GAGAGA
GAGAAAGAATAVAAKPKLAAGEVVDIVSPLEGKAIPLSEVPDPIFAAGKLGPGIAIQPTGNTVVAPAD-ATVI-LVQKSGHA
VALRLDSGVEILVHVGLDTVQLGGEG--------FTVHVERRQQVKAG-DPLITFDADFIRSKDLPLITPVVVSNA--AKFGEIEGIP
ADQANSSTTVIKVNGKNE-----------</seq-data>
</sequence>
<sequence seq-type="Protein">
<seq-name>ptga_brela</seq-name>
<seq-info><length>1088</length></seq-info>
<seq-data>--MASKLTTTSQHILENLGGPDNITSMTHCATRLRFQVKDQSIVDQQEIDSDPSVLGVVPQGSTGMQVVMG-
GSVANYYQEILKLDGMKHFADGEATESSSKKEYGGVR---GKYSGIDYAFEFLSDTFRPILWALLGASLIITLLVLADTFGLQDF
RA------PMDEQPDTYVFLHSMWRSVFYF---LPIMVGATAARKL--------GANEWIGAAIPAALL---TPEFL--------------------
-------ALGSAGDTVTVFGLP--MVLNDYSGQVFPPLIAAIGLYWVEKALKKIIPEAVQ---------MVFVPFFS-LLIMIPATAFLLG
PFGIGVGNGISSLLEAVN-NFSPFILSIVIPLLYPFLVPLGLHWPLNAIMIQNLNT---

-----LGYDFIQGPMGAWNFAC-FGLVTGVFLIALKEKNRAMRQVSLGGMLAGLLGGISEPSLYGVLLRFKKTYF-RLLPGCLV
GGIVMGIF------DIKAYAFVFTSLLTIPAM-----------DPWLGYTVGIAAAFFTSMLLVLFFDYRSDAERDEAKAQMAAAEQT
NN--------TPAAPAAPV--APAAGAAA
AGGAAGATAVATKPRLAAGQLVEITSPLEGHAVPLSEVPDPIFAAGKLGPGIAIEPTGNTVVAPAD-ATVI-LVQKSGHAVAL
RLESGVELLIHIGLDTVQLGGEG--------FKVHVERKQQVKAG-DPLITFDPEFIRSKNLPLITPVVVSNA--NKFGEIVGIEAAQ
ADATTTVIKVNGAE------------</seq-data>
</sequence>
<sequence seq-type="Protein">
<seq-name>o68468</seq-name>
<seq-info><length>1088</length></seq-info>
<seq-data>-MSKTEMRTAAEEILAGIGGADNIASFTHCATRLRFELNDAFKADKDKLDAIPKVMGAVPQGGRNYQVVIG-
GDVASVYNEMVQLPGVRKSDADVKAASRAKTQ--------GKMPWLDTAFEYLSDSFRPILGVLLGASLIIAFTAVMEAFGVV
DTRA--------DDKAAIWFFIDAMWRSVFYF---LPVMVAYNAGNKL--------RIDPWVPAAVMLALF---TPEFLGLKENPVAN
CVVDE--------------ALGTETCSIDLLGIN--VALPDYGGNVFVPLIMALVAAGIYKGFQKIIPSAVH---------MVFVPFLT-LLFTI
PITAVLIGPFGVWLGSTIGVGLAWMN-GNAPFVFAILIPMLYPFLVPLGLHWPLNALMLVNIEA------------------------------
----
------------------------LGYDFIQGPMGAWNFAC-FGATAAVLAISMREKDPEMRQTSGSALAAGLFGGISEPSLYGIHLRFKRI
YP-RMLVGCFAGGLTIAILGTASG-GVTTNAFVFTSLLTIPVF-----------SPMLTYTIAVAVAFAVAFLLIYFTDYRTAEEKEASR
ERAAEA---
GLVPSGTNPETEASTTTETADLVSPVDGMAVKLENIDDKVFASGTLGNGIGIVPVNGNIQSPVA-GTIA-TVTKTGHAFGIKT
DDGVEVLVHIGINTVRMKGEG--------FEPKVAKGDVVSIG-TPLAEVDLDKIKEAGYDNTVVVTVTNT--KAMGEVASIAEG
AVKAGEAVVAIKR--------------</seq-data>
</sequence>
<sequence seq-type="Protein">
<seq-name>q9x565</seq-name>
<seq-info><length>1088</length></seq-info>
<seq-data>---MDKYHDFNKHIIELVGGKENIEAVVHCMTRLRFTLIDKTKAQTNKLKNLDGVIDVVSNNV-AYQIIIGT-HV

NDVYKELMTQLELSPKTTLEEQS--------------GKKNPIKAALDVISESMTPILEPIICAGLLAAVLSIASLTGIISP------------DSS
TYHIFDALRSAVFYF---LPILMAMSCAKRL--------GASPYLAVALAATIL----SSSINDVK-------------------------------GLELFGI
G--LPQITYSNSFIPILLAIWFMGYVTKVVKRVIPNTLQ---------YFLTPLLV-MLITLPATLLIFGPLGFYMGEGIIGFFNLLMKYV
GSWFVMMLYSALQPFIIMLGAGNFIMPVVASLIAA---
--NGYDPAFISSCTISDI
AVGGAMLGYFLRT---RNLKQKQLFGTVTLSAVL-GVTEPAIYGVFVKYRRPFV-AVMIGGGLGGLFAGLT------SVKAYSVA
W-GLFGLPAYIGE-----GDFMNFWFMLAAVVISFAGSAVAAYLLGVPAQEEDQETINHIIAEKGKDKN-----------------------
--LRTSSLGNVVEGEVI
SLNEVKDQAFSTGALGKGIAIKPIDTHLYSPVD-GEIT-VVFPTNHAIGIQSQDGIEILIHIGIDTVELEGK--------YFNLNIKQGD
KIQRG-QLLGTVDFDKIQEAGYDTSTIVVVTNT--DSYLDVIPSN--SEQVIKTDNLLNVIL-----------</seq-data>
</sequence>
<sequence seq-type="Protein">
<seq-name>Q9RLU2</seq-name>
<seq-info><length>1088</length></seq-info>
<seq-data>---MGKYQALSKFILENVGGKENVLSVTHCMTRLRFTLKDDSKVNEDILKKSSEIMTAQFRGG-RFQVVIGT-HV
SDVYNEIQGVTGADKIAEAE-----------------VKKTGLNRLIEIITKVITPVLGILTASGLLQGILALLTITNVIDV------------NDGG
YIVLHAMGQAVFYF---LPVVLGYTSAKAF--------NLNPYVGMMLGATLV---IPELMTNLVTDKALYT------------LFDGTMF
STPVYN-TFFGIPILFPPNGYQYTVIPVIFITFLGAKVEKILKKIVPPLLA---------HNLNSFLT-ILITFPLAILIIGPVTNVLSSLISAG
VSGLY-EVSPVITSVVVGLLYQPLVIFGLHWPMSAIGINNLAQ--
--YGVDYIFP
MSFTASFAQ-TAVVLAVYLKT---RSRNQKALAIPAMISGMF-CIIEPAIYGFSLPVKKRFA-FSMIGGAVGSLIISIF------SVKM
YAFAF-GVLGFVSFINP----ANSSLGGLYVAIFATIVTMAVSFSLTFFTFKSSEDVVDEETKPLIKQQEVLA-----------------------
---PIAGTMQPISS
SADETFASKTMGEGVVIFPTGSKVVAPAA-GVVT-TVFPGGHALGITTENGTEILIHIGIDTVELNGE--------GFDVQVEVGS
KVKQG-QTLVNVDFEAIENKGYKSEVLVVILNT--DDFLDIISEEKTTVKQ-GG-KMLTLIPFNGKATNDMEVSY</seq-data>
</sequence>
<sequence seq-type="Protein">
<seq-name>PTBA_ERWCH</seq-name>
<seq-info><length>1088</length></seq-info>
<seq-data>----MNYETLASEIRDGVGGQENIISVIHCATRLRFKLRDNTNANADALKNNPGIIMVVESGG-QFQVVVGN-Q
VADVYQALLSLDGMARFSDSAAPEEE------------KKNSLFSGFIDIISSIFTPFVGVMAATGILKGFLALGVATHVISE-----------
-SSGTYKLLFAASDALFYF---FPIVLGYTAGKKF--------GGNPFTTLVIGATLV---HPSMIAAFNAMQAP----------------------
DHSTLHFLGIP--ITFINYSSSVIPILFASWVSCKLEKPLNRWLHANIR---------NFFTPLLC-IVISVPLTFLLIGPSATWLSQMLA
GGYQWLY-GLNSLLAGAVMGALWQVCVIFGLHWGFVPLMLNNFSV--
--IG
HDTLLPLLVPAVLGQ-AGATLGVLLRT---QDLKRKGIAGSAFSAAIF-GITEPAVYGVTLPLRRPFI-FGCIGGALGAAVMGYA-
-----HTTMYSFGFPSIFSFTQVIPPT----GVDSSVWAAVIGTLLAFAFAALTSWSFGVPKDETQPAAADSPAVLAETQANAGA
---VRDETL
FSPLAGEVLLLEQVADRTFASGVMGKGIAIRPTQGRLYAPVD-GTVA-SLFKTHHAIGLASRGGAEVLIHVGIDTVRLDGR----
----YFTPHVRVGDVVRQG-DLLLEFDGPAIEAAGYDLTTPIVITNS--EDYRGVEPVA--SGKVDANAPLTQLVC----------------
</seq-data>
</sequence>

<sequence seq-type="Protein">
<seq-name>q48408</seq-name>
<seq-info><length>1088</length></seq-info>
<seq-data>----MEYKALAQDILTRVGGRENIVSLVHCATRLRFKLKDSKKADAEGLKANPGVIMVVESGG-QFQVVIGN-H
VHDVWLAVRSEAGLTDDSEPVAVKG-------------EKVSLIGQLIDIVSGIFTPFIGVLAASGILKGLLALAVVCGWLTT----------
--QQATYKIWFAASDALFFF---FPLFLGYTAGKKF--------GGNPYITMVIGGALT---HPLMIQAFDASMAP--------------------
--GAATEYFLGIP--VTFINYSSSVIPIILASWVSCWIEKRSNAILPSSMK---------NFFTPAIC-LAVVVPLTFLIIGPVATWLSQLL
ANGYQLIY-QVAPWLAGAAMGALWQVCVIFGLHWGLIPLMINNLAV---
--LG
HDSMMPMLLPAVMGQ-VGAALGIFLRT---RDARQKVLAGSAVSAGIF-GVTEPAIYGLNLPLRRPFI-FGCVYWPIGGAMV
GFSDS----HVSTYSFGFGNIITLAQMIPPE----GIDATVWGGAAGMFASLIIACVLTLVAGLPRSSAEQAAVVAP------------
---ASVNDIL
APMTGSVLALDQVPDSTFASGLLGQGVAIIPSVGKVIAPFS-GEVA-SIFQTKHAIGLLSDSGIELLIHVGIDTVKLDGA--------
PFTAHVKEGDKIKAG-DLLLEFDRQAILDAGYDLATPIIISNS--DDFRTLDMVS--ASAVDAGQPLLSVSR----------------</se
q-data>
</sequence>
<sequence seq-type="Protein">
<seq-name>PTBA_ECOLI</seq-name>
<seq-info><length>1088</length></seq-info>
<seq-data>----MTE--LARKIVAGVGGADNIVSLMHCATRLRFKLKDESKAQAEVLKKTPGIIMVVESGG-QFQVVIGN-HV
ADVFLAVNSVAGL-DEKAQQAPEND------------DKGNLLNRFVYVISGIFTPLIGLMAATGILKGMLALALTFQWTTE--------
----QSGTYLILFSASDALFWF---FPIILGYTAGKRF--------GGNPFTAMVIGGALV---HPLILTAFENGQKA---------------------
-DALGLDFLGIP--VTLLNYSSSVIPIIFSAWLCSILERRLNAWLPSAIK---------NFFTPLLC-LMVITPVTFLLVGPLSTWISELIAA
GYLWLY-QAVPAFAGAVMGGFWQIFVMFGLHWGLVPLCINNFTV--
---LGY
DTMIPLLMPAIMAQ-VGAALGVFLCE---RDAQKKVVAGSAALTSLF-GITEPAVYGVNLPRKYPFV-IACISGALGATIIGYA--
----QTKVYSFGLPSIFTFMQTIPST----GIDFTVWASVIGGVIAIGCAFVGTVMLHFITAKRQPAQGAPQEKTPEVITP----------
---PEQGGICSP
MTGEIVPLIHVADTTFASGLLGKGIAILPSVGEVRSPVA-GRIA-SLFATLHAIGIESDDGVEILIHVGIDTVKLDGK--------FFSA
HVNVGDKVNTG-DRLISFDIPAIREAGFDLTTPVLISNS--DDFTDVLPHGTAQISA-GE-PLLSIIR----------------</seq-data
>
</sequence>
<sequence seq-type="Protein">
<seq-name>q46129</seq-name>
<seq-info><length>1088</length></seq-info>
<seq-data>----MKYEQLAKDILKNVGGKENVNSLTHCITRLRFKLKDEGKANTDILKNMDGVVTVVKSGG-QYQVVIGN-H
VPDVFAAVNAVGGLQGASEEDE-TE-------------EKMNPLNKFIDIVSGVFQPILGVLCATGMIKGLNAVLVAAGVLAS-------
-----TDSTYIILNAIGDCLFNF---FPIFLGFTAAKKF--------KLNQFTGMAVGAAMV---YPAITSFAG-----------------------------
QTVDFFGIPVVMPSSSYQSTVIPIILAIYIASKVEKLFKKIIPDMVK---------TFLVPFAT-LLVVVPVTFMAIGPISTIAANALGDL
TLAIY-NFNPTIAGLFIGGFWQVFVMFGLHWGLVPIAMNNLAV---
--IGYDPVL
ATSVAVCFAQ-TGVVMAILAKT---KDKKLKSLCIPAIISGFF-GVTEPAIYGITLPRKKPFI-LSCIAAGVTGGIIGFF------ESKGYS

MGGLGIFALPSYINPE----GIDRGFYGMVIAMVAGIVVGFILMFVTKLNDEEEVKTTESKKEESL--------------------------------
---VKQEEIVSPIQGEVVTLAEV
KDEAFSSGALGKGVAINPIEGKVYAPAD-GTLT-TLFPSLHALGITTENGAEILIHVGMDTVQLEGK--------HFTAKVKQGDKI
KKG-QLLIEFDKEAIEKAGYSTITPVLITNS--DQYLDVIETD--KRKVDVNSELLTVVI-----------------</seq-data>
</sequence>
<sequence seq-type="Protein">
<seq-name>ptsa_pedpe</seq-name>
<seq-info><length>1088</length></seq-info>
<seq-data>----MNHQEVADRVLNAIG-KNNIQAAAHCATRLRLVIKDESKIDQQALDDDADVKGTFETNG-QYQIIIGPGD
VDKVYDALIVKTGLKEVTPDDIKAVAAAG---------QNKNPLMDFLKVLSDIFIPIVPALVAGGLLMALNNVLTAEHLFMA---K
SVVEVYPGLKGIAEMINAMASAPFTF---LPILLGFSATKRF--------GGNPYLGATMGMIMV---LPSLVNGYSVATTMAAG--
------------------KMVYWNVFGLH--VAQAGYQGQVLPVLGVAFILATLEKFFHKHIKGAFD---------FTFTPMFA-IVITGFLTF
TIVGPVLRTVSDALTNGLVGLY-NSTGWIGMGIFGLLYSAIVITGLHQTFPAIETQLLAN--
----
-----------VAKTGGSFIFPVASMANIGQ-GAATLAIFFAT---KSQKQKALTSSAGVSALL-GITEPAIFGVNLKMKFPFV-FAAIA
SGIASAFLGLF------HVLSVAMG-PASVIGFISI--------ASKSIPAFMLSAVISFVVAFIPTFIYAKRTLGDDRDQVKSPAPTSTVI
N---
--VNDEIISAPVTGASESLKQVNDQVFSAEIMGKGAAIVPSSDQVVAPAD-GVIT-VTYDSHHAYGIKTTAGAEILIHLGLDTV
NLNGE--------HFTTNVQKGDTVHQG-DLLGTFDIAALKAANYDPTVMLIVTNT--ANYANVERL-KVTNVQAGE-QLVALTA
PAASSVAATTV</seq-data>
</sequence>
<sequence seq-type="Protein">
<seq-name>Q9S6S6</seq-name>
<seq-info><length>1088</length></seq-info>
<seq-data>----MNHKQVAERILNAVG-RDNIQGARHCATRLRLVLKDTGVIDQEALDNDPDLKGTFEAAG-QYQIIVGPGD
VNTVYEEFIKLTGISEASTADLKEIAGSQ---------KKQNPVMALVKLLSDIFVPLIPALVAGGLLMALNNALTAEHLFAT---KSL
VEMFPMWKGFADIVNTMSAAPFTF---MPILIGYSATKRF--------GGNPYLGAVVGMIMV---MPGLINGYNVAEAISNH---
-----------------TMTYWDIFGFK--VAQAGYQGQVLPVIGVAFILAKLERFFHKYLNDAID---------FTFTPLLS-VIITGFLTFTIV
GPALRFVSNGLTDGLVGLY-NTLGALGMLVFGGFYSAIVVTGLHQSFPAIETMLITN---

------YQHSGIGGDFIFPVAACANMAQ-AGATFAILFVT---KNIKTKALAAPAGVSAIL-GITEPALFGINLKLKYPFF-IALGASAI
GSLFMGLF------HVLAVSLG-SAGLIGFISI--------KAGYNLQFMISIFISFLIAFVVTSIYGRRMEAKSITKEKNKQNATTQYQP
EKVIIDP---
---VKSGELLAPINGFVIPLSDVSDPVFSKEIMGKGIAIKPKSGELFSPAD-GEII-IAYETGHAYGIKTKNGGEVLLHIGIDTVSMN
GN--------GFIQNVKVGQKVKAG-DLLGSFDKEEIKKSGLDDTVIIVITNS--ASYNEILPLSENVDIKVGE-KILLLN------------</
seq-data>
</sequence>
<sequence seq-type="Protein">
<seq-name>PTSA_STRMU</seq-name>
<seq-info><length>1088</length></seq-info>
<seq-data>----MDYSKVASEVITAVG-KDNLVAAAHCATRLRLVLKDDSKVDQKALDKNADVKGTFKTDG-QYQVIIGPGD
VNFVYDEIIKQTGLTEVSTDDLKKIAASG---------KKFNPIMALIKLLSDIFVPIIPALVAGGLLMALNNFLTSEGLFGT---KSLV

QQFPIIKGSSDMIQLMSAAPFWF---LPILVGISAAKRF--------GANQFLGASIGMIMV---APGAANIIGLAANAPIS-----------
--KAATIGAYTGFWNIFGLH--VTQASYTYQVIPVLVAVWLLSILEKFFHKRLPSAVD---------FTFTPLLS-VIITGFLTFIVIGPV
MKEVSDWLTNGIVWLY-DTTGFLGMGVFGALYSPVVMTGLHQSFPAIETQLISA---

----FQNGTGHGDFIFVTASMANVAQ-GAATFAIYFLT---KDKKMKGLSSSSGVSALL-GITEPALFGVNLKYRFPFF-CALIGSA
SAAAIAGLL------QVVAVSLG-SAGFLGFLSI--------KASSIPFYVVCELISFAIAFAVTYGYGKTKAVDVFAAEAAVEEAIEEVQ
EIPEEAASAANKAQ--
----------VTDEVLAAPLAGEAVELTSVNDPVFSSEAMGKGIAIKPSGNTVYAPVD-GTVQ-IAFDTGHAYGIKSDNGAEILIHIG
IDTVSMEGK--------GFEQKVQADQKIKKG-DVLGTFDSDKIAEAGLDNTTMFIVTNT--ADYASVETLASSGTVAVGD-SLLE
VKK-----------</seq-data>
</sequence>
<sequence seq-type="Protein">
<seq-name>ptsb_staxy</seq-name>
<seq-info><length>1088</length></seq-info>
<seq-data>----MNYKKSAENILQALGGEDNVEAMTHCATRLRLVLKDEGLVDEKALGDMDVVKGTFSTGG-QYQVIIGSG
TVNKVFSELEKITGKEASSVSEVKTQGT-----------KNMNPFQRFVKMLSDIFVPIIPAIVAGGLLMGINNILTAPGIFYDN--QS
LIEVQNQFSGLAEMINIFANAPFTL---LPILIGFSAAKRF--------GGNAYLGAALGMILV---HPELMSAYDYPKALEAG--------
-----------KEIPHWNLFGLE--INQVGYQGQVLPMLVATYILATIEKGLRKVIPTVLD---------NLLTPLLA-ILSTGFITFSFVGPLT
RTLGYWLSDGLTWLY-EFGGAIGGLIFGLLYAPIVITGMHHSFIAIETQLIAD--
---S
SSTGGSFIFPIATMSNIAQ-GAAALAAFFII--KENKKLKGVASAAGVSALL-GITEPAMFGVNLKLRYPFI-GAIVGSGIGSAYIA
FF------KVKAIALG-TAGIPGFISI--------SGQNNGWLHYGIAMIIAFIVAFGVTYALSYRKKYRNIEA----------------------------
----

----------------</seq-data>
</sequence>
<sequence seq-type="Protein">
<seq-name>ptsb_vibal</seq-name>
<seq-info><length>1088</length></seq-info>
<seq-data>----MNYPAVAKELLTLLGGKSNITALAHCATRLRLAVADEQKIDEQAIDNLEGVKGQFKVAG-QYQIIFGSGIV
NQVYAEMAKLTGMSEMSTNDVASAGA-----------EKQNIVQPAVKGLSDIFVPIIPAIVAGGLLMGIYNLLTAQGLFIDG--K
SLIEANPGLTDLANMINTFANAPFVY---LPILLAFSASKKF--------GGNPYLGAALGMLMV---HPDLLNGWGFGGASVSG--
------------------NIPVWNILGFE--IQKVGYQGSVLPVLVSAFILAKVELGLRKVIPSVLD---------NLLTPLLA-IFIAGLLTFTVVG
PFTRDIGFLLGDGLNWLY-NTAGFVGGAVFGLIYAPFVITGMHHSFIAIETQLLAD---

-----IATTGGTFIFPIAAMSNVSQ-GAAALAVGVMS---KDKKMKGIAIPSGVTGLL-GITEPAMFGVNLKLRYPFI-AAVCAAA
LSSAFITMF------NVKAQALG-AAGLPGIISI--------TPDKIGYYIAGMVIAFLTAFVLTIVLGIGDRAKVGKKAAA--------------
----

-----------------------------</seq-data>
</sequence>
<sequence seq-type="Protein">

<seq-name>PTTB_BACSU</seq-name>
<seq-info><length>1088</length></seq-info>
<seq-data>---MGELNKSARQIVEAVGGAENIAAATHCVTRLRFALIDESKVDQEMLDQIDVVKGSFSTNG-QFQVVIGQG
TVNKVYAELVKETGIGESTKDEVKKASE-----------KNMNPLQRAVKTLADIFIPILPAIVTAGLLMGINNILTAEGISFST--KSIV
QVYPQWADLANMINLIAGTAFTF---LPALIGWSAVKRF--------GGNPLLGIVLGVMLV---HPDLLNAWGYGAAEQSG-----
---------------EIPVWNLFGLE--VQKVGYQGQVLPILLASYMLAKIEVFLTKRTPEGIQ---------LLVVAPIT-LLLTGFASFIIIGPIT
FAIGNVLTSGLISVF-GSFAALGGLLYGGFYSALVITGMHHTFLAVDLQLIGS---

KLGGTFLWPMLALSNIAQ-GSAALALMFIV---KDEKQKGLSLTSGISAYL-GITEPAIFGVNLRYRFPFI-IAMVSSGLAGMYIS
SQ------GVLASSVG-VGGVPGIFSI--------MSQYWGAFAIGMAIVLIVPFAGTYAYGRFKHK------------------------------------
----

-----------------</seq-data>
</sequence>
<sequence seq-type="Protein">
<seq-name>PTTB_ECOLI</seq-name>
<seq-info><length>1088</length></seq-info>
<seq-data>-MMSKINQTDIDRLIELVGGRGNIATVSHCITRLRFVLNQPANARPKEIEQLPMVKGCFTNAG-QFQVVIG-TN
VGDYYQALIASTGQAQVDKEQVKKAAR-----------HNMKWHEQLISHFAVIFFPLLPALISGGLILGFRNVIGDLPMSNG---Q
TLAQMYPSLQTIYDFLWLIGEAIFFY---LPVGICWSAVKKM--------GGTPILGIVLGVTLV---SPQLMNAYLLGQQLP---------
---------------EVWD-FGMFS-IAKVGYQAQVIPALLAGLALGVIETRLKRIVPDYLY---------LVVVPVCS-LILAVFLAHALIGPF
GRMIGDGVAFAVRHLMTGSFAPIGAALFGFLYAPLVITGVHQTTLAIDLQMIQS---

-------MGGTPVWPLIALSNIAQ-GSAVIGIIISS---RKHNEREISVPAAISAWL-GVTEPAMYGINLKYRFPML-CAMIGSGLAG
LLCGLN------GVMANGIG-VGGLPGILSI--------QPSYWQVFALAMAIAIIIPIVLTSFIYQRKYRLGTLDIV----------------------
----

------------------------</seq-data>
</sequence>
<sequence seq-type="Protein">
<seq-name>q9zhj9</seq-name>
<seq-info><length>1088</length></seq-info>
<seq-data>----MKEQIVAKEILENIGGKENIKSVEHCATRLRLILNDKEKINEKAIENIDGVKGQFFSAA-QYQIILGTGFVNEV
YDVIVGQNSDLVTGNNKEEAY-------------SQMTLIQKISRTFGDVFVPIIPVLVATGLFMGLRGLLTNLGVQMN-------------
ENFVLFTQVLTDTAFAF---LPALVAWSTMKKF--------GGTPVIGIVIGLMLV---SPSLPNAYAVAAGT----------------------AT
PINLTILGLN--IPVVGYQGSVLPALVLGIIAAKTQKALKKVVPDVLD---------LIVTPFIT-LLFSMVLGLLIVGPIMHNAEQLIFG
AIKGFM-GLPFGLGGLVVGGVHQLIVVTGVHHALNALEVELLSS---
--TGKDAF
NAMITCGIVAQ-GAAALAVAVKT---KDKKKRSLYISSAIPAFL-GITEPAIFGVNLRFIKPFI-FGCAGGAVGGMLSGIL------HL
AGTGMG-ITALPGMLLY---------VNNLGSYILVNVVAIAVAFCLTLFFFKPEE--

-</seq-data>
</sequence>
<sequence seq-type="Protein">
<seq-name>p94470</seq-name>
<seq-info><length>1088</length></seq-info>
<seq-data>----MNYERVAKQLIPLLGGKENIISAVHCATRLRLVLKDDTKADTKAIENIEGVKGAFSSSG-QYQIIFGTGVVNK
VYDSFVQEAGLQHVSADVHQEAVK-----------QKMNPLARLAKTLSNIFVPIIPAIVASGLLMGPLGMMKAFKWVAP-------
-----DSPLYVLLDMFSSAAFII---LPILIGFSAAKEF--------GGTPFLGAVIGGIMT---HPALLNPWGLAEAK----------------------
--PNYMHFLGFD--IAMVGYQGTVVPILLATYVMCKIERGLRNVVPHTVS---------LLVVPFVT-VISTGFITILAIGPLGNLLGD
GITTVLNFIY-HYGGALAGLIFGGLYSMIVITGVHHSFHAIEANLLAK---
---LGVN
YLLPIWSMANVAQ-GGAGLAVFVKA---KSLKTKEVALPSALSAFL-GITEPVIFGVNLKYRKPFI-AAAIGGALGGAYVVFT----
--NVVANAYG-LTGIPMIANRGARRNA--KPHQLFNWICDCGRIGVYCHVIAWISRGRINKAPDNR-----------------------------
----

---------------------</seq-data>
</sequence>
<sequence seq-type="Protein">
<seq-name>ptsb_klepn</seq-name>
<seq-info><length>1088</length></seq-info>
<seq-data>----MDFEQISRSLLPLLGGKENIASAAHCATRLRLVLVDDALADQQAIGKIDGVKGCFRNAG-QMQIIFGTGVV
NKVYAAFIQAAGISESSKSEAADLAA-----------KKLNPFQRIARLLSNIFVPIIPAIVASGLLMGLLGMVKTYGWVDP----------
--SNALYIMLDMCSSAAFII---LPILIGFTAAREF--------GGNPYLGATLGGILT---HPALTNAWGVAAG--------------------------
FHTMNFFGIE--VAMIGYQGTVFPVLLAVWFMSMVEKRLRRVIPDALD---------LILTPFLT-VIISGFIALLLIGPAGRALGDGI
SFILSTLI-SHAGWLAGLLFGGLYSVIVITGIHHSFHAIEAGLLGN--
---PSIGVNFLL
PIWAMANVAQ-GGACFAVWFKT---KDAKIKAITLPSAFSAML-GITEAAIFGINLRFVKPFI-AALVGGAAGGAWVVSM----
--HVYMTAVG-LTAIPGMAIV--------QASSLLNYIIGMAIAFAVAFALSLTLKYKTDAE---
----

--------</seq-data>
</sequence>
<sequence seq-type="Protein">
<seq-name>ptsb_salty</seq-name>
<seq-info><length>1088</length></seq-info>
<seq-data>----MDFEQISCSLLPLLGGKENIASAAHCATRLRLVLVDDSLADQQAIGKVEGVKGCFRNAG-QMQIIFGTGVV
NKVYAAFTQAAGISESSKSEAADIAA-----------KKLNPFQRIARLLSNIFVPIIPAIVASGLLMGLLGMVKTYGWVDP----------
--GNAIYIMLDMCSSAAFII---LPILIGFTAAREF--------GGNPYLGATLGGILT---HPALTNAWGVAAG--------------------------
FHTMNFFGFE--IAMIGYQGTVFPVLLAVWFMSIVEKQLRRAIPDALD---------LILTPFLT-VIISGFIALLIIGPAGRALGDGIS
FVLSTLI-SHAGWLAGLLFGGLYSVIVITGIHHSFHAVEAGLLGN---
--PSIGVNFLL
PIWAMANVAQ-GGACLAVWFKT---KDAKIKAITLPSAFSAML-GITEAAIFGINLRFVKPFI-AALIGGAAGGAWVVSV------

HVYMTAVG-LTAIPGMAIV--------QASSLLNYIIGMVIAFGVAFTVSLVLKYKTDAE---
----

-------</seq-data>
</sequence>
<sequence seq-type="Protein">
<seq-name>sacx_bacsu</seq-name>
<seq-info><length>1088</length></seq-info>
<seq-data>-----MHKEIAKELLLLAGGKNNIISISHCTTRLRFDVKDETKIDIHAIENLQGVQGTFFRYG-LFQIIFGAGVVNKIY
KEVVHVWETAPSEEPVHQKKAS-----------RKLNPAAAFAKTLSDIFVPIIPAITASGLLMGLIGMIKVFHWFAA------------GS
PWIKMLDLVSSTAFIL---LPILVGFSAARQF--------GSNPYLGAVIAGLLT---HPDLLDPSMLGSKT-----------------------PSSL
DIWGLH--IPMMGYQGSMIPILLSVFVMSKIEKLLKSIVPKSLD---------VVIIPFIT-VMVTGCLALIVMNPAASIIGQIMTQSI
VYIY-DHAGIAAGALFGGIYSTIVLSGLHHSFYAIEATLLAN--
--PHVGVNFLVPI
WSMANVAQ-GGAGLAVFLKT---KQSSLKKIALPASLTAFL-GIVEPIVFGVNLKLIRPFI-GAAIGGAIGGAYVVAV------QVV
ANSYG-LTGIPMISIVLPF-----GAANFVHYMIGFLIAAVSAFIATLFLGFKEETE---

</seq-data>
</sequence>
<sequence seq-type="Protein">
<seq-name>ptsb_bacsu</seq-name>
<seq-info><length>1088</length></seq-info>
<seq-data>----MDYKETAKRLIELLGGKENIISAAHCATRLRLVMKDESKIDQAQVEELDGVKGAFSSSG-QYQIIFGTGLVN
KVFDAFSKEADIEREEHVNHQDAAK-----------EKLNPAARFAKTLSNIFVPIIPAIVASGLLMGLLGMINAFHWMSK---------
---DSALLQLLDMFSSAAFIF---LPILIGVSASKEF--------GSNPYLGAVIGGIMI---HPNLLNPWGLAEEQ------------------------
LITCIFSDL---ISLFSATGNCYPCPACGVCDEQGREMDEKSGSTCGD---------LLVTPFVT-VIVTGFVAFIAIGPLGRALGSGIT
VALTYVY-DHAGFVAGLIFGGTYSLIVLTGVHHSFHAIEAGLIAD---
---IGKNYLL
PIWSMANVAQ-GGAGLAVFFMA---KKAKTKEIALPAAFSAFL-GITEPVIFGVNLRYRKPFI-AAMIGGALGGAYVVFT------
HVAANAYG-LTGIPMIAIAAPF-----GFSNLIHYLIGMAIAAVSAFIAAFVMKINEDEERKK--------------------------------------
----

----------</seq-data>
</sequence>
<sequence seq-type="Protein">
<seq-name>PTBA_BACSU</seq-name>
<seq-info><length>1088</length></seq-info>
<seq-data>----MDYDKLSKDILQLVGGEENVQRVIHCMTRLRFNLHDNAKADRSQLEQLPGVMGTNISGE-QFQIIIGN-D
VPKVYQAIVRHSNLSDEKSAGSSS--------------QKKNVLSAVFDVISGVFTPILPAIAGAGMIKGLVALAVTFGWMAE--------
----KSQVHVILTAVGDGAFYF---LPLLLAMSAARKF--------GSNPYVAAAIAAAIL---HPDLTALLGAGKP----------------------
-----ISFIGLP--VTAATYSSTVIPILLSIWIASYVEKWIDRFTHASLK---------LIVVPTFT-LLIVVPLTLITVGPLGAILGEYLSSGVN

YLF-DHAGLVAMILLAGTFSLIIMTGMHYAFVPIMINNIAQ--
---NGHDYLLPA
MFLANMGQ-AGASFAVFLRS---RNKKFKSLALTTSITALM-GITEPAMYGVNMRLKKPFA-AALIGGAAGGAFYGMT------
GVASYIVGGNAGLPSIPVFI--------GPTFIYAMIGLVIAFAAETAAAYLLGFEDVPSDGSQQPAVHE---------------------------
--GSREIIHSPIKGEV
KALSEVKDGVFSAGVMGKGFAIEPEEGEVVSPVR-GSVT-TIFKTKHAIGITSDQGAEILIHIGLDTVKLEGQ--------WFTAHIK
EGDKVAPG-DPLVSFDLEQIKAAGYDVITPVIVTNT--DQYSFS-PVKEIGKVQPKE-ALLALS------------</seq-data>
</sequence>
<sequence seq-type="Protein">
<seq-name>p77272</seq-name>
<seq-info><length>1088</length></seq-info>
<seq-data>-MAKEISSELLNTILTRVGGPGNIASCGNCMTRLRLGVHDSSLVD-PNIKTLEGVKGVILTSD-QVQVVFGPGKA
HRAAKAMSELLGEAPVQDAAEIAAQNKRQLKA-----KQTSGVQQFLAKFATIFTPLIPGFIAAGLLLGIATLIATVMHVPA----
---DAQGTLPDALNFMKVFSKGLFTF---LVILVGYNAAQAF--------GGTGVNGAIIAALFLLGYNPAATTGYYAG-------------
--------------FHDFFGLP-----IDPRGNIIGVLIAAWACARIEGMVRRFMPDDLD---------MLLTSLIT-LLITATLAYLIIMPLGG
WLFEGMSWLFMHL--NSNPFGCAVLAG-LFLIAVVFGVHQGFIPVYLALMDS---

----QGFNSLFPILSMAGAGQ-VGAALALYWRA--QPHSALRSQVRGAIIPGLL-GVGEPLIYGVTLPRMKPFV-TACLGGAAG
GLFIGLIAWWGLPMGLNSAFG-PSGLVALPLMTSA----QGILPAMAVYAGGILVAWVCGFIFTTLFGCRNVNLD------------
----

-----------------------------------</seq-data>
</sequence>
<sequence seq-type="Protein">
<seq-name>Q45579</seq-name>
<seq-info><length>1088</length></seq-info>
<seq-data>MSKENNYHHLAQKILELCGGKSNISSYTHCMTRLRITPYDESKADAQALRKLDGVLGVVEAET--LQIILGTGVV
NHVTAAFSKLVSAEEGADVKEAAAKKKADINR-----KNATPFKLFLRKIASIFIPLIPALVASGLITGITKAIIQAGWLSA----------
--DSQIAIILTVIGSGLFTY---LGILVGINASKEF--------GGTPALGALAGILII---NPEIAKI---------------------------------SLFGE
E----LLPGRGGLIGVLFAAIFIAYTEQFIRRFIPQSLD---------IIVTPTVS-LLITGIVTYVVFMPLGGFISDAIASGLLSIL-DIGGIA
AGFILGATFLPLVVTGLHQGLTPMHMELINS--
--IGNDPLLPILAMGGAGQ-V
GAAFAVFVKT---KKTTLRKAIAGGLPSGLL-GIGEPLIFGVTLPLGRPVF-NSMFRSWNRRRLSSLF------SSSHHCDW-CLRTA
AIF-----------SSPAFANHPVYRWALYFLRGRFFIDLCIRI--

--</seq-data>
</sequence>
<sequence seq-type="Protein">
<seq-name>PTDA_ECOLI</seq-name>
<seq-info><length>1088</length></seq-info>
<seq-data>--MAKNYAALARSVIAALGGVDNISAVTHCMTRLRFVIKDDALIDSPTLKTIPGVLGVVRSDN-QCQVIIGN-TV
SQAFQEVVSLLPGDMQPAQPVGKPKL-----------TLRRIGAGILDALIGTMSPLIPAIIGGSMVKLLAMILEMSGVLTK---------

---GSPTLTILNVIGDGAFFF---LPLMVAHLPRIKF--------KTNMSLAIAIAGVLV---HPSFIELMAKAAQG-----------------------
--EHVEFALIP--VTAVKYTYTVIPALVMTWCLSYIERWVDSITPAVTK---------NFLKPMLI-VLIAAPLAILLIGPIGIWIGSAISA
LVYTIH-GYLGWLSVAIMGALWPLLVMTGMHRVFTPTIIQTIAE--
--TGKEGM
VMPSEIGANLSL-GGSSLAVAWKT--KNPELRQTALAAAASAIMA-GISEPALYGVAIRLKRPLI-ASLISGFICGAVAGMA-----
-GLASHSMAAPGLFTSVQFFDP-------ANPMSIVWVFAVMALAVVLSFILTLLLGFEDIPVEEAAAQARKYQSVQPTVAKEV
SLN---

-------------------------------</seq-data>
</sequence>
<sequence seq-type="Protein">
<seq-name>ptcc_ecoli</seq-name>
<seq-info><length>1088</length></seq-info>
<seq-data>---MSNVIASLEKVLLPFAVKI
GKQPHVNAIKNGFIRLMPLTLAGAMFVLINNVFLSFGEGSFFYSLGIRLDASTIETLNGLKGIGGNVYNGTLGIMSLMAPFFI
GMALAEERKV----DALAAGLLSVAAFMT---VTPYSVGEAY--------------------------------AVGANWLGGANIISGIIIGLVVA
EMFTFIVRRNWVIKLPDSVPASVSRSFSALIPGFIIL-SVMGIIAWALNTWGTNFHQIIMDTISTPLAS-L-GSVVG-WAYVIFV
PLLWFFGIHGALALTALDNGIMTPWALENIATYQQYGSVEAALAAGKTF--
---HIWAKPMLDSFIFLG
G-SGATLGLILAIFIASRRADYRQVAKLALPSGIFQINEPILFGLPIIMNPVMF-IPFVLVQPILAAITLAAYYMGIIPPVTNIA-PW
TMPTGLGAFF-----NTNGSVAALLVALFNLGIATLIYLPFVVVANKAQNAIDKEESEEDIANALKF------------------------------
----

---</seq-data>
</sequence>
<sequence seq-type="Protein">
<seq-name>PTCC_BACST</seq-name>
<seq-info><length>1088</length></seq-info>
<seq-data>---MDRFIRMLEDRVMPVAG
KIAEQRHLQAIRDGIILSMPLLIIGSLFLIVGFLPIPGYNEWMAKW----------FGEHWLDKLLYPVGATFD-IMALVVSFGVAY
RLAEKYKV----DALSAGAISLAAFLL---ATPYQVPFTPEGAKETIMVSG---------------------GIPVQWVGSKGLFVAMILAIVS
TEIYRKIIQKNIVIKLPDGVPPAVARSFVALIPGAAVLVVVWVARLILEMTPFESFHNIVSVLLNKPLSV-LGGSVFGAIVAVLLV
QLLWSTGLHGAAIVGGVMGPIWLSLMDENRMVFQQNPNAEL---
---PNVITQQFFDLWIYIGG
-SGATLALALTMMFRARSRQLKSLGRLAIAPGIFNINEPITFGMPIVMNPLLI-IPFILVPVVLVVVSYAAMATGLVAKPSGVA
VPWTTPIVISGYL-----ATGGKISGSILQIVNFFIAFAIYYPFFSIWDKQKAAEEQADPTISSGAGATHSL--------------------------
----

------</seq-data>
</sequence>
<sequence seq-type="Protein">
<seq-name>PTLB_LACLA</seq-name>

<seq-info><length>1088</length></seq-info>
<seq-data>---MHKLIELIEKG-KPFFEKIS
RNIYLRAIRDGFIAGMPVILFSSIFILIAYVPNA-------------------WGFHWSKDIETFLMTPYS-YSMGILAFFVGGTTAKALTD
SKNRDLPATNQINFLSTML---ASMVGFLLMAAEPAKEG--------------------------GFLTAFMGTKGLLTAFIAAFVTVNVYKV
CVKNNVTIRMPEDVPPNISQVFKDLIPFTVSV-VLLYGLELLVKGTLGVTVAESIGTLIAPLFS-AADGYLGITLIFGAYAFFWFV
GIHGPSIVEPAIAAITYANIDVNLHLIQAGQHA---
---DKVITSGTQMFIATMGG-TGATLIVPFLF
MWICKSDRNRAIGRASVVPTFFGVNEPILFGAPIVLNPIFF-VPFIFAPIVNVWIFKFFVDTLNMNSFSANL-PWVTPGPLGIV
L-----GTNFQVLSFILAGLLVVVDTIIYYPFVKVYDEQILEEERSGKTNDALKEKVAANFNTAKADAVLGKADVAKEDVAANN
NITKETNVLVLCAGGGTSGLLANALNKAAAEYNVPVKAAAGGYGAHREMLPEFDLVILAPQVASNFDDMKAETDKLGIKL
VKTEGAQYIKLTRDGQGALAFVQQQFD---
--</seq-data>
</sequence>
<column-score>
<colsco-name>coreblock</colsco-name>
<colsco-owner>0</colsco-owner>
<colsco-type>int</colsco-type>
<colsco-data>0 0 0 0 0 0 0 0 0 0 0 0 0 0 0 0 0 0 0 0 0 0 0 0 0 0 0 0 0 0 0 0 0 0 0 0 0 0 0 0 0 0 0 0 0 0 0 0 0 0 0 0
0 0 0 0 0 0 0 0 0 0 0 0 0 0 0 0 0 0 0 0 0 0 0 0 0 0 0 0 0 0 0 0 0 0 0 0 0 0 0 0 0 0 0 0 0 0 0 0 0 0 0 0 0 0 0 0 0 0 0
0 0 0 0 0 0 0 0 0 0 0 0 0 0 0 0 0 0 0 0 0 0 0 0 0 0 0 0 0 0 0 0 0 0 0 0 0 0 0 0 0 0 0 0 0 0 0 0 0 0 0 0 0 0 0 0 0 0 0
0 0 0 0 0 1 1 1 1 1 1 1 1 1 1 0 0 0 0 0 0 1 1 1 1 1 1 1 0 0 0 0 0 0 0 0 0 0 0 0 0 0 0 0 0 0 0 0 0 0 0 0 0 0 0 0 0 0 0
0 0 0 0 0 0 0 0 0 0 0 0 0 0 0 0 0 0 0 0 0 0 0 0 0 0 0 0 0 0 0 0 0 0 0 0 0 0 0 0 0 0 0 0 0 0 0 0 0 0 0 0 0 0 0 0 0 0 0
0 0 0 0 0 0 0 0 0 0 0 0 0 0 0 1 1 1 1 1 1 1 1 1 0 0 0 0 0 0 0 0 0 0 0 0 0 0 0 0 0 0 0 0 0 0 0 0 0 0 0 0 0 0 0 0 0 0 0
0 0 0 0 0 0 0 0 0 0 0 0 0 0 0 0 0 0 0 0 0 1 1 1 1 1 1 1 1 1 1 1 1 1 1 1 1 1 1 1 1 1 1 0 0 0 0 0 0 0 0 0 0 0 0 0 0 0 0
0 0 0 0 0 0 0 0 0 0 0 0 0 0 0 0 0 0 0 0 0 0 0 0 0 0 0 0 0 0 0 0 0 0 0 0 0 0 0 0 0 0 0 0 0 0 0 0 0 0 0 0 0 0 0 0 0 0 0
0 0 0 0 0 0 0 0 0 0 0 0 0 0 0 0 0 0 0 0 0 0 0 0 0 0 0 0 0 0 0 0 0 0 0 0 0 0 0 0 0 0 0 0 0 0 0 0 0 0 0 0 0 0 0 0 0 0 0
0 0 0 0 0 0 0 0 0 0 0 0 0 0 0 0 0 0 0 0 0 0 0 0 0 0 0 0 0 0 0 0 0 0 0 0 0 0 0 0 0 0 0 0 0 0 0 0 0 0 0 0 0 0 0 0 0 0 0
0 0 0 0 0 0 0 0 0 0 0 0 0 0 0 0 0 1 1 1 1 1 1 1 1 1 1 0 0 0 0 0 0 0 0 1 1 1 1 1 1 1 1 1 1 0 0 0 0 0 0 0 0 0 0 0 0 0 0
0 0 0 0 0 0 0 0 0 1 1 1 1 1 1 1 1 1 1 1 0 0 0 0 0 0 0 0 0 0 0 0 0 0 0 0 0 0 0 0 0 0 0 0 0 0 0 0 0 0 0 0 0 0 0 0 0 0 0
0 0 0 0 0 0 0 0 0 0 0 0 0 0 0 0 0 0 0 0 1 0 0 0 0 0 0 0 0 0 0 0 0 0 0 0 0 0 0 0 0 0 0 0 0 0 0 0 0 0 0 0 0 0 0 0 0 0 0
0 0 0 0 0 0 0 0 0 0 0 0 0 0 0 0 0 0 0 0 0 0 0 0 0 0 0 0 0 0 0 0 0 0 0 0 0 0 0 0 0 0 0 0 0 0 0 0 0 0 0 0 0 0 0 0 0 0 0
0 0 0 0 0 0 0 0 0 0 0 0 0 0 0 0 0 0 0 0 0 0 0 0 0 0 0 0 0 0 0 0 0 0 0 0 0 0 0 0 0 0 0 0 0 0 0 0 0 0 0 0 0 0 0 0 0 0 0
0 0 0 0 0 0 0 0 0 0 0 0 0 0 0 0 0 0 0 0 0 0 0 0 0 0 0 0 0 0 0 0 0 0 0 0 0 0 0 0 0 0 0 0 0 0 0 0 0 0 0 0 0 0 0 0 0 0 0
0 0 0 0 0 0 0 0 0 0 0 0 0 0 0 0 0 0 0 0 0 0 0 0 0 0 0 0 0 0 0 0 0 0 0 0 0 0 0 0 0 0 0 0 0 0 0 0 0 0 0 0 0 0 0 0 0 0 0
0 0 0 0 0 0 0 0 0 0 0 0 0 0 0 0 0 0 0 0 0 0 0 0 0 0 0 0 0 0 0 0 0 0 0 0 0 0 0 0 0 0 0 0 0 0 0 0 0 0 0 0 0 0 0 0 0 0 0
0 0 0 0 0 0 0 0 0 0 0 0 0 0 0 0 0 0 0 0 0 0 0 0 0 0 0 0 0 0 0 0 0 </colsco-data>
</column-score>
</alignment>
</macsim>
