# Supplementary material for: Accurate multiple sequence alignment of transmembrane proteins with PSI-Coffee
Source: BMC Bioinformatics. 2012 Mar 28;13(Suppl 4):S1. doi: 10.1186/1471-2105-13-S4-S1 (PMC3303701; doi:10.1186/1471-2105-13-S4-S1)
Supplement: Additional file 2 — The performance of each TMP family by individual database. default means T-Coffee without homology extension. Others are PSI-Coffee searching against corresponding databases. The construction of databases is explained in "Methods" section. [file 1471-2105-13-S4-S1-S2.pdf]

The performance of each TMP family by individual database

default means T-Coffee without homology extension. Others are PSI-Coffee searching against corresponding databases. The construction of databases is explained in "Methods" section.

mode	default	UniRef50-TM	UniRef90-TM	UniRef100-TM	UniProt-TM	UniRef50	UniRef90	UniRef100	UniProt	NR
SP										
7TM	0.977	0.983	0.983	0.985	0.985	0.982	0.985	0.985	0.985	0.986
Nat	0.740	0.787	0.782	0.787	0.789	0.807	0.797	0.780	0.790	0.779
ACR	0.989	0.991	0.991	0.987	0.985	0.991	0.993	0.993	0.992	0.992
DTD	0.965	0.974	0.975	0.974	0.975	0.974	0.978	0.978	0.977	0.977
ION	0.785	0.753	0.756	0.797	0.797	0.754	0.782	0.783	0.785	0.783
MSL	0.965	0.986	0.971	0.978	0.978	0.986	0.971	0.971	0.978	0.971
PHOTO	0.955	0.955	0.956	0.954	0.954	0.954	0.954	0.955	0.954	0.955
PTGA	0.908	0.900	0.927	0.935	0.921	0.911	0.928	0.928	0.920	0.926
AVG	0.911	0.916	0.918	0.925	0.923	0.920	0.924	0.922	0.923	0.921
TC										
7TM	0.520	0.650	0.550	0.610	0.580	0.540	0.590	0.610	0.610	0.620
Nat	0.130	0.240	0.240	0.240	0.240	0.240	0.310	0.250	0.310	0.250
ACR	0.830	0.870	0.860	0.790	0.760	0.880	0.900	0.890	0.880	0.880
DTD	0.470	0.610	0.640	0.610	0.600	0.620	0.640	0.630	0.610	0.620
ION	0.210	0.210	0.240	0.240	0.240	0.210	0.210	0.210	0.240	0.210
MSL	0.910	0.960	0.930	0.950	0.950	0.960	0.930	0.930	0.950	0.930
PHOTO	0.530	0.520	0.520	0.520	0.520	0.510	0.510	0.510	0.500	0.520
PTGA	0.380	0.430	0.400	0.400	0.400	0.460	0.400	0.400	0.400	0.400
AVG	0.498	0.561	0.548	0.545	0.536	0.553	0.561	0.554	0.563	0.554
